# Structural Adaptation of the Single-Stranded DNA-Binding Protein C-Terminal to DNA Metabolizing Partners Guides Inhibitor Design

**DOI:** 10.3390/pharmaceutics15041032

**Published:** 2023-03-23

**Authors:** Attila Tököli, Brigitta Bodnár, Ferenc Bogár, Gábor Paragi, Anasztázia Hetényi, Éva Bartus, Edit Wéber, Zsófia Hegedüs, Zoltán Szabó, Gábor Kecskeméti, Gerda Szakonyi, Tamás A. Martinek

**Affiliations:** 1 Department of Medical Chemistry, University of Szeged, H6720 Szeged, Hungary; tokoli.attila@med.u-szeged.hu (A.T.);; 2 ELKH-SZTE Biomimetic Systems Research Group, Eötvös Loránd Research Network (ELKH), H6720 Szeged, Hungary; 3 Institute of Physics, University of Pécs, H7624 Pécs, Hungary; 4 Department of Theoretical Physics, University of Szeged, H6720 Szeged, Hungary; 5 Institute of Pharmaceutical Analysis, University of Szeged, H6720 Szeged, Hungary

**Keywords:** intrinsically disordered proteins, single-stranded DNA-binding protein, peptide, non-natural amino acid, antimicrobial

## Abstract

Single-stranded DNA-binding protein (SSB) is a bacterial interaction hub and an appealing target for antimicrobial therapy. Understanding the structural adaptation of the disordered SSB C-terminus (SSB-Ct) to DNA metabolizing enzymes (e.g., ExoI and RecO) is essential for designing high-affinity SSB mimetic inhibitors. Molecular dynamics simulations revealed the transient interactions of SSB-Ct with two hot spots on ExoI and RecO. The residual flexibility of the peptide–protein complexes allows adaptive molecular recognition. Scanning with non-canonical amino acids revealed that modifications at both termini of SSB-Ct could increase the affinity, supporting the two-hot-spot binding model. Combining unnatural amino acid substitutions on both segments of the peptide resulted in enthalpy-enhanced affinity, accompanied by enthalpy–entropy compensation, as determined by isothermal calorimetry. NMR data and molecular modeling confirmed the reduced flexibility of the improved affinity complexes. Our results highlight that the SSB-Ct mimetics bind to the DNA metabolizing targets through the hot spots, interacting with both of segments of the ligands.

## 1. Introduction

Drug-resistant bacterial infections will remain a focal drug development issue in the coming decade [1,2,3,4,5]. New classes and modes of action are required to tackle the pathogens escaping rapidly aging antibiotics. Disrupting the interactions of the eubacterial single-stranded DNA-binding protein (SSB) with the proteins responsible for genome maintenance (SSB interacting proteins, SIP) has emerged as a promising mechanism [6,7,8]. SSB–SIP interactions are present in both Gram-positive and-negative species, and the sizeable interactome of SSB [9,10,11,12,13,14,15,16,17,18,19,20] renders this target advantageous in fighting resistance development. Despite these attractive features, this mode of action has remained therapeutically untapped.

**Figure 1:**
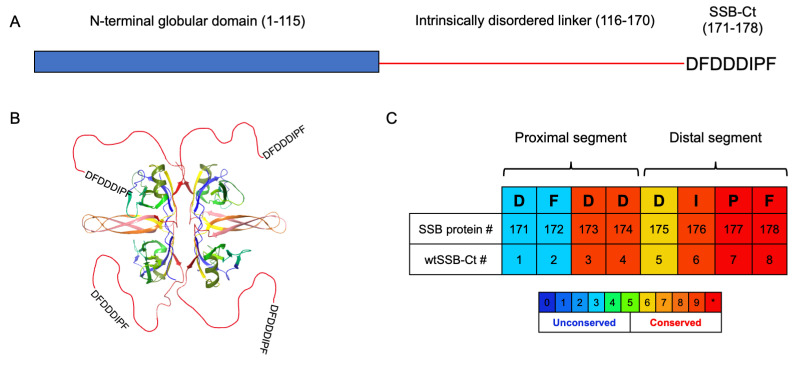
(**A**) Domain structure of *E. coli* SSB protein. (**B**) Structure of *E. coli* single-stranded-DNA binding protein as a tetramer (PDB structure 4MZ9). The IDP regions are red lines, and the acidic tips are shown as amino acid sequences. (**C**) SSB-Ct sequence and its numbering as a segment of the whole protein. wtSSB-Ct is an octapeptide with a sequence identical to the SSB-Ct segment. Sequences are colored according to the conservation scores (see lower part of panel (**C**), * denotes the highest score) obtained from PRALINE [31].

SSB consists of an N-terminal globular domain and a C-terminal intrinsically disordered protein (IDP) region responsible for the SSB–SIP interactions (Figure 1A). Within the IDP region, the PXXP motifs and the C-terminal acidic tip (SSB-Ct) govern the contacts with the SIPs. PXXP motifs bind to the oligonucleotide-binding (OB) folds in another SSB or in SIP proteins [21], and these interactions are crucial for the cooperative binding to single-stranded DNA (ssDNA) [22] and the regulation of the SSB interactome [23,24,25]. SSB-Ct (residues 171–178 of SSB) is an essential segment for SIP recognition and a highly conserved sequence among all eubacteria (Figure 1A,C and Figure S1) [26]. The octapeptide consists of a proximal (DFDD) and distal (DIPF) segment. The C-terminal Ile-Pro-Phe (IPF) motif of SSB-Ct is required for proper functioning [21,27,28]. The affinity of the SSB-Ct–SIP interactions is also determined by the abundant Asp side chains and Phe172 [29]. Although conservation scores and deletion/mutation studies have clarified the biological importance of the acidic tip, X-ray measurements have only revealed binding sites and geometries for the distal IPF or DIPF tail [10,11]. The structural background of the interaction between the proximal DFDD motif of SSB-Ct and SIPs is puzzling, but X-ray results support that the SSB-Ct is highly sensitive to the structuring effects of its interaction partner [30].

**Figure 2:**
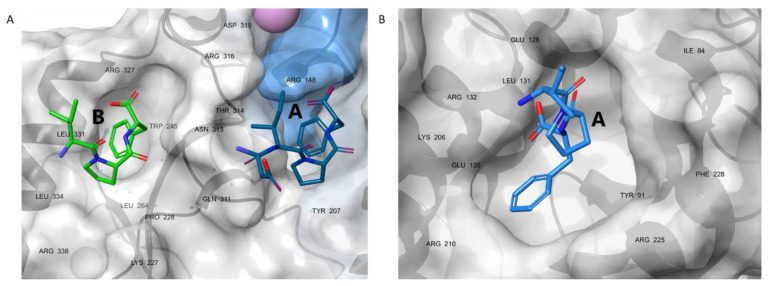
(**A**) The resolved C-terminal residues of wtSSB-Ct peptide (sticks) bound to sites A (DIPF) and B (IPF) of ExoI and their critical interacting residues (PDB structure 3C94). (**B**) Resolved C-terminal IPF segment of wtSSB-Ct peptide (sticks) bound to RecO (PDB structure 3Q8D). Interacting residues are labeled black. ExoI and RecO appear in the surface representation.

Small molecule mimetics of the IPF motif have been reported to inhibit the interactions of SSB with ExoI [6,7], DnaG [32], and PriA [33,34]. These efforts led to inhibitors with inhibitory concentrations (IC_50_) at around 1 µM or higher values, which leaves room for improvement. The low affinities accord with the limited and solvent-exposed contact surface available for small molecules on the SIP proteins (Figure 2) [10,11,35]. For ExoI, the crystal structures [6] showed a secondary binding site for SSB-Ct at the SH3 domain, which does not play a primary role in enzyme activation (Figure 2). However, this site offers the possibility for an additional hot spot, to stabilize the binding of a peptidomimetic ligand.

We hypothesized that certain SIPs display surface features near the confirmed IPF binding sites, accessible using the proximal DFDD motif. We set out to test the role of these secondary hot spots for SIPs ExoI and RecO, which are highly conserved across eubacterial species (Figure S1). Our data revealed that SSB-Ct could simultaneously land DIPF and DFDD motifs onto SIPs, and the binding involved a separate hot spot beside the confirmed IPF-binding site. Based on this structural model, we aimed to chemically modify the residues, including both segments, to explore the possibility of increasing the affinity by simultaneously enhancing interactions with the two hot spots.

## 2. Materials and Methods

### 2.1. Buffers and Reagents

Buffers were prepared with reagent-grade chemicals using distilled, deionized water (Direct-Q^®^ 3 UV Water Purification System). Buffer compositions are specified in the text for each experiment.

### 2.2. Proteins and Peptides

pET14b—*E. coli* Exonuclease I was a gift from Charles Bell (Addgene plasmid # 104552; http://n2t.net/addgene:104552; RRID: Addgene_104552, accessed on 23 July 2019). ExoI was overexpressed using the BL21 (DE_3_) pLysS strain and purified using gravity flow HisPur Ni-NTA (Thermo Scientific, Waltham, MA, USA) affinity chromatography and HiTrap Heparin HP (Cytiva) affinity chromatography coupled to an AKTA Purifier FPLC chromatography system. Purified HisExoI was incubated with Thrombin (Sigma-Aldrich, St. Louis, MI, USA) before Heparin chromatography, as described in [36]. Escherichia coli RecO gene was synthesized and cloned into a pET28a+ vector (Proteogenix, Schiltigheim, France). A TEV cleavage site was cloned to the 5′ end of the RecO gene. Protein was overexpressed in *E. coli* Rosetta cells and purified using gravity flow Ni-NTA chromatography and a HiTrap Heparin HP column. N-terminal His-tag was cleaved using TEV protease (Sigma-Aldrich), before Heparin chromatography [37]. Proteins were extensively dialyzed against their reaction buffers prior to the experiment. The concentrations of ExoI and RecO were determined using NanoDrop 2000 Spectrophotometer in advance. ExoI and RecO extinction coefficients were ε_280_ = 7.483 × 10^4^ and 2.441 × 10^4^, respectively, as calculated using the Expasy Protparam web tool [38].

SSB-Ct peptide variants were synthesized on solid phase using manual synthesis on Wang resin (Sigma-Aldrich). Functionalization of Wang resin was carried out using DCC (Fluorochem, Glossop, UK)/HoBt (Fluorochem) coupling, adding DMAP (Fluorochem) as a catalyst. Three equivalents of Fmoc amino acid, three equivalents of 1-[Bis(dimethylamino)methylene]-1H-1,2,3-triazolo [4.5-b]pyridinium 3-oxid hexafluorophosphate (Iris Biotech) and six equivalents of N,N-diisopropylethylamine (Fluorochem) were used for each coupling cycle. Fmoc-protected amino acids were purchased from Iris Biotech and Fluorochem. Deprotection steps used 5% piperazine (Molar) and 2% DBU (Alfa Aesar) in DMF (VWR) for two 5 min periods. The N-terminus of the peptides was closed by either acetylation or CFU-GG-moiety (5,6-carboxyfluorescein, followed by two glycines), yielding F-SSB-Ct. Peptides were cleaved by shaking with trifluoroacetic acid:H2O:triisopropylsilane:dithiothreitol:DCM, 50:2.5:2.5:45 *v*/*v*/*m*/*v* (1.25 mL/25 mmol) for 1 h, followed by precipitating into cold diethyl ether.

The precipitate was collected through centrifugation/decantation before purification. All peptides were purified by reverse-phase HPLC; purity was >95%. Peptides were further characterized through analytical HPLC using a Phenomenex Aeris™ 3.6 µm Peptide XB-C18, 100 Å, 250 × 4.6 mm column with UV and electron spray ionization mass spectrometry detection for purity and identity, respectively (Supplementary Material, peptide analytical data). Pure peptides were lyophilized. Peptides for fluorescence anisotropy were stored in 100% dimethyl sulfoxide (Thermo Scientific) and freshly diluted into appropriate buffers. The peptide concentration was determined using HPLC-UV and Nanodrop in water.

### 2.3. Fluorescence Anisotropy Assay

Triplicate measurements were performed using a Clariostar Plus Microplate Reader in 384-well plates for all fluorescence anisotropy (FA) experiments. The settings were 25 °C, excitation at 485 nm, emission at 510 nm, settling time of 0.2 s, and 200 flashes per well.

Competition fluorescence anisotropy experiments were carried out as follows: protein (0.5 μM either ExoI or RecO) was incubated with 50 nM F-wtSSB peptide and 0–500 μM unlabeled wtSSB (or a variant) peptide for 30 min at room temperature. For direct fluorescence anisotropy measurements, 16 µM ExoI and 7.5 µM RecO were used in the first well. After serial dilution, either 50 nM of F-mSSB or F-sSSB was added to the wells and then incubated for 30 min. Buffers used included 20 mM Tris-HCl (pH 8.0), 100 mM NaCl, 1 mM MgCl_2_, 1 mM 2-mercaptoethanol, 10% (*v*/*v*) glycerol, for ExoI and 20 mM Tris-HCl (pH 8.0), 200 mM NaCl, 1 mM 2-mercaptoethanol, 10% (*v*/*v*) glycerol, and 0.01% Triton-X 100 for RecO [37].

Fluorescence anisotropy values were normalized and are shown as bound fractions (BF, %).

$$BF=\frac{r-r_{min}}{\lambda (r_{max}-r)+r-r_{min}},$$

I=2∗PG+S,   r=S−PG/I,

where r: anisotropy, I: total intensity, P: perpendicular intensity, S: parallel intensity, G: an instrument factor set to 1, BF: ligand fraction bound, and $\lambda =\lambda_{bound}/\lambda_{unbound}=1$.

Competitive fluorescence anisotropy was analyzed in Origin Pro 9.5, and IC_50_ values were determined using the Logistic Nonlinear fit function and are shown as plots for each mutated position for each protein in Figures S4 and S6. EC_50_ values from direct fluorescence anisotropy titrations were plotted in Origin 9.5. Values were determined using the Logistics Nonlinear fit function.

### 2.4. Isothermal Titration Calorimetry

Isothermal titration calorimetry (ITC) experiments were performed using a MicroCal VP-ITC titration microcalorimeter. ExoI was dialyzed extensively against the indicated buffer and cleared through centrifugation at 14,000 rpm for 15 min at 4 °C. RecO was subjected to a quick buffer exchange using an Amicon Ultra ultrafiltration device (Sigma). Protein concentrations were determined after the buffer exchange procedure. Wild-type SSB-Ct or modified SSB-Ct were titrated into ExoI or RecO in 20 mM Tris–HCl (pH 8.0), 100 mM NaCl, 1 mM MgCl_2_, 4% glycerol, 1 mM β-mercaptoethanol or 50 mM HEPES pH 7.5, 50 mM NaCl, 25% glycerol, and 1 mM TCEP, respectively. The protein and peptide concentrations used in the ITC experiments are shown in Table 1.

The raw data were integrated using NITPIC [39]. The binding parameters, association equilibrium constant (KA), binding enthalpy (ΔH), and binding entropy (ΔS) were obtained by fitting the titration curves to a model of A + B = C in SEDPHAT [40]. Stoichiometry was fixed to 1:1 and the incompetent fraction was fitted for the protein concentration.

### 2.5. Pull down Assay

ExoI and RecO expressing cells were grown and lysed, as mentioned above. A fraction of cell lysate was used to quantify the recombinant protein content. BL21 (DE)_3_ pLysS cells were grown on LB media until OD_600_ = 2.0 and lysed using sonication. Recombinant protein containing lysates were diluted with BL21 (DE)_3_ pLysS lysate, setting the final concentration of the recombinant protein to 1 µM. SSB peptides (wtSSB, E1-sSSB, R2-sSSB) were immobilized onto Streptavidin DynaBeads using an N-terminal biotin tag. Then, 1 µM immobilized peptides were incubated with cell lysate for 30 min. Beads were washed with trypsin digestion buffer (20 mM TRIS pH = 8.0, 100 mM NaCl, 2 mM CaCl_2_). Beads were processed with an on-pellet digestion protocol. NanoLC-MS/MS analysis was carried out on a Waters ACQUITY UPLC M-Class LC system (Waters, Milford, MA, USA) coupled with a Q Exactive^TM^ Plus Hybrid Quadrupole-Orbitrap^TM^ mass spectrometer (Thermo Fisher Scientific, Waltham, MA, USA). The instrument was operated in the data-dependent mode. The RAW files were searched against the latest Uniprot Escherichia coli reference proteome FASTA (4448 entries, https://www.uniprot.org/proteomes/UP000000625, accessed on 14 October 2022) with MaxQuant (2.2.0.0).

### 2.6. Modeling

For the structural characterization of the wild-type and the modified SSB-Ct peptide to the investigated enzymes, an extended conformational sampling method, replica-exchange solute tempering molecular dynamics (REST) [41,42], was applied, as implemented in the Desmond package [43]. The initial binding modes of the peptides to the ExoI enzyme were obtained by docking them into the protein structures (PDB ID 3C94 and 3Q8D) using the peptide docking protocol of the Schrödinger program suit [43]. For ExoI, the modified wtSSB-Ct was docked to match the initial crystal structure with the modified-Phe8 moiety oriented towards site A. For the initial complex of RecO, the distal part of wtSSB-Ct was fitted to the original ligand in the crystal structure (PDB ID 3Q8D), and the proximal part was minimized and relaxed by MD simulation. A geometry with both Phe residues in close contact with RecO was selected as a starting complex for the production run. This starting geometry was tested by redocking the wtSSB-Ct to the receptor using the same peptide docking protocol as applied for Exol. The MD calculation for the RecO-modified wtSSB-Ct complex was started from the same geometry. This structure was relaxed before the replica-exchange solute tempering simulation. The peptide-protein complexes were immersed in a rectangular simulation box filled with SPC [44] water. The total charge of the complex was neutralized with counter ions, and 0.15 M NaCl was added to the solution. The distance between the complex and the box walls was set to at least 10 Å. The proteins and peptides were parametrized using the recently developed OPLS4 [45] force field. This force field, together with the SPC water model, has already been successfully applied in several drug design-related studies (e.g., [46,47,48]). The whole system was relaxed according to the default protocol of the Desmond program. The REST molecular dynamics was run with six parallel simulations for 200 ns, with an cumulated time of 1.2 μs, in the temperature range of 308–510 K. One thousand complex structures from the second 100 ns of the 308 K replica were collected and used for the structural characterization of protein–peptide binding. To this end, the root-mean-squared fluctuation of the backbone atom coordinates of the ligands was calculated and the binding poses of the peptides were clustered using the affinity propagation method [49]. The central structures of these clusters are used as representatives in our figures.

## 3. Results

### 3.1. Molecular Dynamics Simulations of SSB-Ct–SIP Interactions

We built our models based on the X-ray analysis by Lu and Keck, who identified two hot spots on ExoI, both binding an octapeptide derived from the C-terminal segment of the SSB protein (wtSSB-Ct, Figure 1) [10]. Site A is located at the border of exonuclease and the SH3-like domains, while site B is in the SH3-like domain (Figure 2A). Due to crystal packing effects and the inherent flexibility of wtSSB-Ct, only four (DIPF at site A) and three (IPF at site B) C-terminal residues were resolved (Figure 2A), despite the presence of the octapeptidic wtSSB-Ct in the crystal. It was proven that wtSSB-Ct connects to site A with its DIPF motif in the bioactive binding mode [10]. The same phenomenon appeared for RecO; only the three C-terminal residues of wtSSB-Ct were resolved in the X-ray [11]. To test the possibility that the proximal segment of wtSSB-Ct (DFDD) interacts with site B, we carried out replica-exchange solute tempering (REST) molecular (MD) dynamics simulations (cumulated time of 1.2 μs, temperature range of 308–500 K).

**Figure 3:**
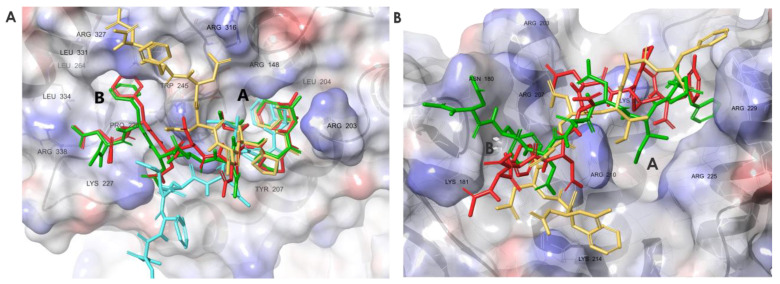
Representative structures of the MD simulations for the wtSSB-Ct–ExoI (**A**) and wtSSB-Ct–RecO (**B**) complexes, indicating the simultaneous binding of both proximal and distal segments at sites A and B. Proteins are shown in surface representation, wtSSB-Ct conformations appear as sticks. Structures were obtained with replica-exchange solute tempering (REST) molecular dynamics (cumulated time of 1.2 μs, temperature range of 308–500 K). The initial binding modes of wtSSB-Ct were obtained by docking wtSSB-Ct into ExoI and RecO structures (PDB ID 3C94 and 3Q8D) [10,11].

The representative structures of the MD simulation (Figure 3A) illustrate the ability of Phe2 of wtSSB-Ct to fit into site B in ExoI, while Phe8 is located in site A. Simultaneously, Asp1 forms ionic bonds with residue Arg338 at the basic ridge of ExoI (Figure S2). During our simulations, high energy complexes were found, where Asp1 was close to the Arg327 of ExoI [10]. However, this binding mode occurred in less than 1% of the simulation time, while the Asp1–Arg338 interaction dominated the conformational space, with 70%.

The modeling results reflected the non-uniform flexibility along the wtSSB-Ct chain in the bound state.

**Figure 4:**
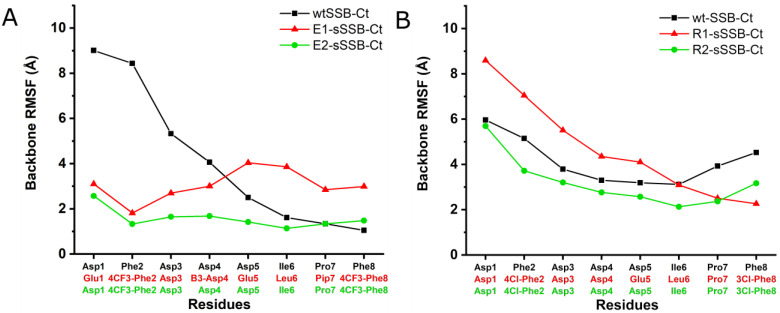
RMSF values of the backbone atom coordinates for wtSSB-Ct (black squares), E1-sSSB-Ct (red triangles), and E2-sSSB-Ct (green circles) in interaction with ExoI (**A**). RMSF values of the backbone atom coordinates displaying wtSSB-Ct (black squares), R1-sSSB-Ct (red triangles), and R2-sSSB-Ct (green circles) in interaction with RecO (**B**).

The root mean square fluctuation (RMSF) of the backbone atoms of Phe8 has a value of ~1 Å, showing a fixed position. Despite the potential transient interactions, the flexibility grows toward the proximal segment, and the RMSF reaches the value of 9 Å at Asp1 (Figure 4A). The modeling results for RecO also revealed a potential secondary hot spot (site B) in the proximity of the IPF-binding pocket (site A) (Figure 3B). Site B is enclosed by a loop (residues 178–183) and a helix (residues 200–212). The helix structure separates sites A and B. Positively-charged amino acids in the helix, especially Arg203, Lys206, and Arg210, interact with the negatively-charged central segment of wtSSB-Ct. Accordingly, we observed relatively low RMSF values for the central acidic segment, whereas modeling revealed a higher residual flexibility for the terminal IPF and DFD motifs (Figure 4B). We concluded from the simulations that the proximal DFD motif can have transient stabilizing contacts with the secondary hot spots on the SIPs studied.

### 3.2. Contributions of the Proximal DFDD and the Distal DIPF Segments to the Binding Affinity

Molecular dynamics simulations suggested that the proximal DFDD and the distal DIPF segments contribute to the binding affinity. We synthesized these tetrapeptides and performed competitive fluorescence anisotropy assays to test this hypothesis. The tracer sequence was the fluorescein-labeled wtSSB-Ct (F-SSB-Ct, Supplementary Material, Compound 53). First, we examined the ability of wtSSB-Ct to compete with the tracer. For ExoI and RecO, the inhibition constants (IC_50_) of wtSSB-Ct were 4.54 ± 0.35 µM and 4.66 ± 0.25 µM, respectively. For ExoI, the IC_50_ values were 311.8 ± 16.7 µM and 101.9 ± 6.61 µM for DFDD and DIPF peptides, respectively. This result supported that DFDD has a comparable contribution to binding affinity in interaction with ExoI. For RecO, DIPF displayed an IC_50_ of 44.84 ± 4.53 µM (Figure S3). We could not detect the direct inhibitory effect of DFDD for RecO (IC_50_ > 3 mM). However, the affinity of distal DIPF to RecO was a magnitude below the value obtained for the whole sequence of wtSSB-Ct (4.66 ± 0.25 µM), indicating a significant contribution to the binding affinity from the proximal DFDD segment. These findings strongly supported the simultaneous contribution of the DFDD and DIPF segments to the binding affinity, justifying the approach of introducing modifications along the whole sequence of wtSSB-Ct to improve affinity.

### 3.3. Synthesis and Screening of the Single Mutant wtSSB-Ct Library

**Figure 5:**
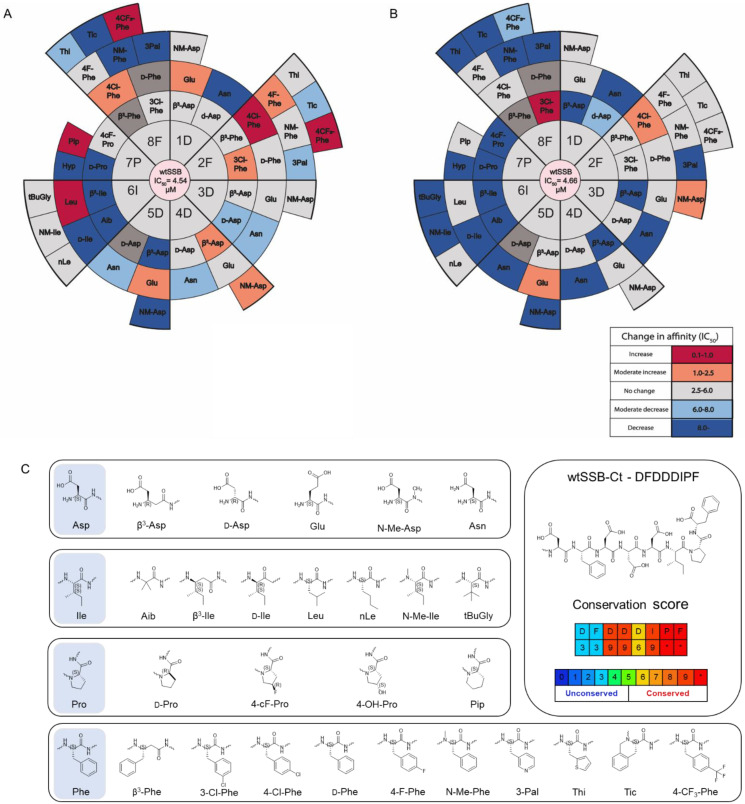
Screening and design of the mSSB-Ct library. Radial heat map showing competitive fluorescence anisotropy screening data of mSSBs on (**A**) ExoI and (**B**) RecO. IC_50_ values lower than 1 µM are highlighted in red. Moderate decreases in IC_50_ (1.0–2.5 µM) are shown in orange. Tolerated or non-significant changes are shown in light pink (IC_50_ = 2.5–6.0 µM). Moderate or significant increases in IC_50_ values (6.0–8.0 µM and >8.0 µM) are shown in light and dark blue, respectively. Modifications in dark grey were not evaluated, due to compound impurity. (**C**) wtSSB-Ct sequence, conservation score, and applied modifications for each occurring amino acid in the wtSSB-Ct sequence [21], * denotes the highest score.

In the next step, we determined if chemical modifications of the wtSSB-Ct improved the affinity and generated a single mutant wtSSB-Ct (mSSB-Ct) peptide library (Figure 5A,B). The importance of the side chain chemistry was investigated using non-natural amino acids having similar physicochemical characteristics and homologous replacements (Figure 5C). Potential effects of sidechain stereochemistry and backbone homology were also addressed using d-enantiomer and backbone homologation scans. N-methylated amino acids were included in all positions, to probe the H-bonding capabilities of the backbone amides. We synthesized 51 mSSB-Ct sequences and screened them in a fluorescence anisotropy-based competition assay on ExoI and RecO. The library members were evaluated against the IC_50_ values obtained for the wtSSB-Ct (Figure 5A,B and Figures S4–S6). The best hits were re-synthesized with a fluorescein label (Supplementary Material, Compound 54–62) and titrated directly, to validate the competition results (Table 2 and Figure S7).

### 3.4. Modifications in Both the Proximal DFDD and the Distal DIPF Segments Increase Affinity

For ExoI, Asp to Glu mutation at position 1 yielded a moderate increase in affinity, whereas no improvement was obtained at position 3. β^3^-Asp and NM-Asp substitutions yielded moderately higher affinities at position 4. A significantly higher affinity was achieved at position 2 with residues 4-Cl-Phe and 4-CF_3_-Phe. Other halogen-substituted phenylalanine modifications (3-Cl-Phe and 4-F-Phe) caused a moderate decrease in IC_50_. At position 5, the Asp to Glu mutation caused a moderate improvement. An isoleucine to leucine mutation at position 6 significantly increased the affinity to ExoI (IC_50_ = 0.92 µM). Of the various proline analogs, 4-F-proline was tolerated by ExoI, and 2-aminopipecolic acid increased the affinity (IC_50_ = 0.89 µM). Halogen-substituted phenylalanine modifications at position 8 were well tolerated (3Cl-Phe and 4F-Phe) or increased the affinity. A moderate increase was observed for 4-Cl-Phe, while 4-CF_3_-Phe in position 8 yielded an IC_50_ of 0.39 µM.

For RecO, positions 1 and 4 did not show any improvement. On the contrary, a moderate increase in affinity was obtained with 4-Cl-Phe and NM-Asp in positions 2 and 3, respectively. The Asp to Glu mutation was moderately advantageous at position 5. Leucine and norleucine were tolerated by RecO in position 6, with IC_50_ values of 3.22 µM and 2.55 µM, respectively. Modifications at position 7 did not result in improved affinity. Except for Pip, all replacements were detrimental to the binding. In stark contrast, Phe8 to 3-chlorophenyl alanine significantly decreased the IC_50_ value to 0.36 µM, whereas 4-F-Phe and 4-Cl-Phe were tolerated.

These results support our hypothesis that appropriate chemical modifications in the side chain of Phe2 can increase the affinity to the targets. The behavior of the two proteins tested was not uniform in their interaction with Phe2, which suggests an explanation for the lower conservation score for Phe2 [21]. Chemical mutation data on the essential DIPF segment revealed that the binding sites have a certain level of residual flexibility and can adapt to the substituted residues, so that the affinity increases.

### 3.5. Combined Modifications Yielded High-Affinity Ligands

**Figure 6:**
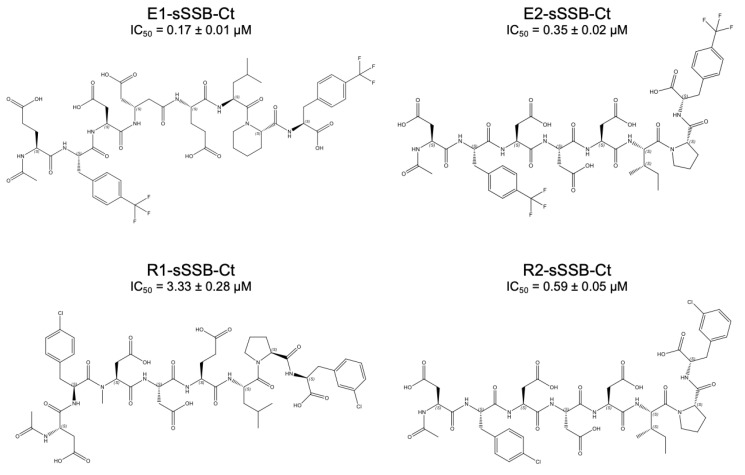
Combined modifications of wtSSB-Ct. E1-sSSB-Ct and E2-sSSB-Ct were tested on Exol. R1-sSSB-Ct and R2-sSSB-Ct were tested on RecO. IC_50_ values show the ability of the sequences to compete effectively with F-wtSSB-Ct in a competitive fluorescence anisotropy assay.

In the next step, we tested if the combination of the favorable target-specific single mutations could increase the affinity any further. For ExoI, we synthesized E1-sSSB-Ct (Glu–4-CF_3_-Phe–Asp–β^3^-Asp–Glu–Leu–Pip–4-CF_3_-Phe) and E2-sSSB-Ct (Asp–4-CF_3_-Phe–Asp–Asp–Asp–Ile–Pro–4-CF_3_-Phe, Figure 6). These sequences were probed against ExoI, and the binding characteristics were measured using competitive fluorescence anisotropy and isothermal titration calorimetry.

**Figure 7:**
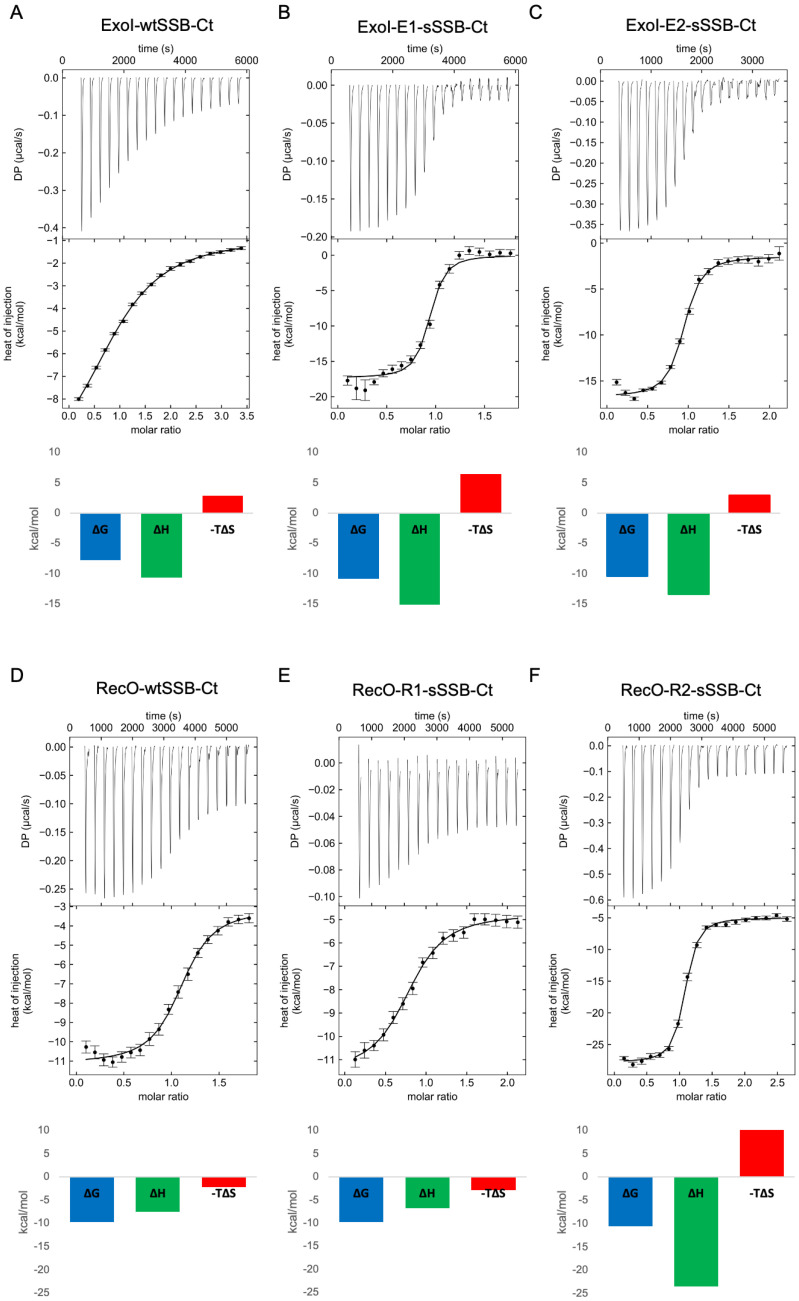
Thermodynamic profiles of binding to ExoI and RecO for the wild-type and modified SSB-Ct sequences. ITC data for ExoI titrated with wtSSB-Ct (**A**), E1-sSSB-Ct (**B**), and E2-sSSB-Ct (**C**). ITC data for RecO titrated with wtSSB-Ct (**D**), R1-sSSB-Ct (**E**), and R2-sSSB-Ct (**F**). The reconstructed thermograms displayed were obtained after global peak-shape analysis and singular value decomposition regularization implemented in the NITPIC program.

For ExoI, the combined modifications incorporated into E1-sSSB-Ct and E2-sSSB-Ct yielded a further improvement; the IC_50_ value decreased to 0.17 µM and 0.35 µM, respectively. However, the effects of the mutations were not fully additive. Within the framework of a two-hot-spot binding model, the enthalpically-stabilized contacts inevitably decreased the residual flexibility of the protein–ligand complex. To test the enthalpy–entropy compensation, we carried out isothermal titration calorimetric measurements and determined the stoichiometry and the thermodynamic parameters of the interactions (Figure 7 and Table 3).

We found a stoichiometry of 1:1, supporting that the secondary binding sites did not bind an additional ligand under the conditions applied. In accordance with the literature results, the wtSSB-Ct sequence without the fluorescent tag had a higher affinity to RecO (K_D_ = 0.14 µM) than to ExoI (K_D_ = 3.07 µM) [50]. E1-sSSB-Ct and E2-sSSB-Ct displayed low nanomolar dissociation constants (K_D_ = 19 nM and 40 nM, respectively) with a 1:1 stoichiometry. The comparison of E1-sSSB-Ct with E2-sSSB-Ct revealed that the affinity gain could be attributed to the Phe –> 4-CF_3_-Phe replacements at positions 2 and 8, supporting the two-hot-spot model. However, the additional modifications in E1-sSSB-Ct could further improve the binding affinity. The absolute value of the binding enthalpy exhibited a marked increase for both peptides. Peptide E1-sSSB-Ct showed an increased enthalpic drive for binding relative to E2-sSSB-Ct, possibly due to the more favorable contacts with the protein. A decreased binding entropy accompanied this because of the flexibility lost in the process. The enthalpy–entropy compensation effect was even more pronounced in comparison with the native wtSSB-Ct. This finding strongly supports that both proximal and distal segments contribute to the affinity, by contacting separate hot spots over the protein surface. To gain further support for this, we carried out ^19^F-NMR measurements with four peptides containing 4-CF_3_-Phe residues: single mutant derivatives (4-CF_3_-Phe2 and 4-CF_3_-Phe8) and sequences with combined modifications (E1-sSSB-Ct and E2-sSSB-Ct). Fluor signals corresponding to the proximal and distal segments disappeared upon adding Exol in a 1:1 ratio (Figure S8). This finding confirmed the stabilization of the peptide–Exol interaction at both terminals of the peptides.

For RecO, R1-sSSB-Ct (Asp–4Cl-Phe–NM-Asp–Asp–Glu–Leu–Pro–3Cl-Phe) was synthesized first (Figure 6). R1-sSSB-Ct contains all favorable modifications, but the effects turned out to be non-additive. R1-sSSB-Ct failed to compete with wtSSB-Ct in the expected manner (IC_50_ = 3.33 µM). ITC titration of R1-sSSB-Ct showed no significant changes in the thermodynamic profile of binding (Figure 7E), resulting in a ΔG value similar to the wtSSB-Ct (Figure 7D). Therefore, we synthesized R2-sSSB-Ct (Asp–4Cl-Phe–Asp–Asp–Asp–Ile–Pro–3Cl-Phe), which only contains modifications at the Phe residues (Figure 6). This peptide competed with wtSSB-Ct, having an IC_50_ value of 0.59 µM. The ITC results confirmed the affinity increase (K_D_ = 35 nM), due to a marked increase in the enthalpic stabilization, which was damped by an enthalpy–entropy compensation effect (Figure 7F).

To test the affinity improvement for E1-sSSB-Ct and R2-sSSB-Ct relative to wtSSB-Ct in a complex environment, we carried out a pull-down assay using bacterial cell lysates. ExoI and RecO overexpressing BL21 (DE)3 pLysS cells were diluted with non-overexpressing cells, to obtain a 1 µM final concentration for the recombinant proteins. The lysates were incubated with wtSSB-Ct, E1-sSSB-Ct, and R2-sSSB-Ct immobilized on streptavidin beads. The washed bead was digested, and tryptic peptides were detected using HPLC-MS. We found a two-fold enrichment of ExoI on E1-sSSB and a ten-fold enrichment of RecO on R1-sSSB as compared to wtSSB-Ct (Figure S10).

### 3.6. Molecular Dynamics Simulations Provided Insight into the Binding Modes of E1-sSSB-Ct and R2-sSSB-Ct

**Figure 8:**
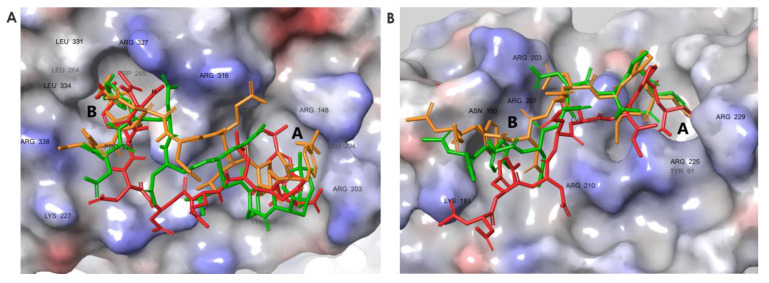
Representative structures for the complexes ExoI–E1-sSSB-Ct (**A**) and RecO–R2-sSSB-Ct (**B**). Binding modes were clustered using the affinity propagation method based on the backbone RMSD values of the ligands, and the central structures of the clusters are displayed.

To test the changes in the residual flexibility of E1-sSSB-Ct and R2-sSSB-Ct, we performed replica-exchange solute tempering simulations. In both cases, the simulations yielded reduced residual flexibility in the bound state, according to the experimental findings (Figure 8). E1-sSSB-Ct was attached to site B through 4CF_3_-Phe2 and formed a stable contact with site A through 4CF_3_-Phe8. This improved stability led to root mean square fluctuation (RMSF) values of 2 and 3 Å toward the ends of the segments. Interestingly, E2-sSSB-Ct (which only has the 4CF_3_-Phe modifications) showed a superior backbone RMSF stabilization compared with the wild-type and E1-sSSB-Ct peptides. This implies that some modifications at positions 5–7 may not be beneficial in the combination applied. R2-sSSB-Ct was connected to sites A and B through 4Cl-Phe2 and 3Cl-Phe8, respectively. The RMSF value of 4 Å at the proximal end decreased to 2.5 Å toward the distal end of the peptide. 

For RecO, the central segment of R2-sSSB-Ct (residues Asp4-Ile6) was the least flexible part compared to the proximal and distal segments. The RMSF values for R2-sSSB-Ct displayed an overall downward shift relative to wtSSB-Ct (Figure 4B).

The ExoI–E1-sSSB-Ct and RecO–R2-sSSB-Ct complexes revealed contacts with site B. The role of site B has been confirmed for ExoI in the literature [10]. For RecO, we tested the stabilization effects of the site B contacts through a computational Ala-scan. Inspection of the RecO–R2-sSSB-Ct structure showed that site B was lined by residues Thr163, Ile178, Asn180, Thr182, Phe183, Thr184, Gln187, Arg203, and Arg207. For the complexes of R2-sSSB-Ct and alanine-mutated RecO, we repeated the REST molecular dynamics calculation described above. The increased RMSF values calculated for the backbone of the ligand indicated a destabilization due to the mutation of the corresponding side chain (Figure S9A). Mutation of the RecO residues Thr182, Thr163, Thr 184, and Phe183 caused destabilization at the proximal segment of R2-sSSB-Ct. Residues Gln187, Arg203, and Arg207 had stabilizing effects along the whole sequence of the ligand. Despite the proximity of the side chains to the ligand, Ile178 and Asn180 do not seem to play important roles. The Ala-scan calculations repeated for the RecO-wtSSB-Ct complex (Figure S9B) showed that the mutations of the above mentioned residues exerted a considerably smaller influence on the wtSSB-Ct than those on the R2-sSSB-Ct binding. It is worth noting that the positively-charged Arg203 and Arg207 residues were also involved in the binding of the wild-type peptide, although their Ala-mutation caused smaller changes in the backbone RMSF of the peptide than in the case of the RecO-R2-sSSB-Ct complex. These findings indicate that the interaction of the ligand’s N-terminal with site B had a detectable positive influence on the binding affinity.

With the above results in hand, we attempted to explain the failure of R1-sSSB-Ct to form a high-affinity interaction with RecO. Replica exchange simulations for R1-sSSB-Ct showed a weakened RecO-R203–R1-sSSB-Ct-Glu5 salt bridge as compared with that formed by R2-sSSB-Ct-Asp5. This interaction is needed to direct the proximal segment toward site B. The unfavored orientation prevents residue 4Cl-Phe2 in R1-sSSB-Ct from occupying the hot spot. This explanation was also supported by the backbone RMSF calculation of the R1-sSSB-Ct peptide bound to the RecO. Although its distal residues show increased stability compared to the wild-type peptide, the proximal end became more flexible (see Figure 4B).

## 4. Discussion

Protei–protein interaction (PPI) inhibitors are becoming increasingly prevalent, and the number of compounds reaching clinical trials is increasing yearly [51]. Many of the available PPI inhibitors inhibit ordered interfaces [51,52,53]. However, interfaces involving intrinsically disordered proteins (IDP) are less understood. Some IDPs may undergo an induced conformational change upon binding and form strong interactions [54]. Others form weak, transient interactions with their partners, creating a fuzzy complex, where multiple conformers of the intrinsically disordered region exist simultaneously, bound to its target. IDPs are involved in many diseases and are pharmacologically relevant targets; thus, understanding their structural biology and how IDP interfaces work is crucial for targeting such PPI interfaces [55,56,57].

For SSB, the C-terminal IDP domain is responsible for the adaptive interactions recruiting several DNA-manipulating enzymes. While X-ray studies revealed the binding geometry for the IPF motif at the tip, the strange target-induced structural behavior and binding geometries of the flexible proximal segment justified our investigations. Thorough conformational sampling for the wtSSB-Ct sequence in the presence of the targets revealed that the proximal segment makes hydrophobic and electrostatic contacts with ExoI and RecO. Contrary to previous deletion studies, we attempted to chemically modify the residues with unnatural amino acids, to gain affinity. Non-natural amino acids are of great importance in medicinal chemistry. They are valuable tools for uncovering structure–activity relationships and designing peptidomimetic drugs [58]. In this study, neither the d- nor the backbone homologation (with one exception) scans led to improved affinity; the side chain orientations and their distance are crucial for the interaction. This observation strongly suggests that wtSSB-Ct binding requires an overall geometry that is disrupted by the variations of stereochemical configurations and the backbone length. However, modifications at both terminals resulted in improved affinities, further supporting the limited flexibility and the role of the proximal segment in binding. We can conclude that it is not only the X-ray visible IPF motif that can positively contribute to the interaction, but the proximal DFDD segment can also establish stabilizing contacts, especially when appropriate chemical modifications are introduced into the side chains. These observations align with the in silico model with the two hot spots. We found different levels of flexibility for the wtSSB-Ct–SIP complexes at the termini of the peptidic ligand, which suggests that SIPs cannot be handled as a homogenous cluster concerning their SSB interfaces. This phenomenon can explain why the conservation scores are lower at the proximal segment of wtSSB-Ct when all SIPs are included in the bioinformatic analysis.

Notably, the halogen substitutions in the Phe residues were among the most preferred. Halogenation is a valuable tool in medicinal chemistry, due to the potential increase in affinity and favorable modification of pharmacokinetic parameters. The introduction of halogen atoms can introduce new interactions with the protein target; halogens form hydrophobic contacts, but the more polarizable halogens (every halogen except for fluorine) can form halogen bonds. Hydrogen bonding is often observed between halogen-containing ligands and proteins [59,60].

## 5. Conclusions

The sequences combining the preferred chemical modifications displayed low nanomolar affinities. However, the thermodynamic characterization of the interactions revealed a marked enthalpy–entropy compensation. This effect erodes the additivity of the enthalpic stabilizing contacts at the anchor points, by decreasing the residual disorder of the complexes. Despite the disadvantageous decrease in the residual entropy, the affinity-increasing approach based on the two-hot-spot model was successful. These results may pave the way for effective SSB mimetic antibiotics design. Mapping multiple *E. coli* SIPs or even screening the SSB-interactome of other bacteria might enable the design of a multi-acting SSB mimetic peptide. To permit the further development of these inhibitors as novel antibiotics, the problem of the bacterial cell penetration of the acidic ligands must be addressed. Conjugation to antimicrobial or cell-penetrating peptides, prodrug formation to mask negative charges, or incorporation into liposomes might be possible solutions. This challenge was beyond the scope of the present work.

## Figures and Tables

**Table 1:** Protein and peptide concentrations used in the ITC experiments.

Protein	ExoI			RecO		
Peptide	wtSSB-Ct	E1-sSSB-Ct	E2-sSSB-Ct	wtSSB-Ct	R1-sSSB-Ct	R2-sSSB-Ct
cell (µM)	7.5	3	5	4	2	2
syringe (µM)	120	25	50	50	20	50

**Table 2:** Results of direct fluorescence anisotropy titrations with ExoI and RecO. IC50 values are shown for comparison.

mSSB	IC50 on ExoI (µM)	EC50 on ExoI (µM)	IC50 on RecO (µM)	EC50 on RecO (µM)
F-wtSSB	4.54 ± 0.34	0.35 ± 0.04	4.66± 0.24	0.37 ± 0.03
F-4Cl-Phe2	0.56 ± 0.03	0.15 ± 0.02	0.41 ± 0.27	0.26 ± 0.01
F-4F-Phe2	1.93 ± 0.81	0.36 ± 0.03	0.52 ± 0.28	0.23 ± 0.02
F-4CF3-Phe2	0.33 ± 0.02	0.04 ± 0.002	0.56 ± 0.42	0.18 ± 0.03
F-NM-Asp3	3.88 ± 0.13	0.86 ± 0.11	1.88 ± 0.09	0.27 ± 0.04
F-Glu5	1.49 ± 0.10	0.11 ± 0.01	1.41 ± 0.55	0.20 ± 0.02
F-Leu6	0.92 ± 0.06	0.20 ± 0.03	3.23 ± 0.16	0.31 ± 0.03
F-nLe6	2.68 ± 0.54	0.20 ± 0.01	9.36 ± 0.08	0.20 ± 0.01
F-Pip7	0.89 ± 0.14	0.09 ± 0.01	3.31 ± 0.44	0.25 ± 0.03
F-3Cl-Phe8	3.40 ± 2.41	0.25 ± 0.02	0.36 ± 0.04	0.22 ± 0.03
F-4CF3-Phe8	0.39 ± 0.07	0.09 ± 0.01	5.97 ± 2.43	0.26 ± 0.02

**Table 3:** Thermodynamic parameters obtained from the ITC binding data for the interactions of wtSSB-Ct variants with ExoI and RecO. Confidence intervals are shown for KD and ΔH values.

	ExoI			RecO		
	wtSSB-Ct	E1-sSSB-Ct	E2-sSSB-Ct	wtSSB-Ct	R1-sSSB-Ct	R2-sSSB-Ct
N	0.9593	1.0000	0.8846	1.0032	0.9353	0.9999
K_D_ (µM)	3.07 (2.62–3.64)	0.02 (0.01–0.03)	0.04 (0.02–0.07)	0.14 (0.08–0.24)	0.13 (0.08–0.22)	0.04 (0.02–0.05)
ΔG (kcal/mol)	−7.78	−10.89	−10.43	−9.77	−9.71	−10.54
ΔH (kcal/mol)	−10.67 (−11.64–−9.97)	−17.35 (−18.34–−16.43)	−13.45 (−14.46–−12.49)	−7.54 (−8.28–−6.90)	−6.79 (−7.84–−6.05)	−23.48 (−24.34–−22.73)
−TΔS (kcal/mol)	2.90	6.46	3.02	−2.28	−2.91	12.32

## Data Availability

The data published in this paper are available using the following link: http://doi.org/10.5281/zenodo.7711152 (accessed on 10 March 2023).

## Supplementary Materials

The following supporting information can be downloaded at https://www.mdpi.com/article/10.3390/pharmaceutics15041032/s1, Figures S1–S10: Analytical data for synthesized peptides; Tables S1–S3: Input files for the ExoI-bound wtSSB-Ct, E1-SSB-Ct, and E2.SSB-Ct and the corresponding trajectory files.
