# Supplementary material for: Structural Adaptation of the Single-Stranded DNA-Binding Protein C-Terminal to DNA Metabolizing Partners Guides Inhibitor Design
Source: Pharmaceutics. 2023 Mar 23;15(4):1032. doi: 10.3390/pharmaceutics15041032 (PMC10143822; doi:10.3390/pharmaceutics15041032)

Supplementary Materials: Structural adaptation of the single-stranded DNA-binding protein C-terminal to DNA metabolizing partners guides inhibitor design

Attila Tököli, Brigitta Bodnár, Ferenc Bogár,‡ Gábor Paragi, Anasztázia Hetényi, Éva Bartus, Edit Wéber† Zsófia Hegedüs, Zoltán Szabó, Gábor Kecskeméti, Gerda Szakonyi, and Tamás A. Martinek

Supplementary information:

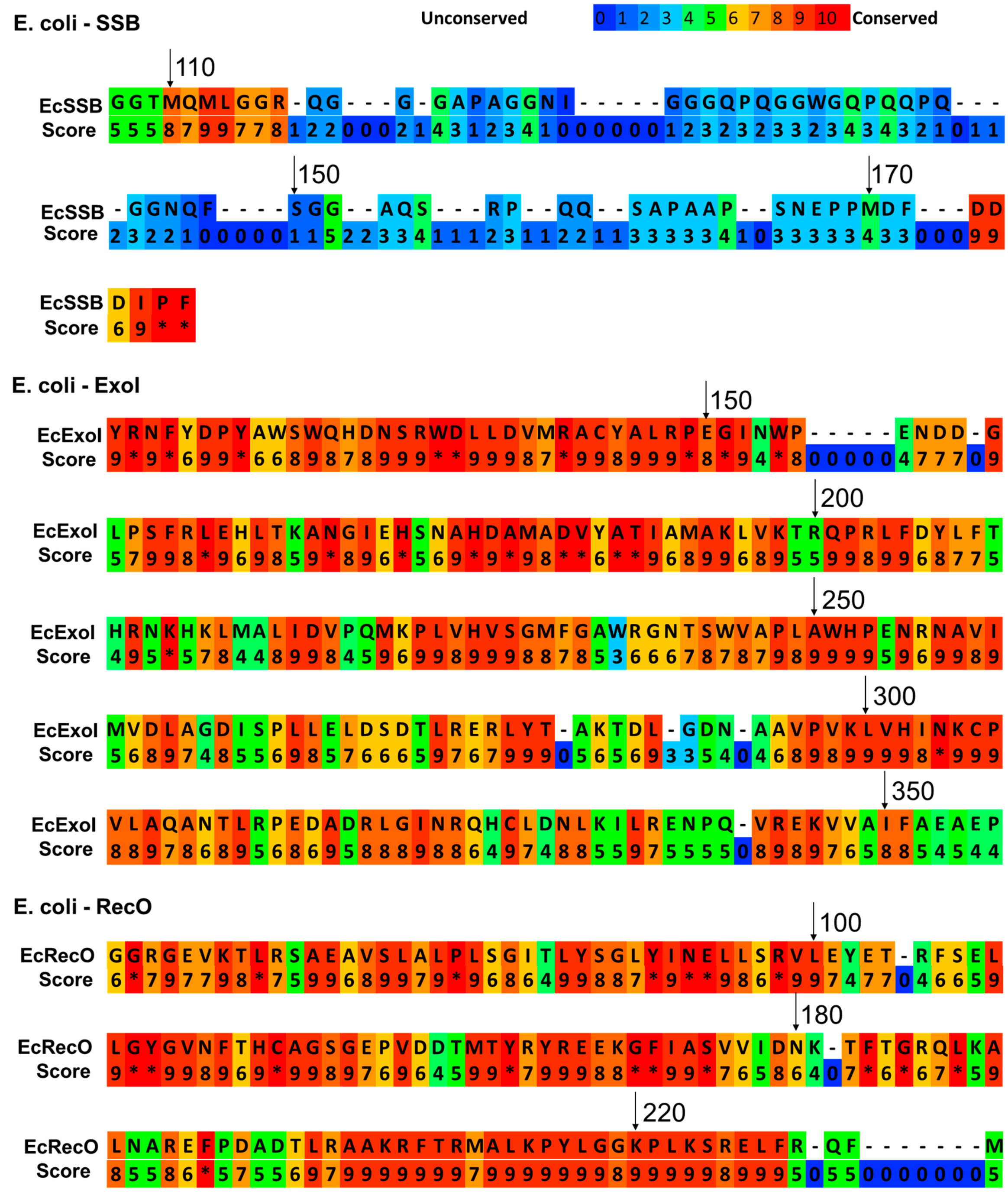

Figure S1. Sequence conservation across 250 eubacterial species shown for (A) SSB C-terminal tail, (B) ExoI, and (C) RecO focusing on segments important for SSB-SIP interactions. NCBI BLAST version 2.12.0. and Uniprot database (accession date: 28.11.2022.) was used for the search filtered for proteobacteria (taxid: 1224). The best 250 non-redundant sequences were further processed. The following parameters were used in PRALINE algorithm.[1] Exchange weights matrix: BLOSUM62, Associated gap penalties:12, Progressive alignment strategy: PSI-BLAST pre-profile processing (Homology-extended alignment). Structural features: DSSP-defined secondary structure search (YES), Secondary structure prediction (PSIPRED), Transmembrane structure prediction (NO). Output customization: Tree representation final alignment (NO), Customize alignment colours (NO), File format final alignment (FASTA).

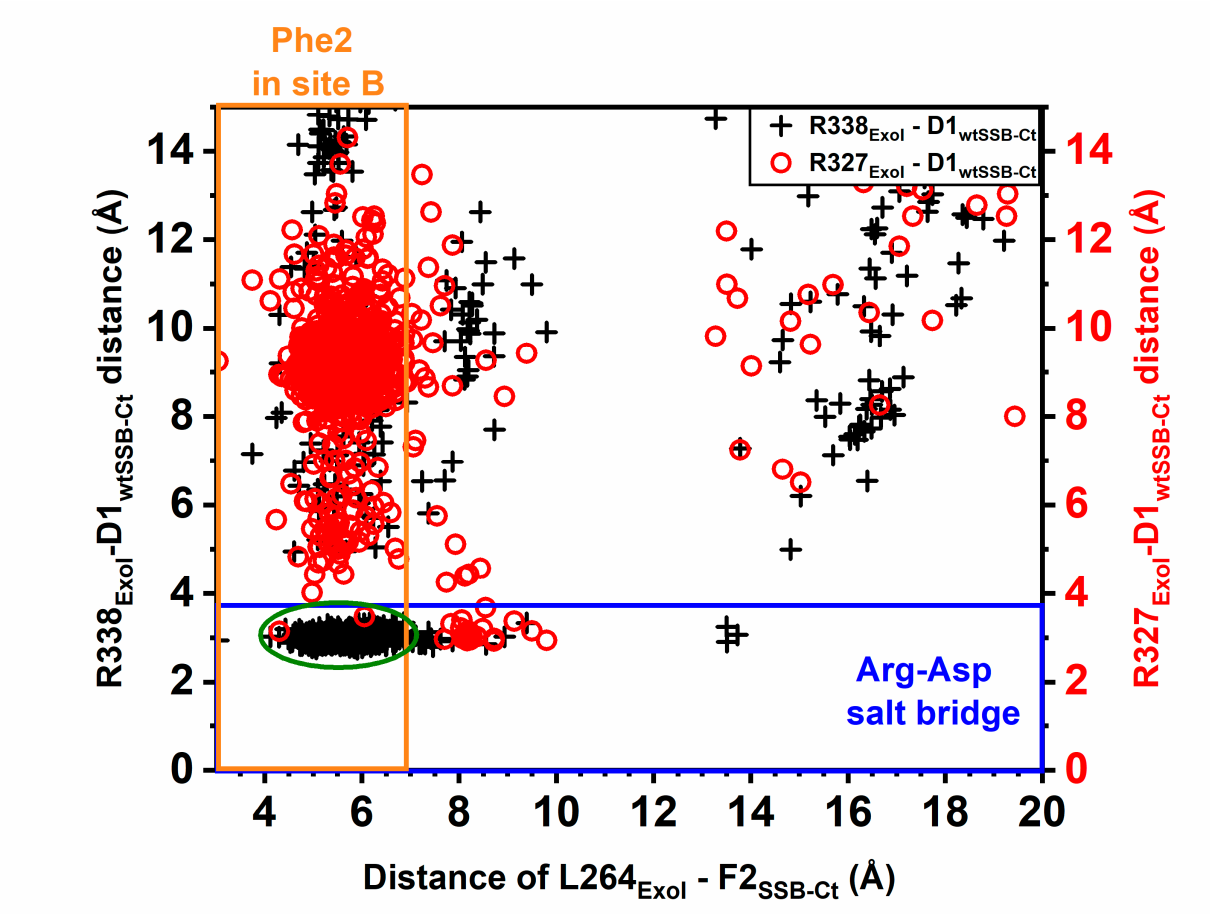

Figure S2. Distribution of the R338$_{ExoI}$–D1$_{wtSSB-Ct}$ distance against the L264$_{ExoI}$–F2$_{wtSSB-Ct}$ distance (black cross). Distribution of the R327$_{ExoI}$–D1$_{wtSSB-Ct}$ distance against L264$_{ExoI}$–F2$_{wtSSB-Ct}$ distance (red circle). Each symbol represents a trajectory point from the lowest temperature replica of the REST simulation. The population appearing within the green ellipse indicates conformations with Phe2 bound to site B and the concomitant stabilizing salt bridge formed by Asp1 of wtSSB-Ct. Low distance for R338$_{ExoI}$–D1$_{wtSSB-Ct}$ is predominant over that for R327$_{ExoI}$–D1$_{wtSSB-Ct}$.

[1] Bawono and Heringa, "PRALINE."

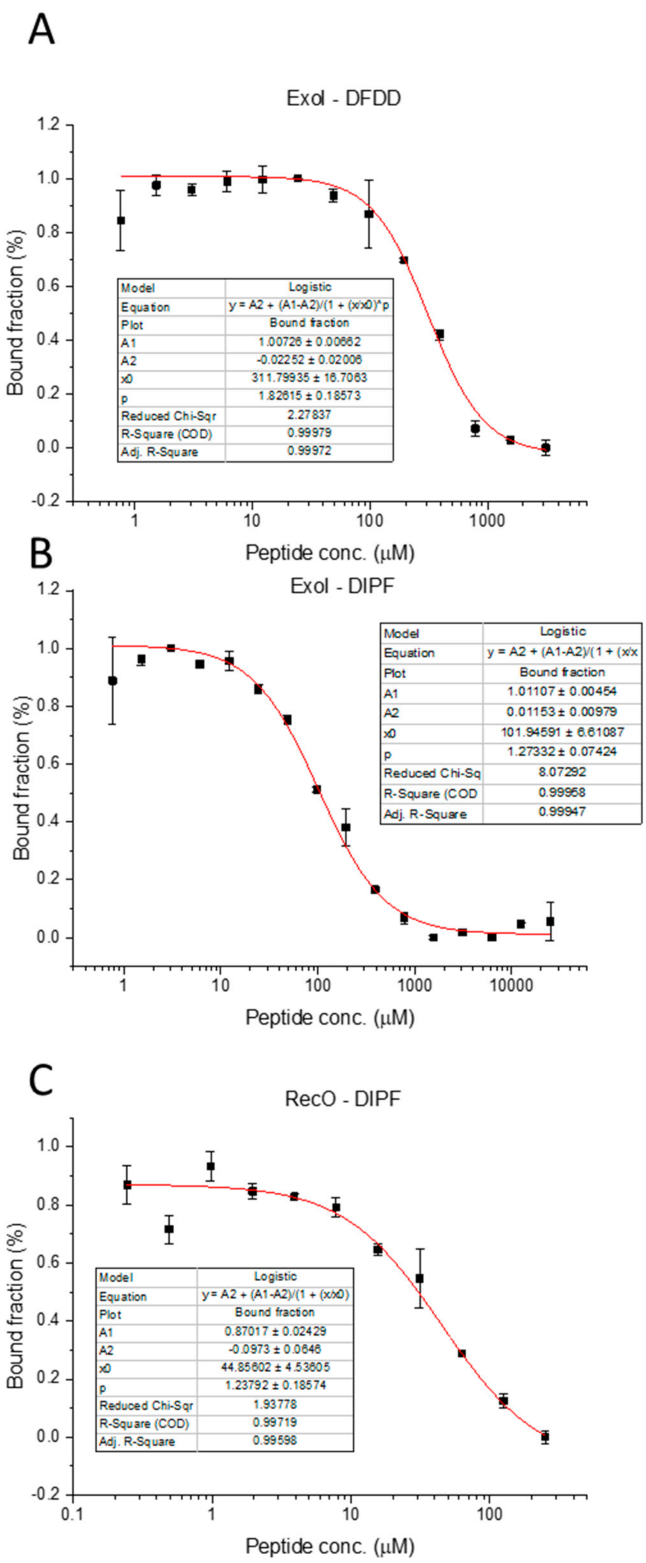

Figure S3. Competitive fluorescence anisotropy titration curves for the ExoI–DFDD (A), ExoI–DIPF (B) and RecO–DIPF (C) interactions.

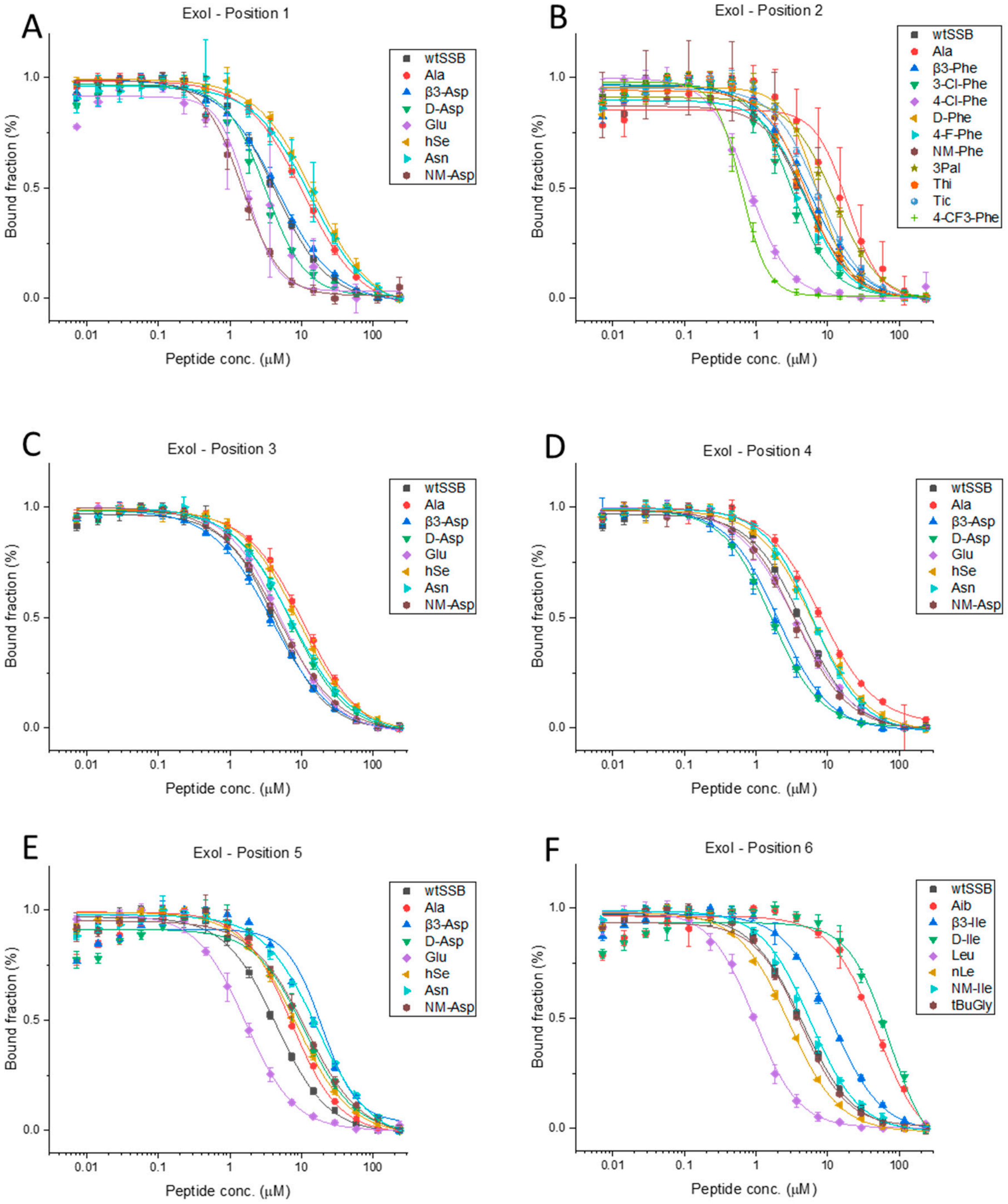

Figure S4 (a). ExoI-mSSB competitive fluorescence anisotropy titration curves for each position (A-F).

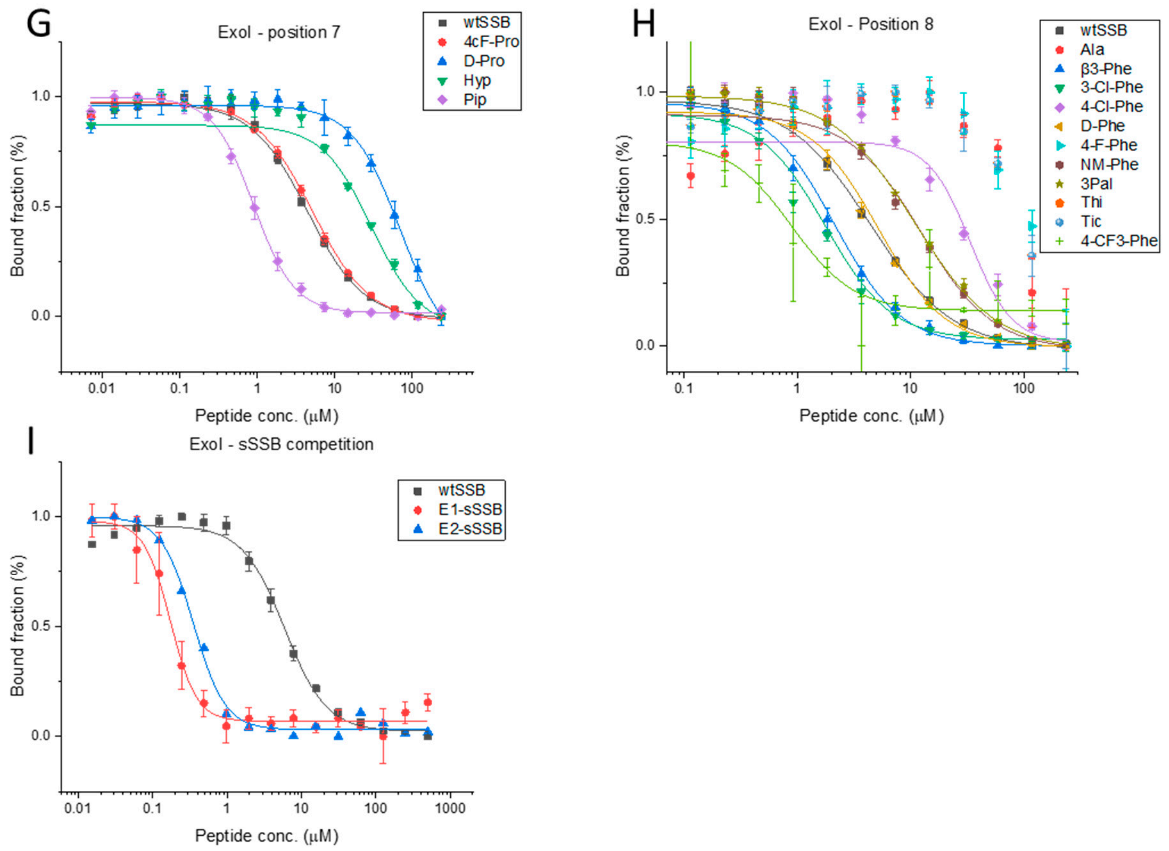

Figure S4 (b). ExoI-mSSB competitive fluorescence anisotropy titration curves for each position (G-H). ExoI-sSSB competition curves (I).

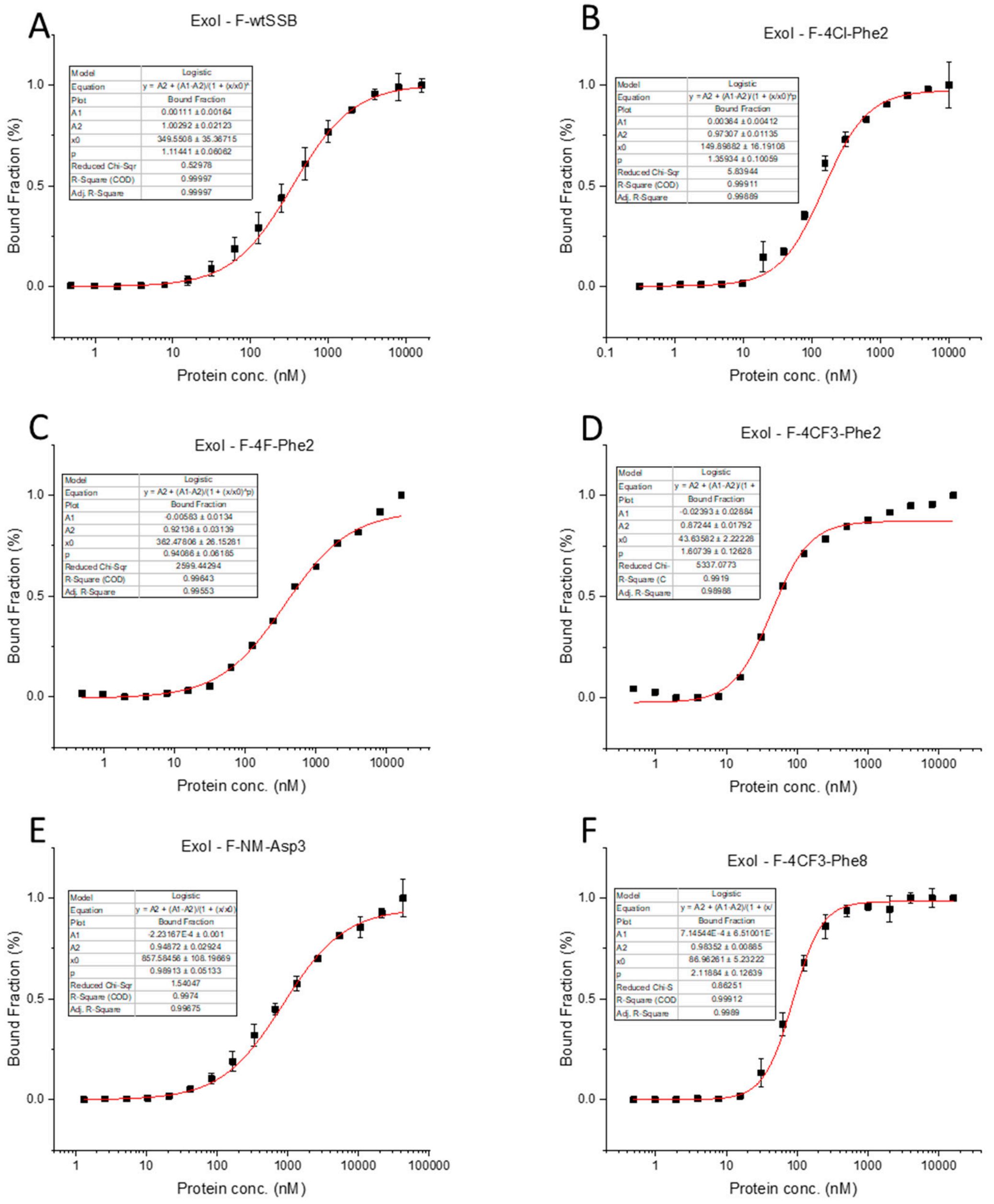

Figure S5 (a). Direct titration of F-mSSBs to ExoI. Validation of competitive fluorescence anisotropy hits.

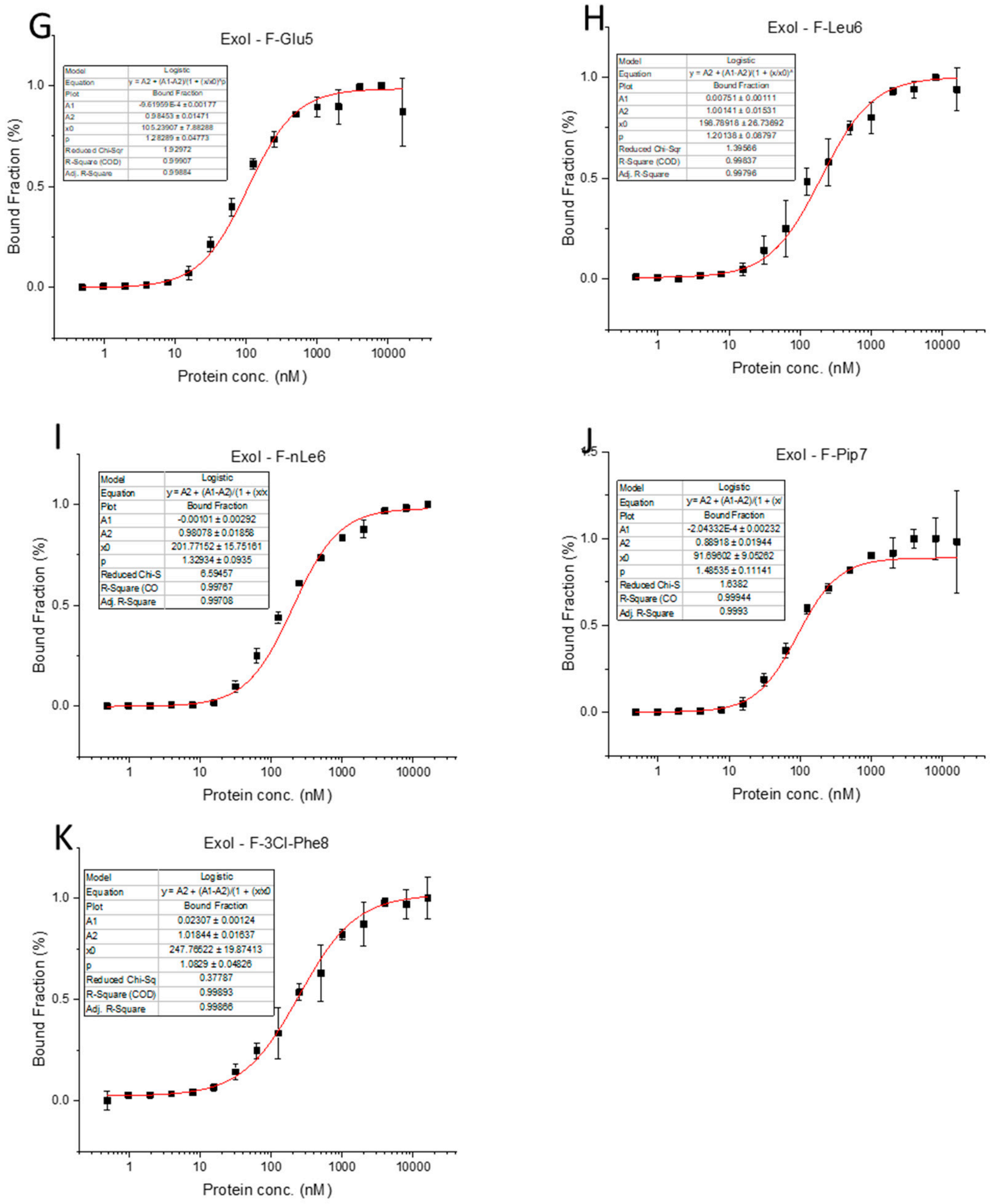

Figure S5 (b). Direct titration of F-mSSBs to ExoI. Validation of competitive fluorescence anisotropy hits.

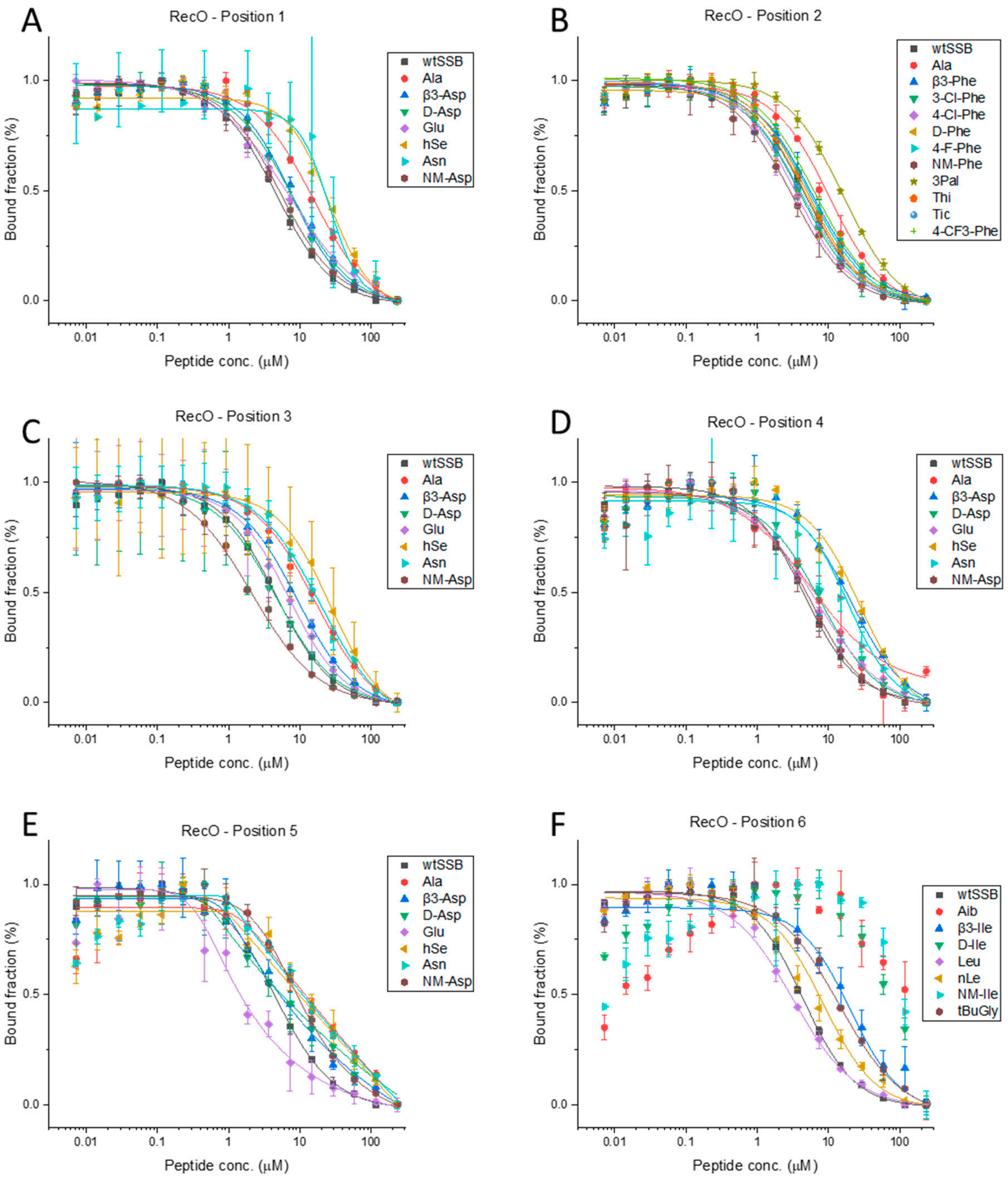

Figure S6. a. RecO-mSSB competitive fluorescence anisotropy titration curves for each position (A-F).

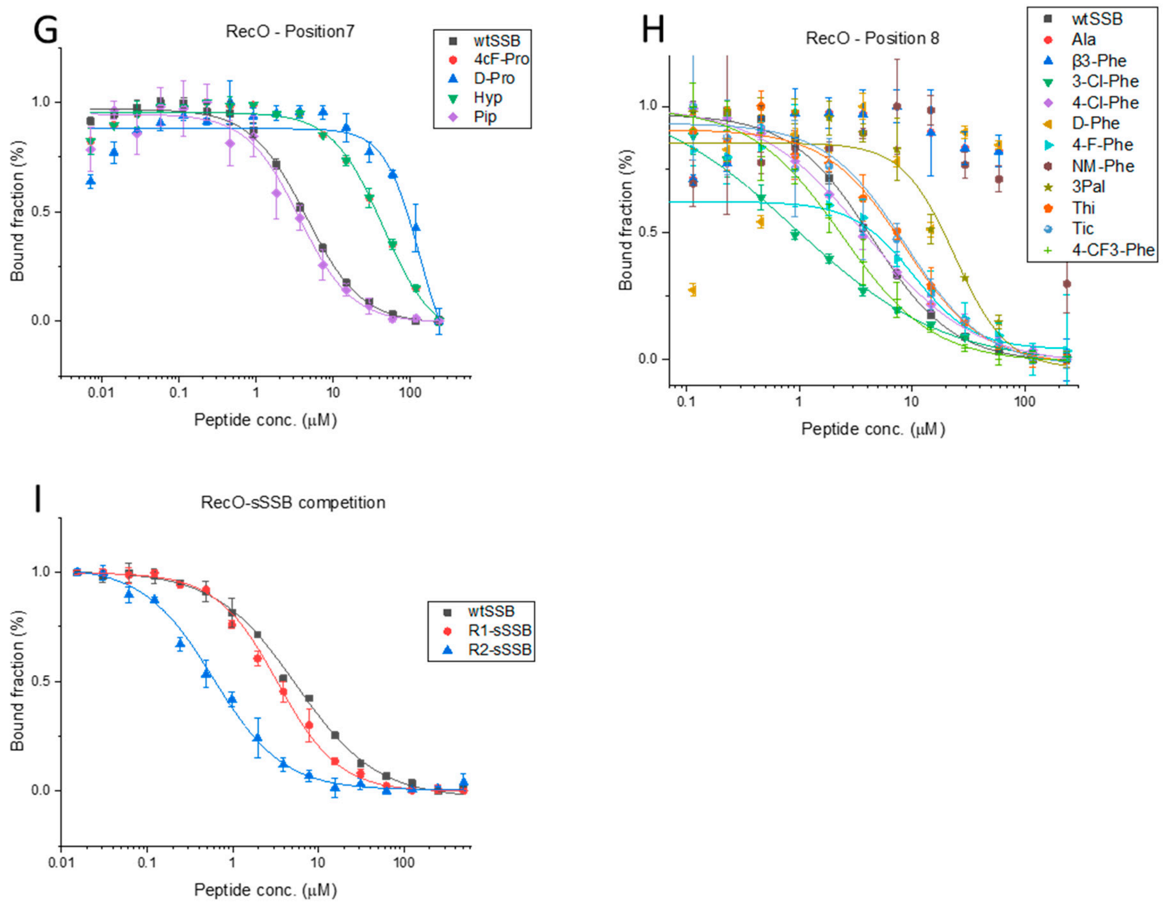

Figure S6. b. RecO-mSSB competitive fluorescence anisotropy titration curves for each position (G-H). RecO-sSSB competition curves (I).

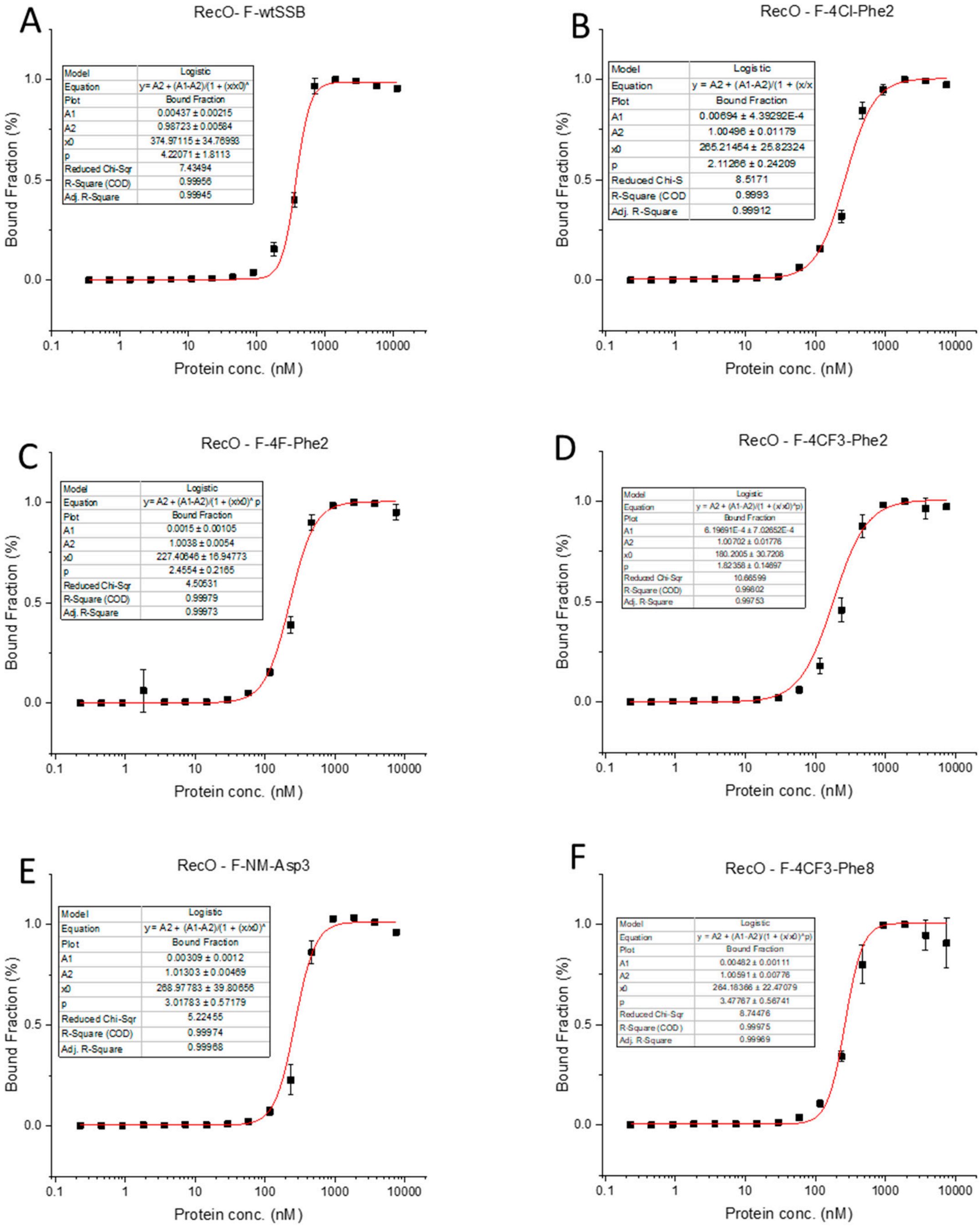

Figure S7. a. Direct titration of F-mSSBs to RecO. Validation of competitive fluorescence anisotropy hits.

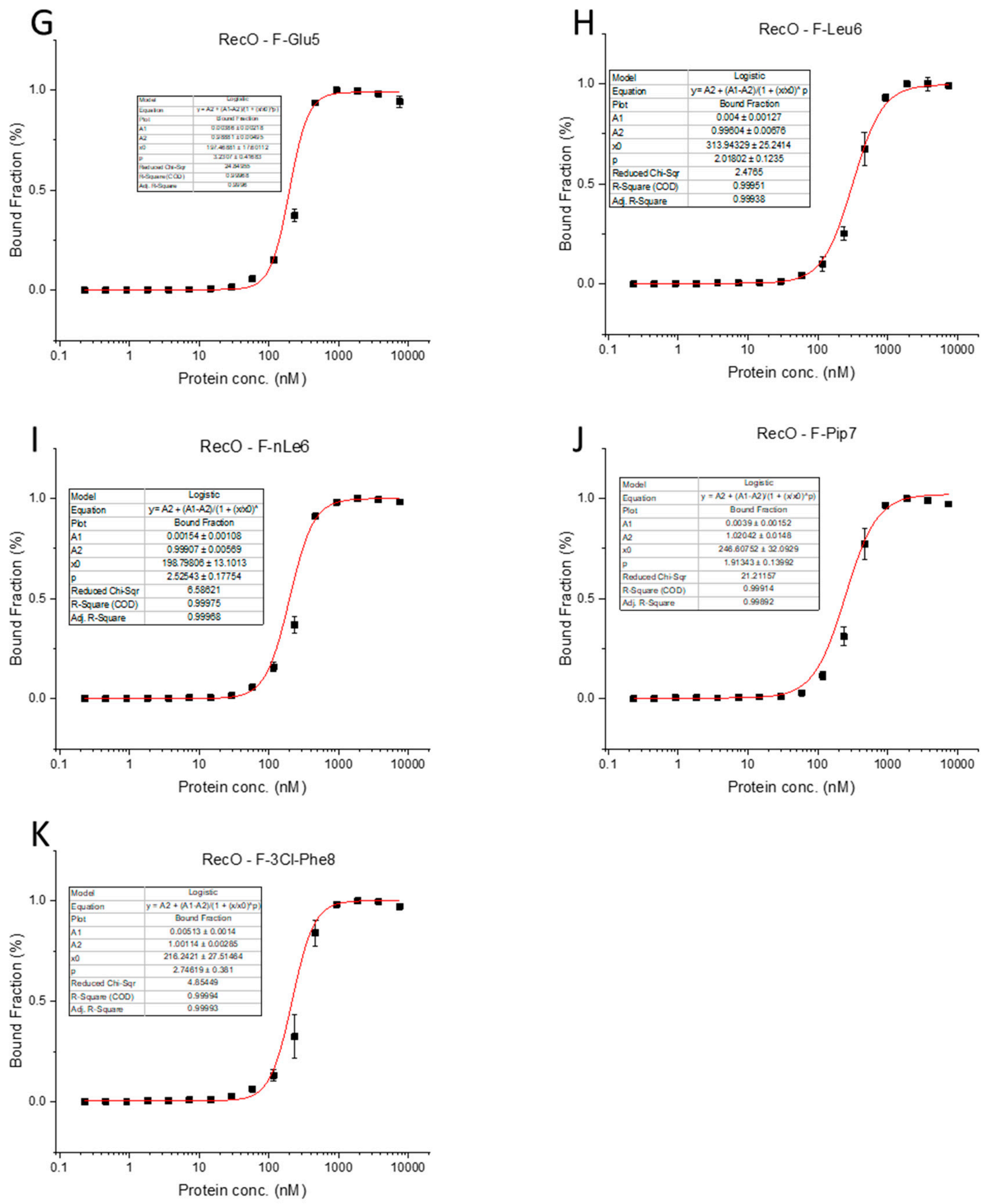

Figure S7. b. Direct titration of F-mSSBs to RecO. Validation of competitive fluorescence anisotropy hits.

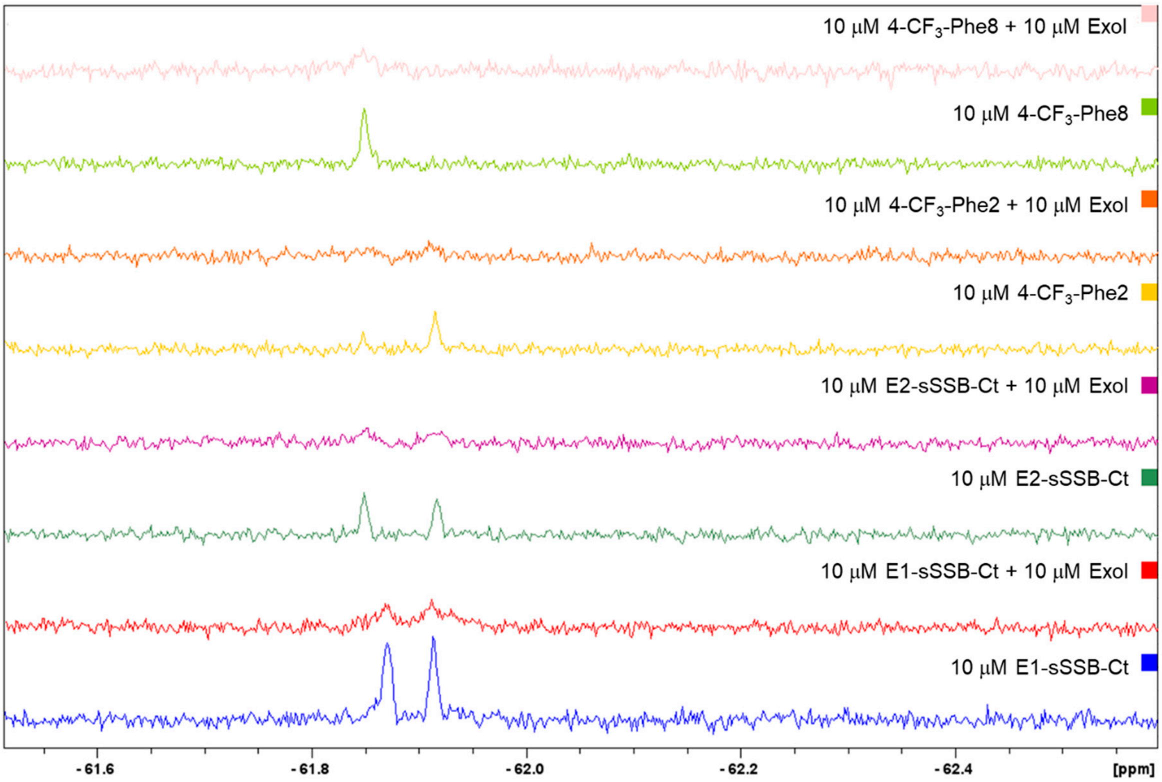

Figure S8. ^{19}F NMR measurements carried out for E1-sSSB-Ct, and the single mutated derivatives 4-CF_3-Phe8 and 4-CF_3-Phe2 with and without ExoI.

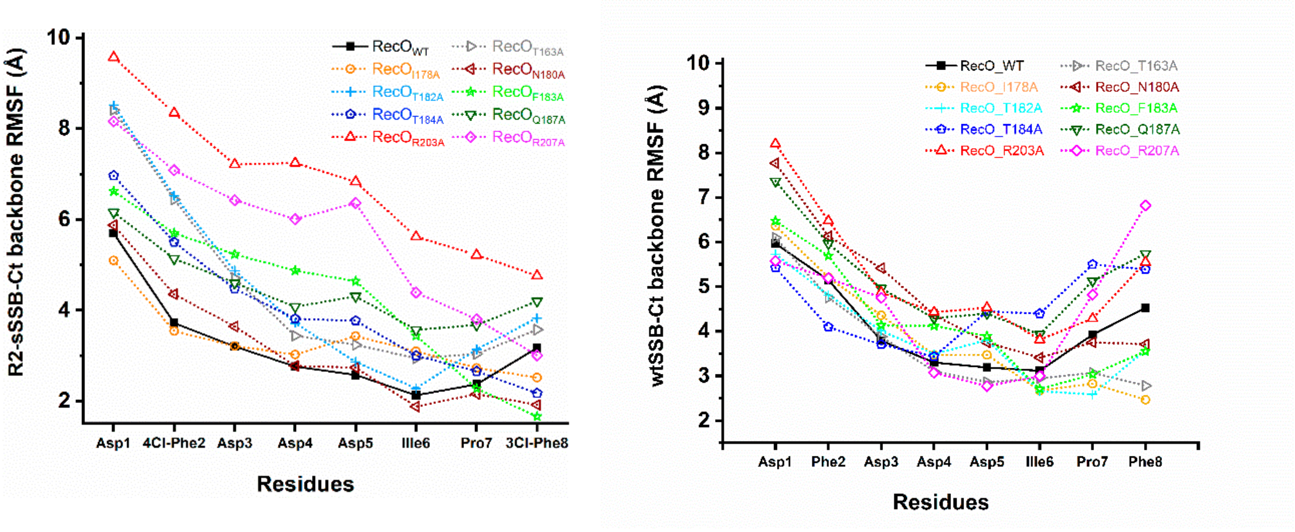

(A) (B)

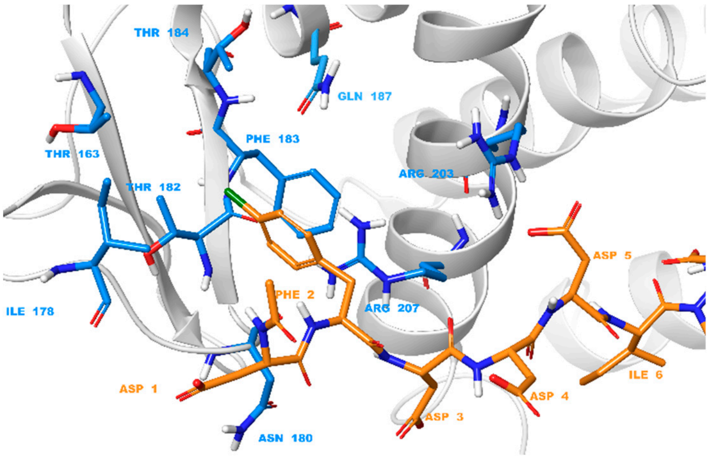

(C)

Figure S9. RMSF values of backbone atom coordinates of A) R2-sSSB-Ct and B) wtSSB-Ct in complex with the wild-type and Ala-mutated RecO proteins. Mutated residues: Thr163, Ile178, Asn180, Thr182, Phe183, Thr184, Gln187, Arg203, and Arg207. C) A binding pose model of R2-sSSB-Ct (orange) in complex with RecO and the positions of the Ala-mutated residues (blue).

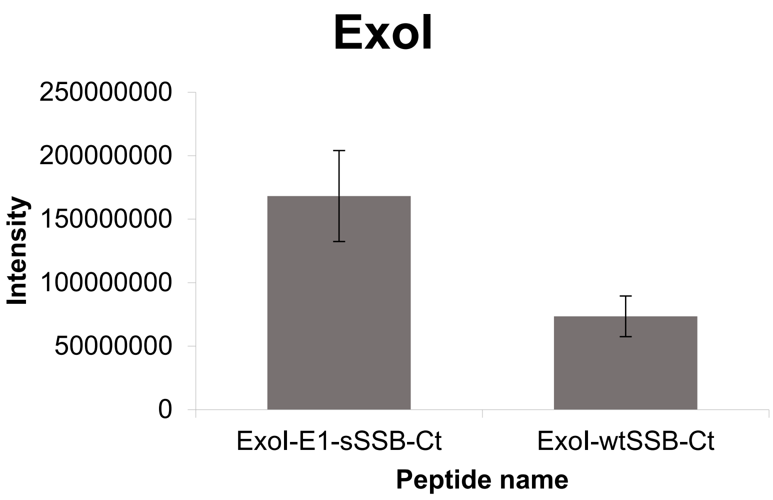

(A)

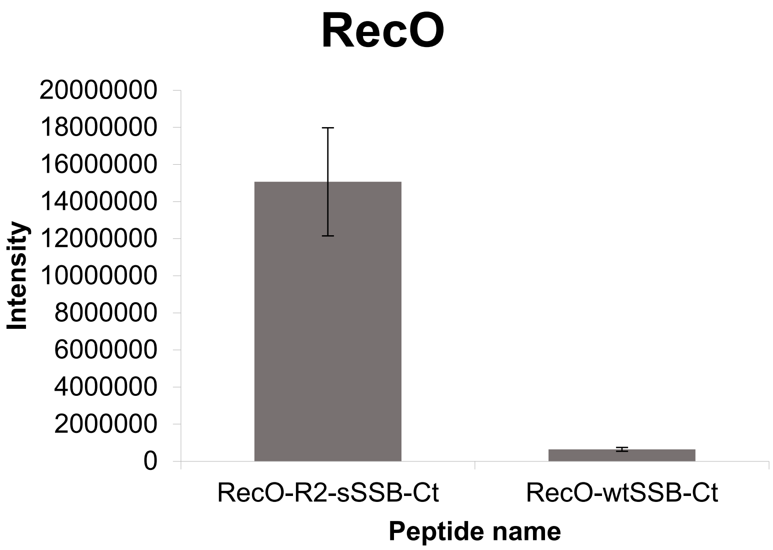

(B)

Figure S10. Protein enrichment values are shown as intensities from pull-down experiments using wtSSB-Ct and sSSB-Ct peptides. Recombinant protein (ExoI or RecO) containing lysate was incubated with peptides immobilized on streptavidin beads. Pull-down assay was performed using E1-sSSB-Ct, and R2-sSSB-Ct for ExoI and RecO, respectively. wtSSB-Ct was used as a control for both proteins. Protein-enriched beads were trypsinized, and tryptic pepides were measured using HLPC-MS. ExoI shows two-fold enrichment on E1-sSSB-Ct compared with wtSSB-Ct (A). RecO was enriched on R2-sSSB-Ct compared with wtSSB-Ct (B).

Table S1. Competitive fluorescence anisotropy data of ExoI. IC_{50} values are shown in µM.

Pos	Peptide	IC_{50} (µM)
	wt	4.54 ± 0.34
1	β³-Asp	4.47 ± 0.25
	D-Asp	5.55 ± 0.19
	Glu	1.33 ± 0.09
	Asn	9.31 ± 0.4
	NM-Asp	2.17 ± 0.15
2	β³-Phe	3.8 ± 0.11
	3-Cl-Phe	2.03 ± 0.51
	4-Cl-Phe	0.56 ± 0.03
	D-Phe	3.49 ± 2.41
	4-F-Phe	1.93 ± 0.81
	NM-Phe	5.37 ± 0.53
	3Pal	7.47 ± 0.88
	Thi	4.22 ± 0.81
	Tic	6.6 ± 0.55
	4-CF3-Phe	0.33 ± 0.02
3	β³-Asp	3.65 ± 0.16
	D-Asp	6.82 ± 0.53
	Glu	4.03 ± 0.24
	Asn	6.36 ± 0.36
	NM-Asp	3.88 ± 0.13
4	β³-Asp	1.54 ± 0.13
	D-Asp	3.08 ± 0.07
	Glu	3.58 ± 0.11
	Asn	6.02 ± 0.3
	NM-Asp	1.27 ± 0.14
5	β³-Asp	13.94 ± 6.11
	D-Asp	N/A
	Glu	1.49 ± 0.1
	Asn	7.92 ± 0.62
	NM-Asp	9.3 ± 0.66
6	Aib	27.55 ± 7.9
	β³-Ile	11.28 ± 0.03
	D-Ile	56.01 ± 18.42
	Leu	0.92 ± 0.06
	nLe	2.68 ± 0.13
	NM-Ile	3.95 ± 0.54
	tBuGly	2.87 ± 0.26
7	D-Pro	61.54 ± 16.18
	4-cF-Pro	3.79 ± 0.36
	Hyp	18.97 ± 3.07
	7Pip	0.89 ± 0.14

8	β-Phe	N/A
	3-Cl-Phe	3.4 ± 2.41
	4-Cl-Phe	1.41 ± 0.18
	D-Phe	N/A
	4-F-Phe	3.04 ± 0.64
	NM-Phe	N/A
	3Pal	19.91 ± 2.81
	Thi	7.72 ± 0.51
	Tic	96.06 ± 83.78
	4-CF3-Phe	0.39 ± 0.07
super	E1-sSSB	0.166 ± 0.11
	E2-sSSB	

Table S2. Competitive fluorescence anisotropy data of RecO. IC_{50} values are shown in µM.

Pos	Peptide	IC50 (µM)
	wt	4.66 ± 0.24
1	β3-Asp	8.03 ± 0.4
	D-Asp	6.56 ± 1.4
	Glu	5.17 ± 1.1
	Asn	13.85 ± 17.2
	NM-Asp	5.1 ± 0.64
2	β3-Phe	3.66 ± 0.61
	3-Cl-Phe	3.44 ± 0.14
	4-Cl-Phe	2.38 ± 0.27
	D-Phe	2.75 ± 0.59
	4-F-Phe	3.05 ± 0.28
	NM-Phe	3.33 ± 0.35
	3Pal	11.68 ± 0.8
	Thi	5.27 ± 0.28
	Tic	3.99 ± 0.1
	4-CF3-Phe	3.27 ± 0.42
3	β3-Asp	8.92 ± 0.39
	D-Asp	4.56 ± 1.53
	Glu	5.81 ± 1.65
	hSe	25.05 ± 10.02
	Asn	14.21 ± 1.8
	NM-Asp	1.88 ± 0.09
4	β3-Asp	19.59 ± 2.78
	D-Asp	5.92 ± 0.91
	Glu	5.68 ± 0.59
	Asn	15.7 ± 3.54
	NM-Asp	3.85 ± 1.03
5	β3-Asp	5.91 ± 0.68
	D-Asp	N/A
	Glu	1.41 ± 0.55
	Asn	16.01 ± 0.51
	NM-Asp	9.33 ± 4.3
6	Aib	N/A
	β3-Ile	19.21 ± 7.47

	D-Ile	N/A
	Leu	3.23 ± 0.16
	nLe	2.55 ± 0.23
	NM-Ile	9.36 ± 0.08
	tBuGly	10.42 ± 1.02
7	D-Pro	11.62 ± 10.94
	4-cF-Pro	240.77 ± 199.68
	Hyp	34.56 ± 10.94
	7Pip	3.31 ± 0.44
8	β3-Phe	N/A
	3-Cl-Phe	0.36 ± 0.03
	4-Cl-Phe	3.62 ± 0.84
	D-Phe	N/A
	4-F-Phe	4.07 ± 7.98
	NM-Phe	64.67 ± 18.92
	3Pal	31.87 ± 4.16
	Thi	8.59 ± 7.26
	Tic	141.27 ± 8.37
	4-CF3-Phe	5.97 ± 2.43
super	R1-sSSB	3.33 ± 0.28
	R2-sSSB	0.59 ± 0.05

Table S3. Mass spectrometry data of SSB analogs.

	compound	MW	calculated m/z	detected m/z
1	wtSSB	1025.02	1024.40	1025.40
2	β³-Asp1	1039.05	1038.42	1039.41
3	D-Asp1	1025.02	1024.40	1025.13
4	Glu1	1039.05	1038.42	1039.25
5	Asn1	1024.04	1023.42	1024.15
6	NM-Asp1	1039.05	1038.42	1039.10
7	β³-Phe2	1039.05	1038.42	1039.38
8	3-Cl-Phe2	1059.47	1058.36	1059.21
9	4-Cl-Phe2	1059.47	1058.36	1059.07
10	D-Phe2	1025.02	1024.40	1025.08
11	4-F-Phe2	1043.01	1042.39	1043.16
12	NM-Phe2	1039.05	1038.42	1039.15
13	3Pal2	1026.01	1025.40	1026.29
14	Thi2	1031.05	1030.36	1031.09
15	Tic2	1037.03	1036.41	1037.11
16	4-CF3-Phe2	1093.02	1092.39	1093.08
17	β³-Asp3	1039.05	1038.42	1093.31
18	D-Asp3	1025.02	1024.40	1025.07
19	Glu3	1039.05	1038.42	1039.23
20	Asn3	1024.04	1023.42	1024.29
21	NM-Asp3	1039.05	1038.42	1039.11
22	β³-Asp4	1039.05	1038.42	1039.36
23	D-Asp4	1025.02	1024.40	1025.04
24	Glu4	1039.05	1038.42	1039.16
25	Asn4	1024.04	1023.42	1024.19

26	NM-Asp4	1039.05	1038.42	1039.05
27	β3-Asp5	1039.05	1038.42	1039.22
28	D-Asp5	1025.02	1024.40	1025.13
29	Glu5	1039.05	1038.42	1039.19
30	Asn5	1024.04	1023.42	1024.28
31	NM-Asp5	1039.05	1038.42	1039.22
32	Aib6	996.97	996.37	996.87
33	β3-Ile6	1039.05	1038.42	1039.06
34	D-Ile6	1025.02	1024.40	1025.04
35	Leu6	1025.02	1024.40	1025.17
36	NM-Ile6	1039.05	1038.42	1038.9
37	nLeu6	1025.02	1024.40	1025.32
38	tBuGly6	1025.02	1024.40	1025.20
39	4-cF-Pro7	1043.01	1042.39	1043.05
40	D-Pro7	1025.02	1024.40	1025.15
41	Hyp7	1041.02	1040.40	1041.18
42	Pip7	1039.05	1038.42	1039.29
43	β3-Phe8	1039.05	1038.42	1039.16
44	3-Cl-Phe8	1059.47	1058.36	1059.23
45	4-Cl-Phe8	1059.47	1058.36	1059.19
46	D-Phe8	1025.02	1024.40	1025.08
47	4-F-Phe8	1043.01	1042.39	1043.08
48	NM-Phe8	1039.05	1038.42	1039.20
49	3Pal8	1026.01	1025.40	1026.33
50	Thi8	1031.05	1030.36	1031.1
51	Tic8	1037.03	1036.41	1037.15
52	4-CF3-Phe8	1093.02	1092.39	1093.25
53	F-wtSSB-Ct	1455.39	1454.51	1455.76
54	F-4-Cl-Phe2	1490.84	1488.47	1489.42
55	F-4-F-Phe2	1474.39	1472.5	1473.5
56	F-4-CF3-Phe2	1523.39	1522.43	1523.53
57	F-Glu5	1469.39	1468.53	1469.55
58	F-Leu6	1455.39	1454.51	1455.56
59	F-nLe6	1455.39	1454.51	1455.41
60	F-Pip7	1469.39	1468.53	1469.47
61	F-3Cl-Phe8	1490.84	1488.47	1489.44
62	F-4CF3-Phe8	1523.39	1522.43	1523.45
63	E1-sSSB-Ct	1217.12	1216.44	1217.16
64	E2-sSSB-Ct	1161.02	1160.38	1161.38
65	R1-sSSB-Ct	1121.97	1120.36	1121.24
66	R2-sSSB-Ct	1093.91	1092.32	1093.11
67	K(Btn)-wtSSB-Ct	1337.45	1336.56	1337.80
68	K(Btn)-E1-sSSB-Ct	1529.56	1528.60	1530.07
69	K(Btn)-R2-sSSB-Ct	1406.34	1404.49	1407.67
70	Ac-DFDD-NH2	551.50	551.19	552.20
71	Ac-DIPF-OH	532.59	532.25	533.26

Compound 1 – wtSSB

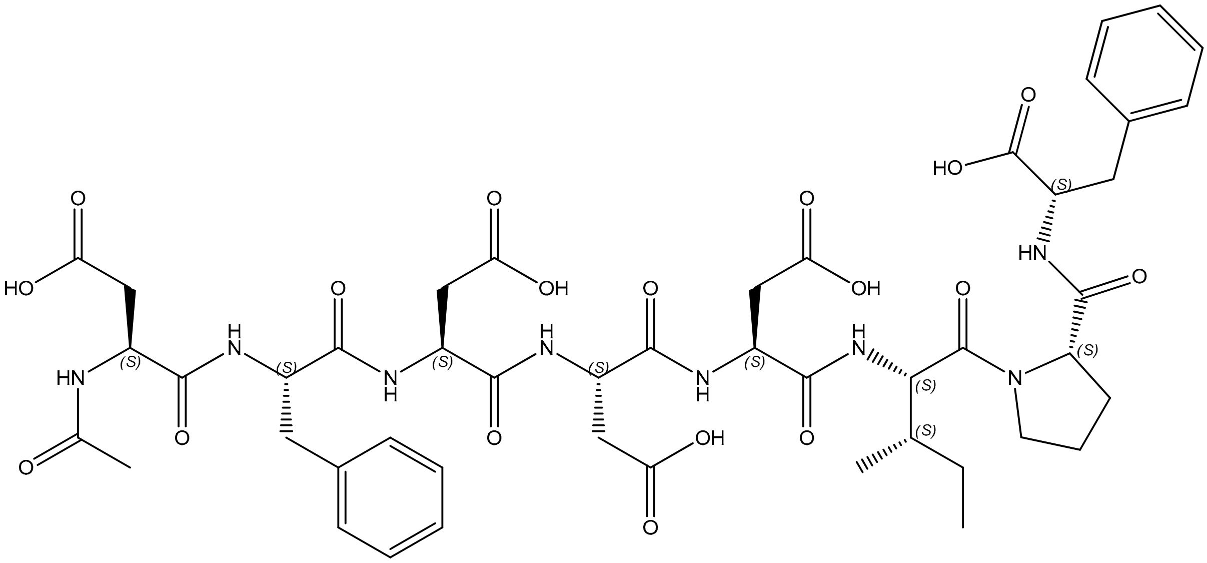

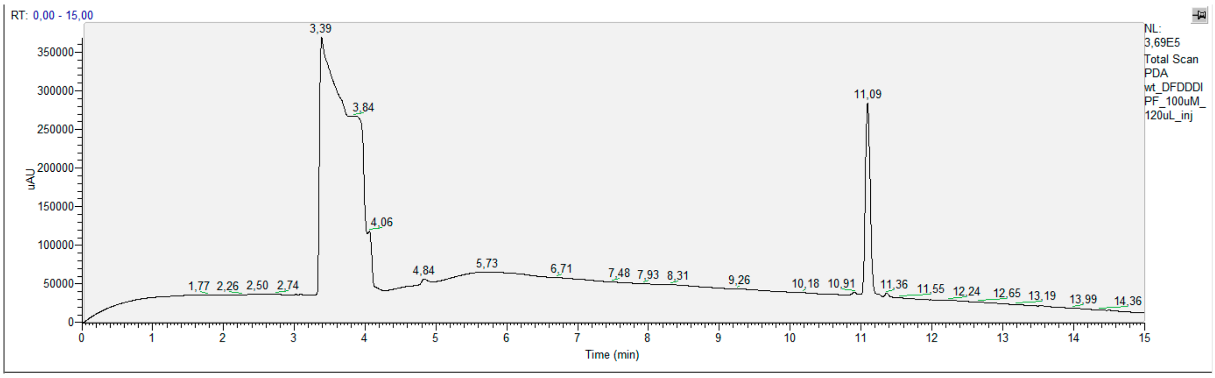

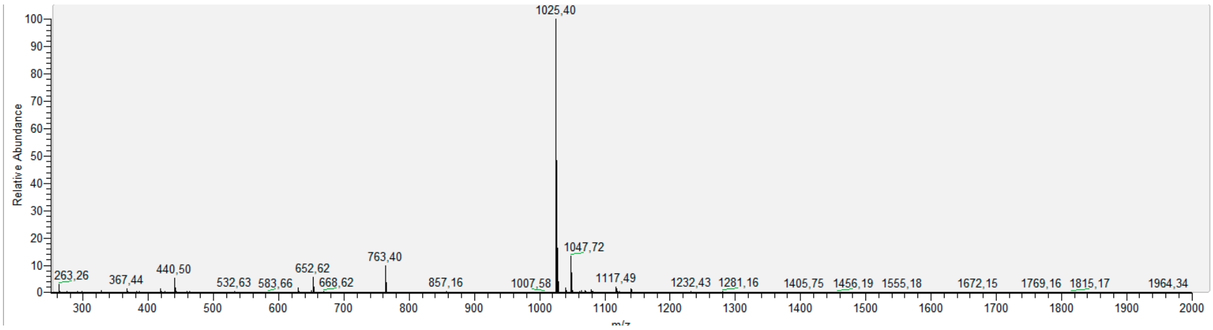

Compound 2 – β³-Asp1

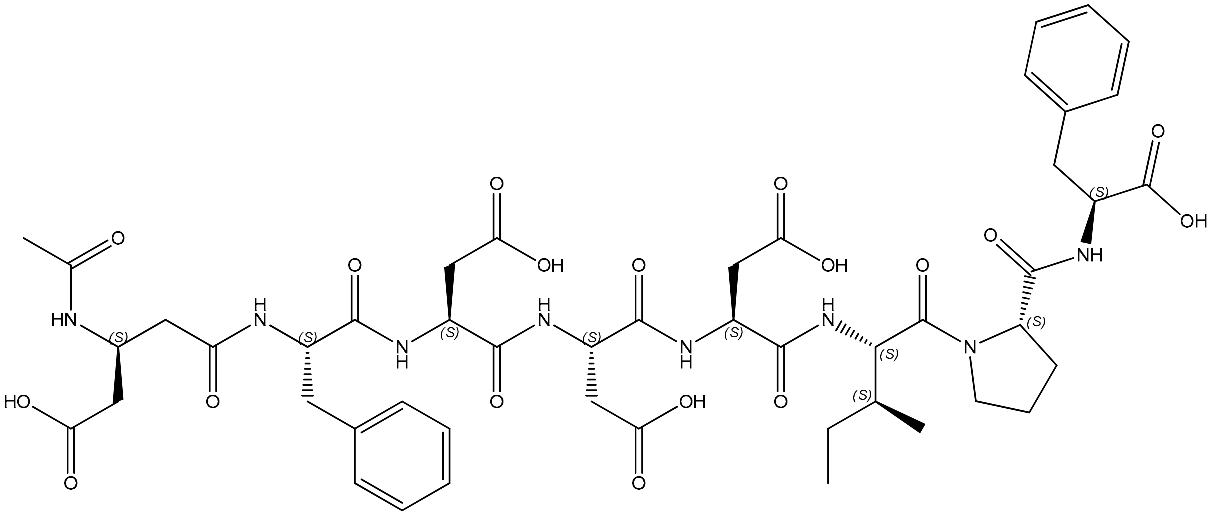

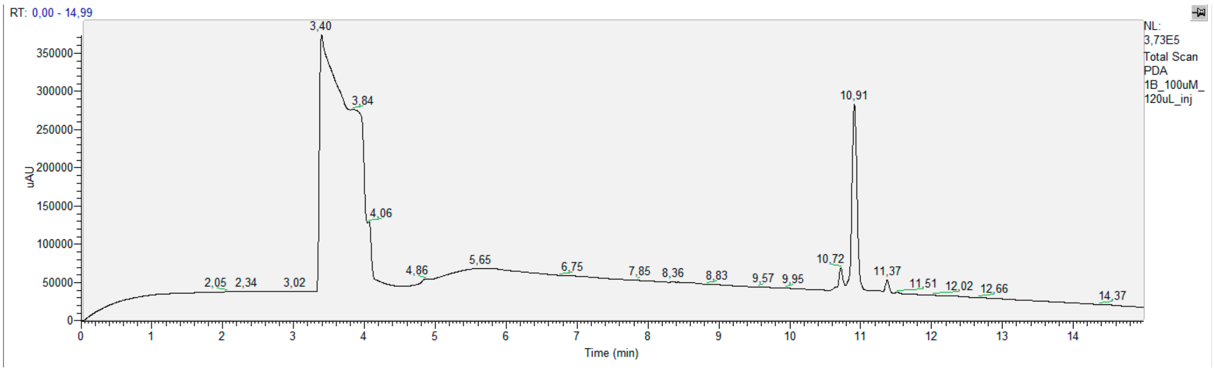

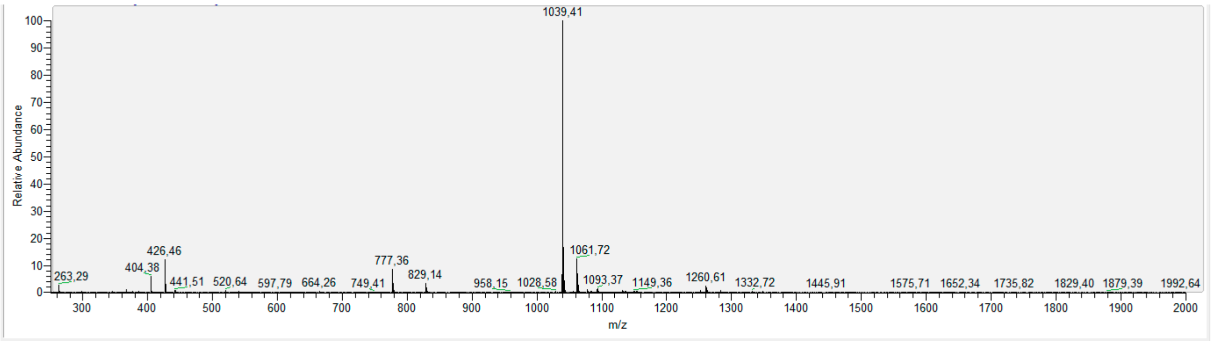

Compound 3 – D-Asp1

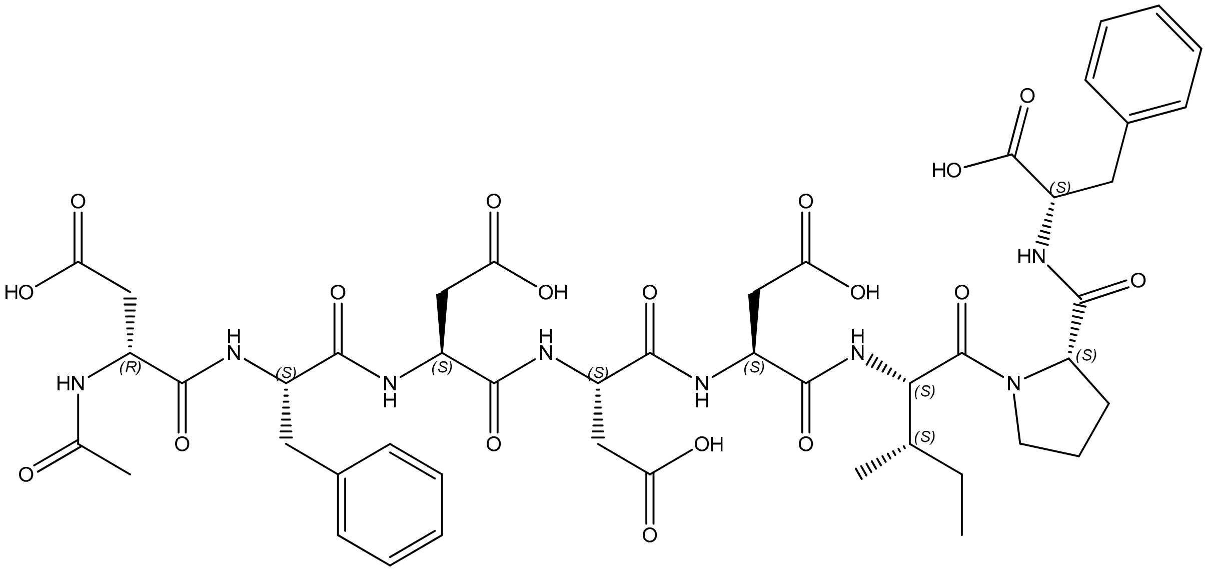

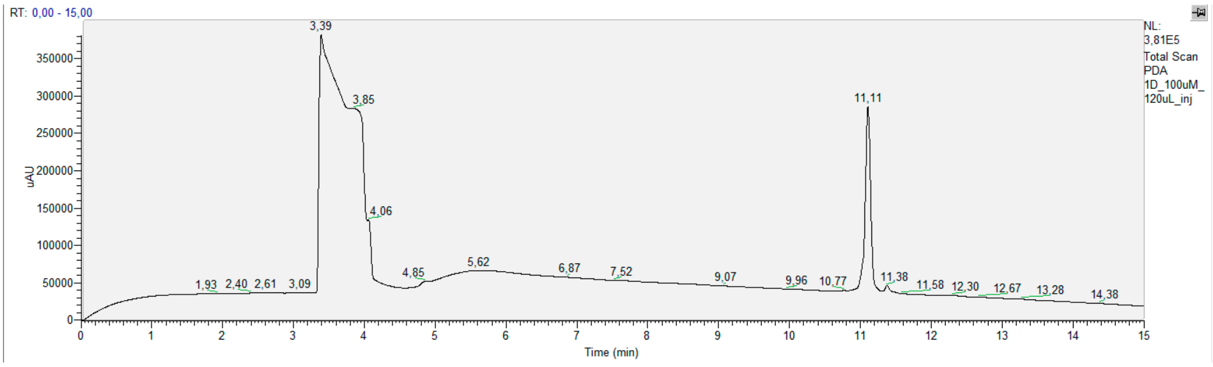

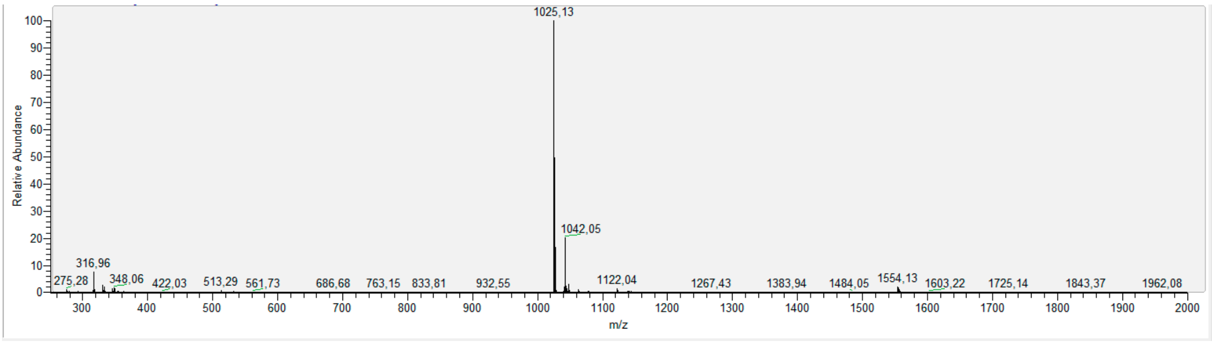

Compound 4 – Glu1

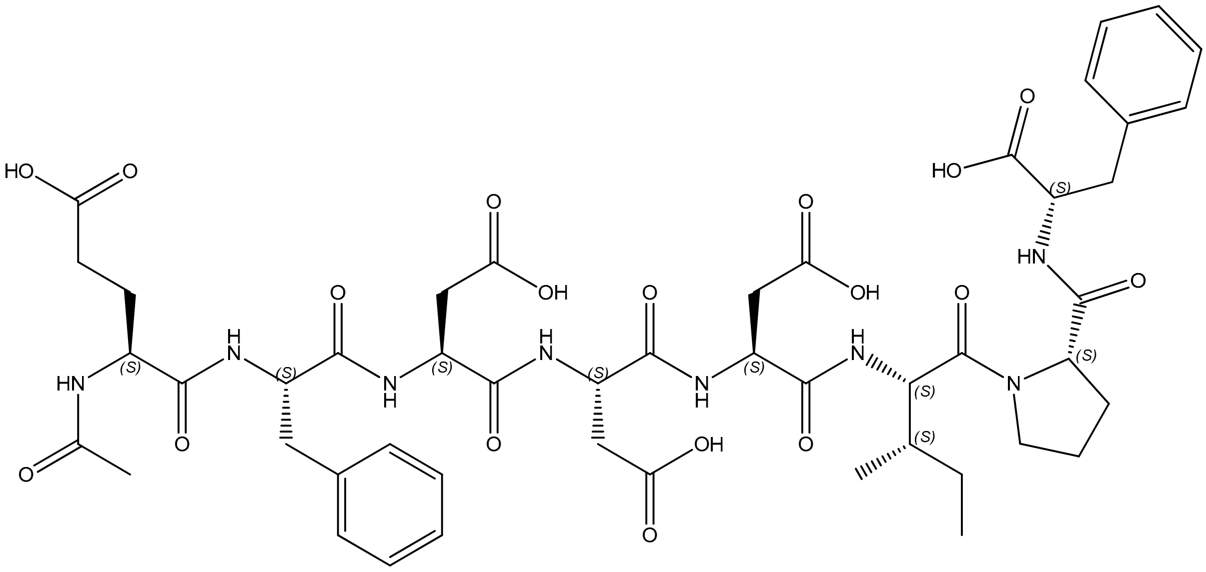

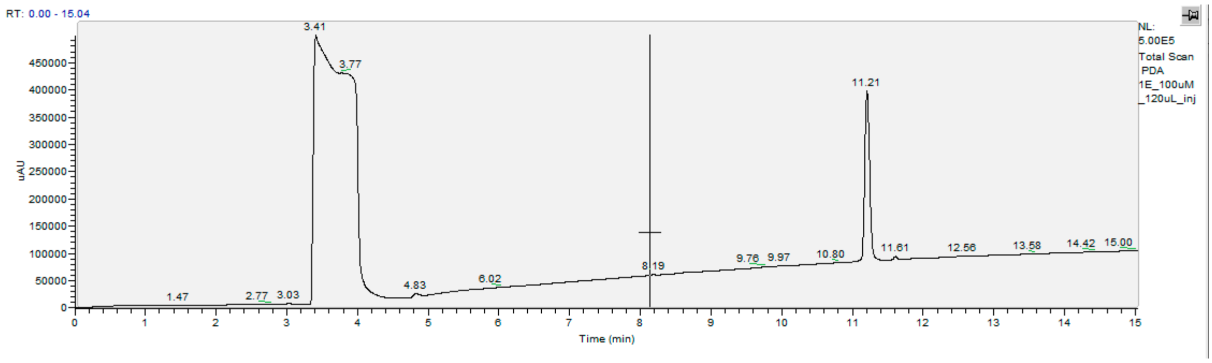

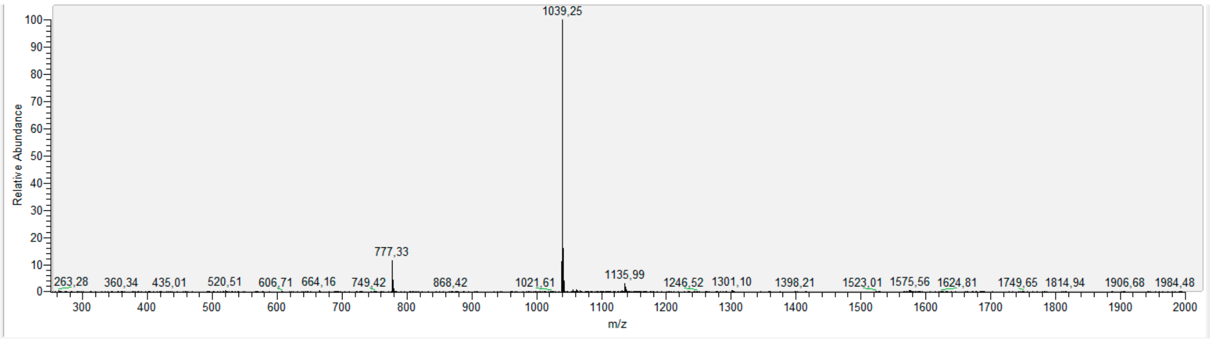

Compound 5 – Asn1

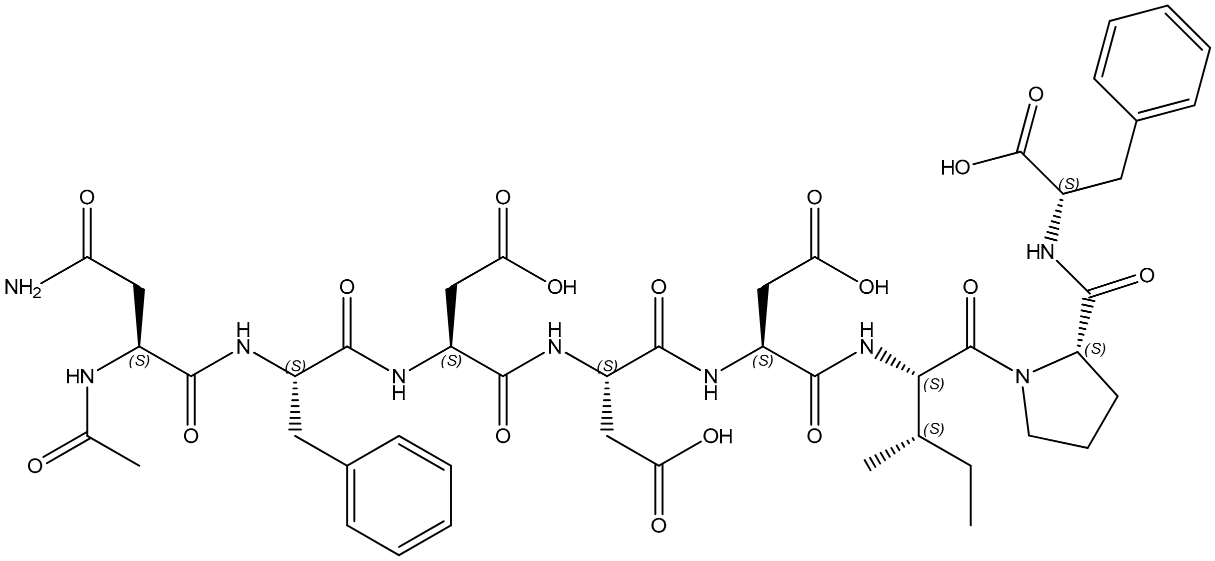

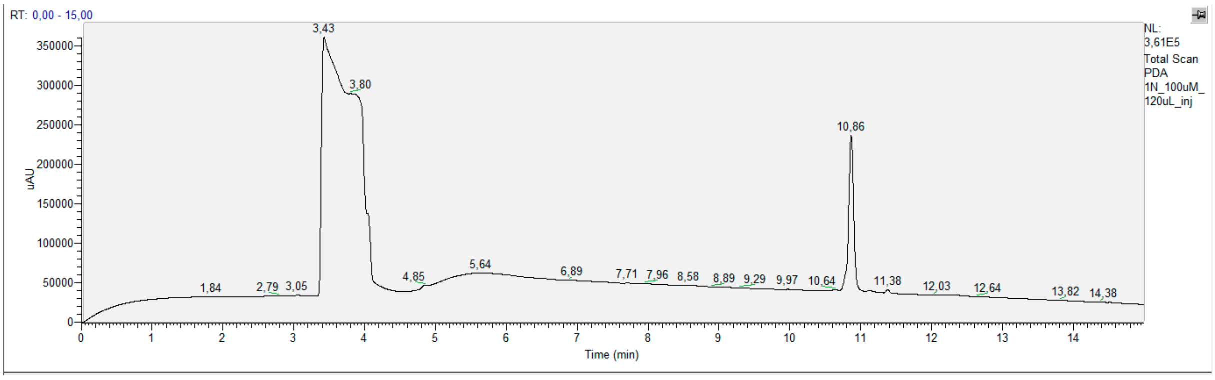

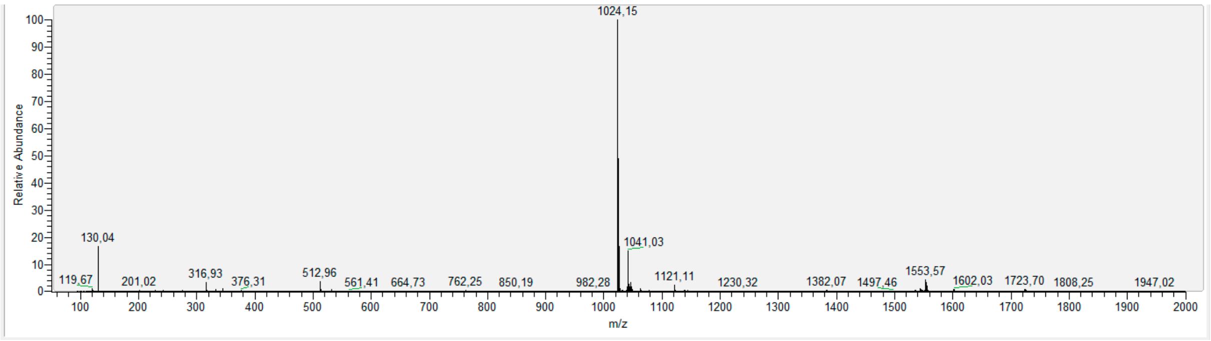

Compound 6 – NM-Asp1

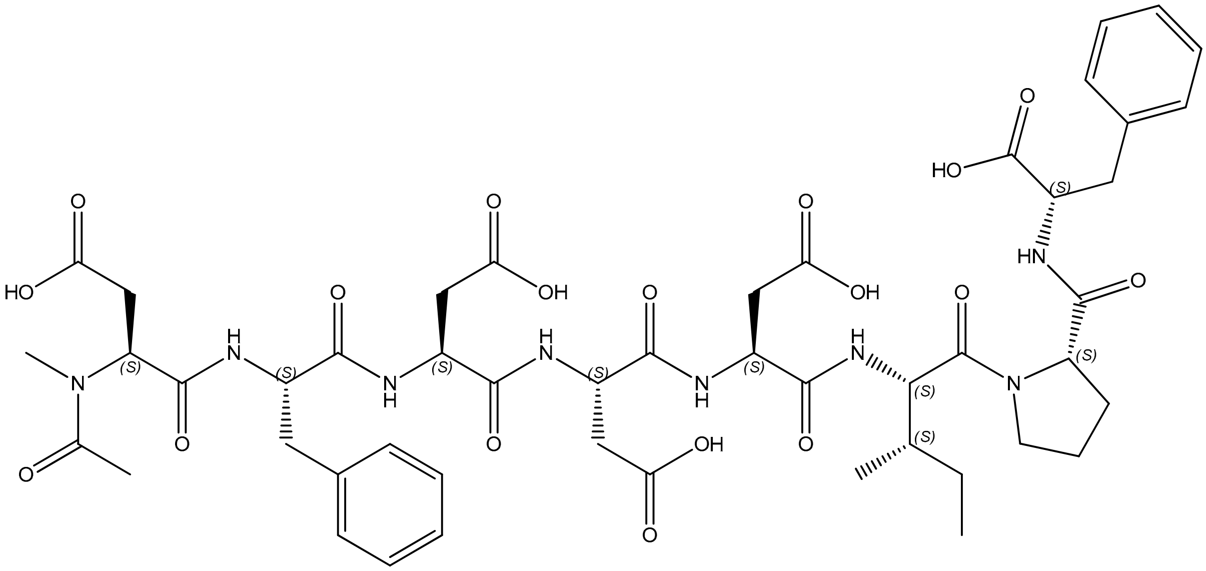

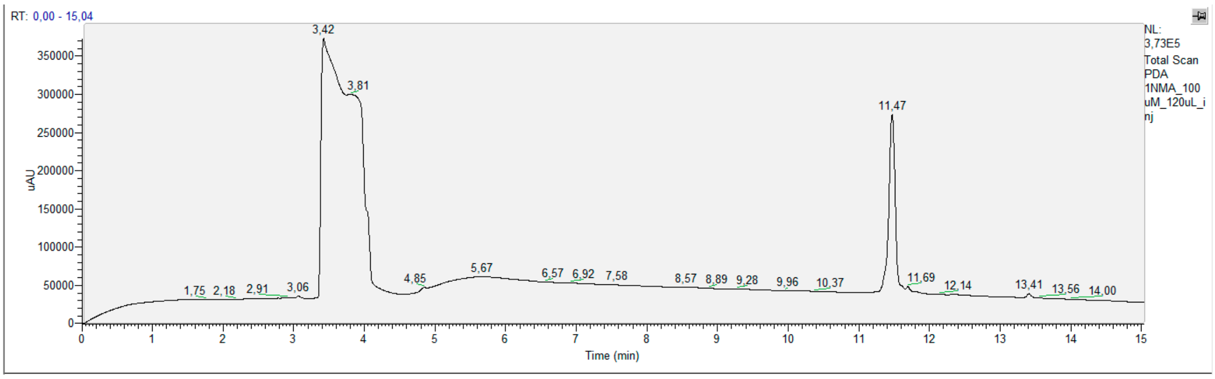

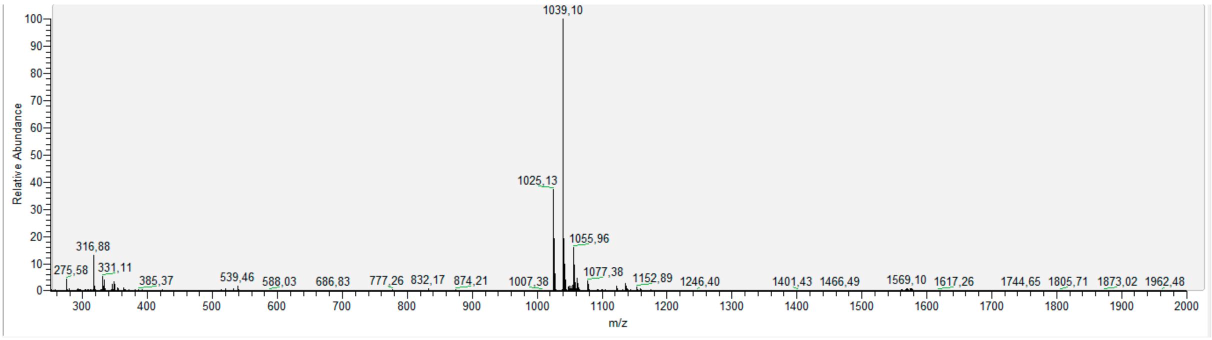

Compound 7 – β 3-Phe2

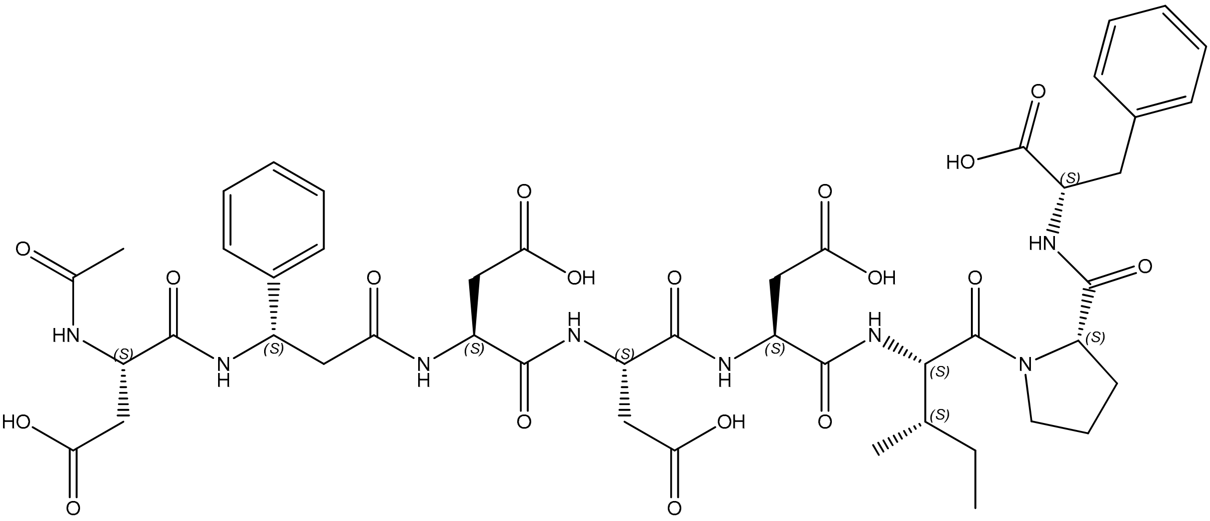

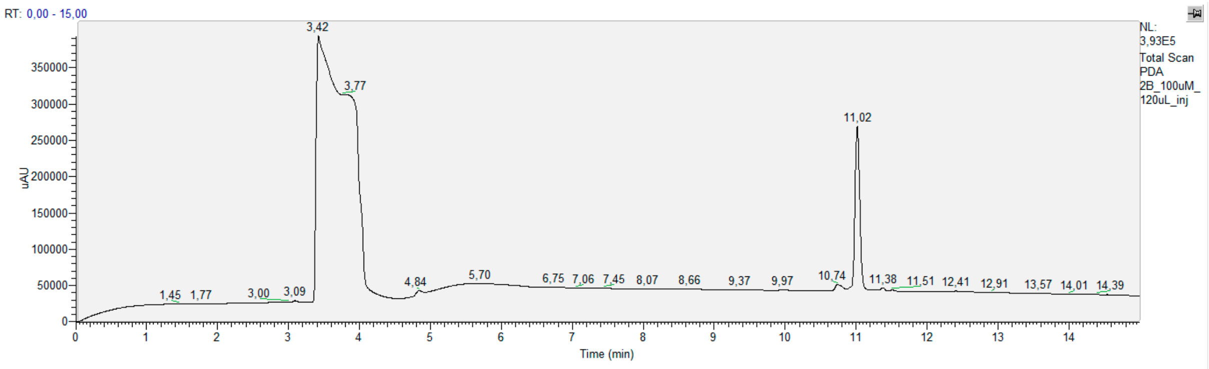

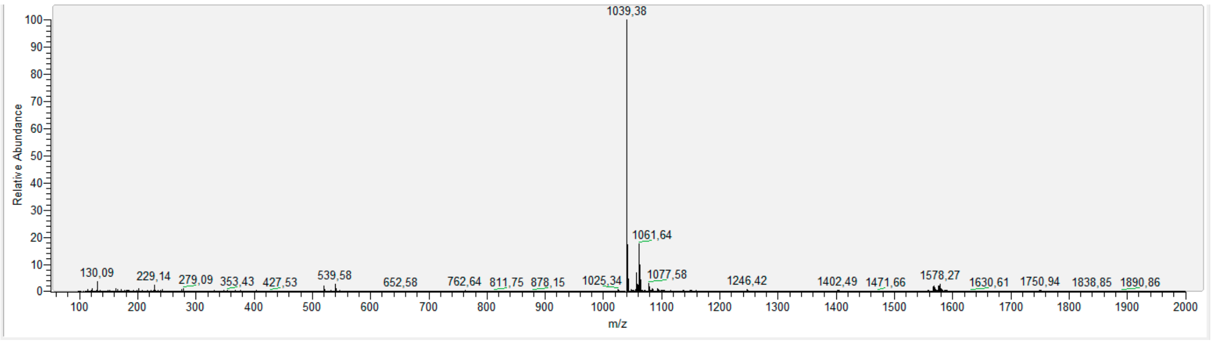

Compound 8 – 3Cl-Phe2

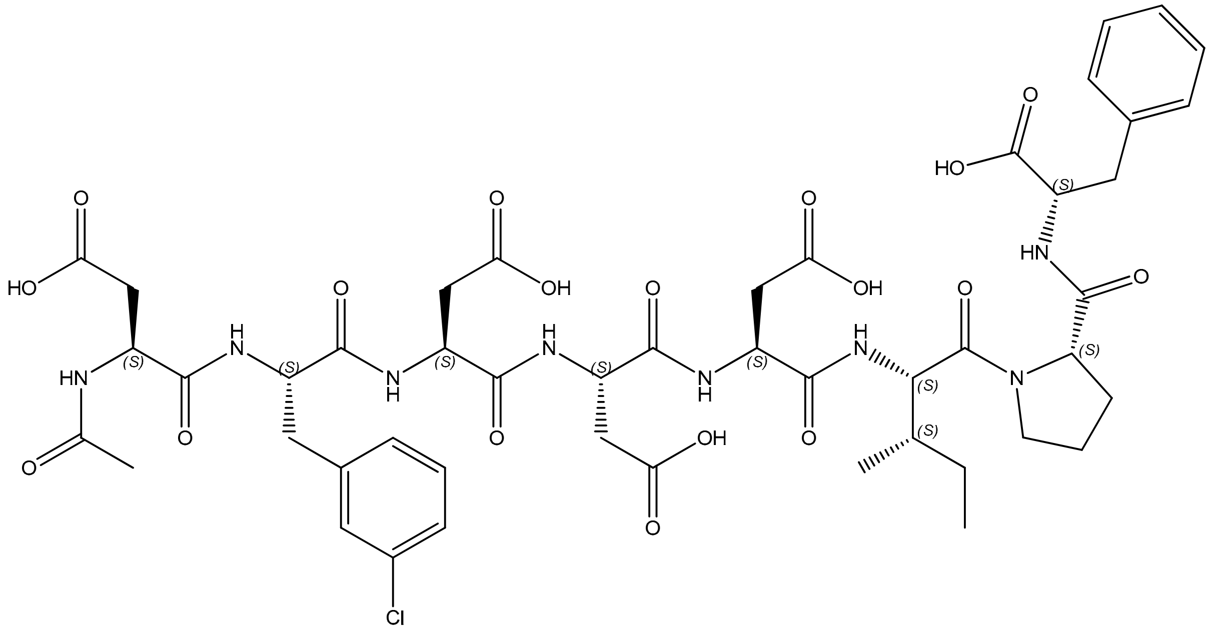

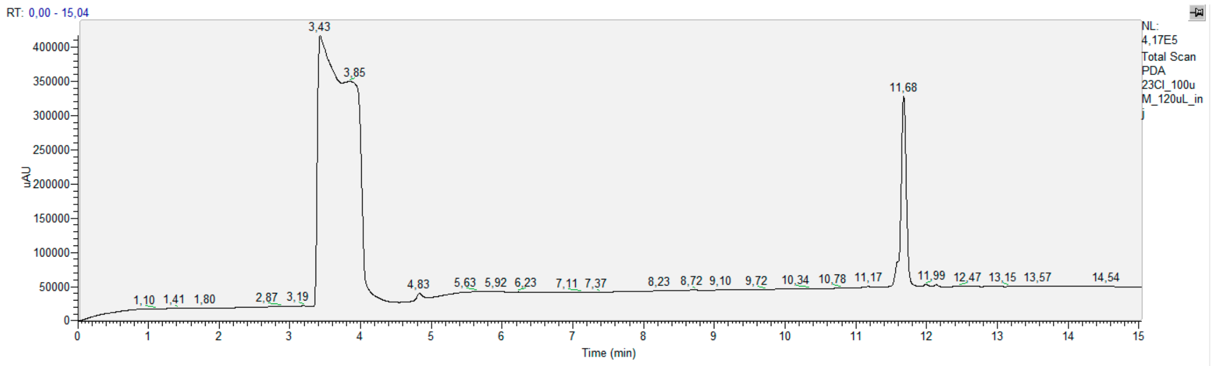

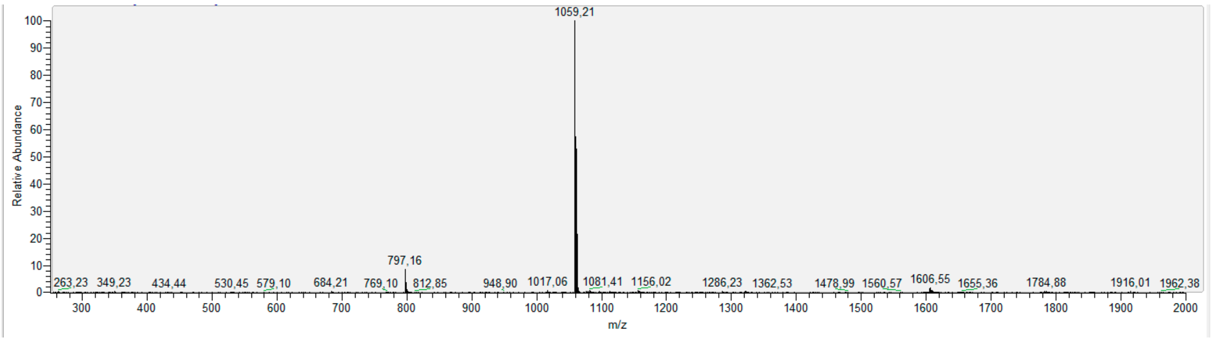

Compound 9 – 4Cl-Phe2

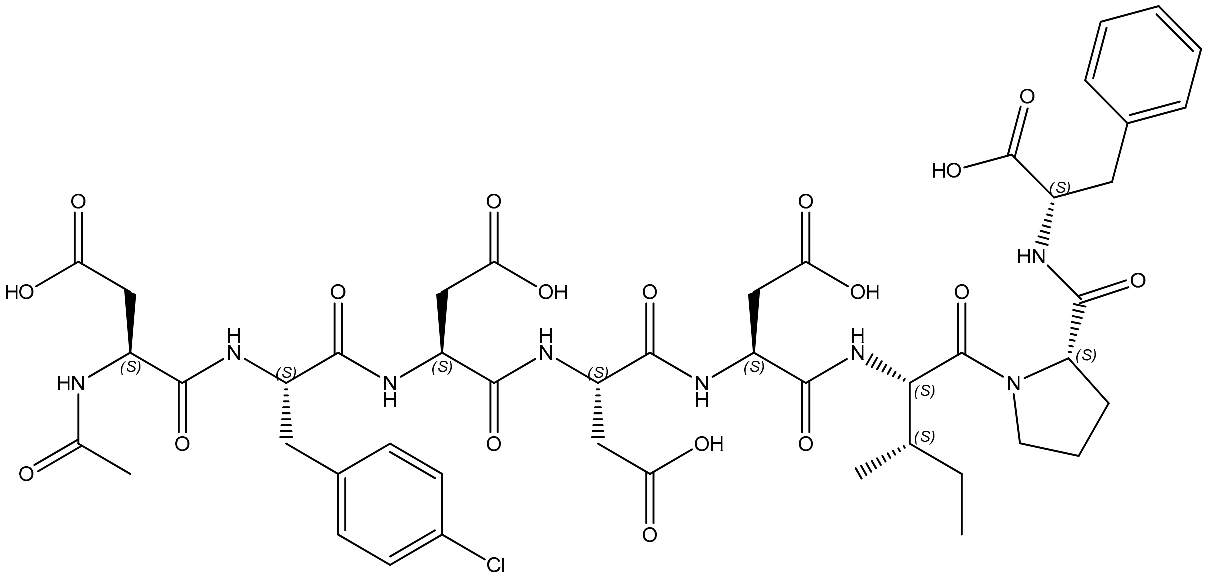

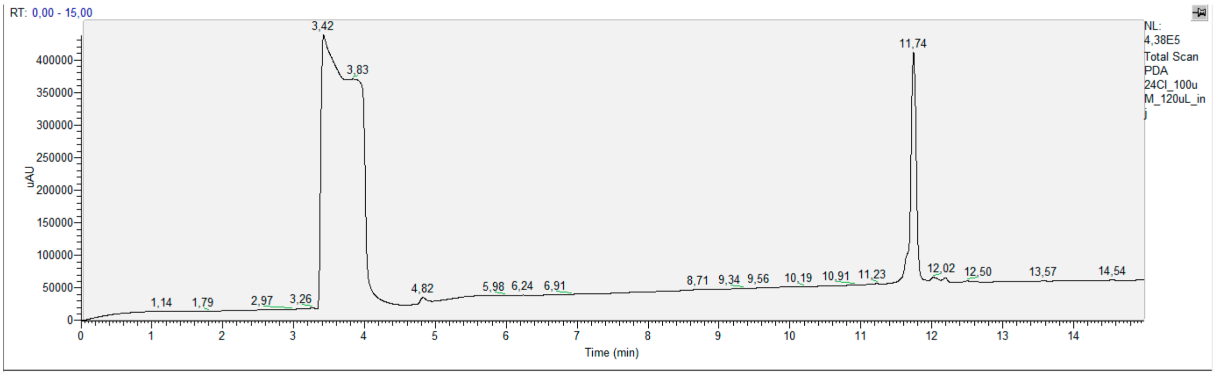

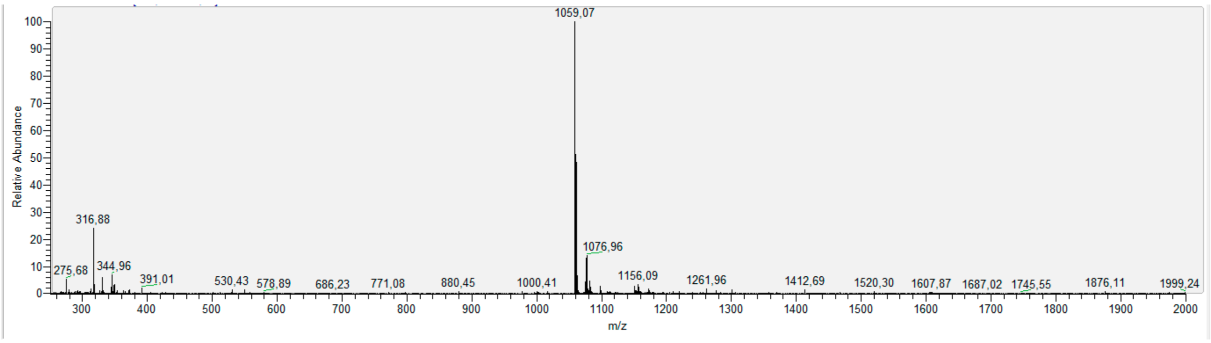

Compound 10 – D-Phe2

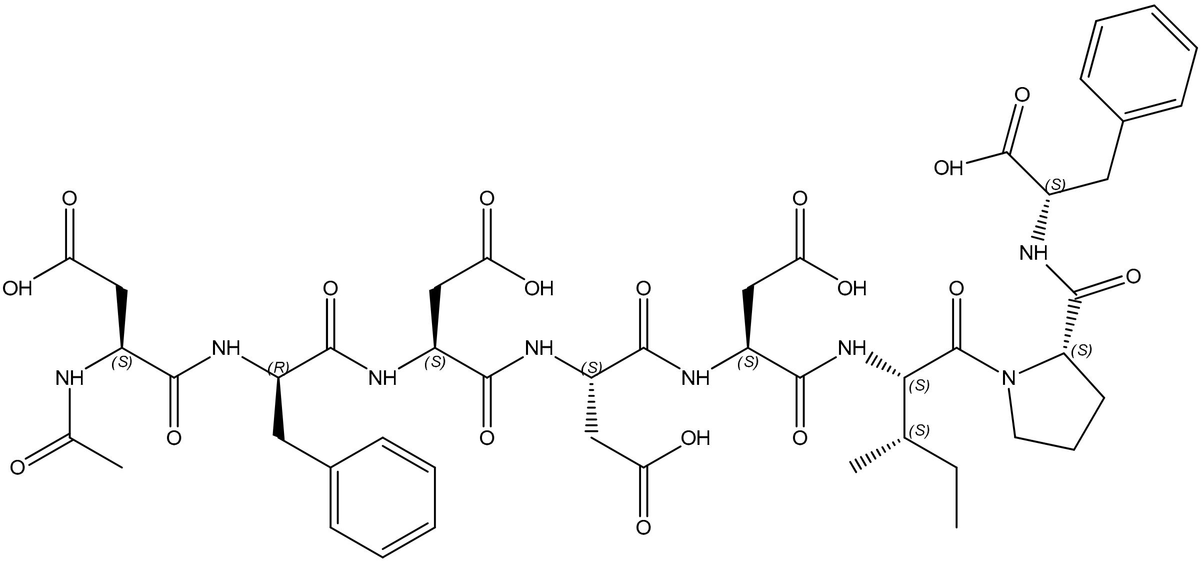

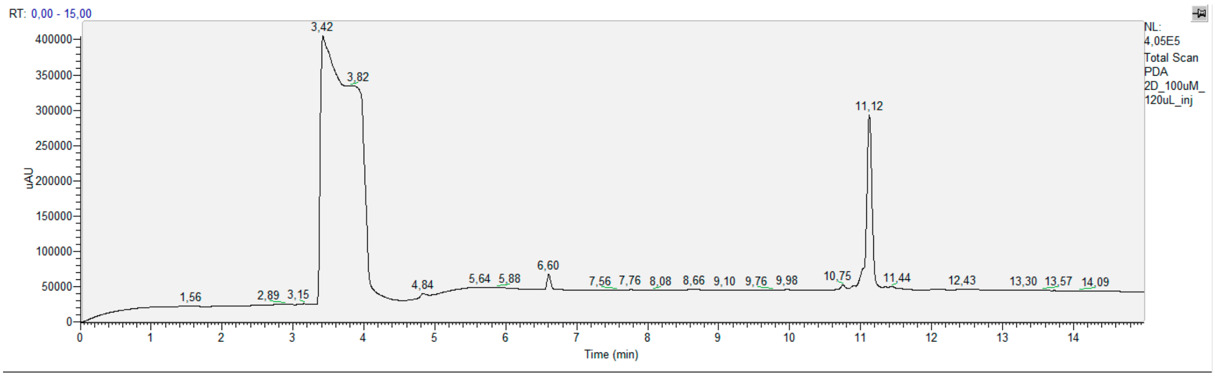

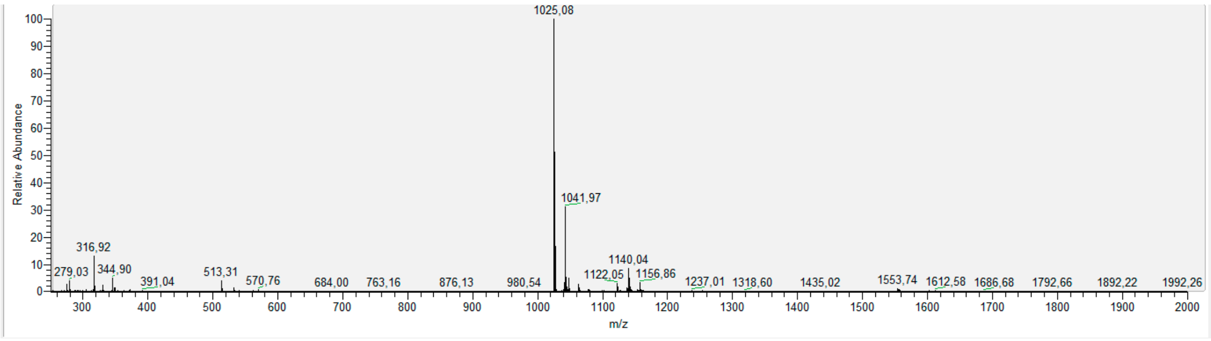

Compound 11 – 4F-Phe2

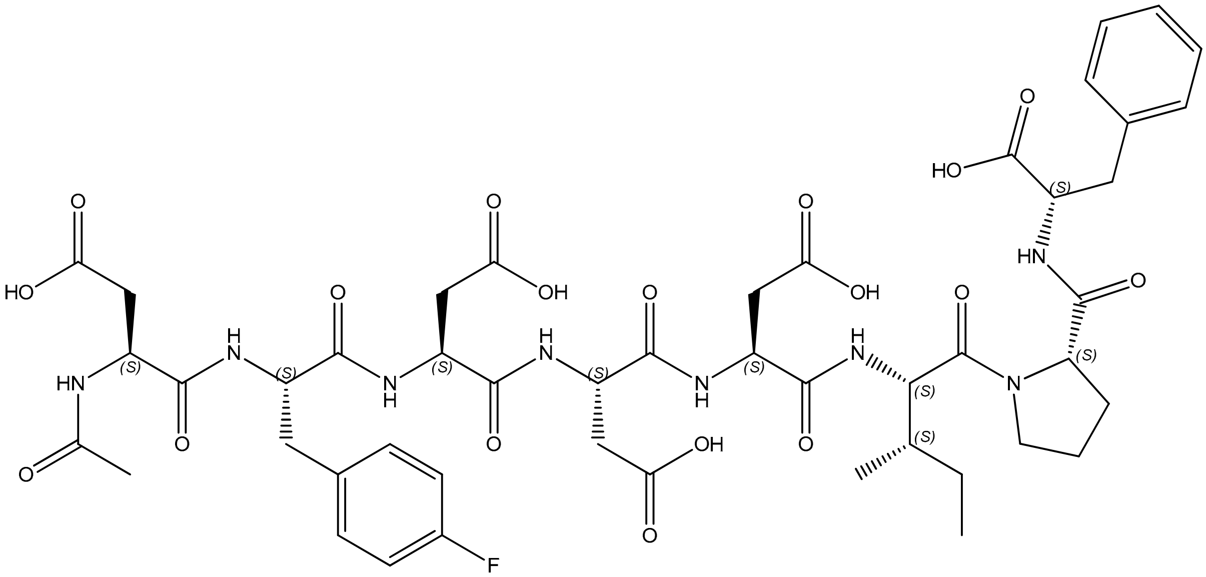

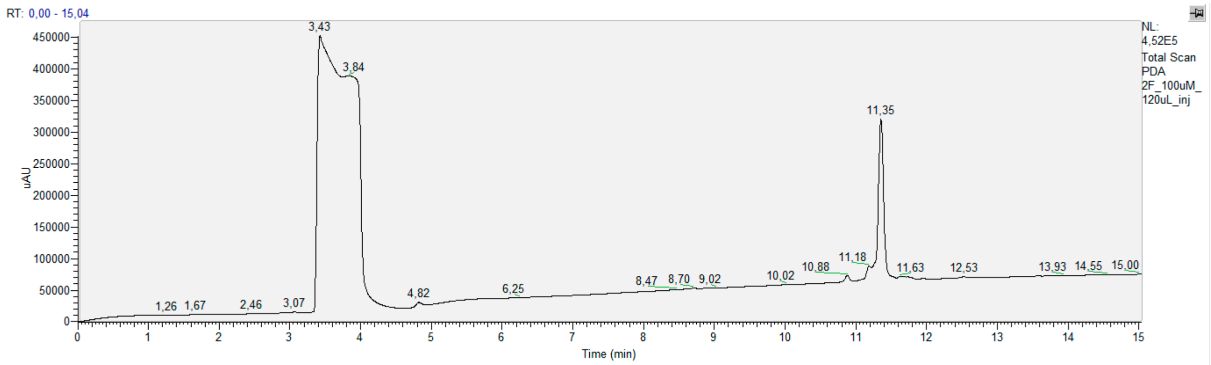

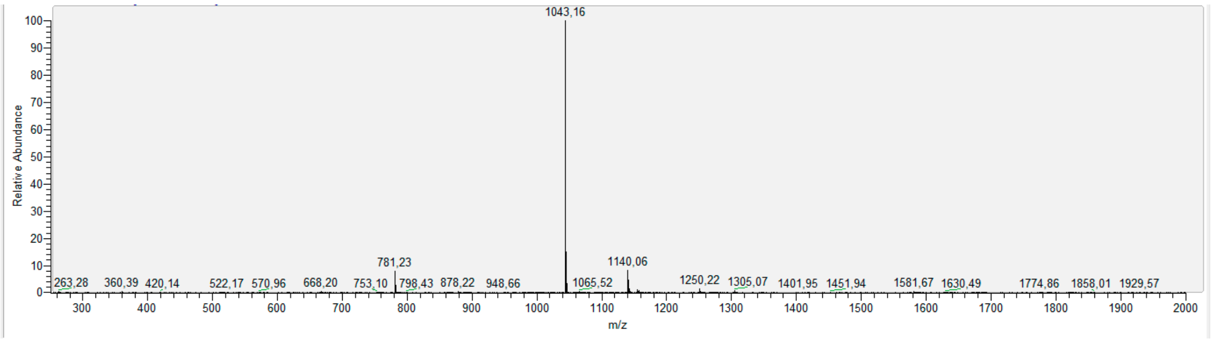

Compound 12 – NM-Phe2

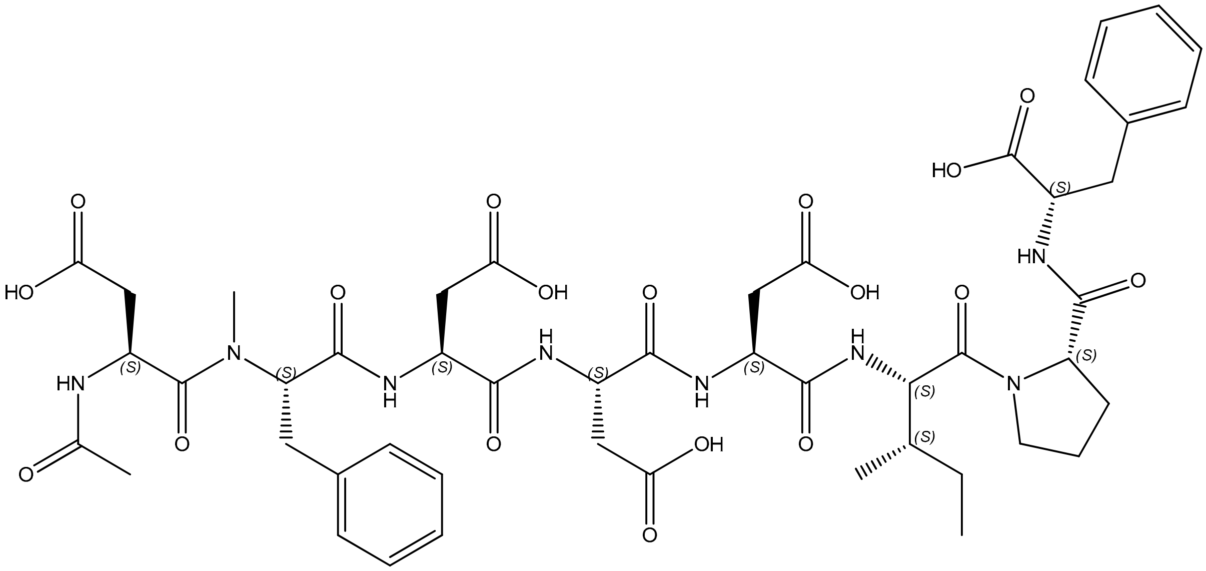

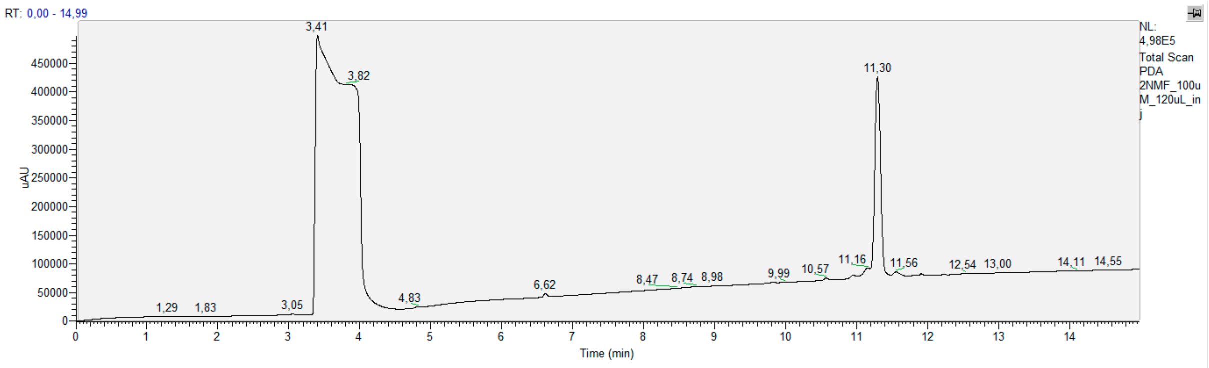

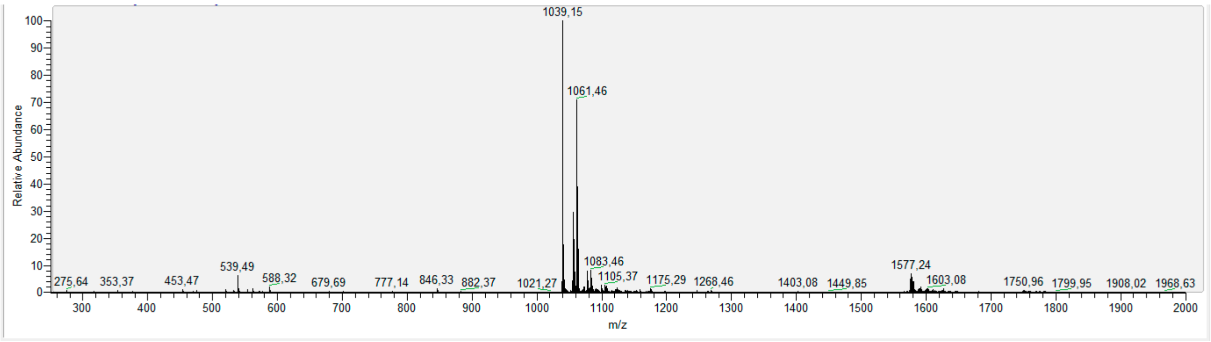

Compound 13 – 3Pal2

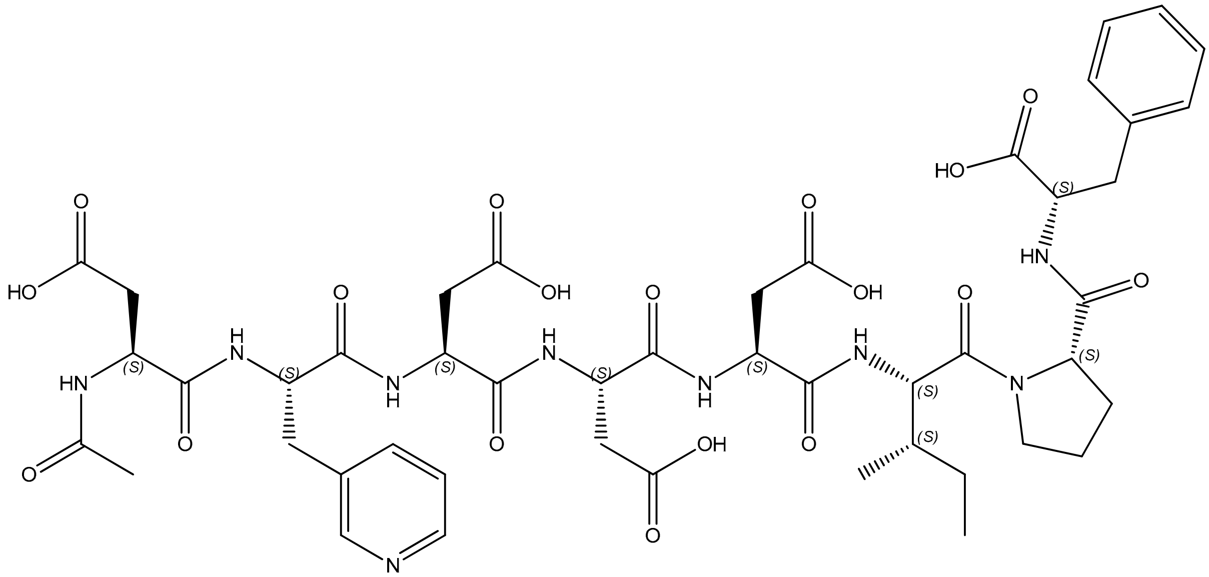

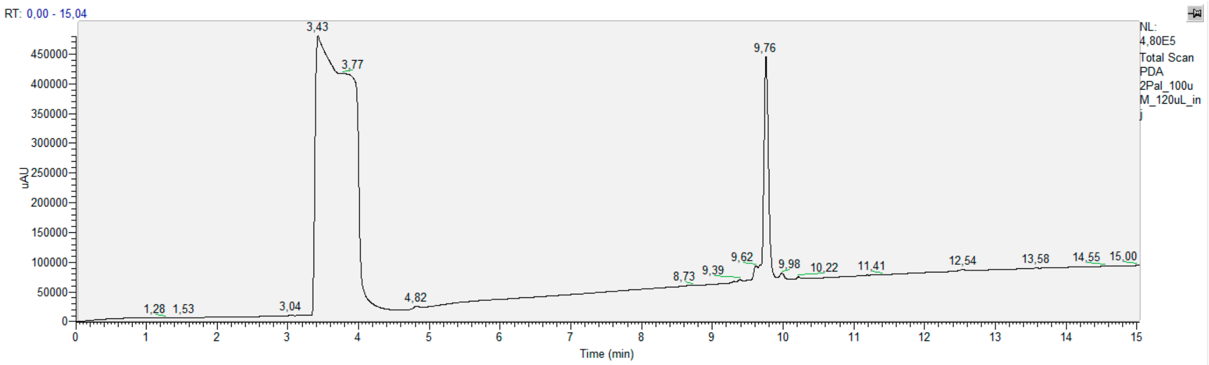

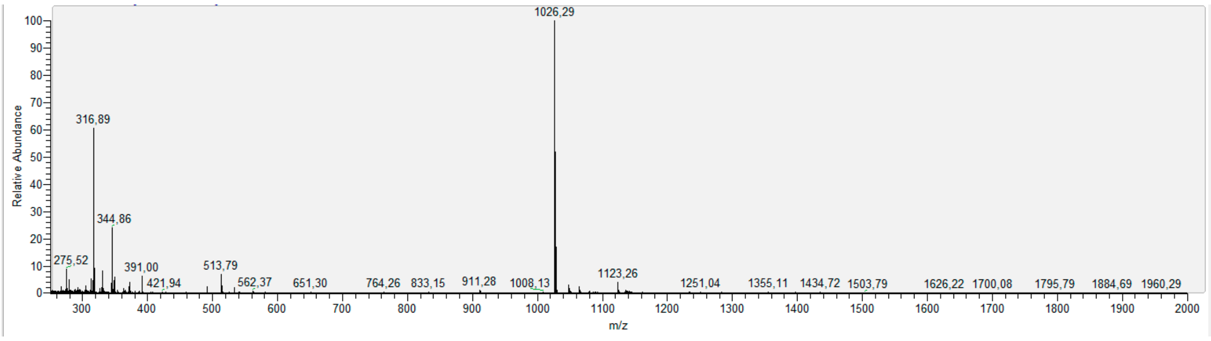

Compound 14 – Thi2

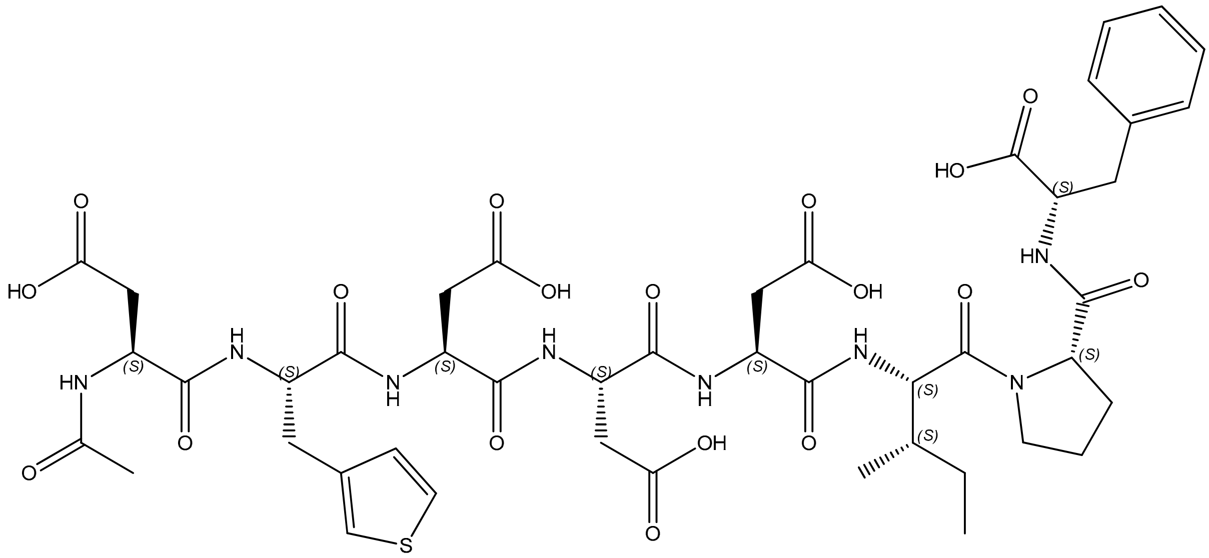

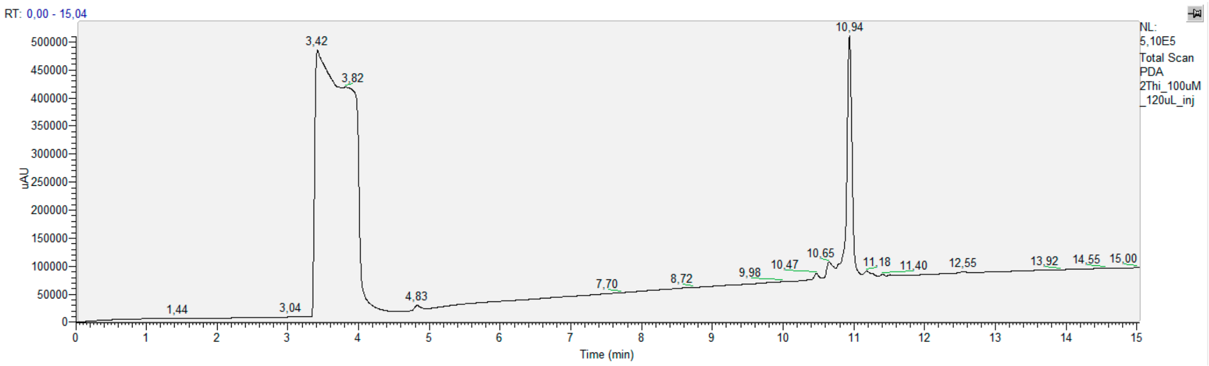

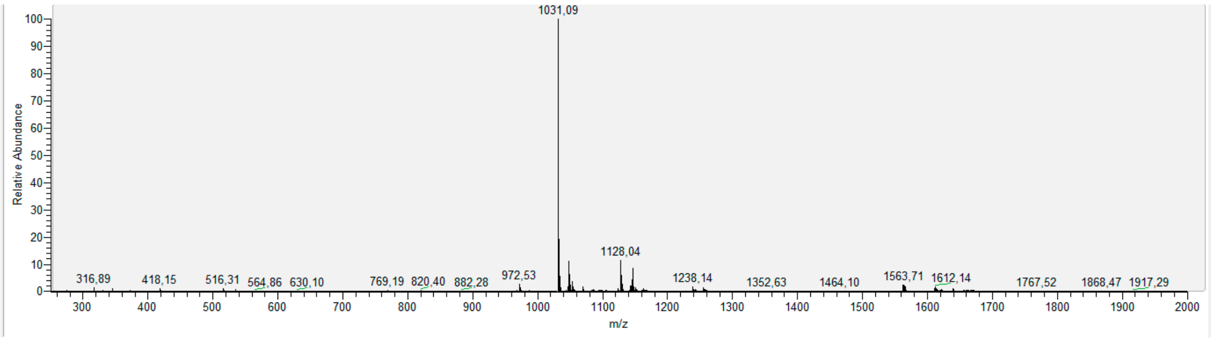

Compound 15 – Tic2

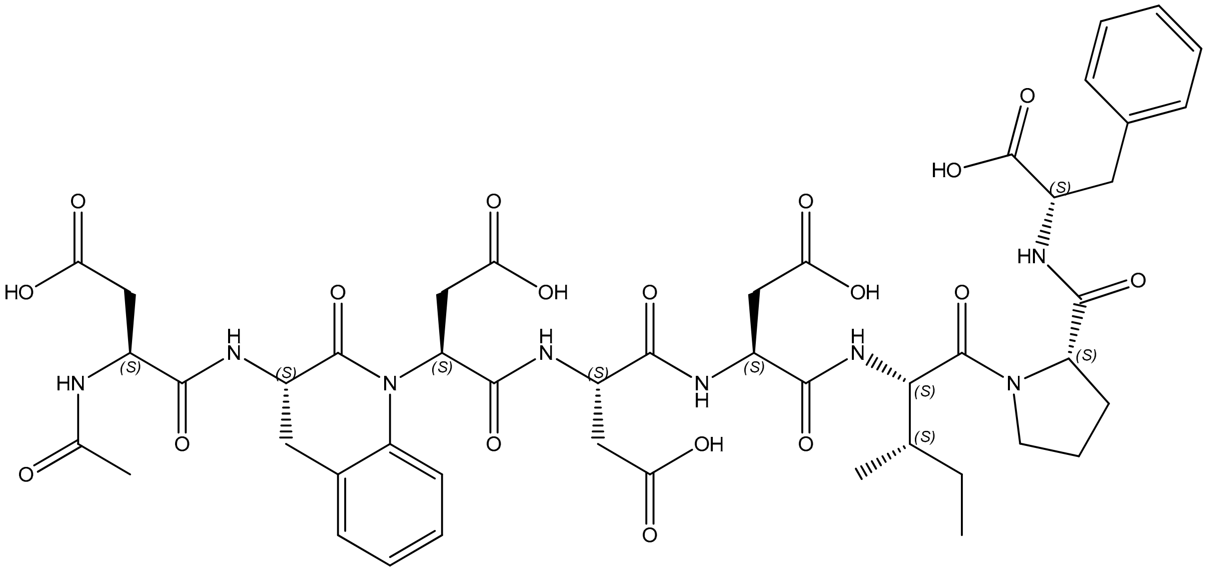

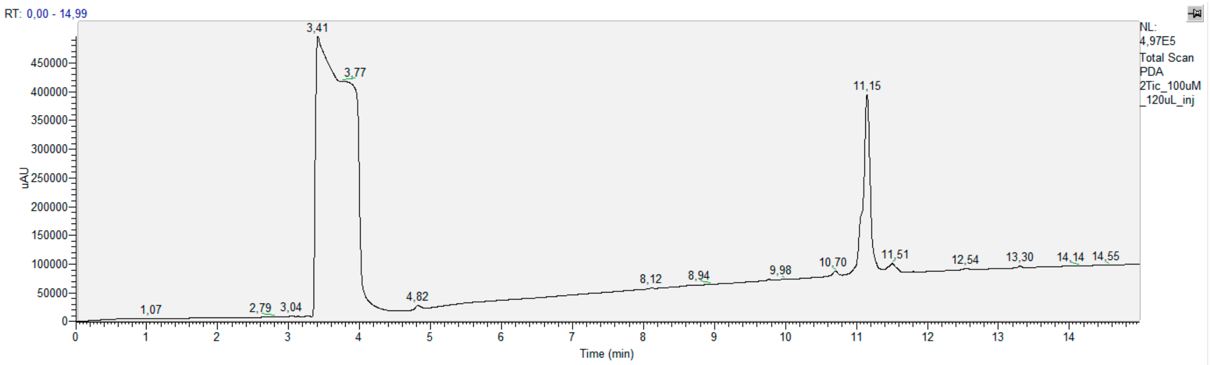

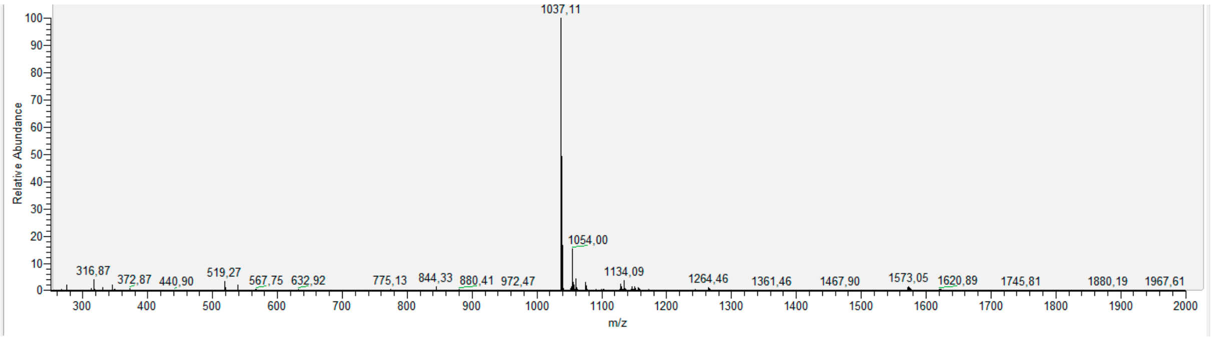

Compound 16 – 4CF3-Phe2

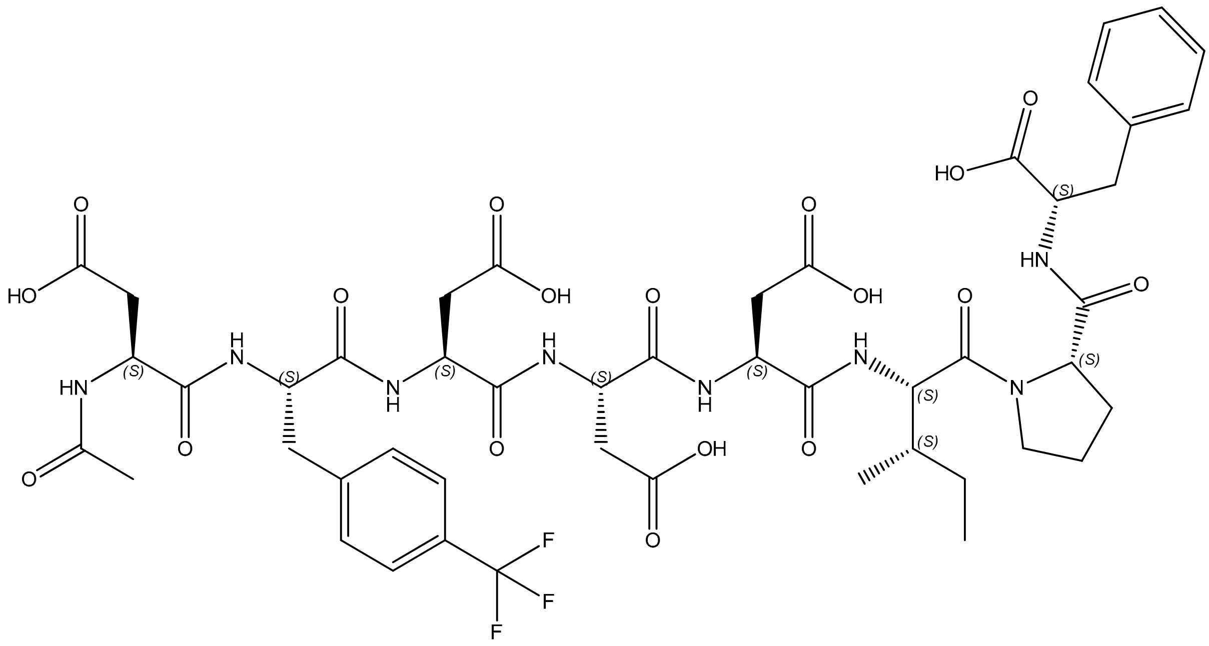

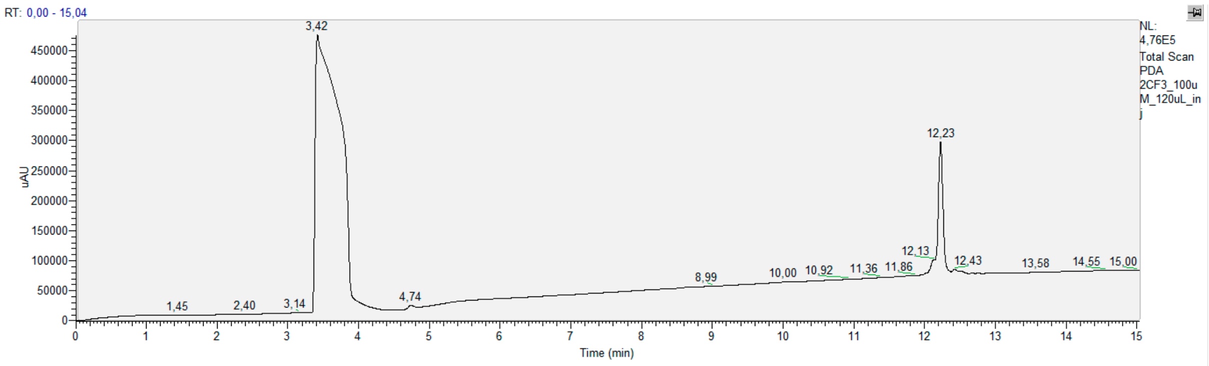

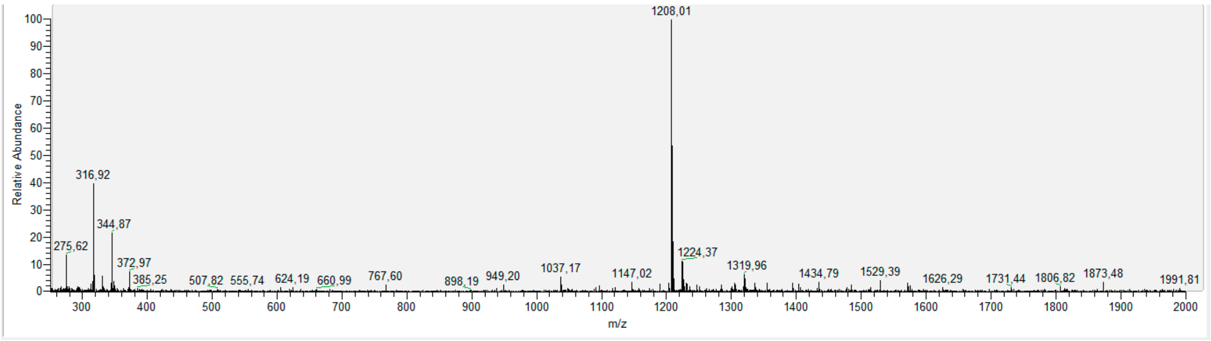

Compound 17 – β[3]-Asp3

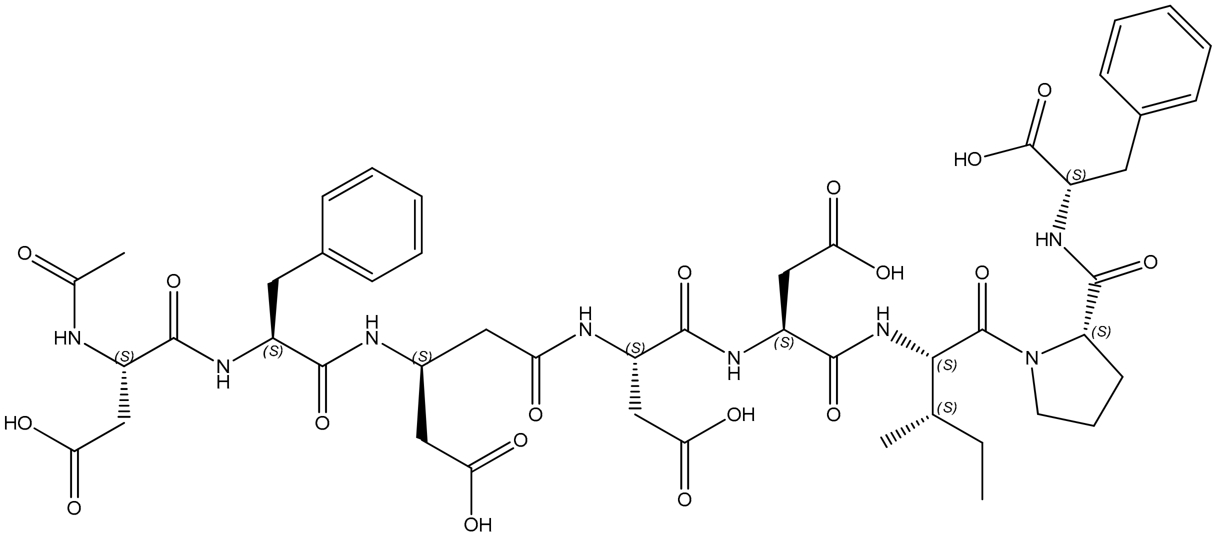

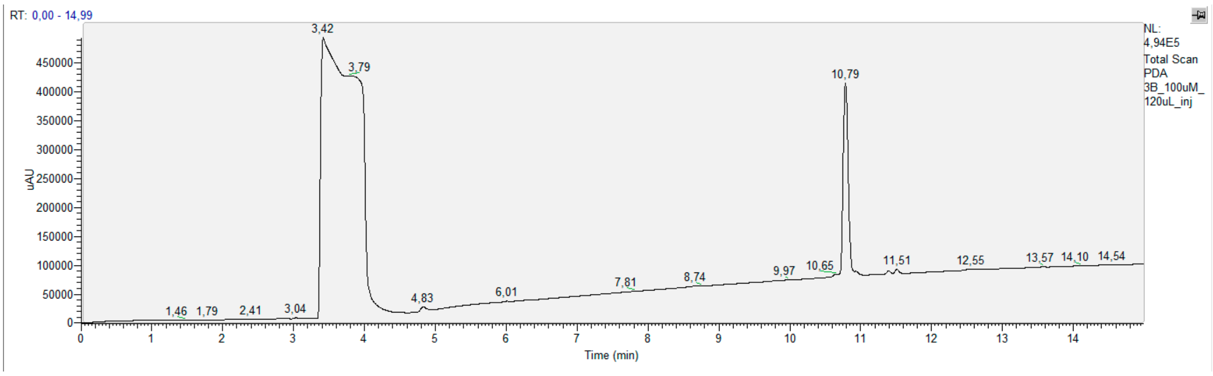

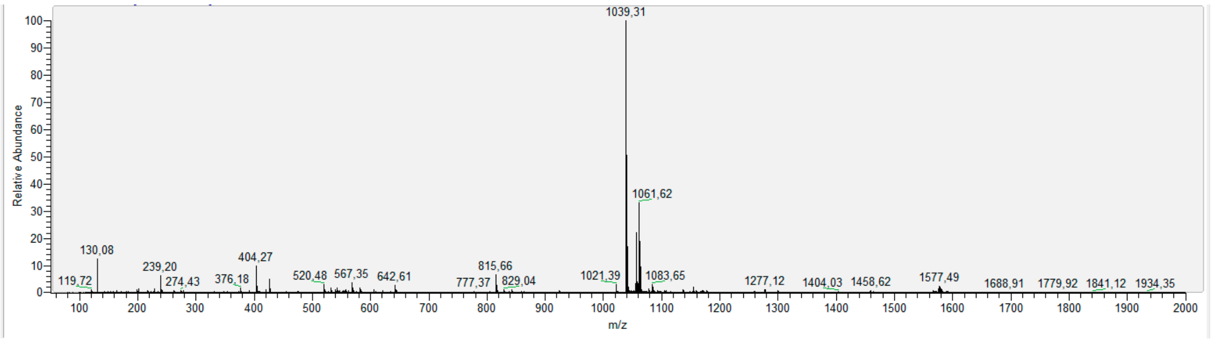

Compound 18 – D-Asp3

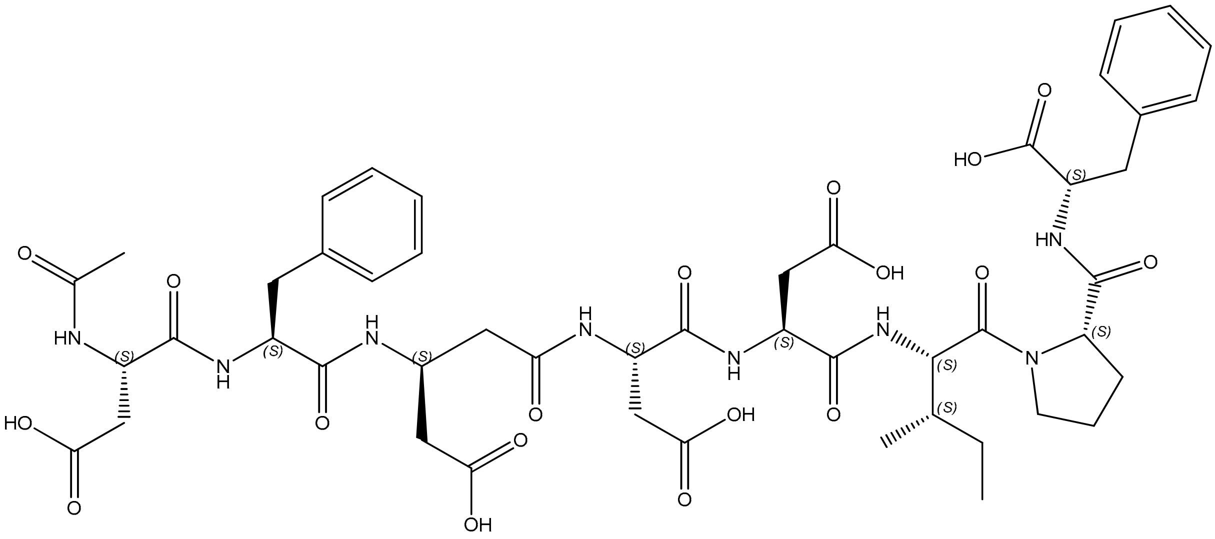

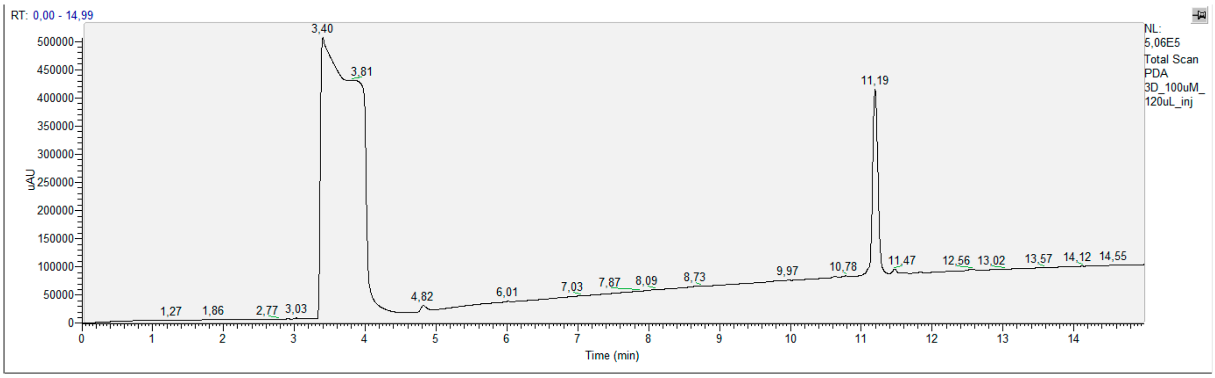

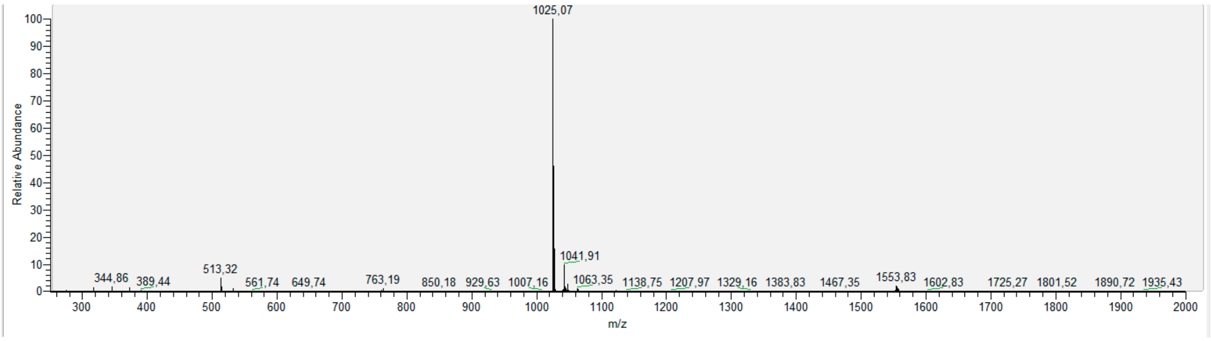

Compound 19 – Glu3

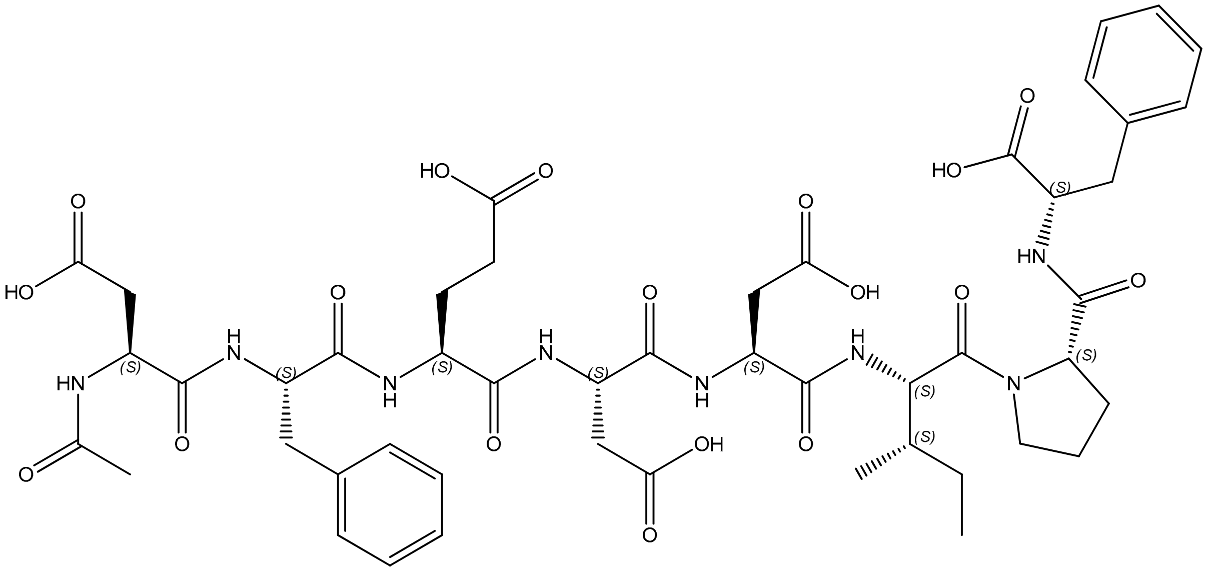

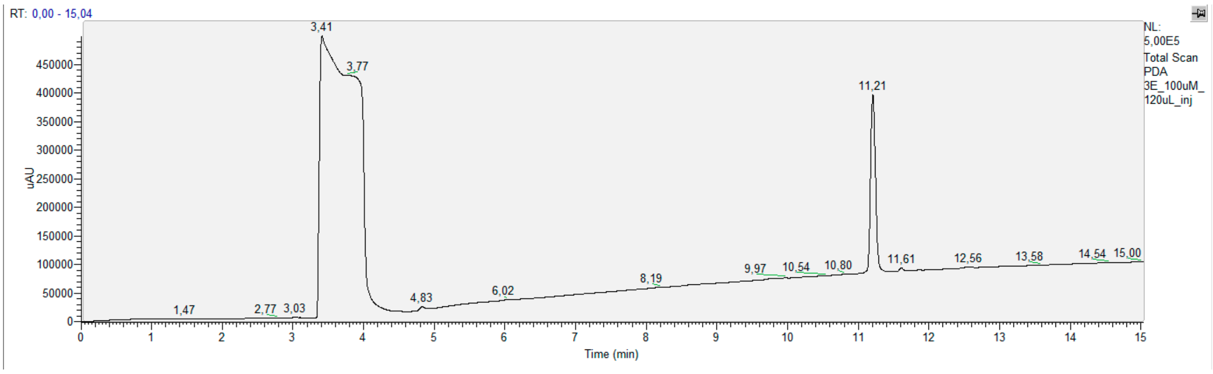

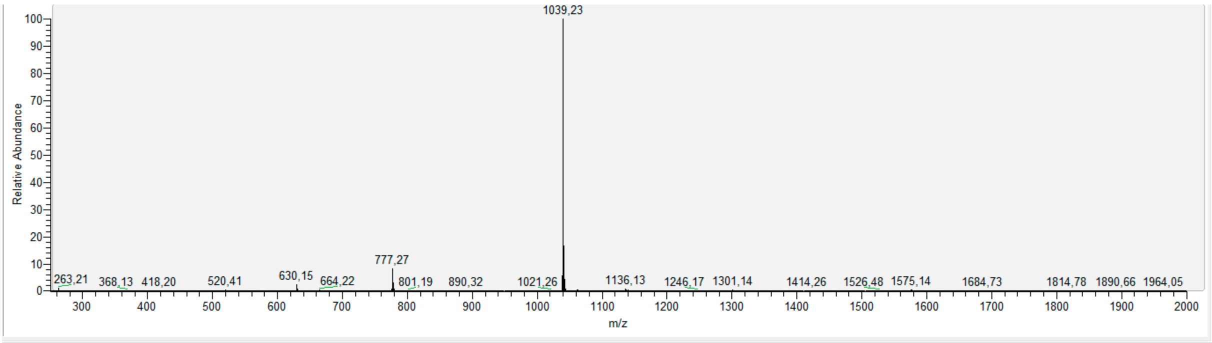

Compound 20 – Asn3

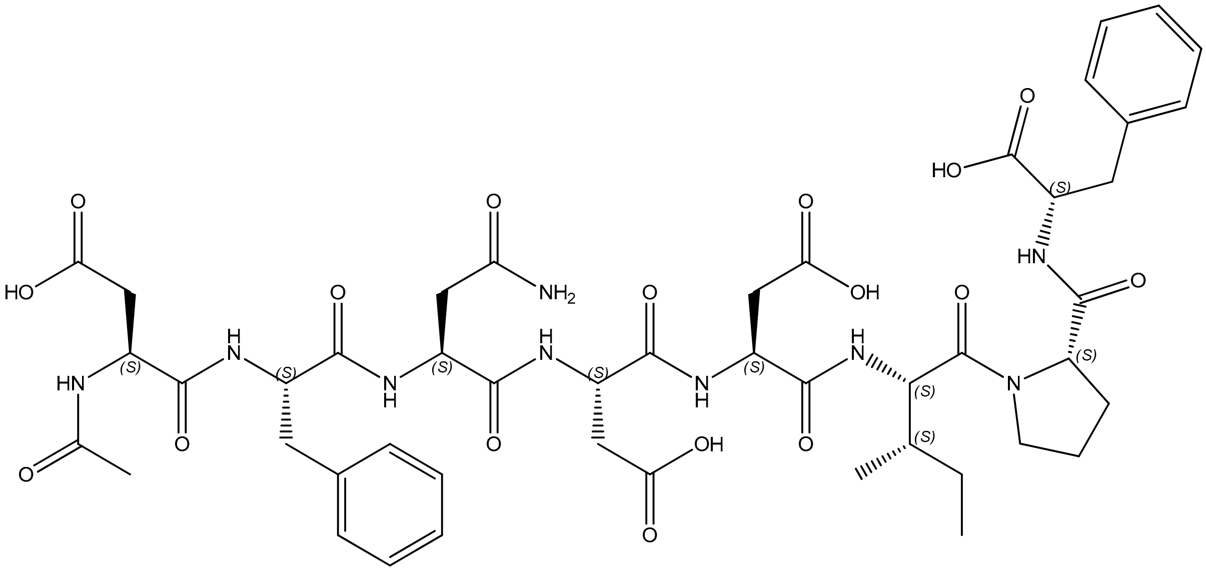

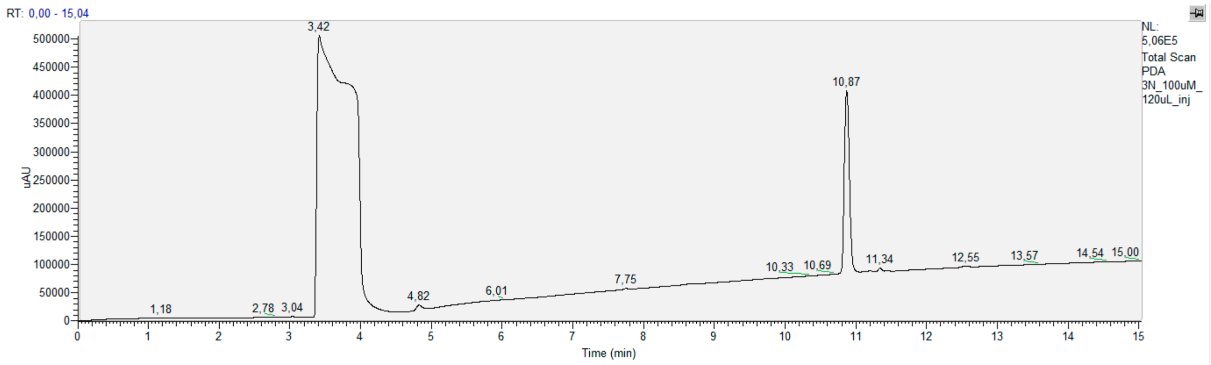

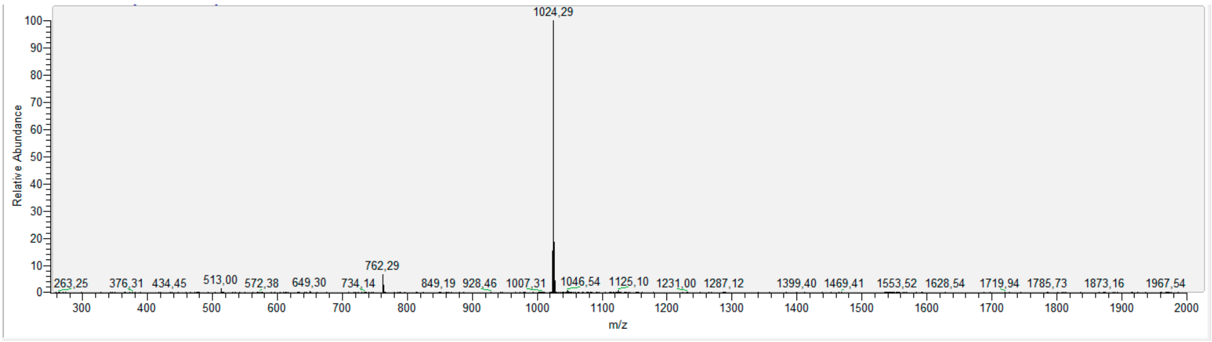

Compound 21 – NM-Asp3

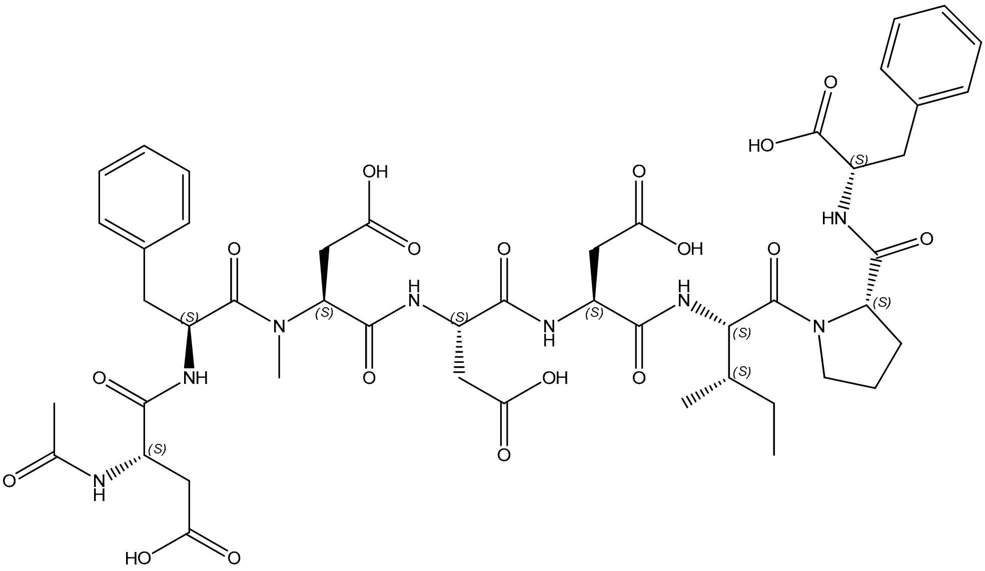

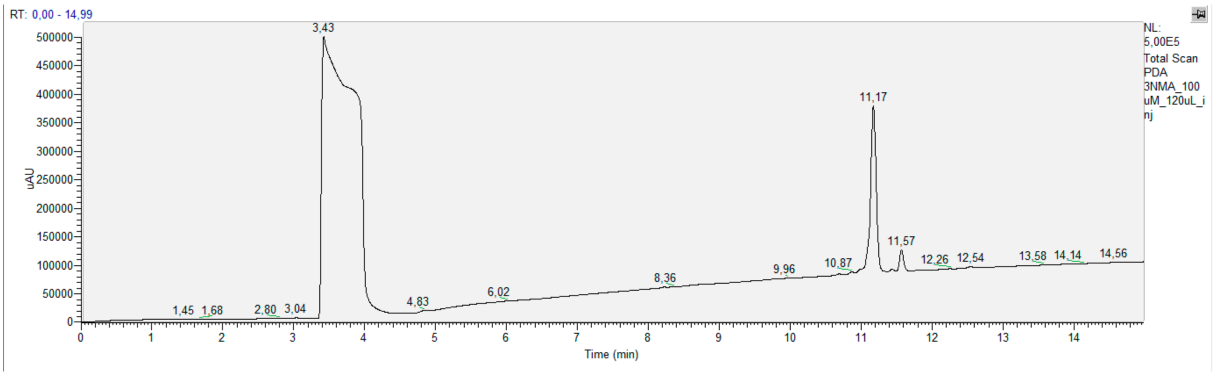

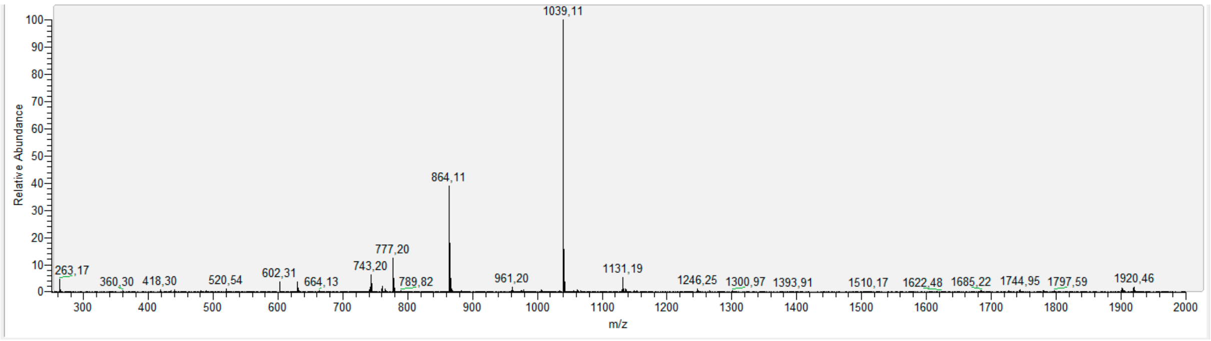

Compound 22 – β^{3}-Asp4

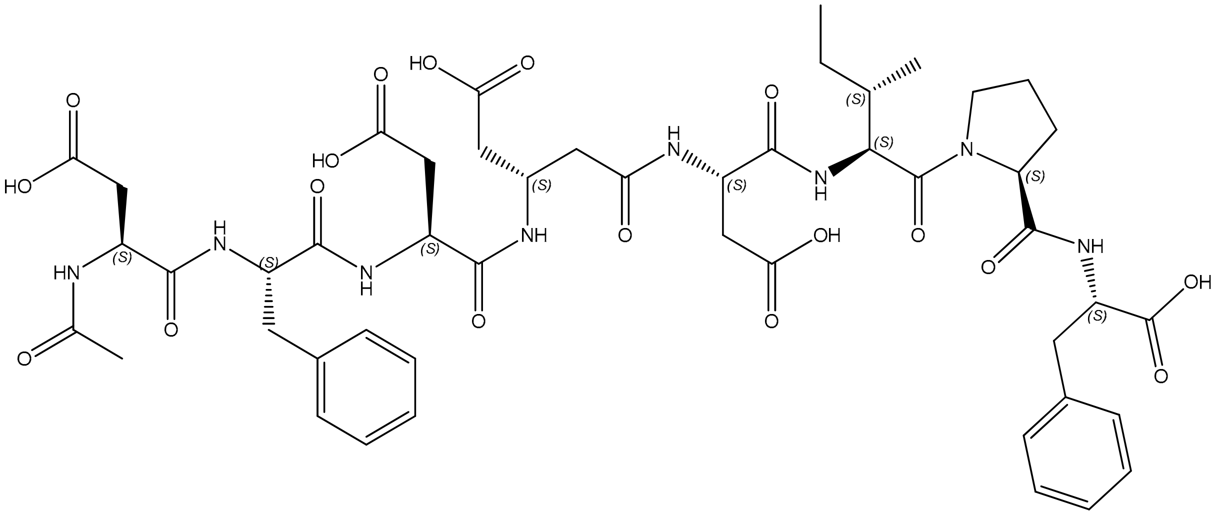

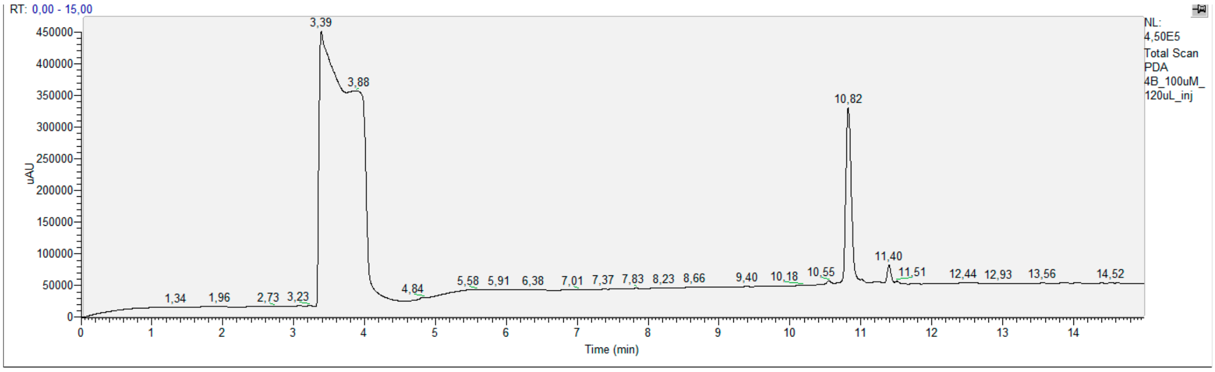

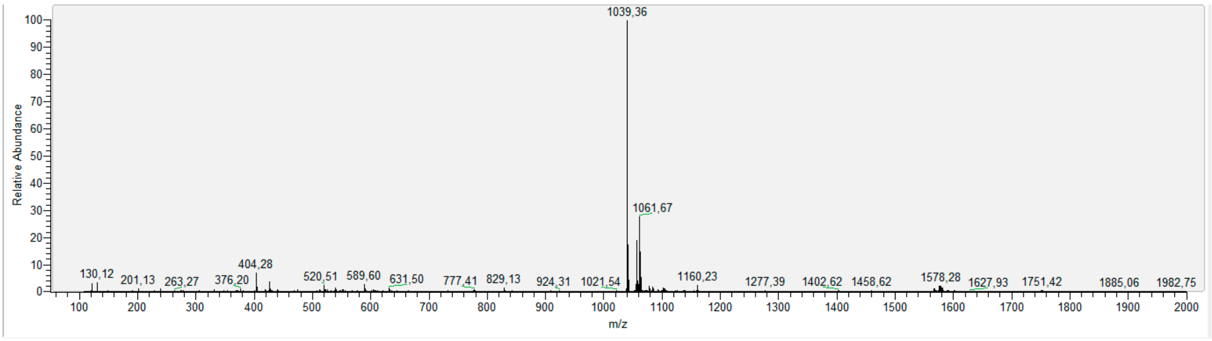

Compound 23 – D-Asp4

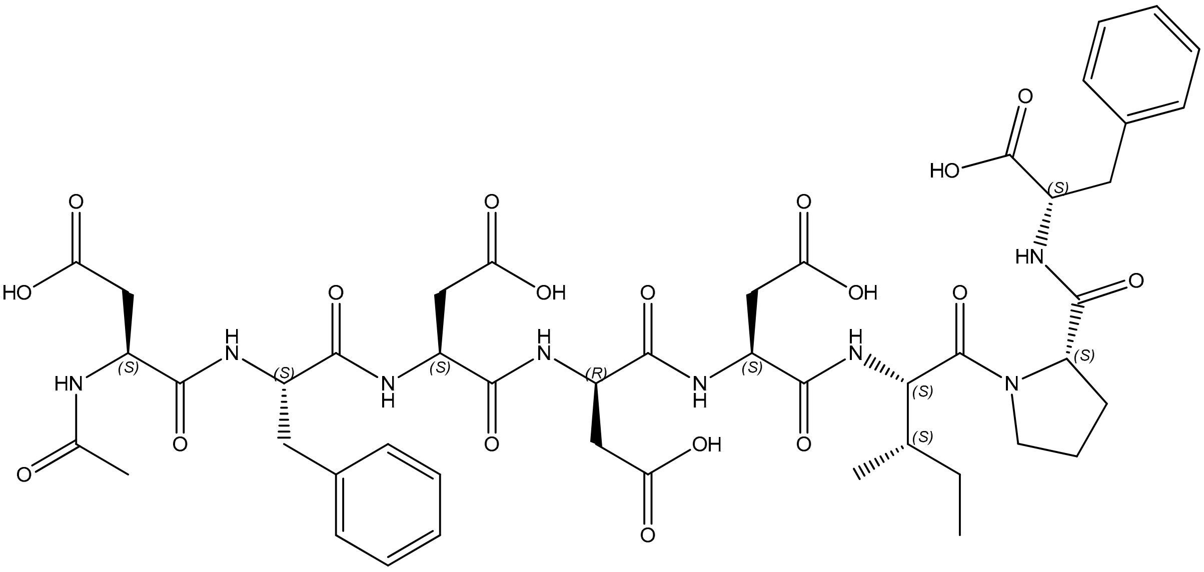

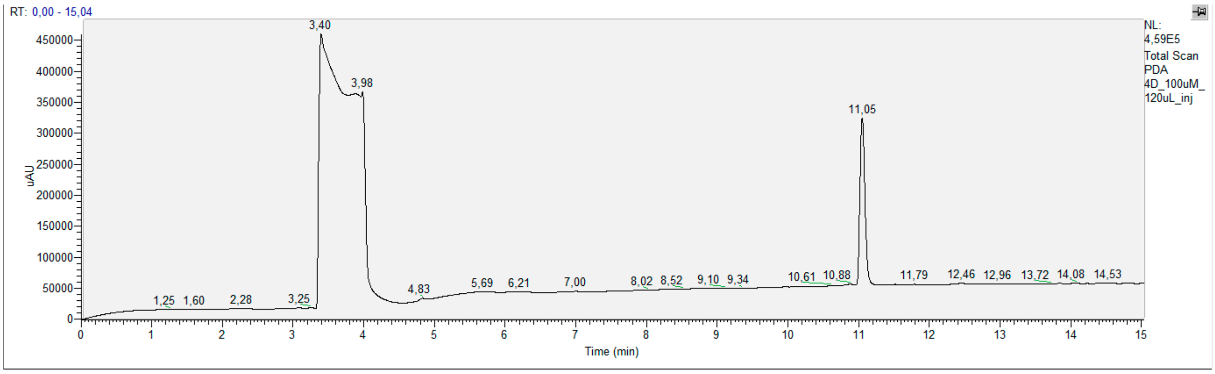

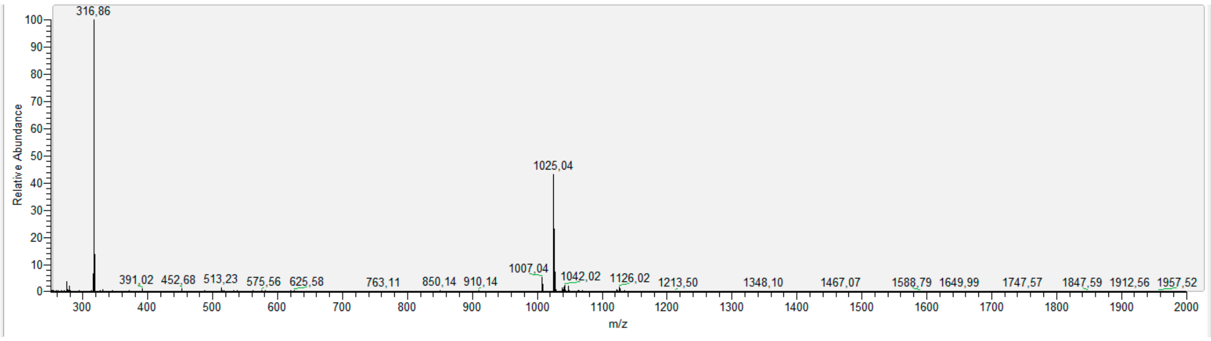

Compound 24 – Glu4

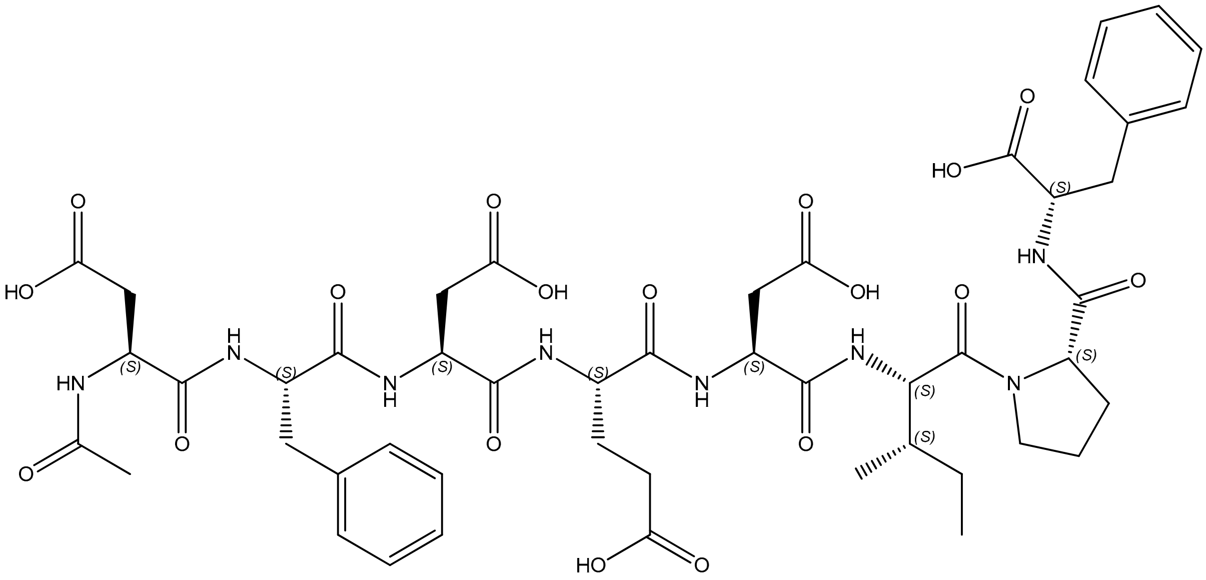

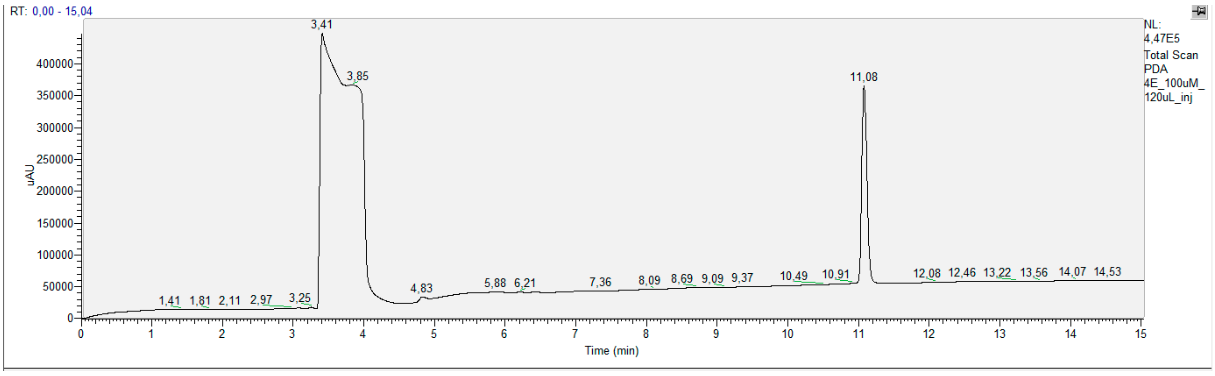

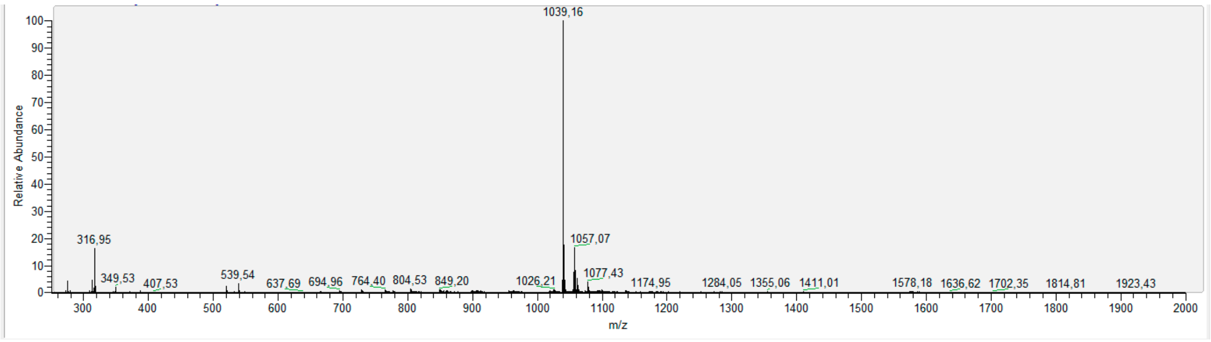

Compound 25 – Asn4

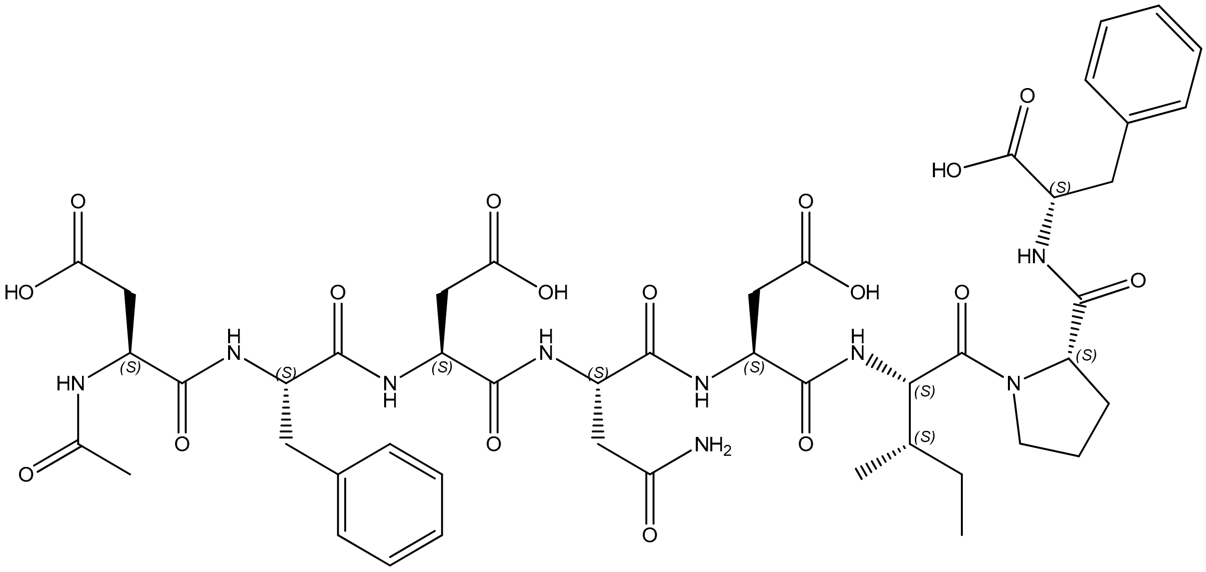

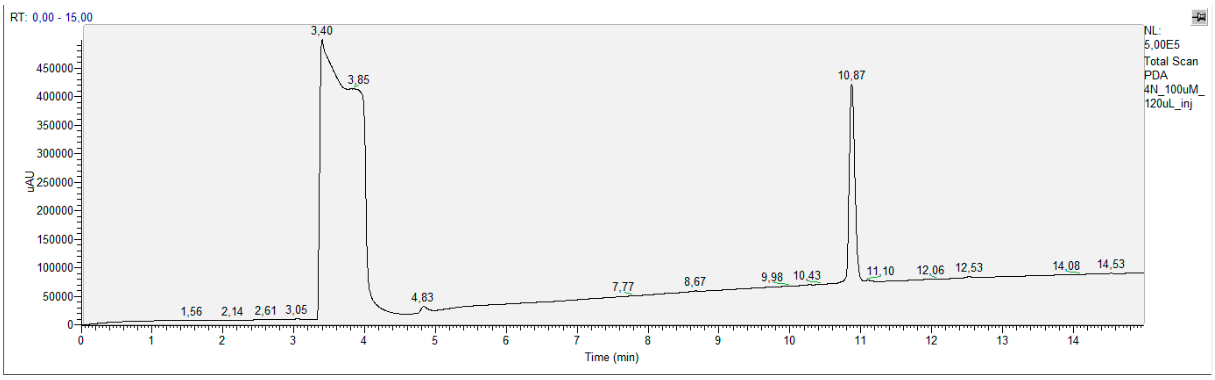

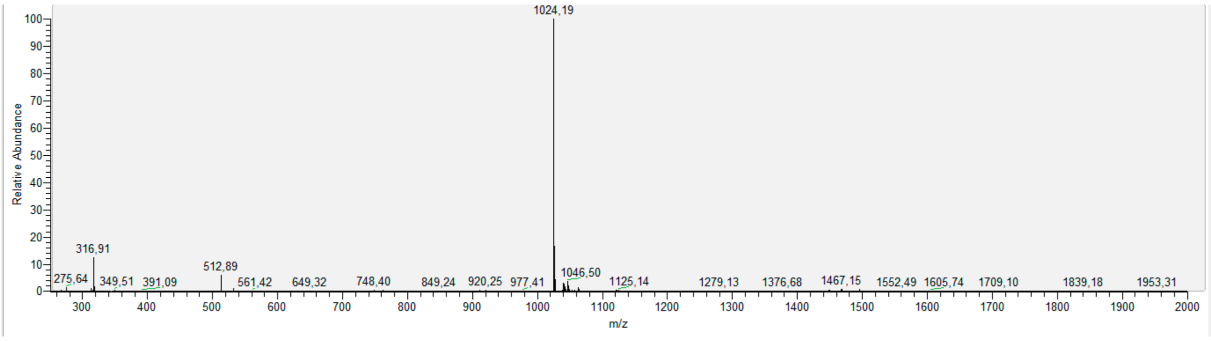

Compound 26 – NM-Asp4

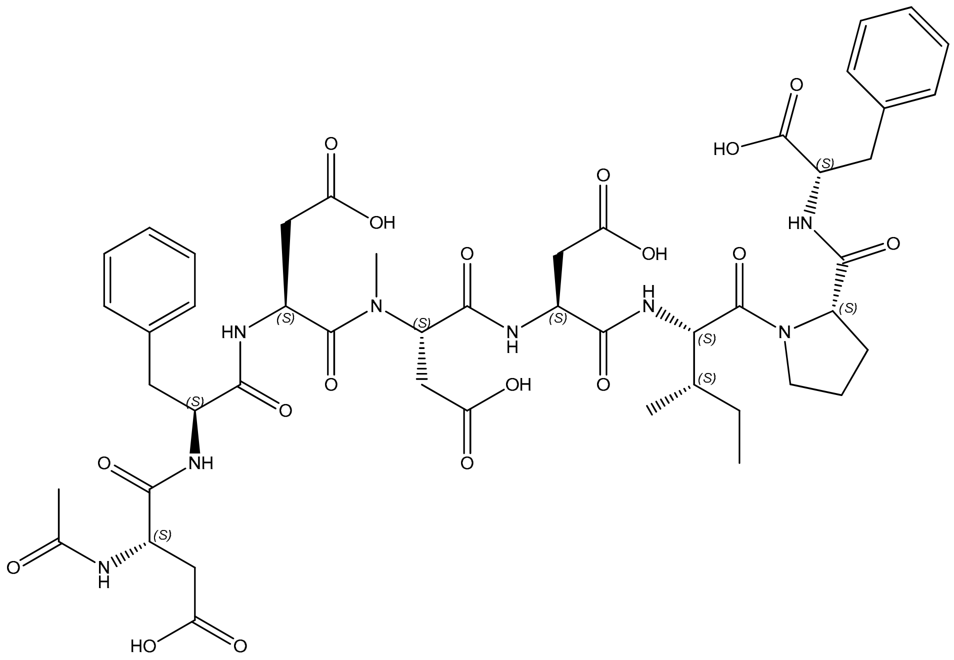

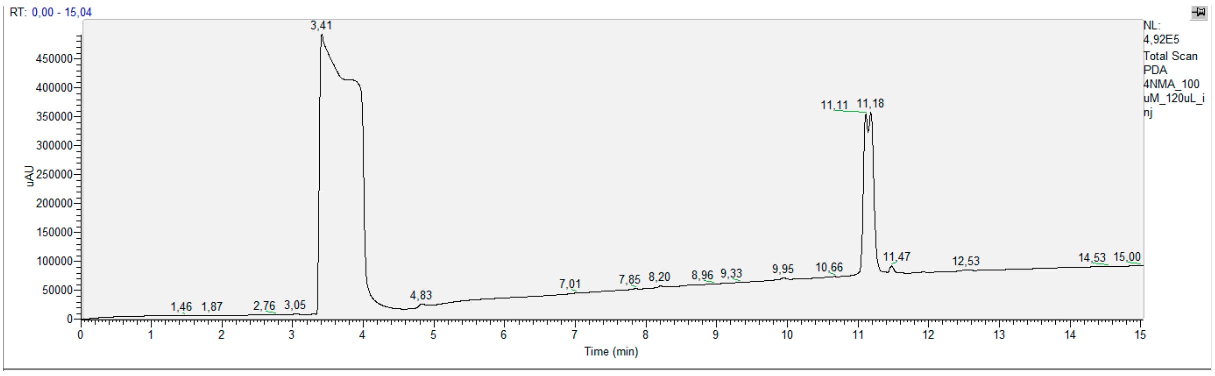

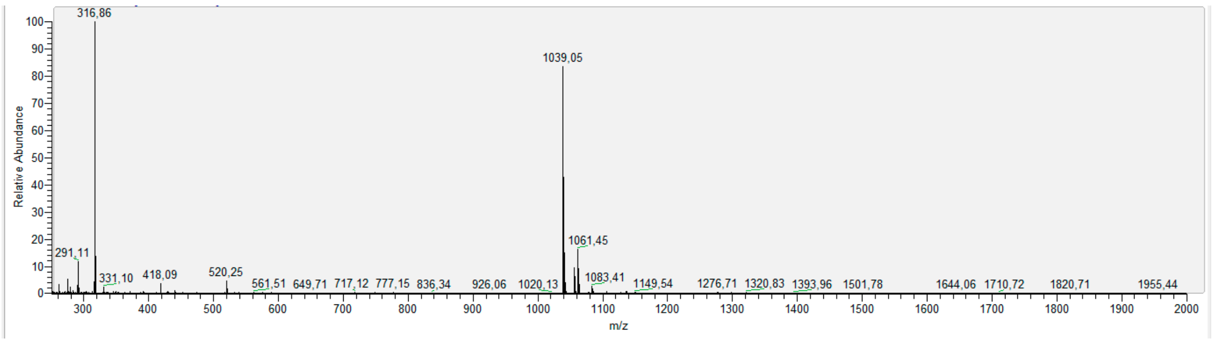

Compound 27 – β³-Asp5

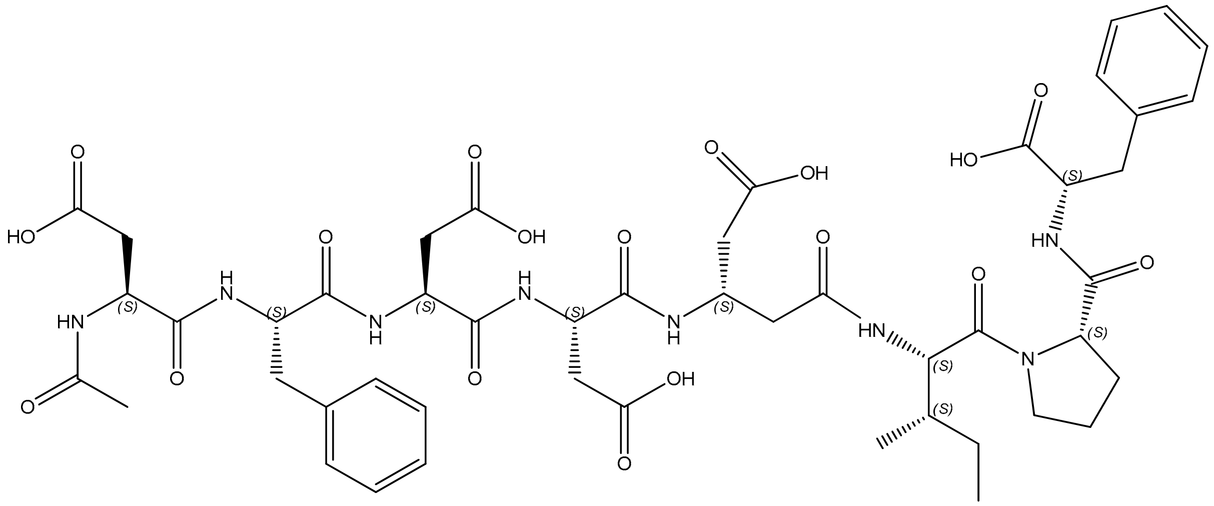

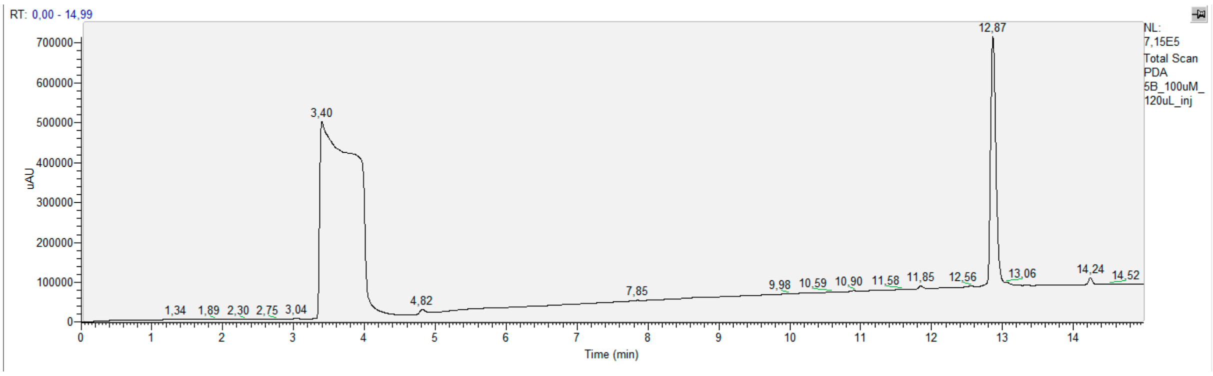

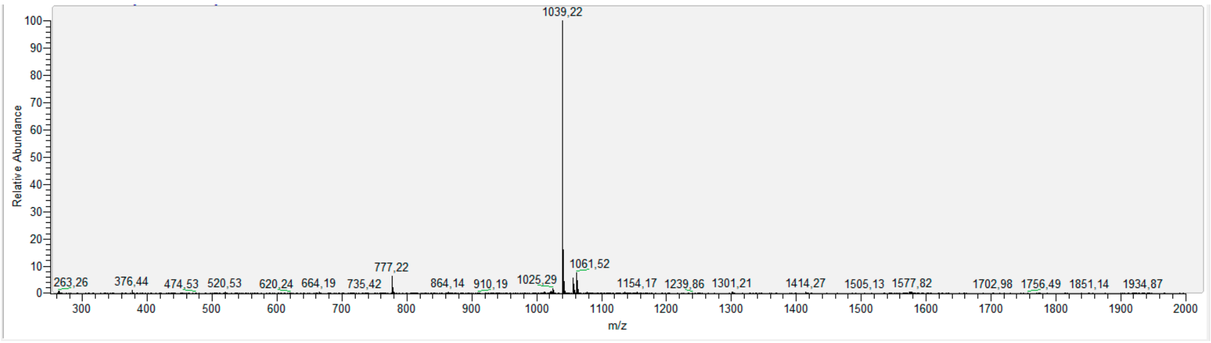

Compound 28 – D-Asp5

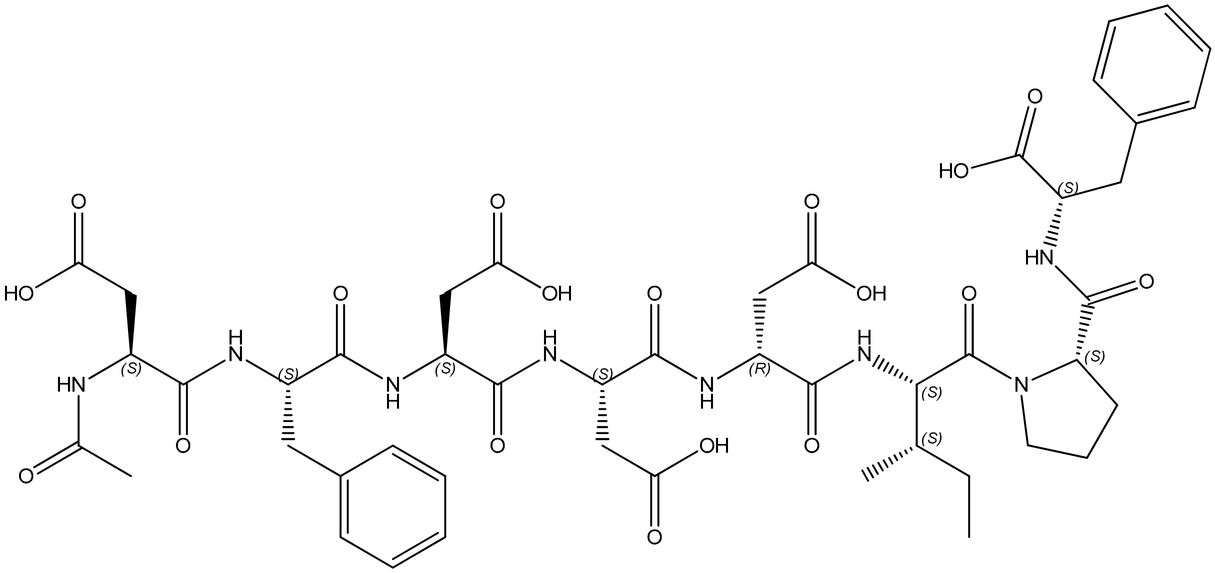

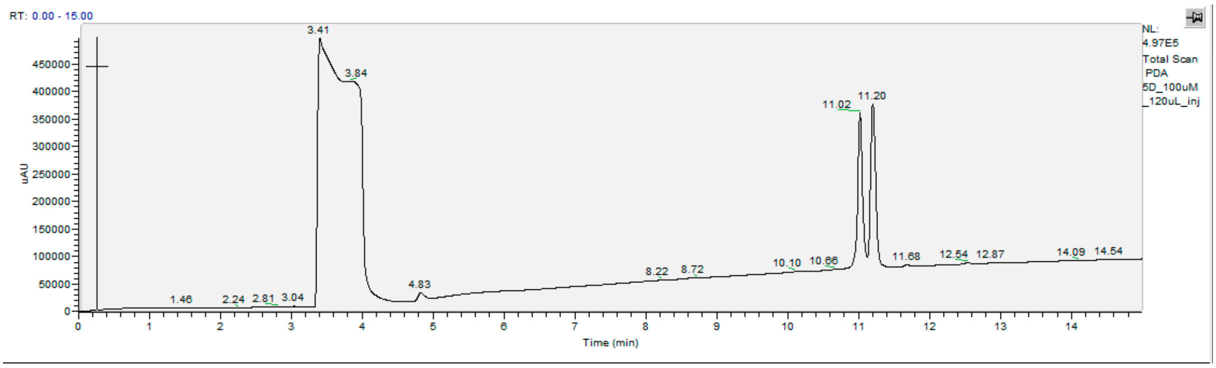

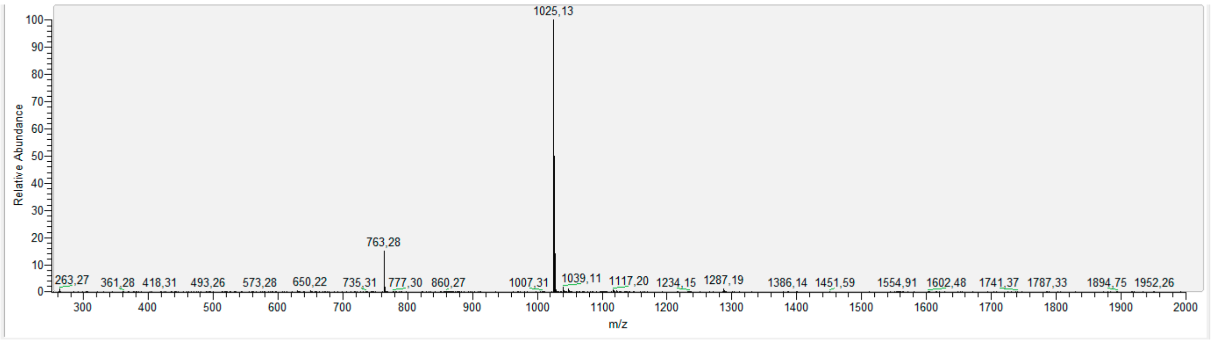

Compound 29 – Glu5

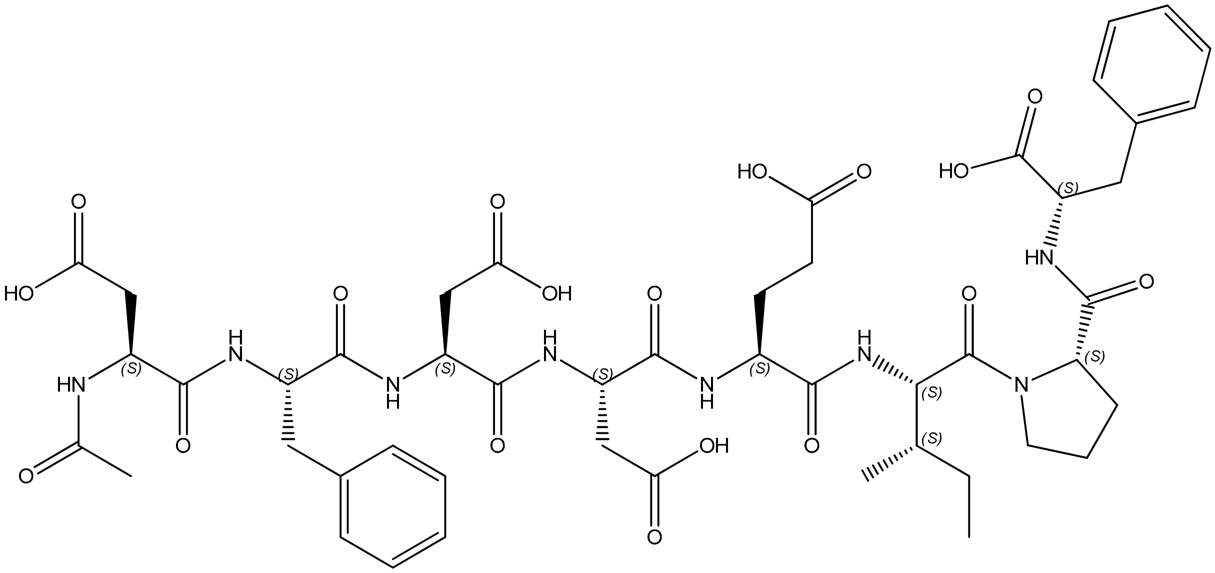

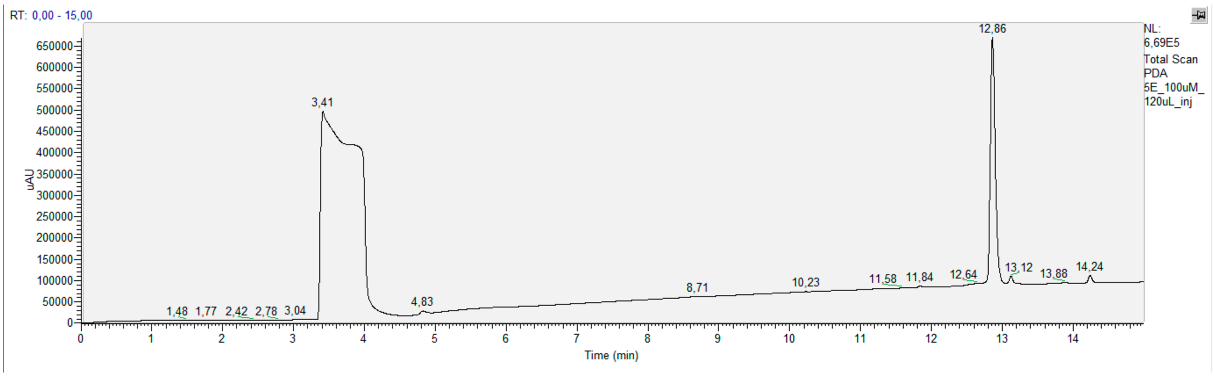

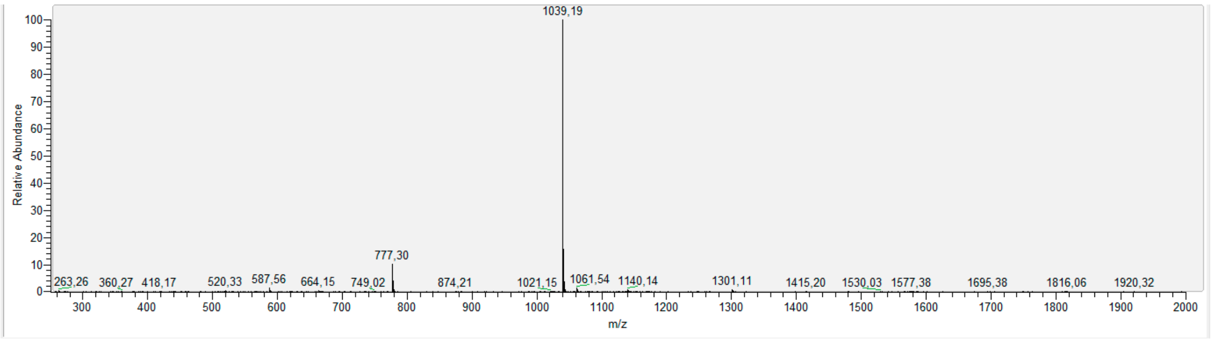

Compound 30 – Asn5

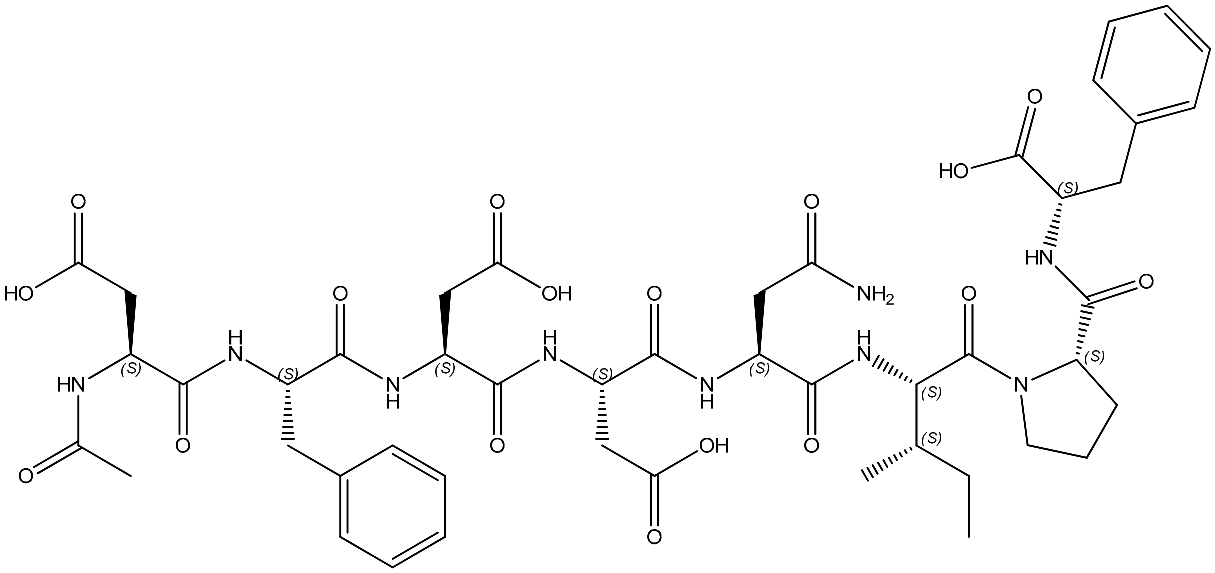

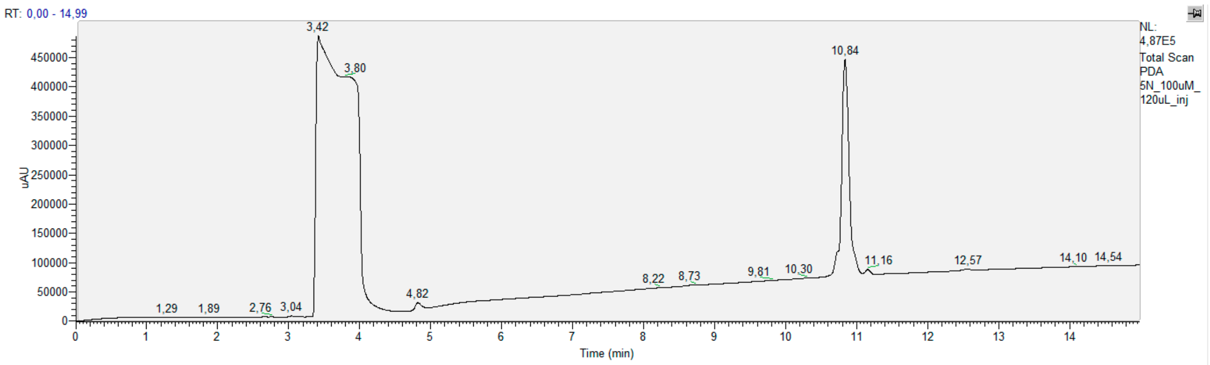

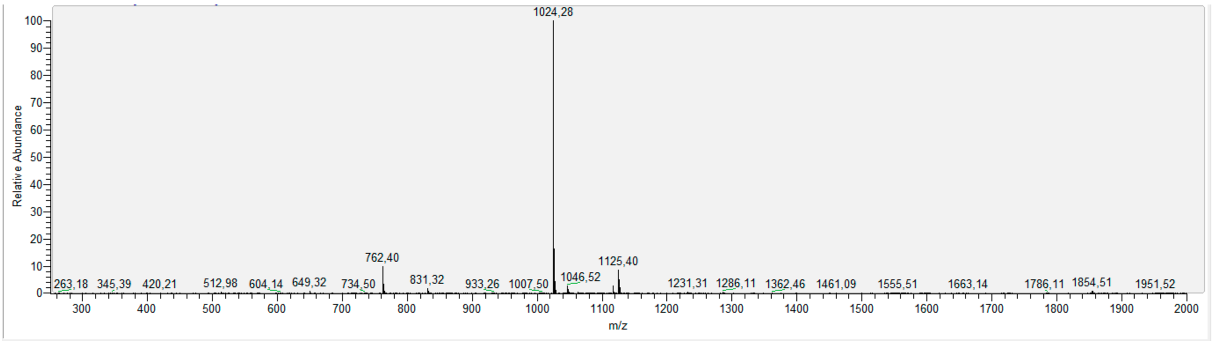

Compound 31 – NM-Asp5

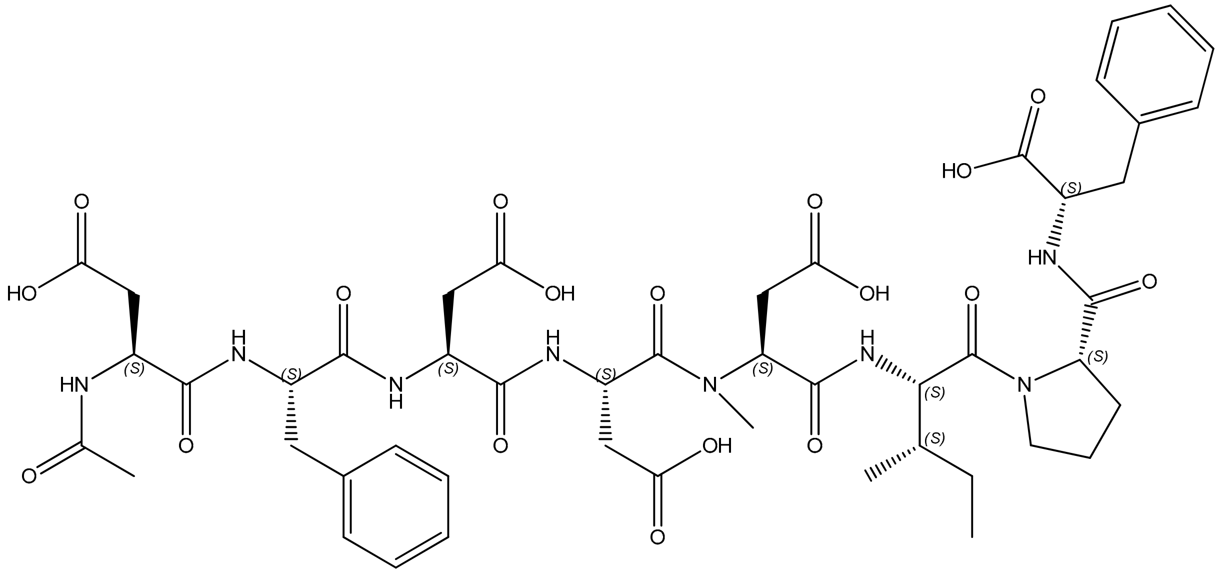

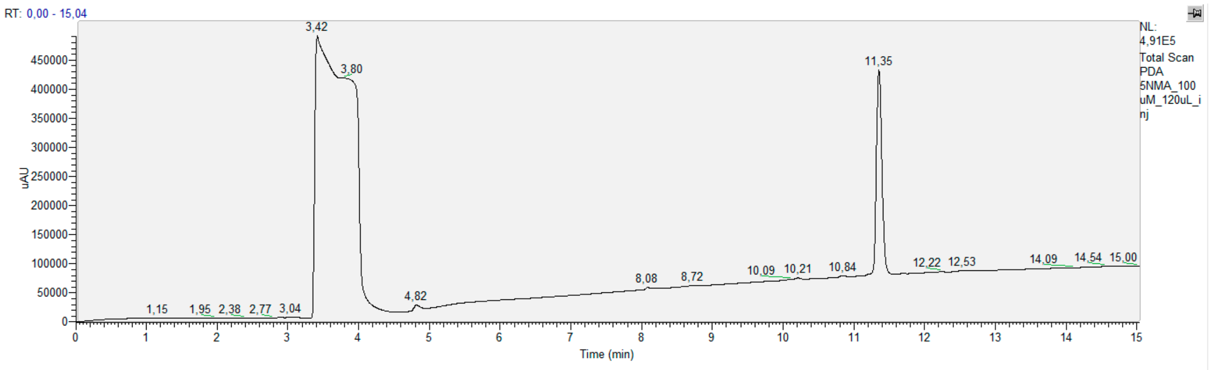

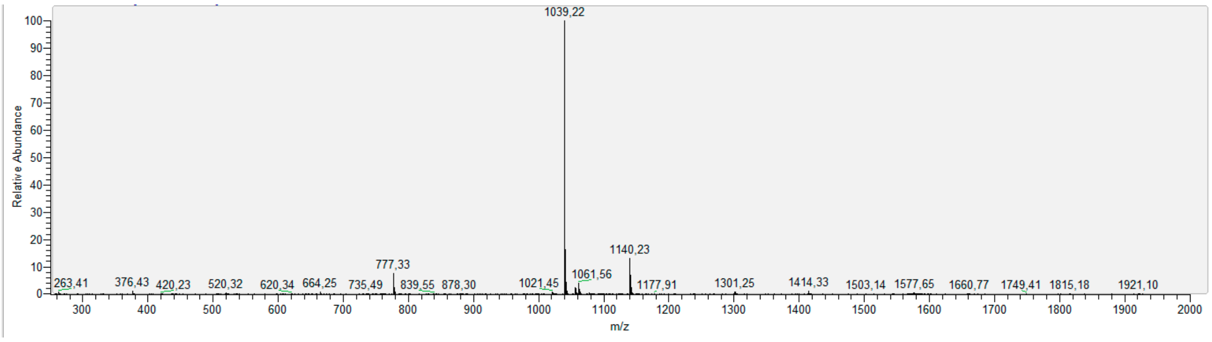

Compound 32 – Aib6

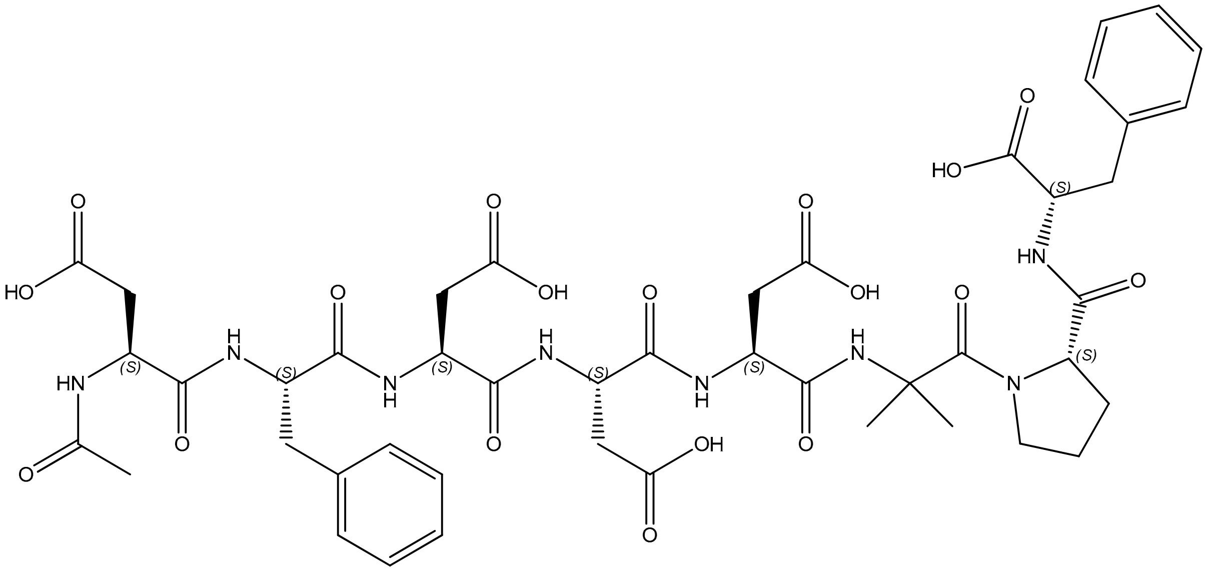

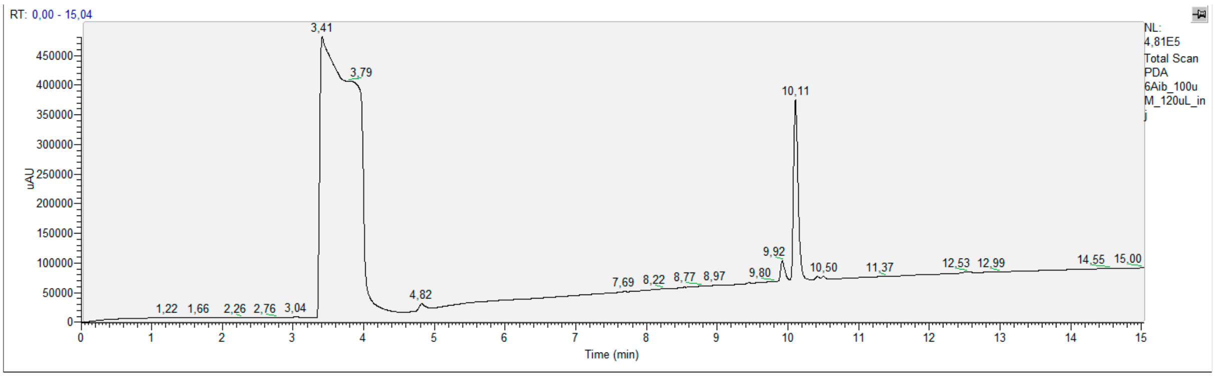

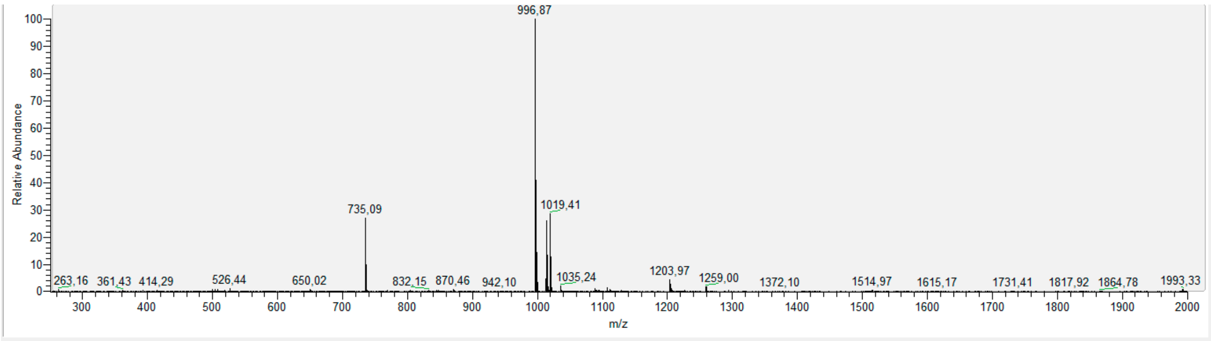

Compound 33 – β^3-Ile6

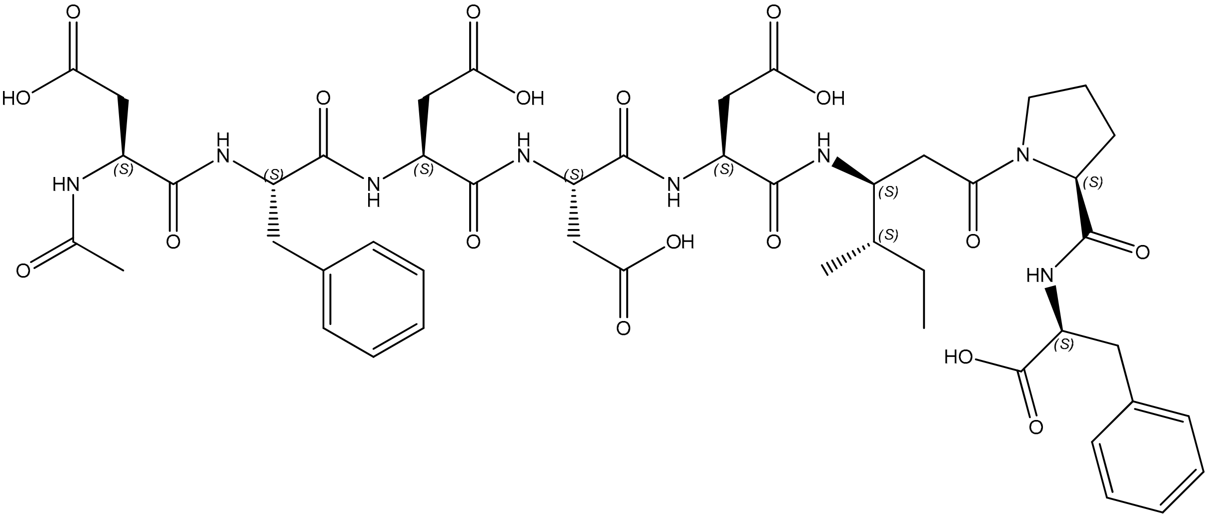

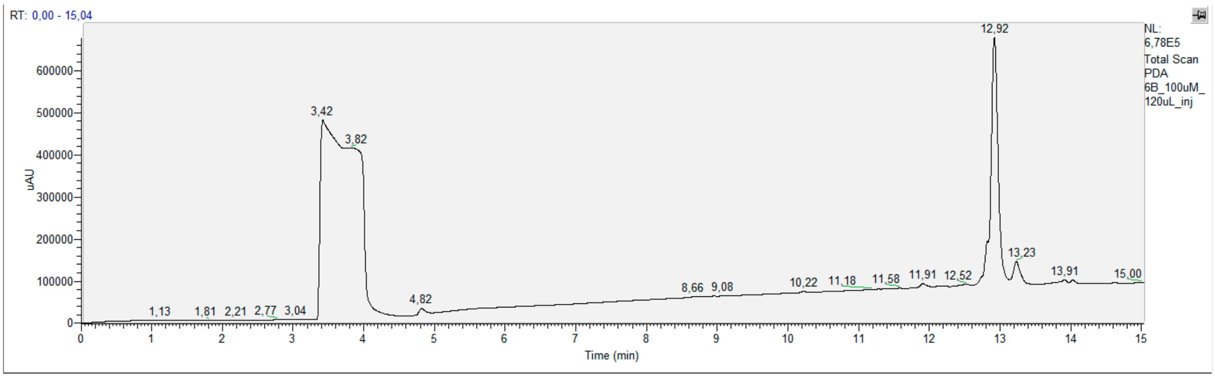

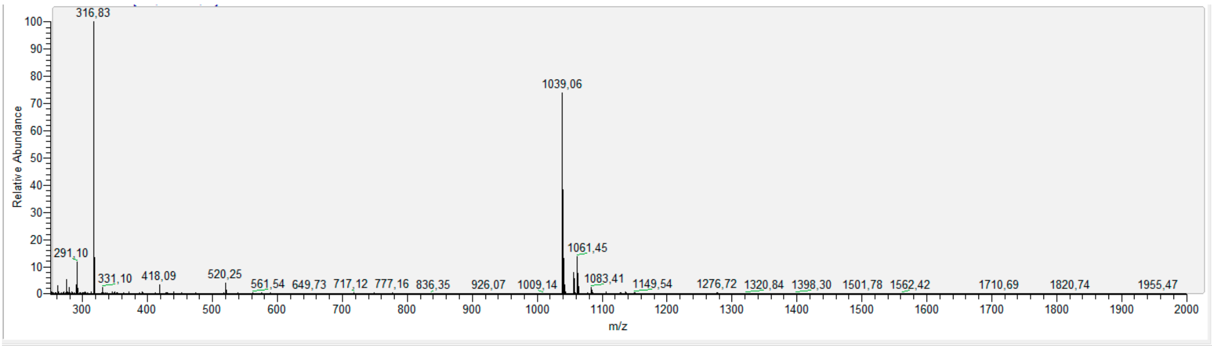

Compound 34 – D-Ile6

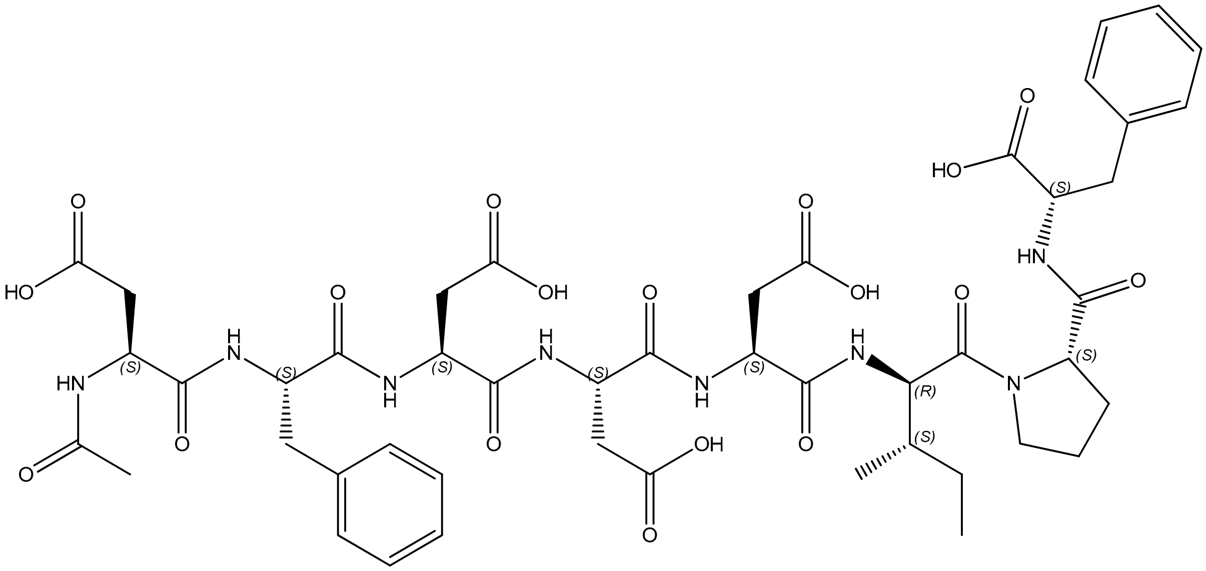

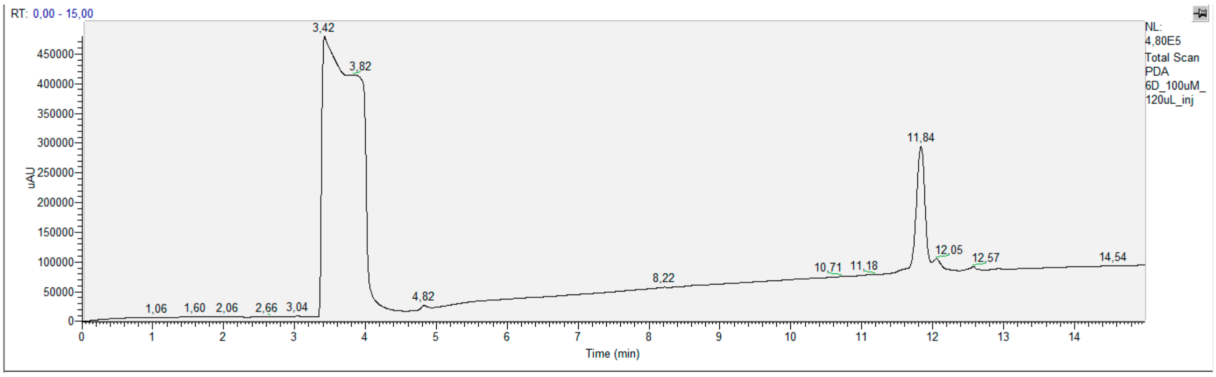

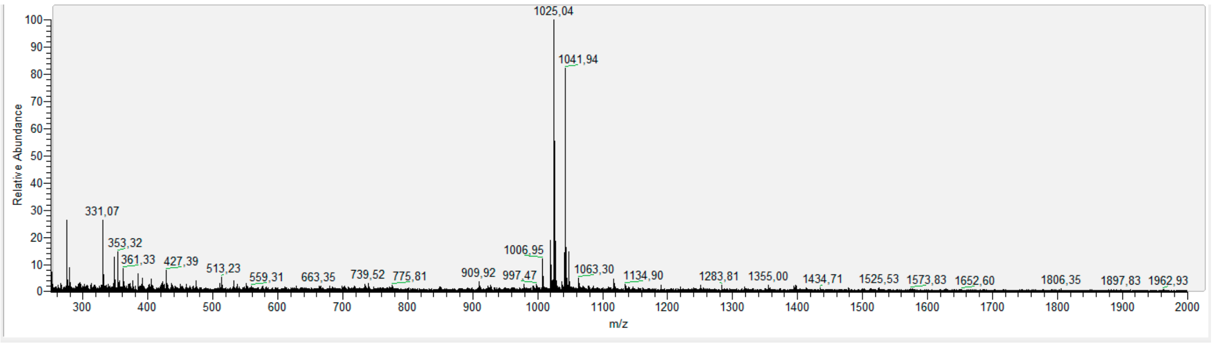

Compound 35 – Leu6

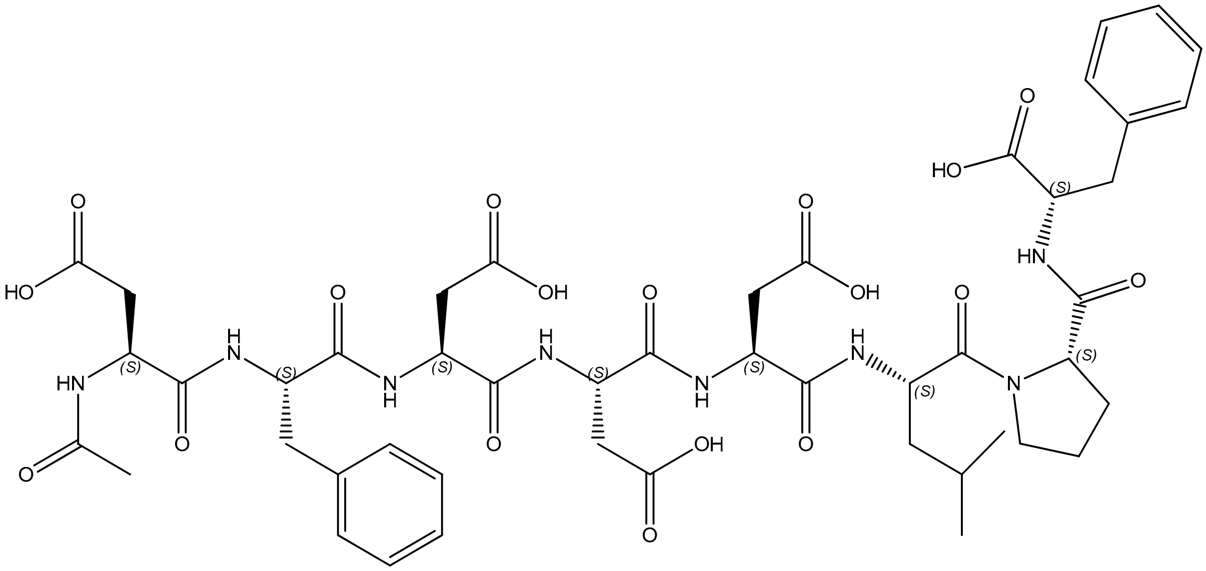

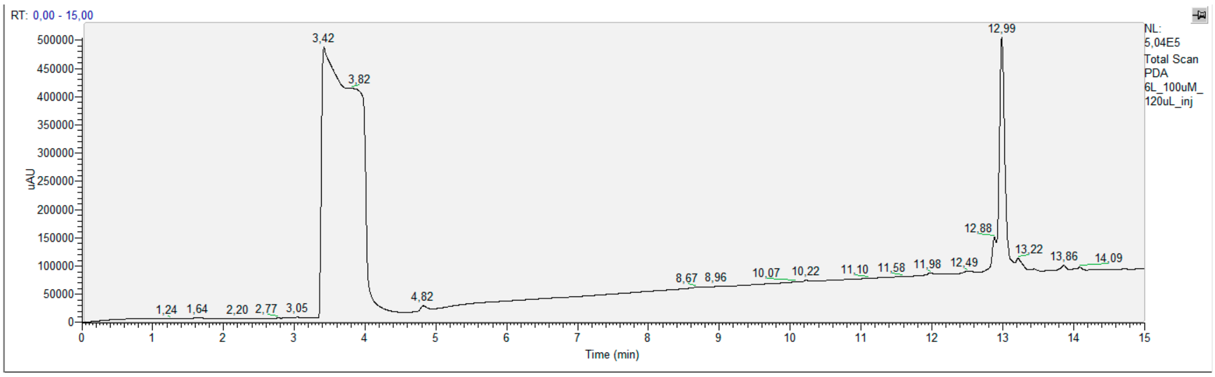

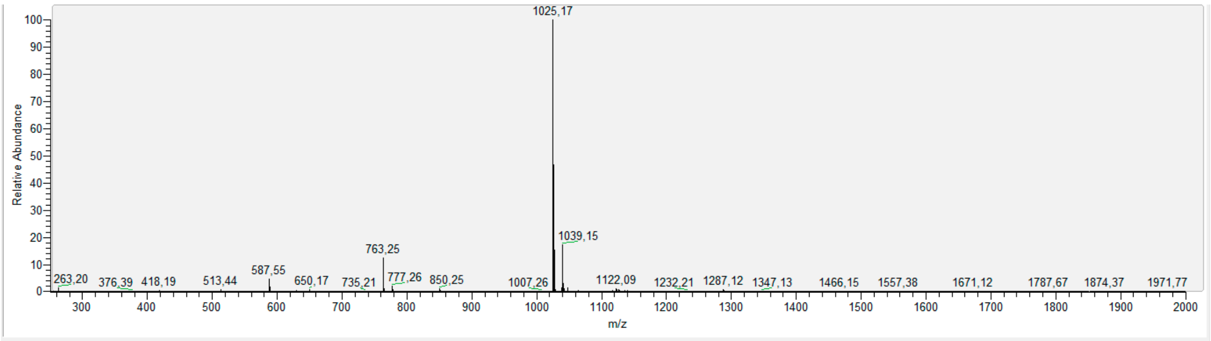

Compound 36 – NM-Ile6

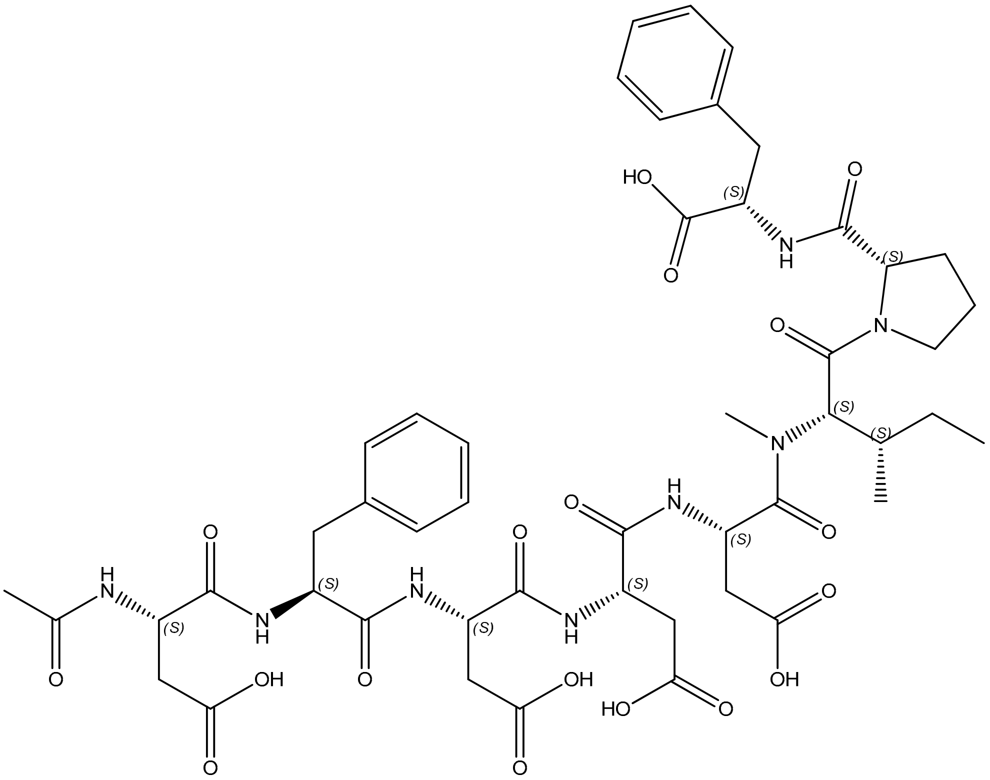

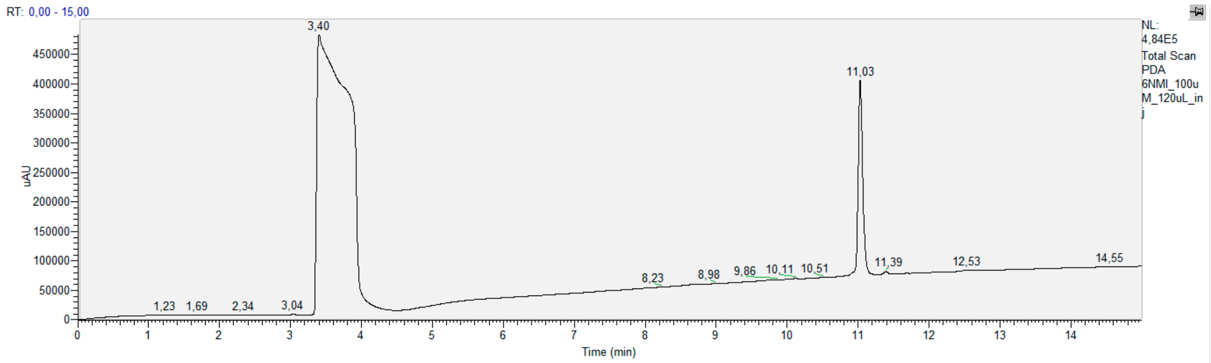

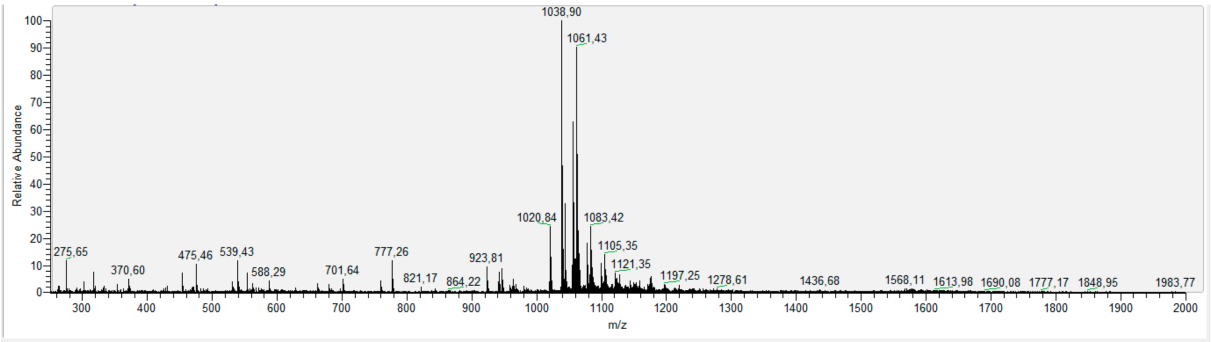

Compound 37 – nLeu6

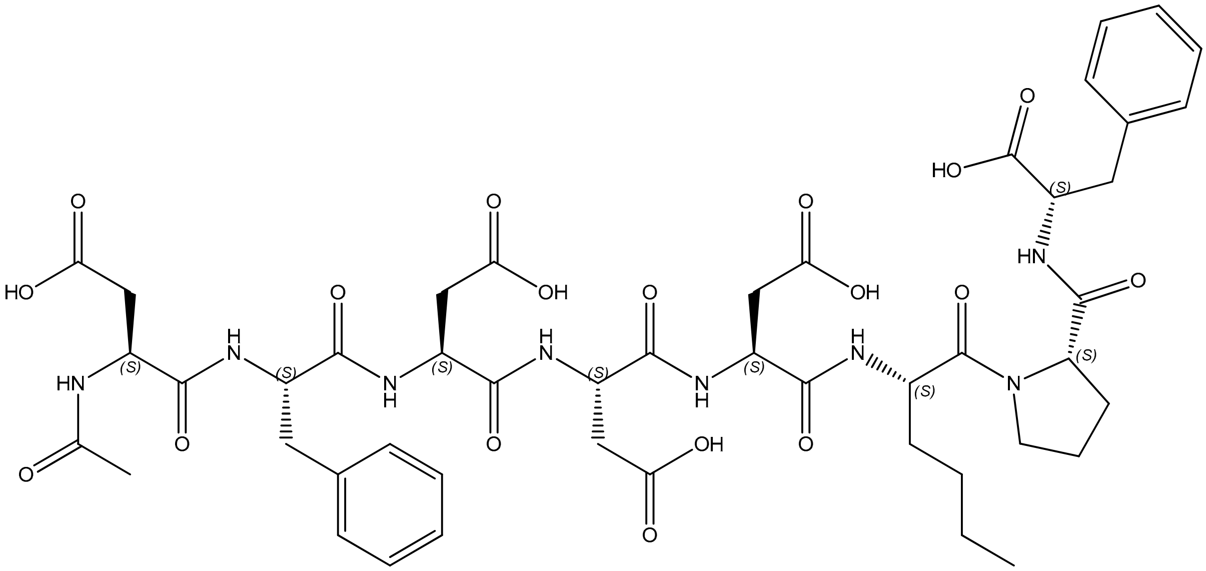

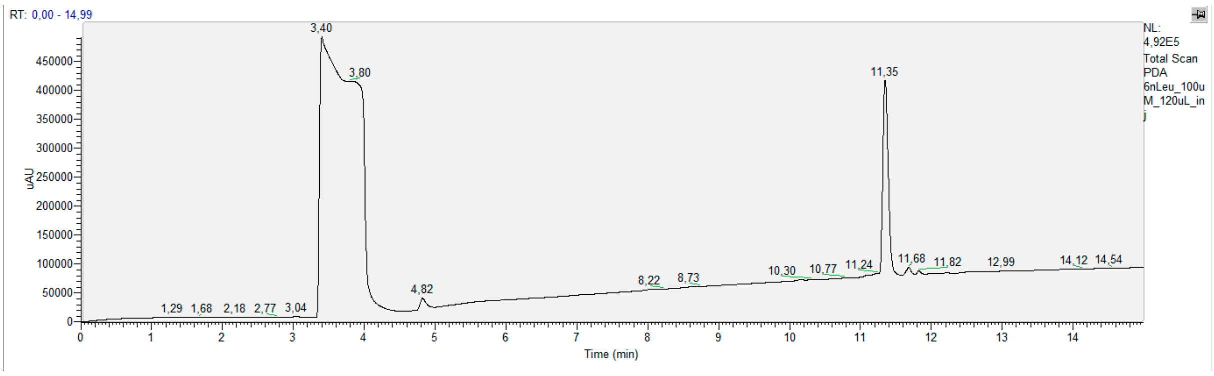

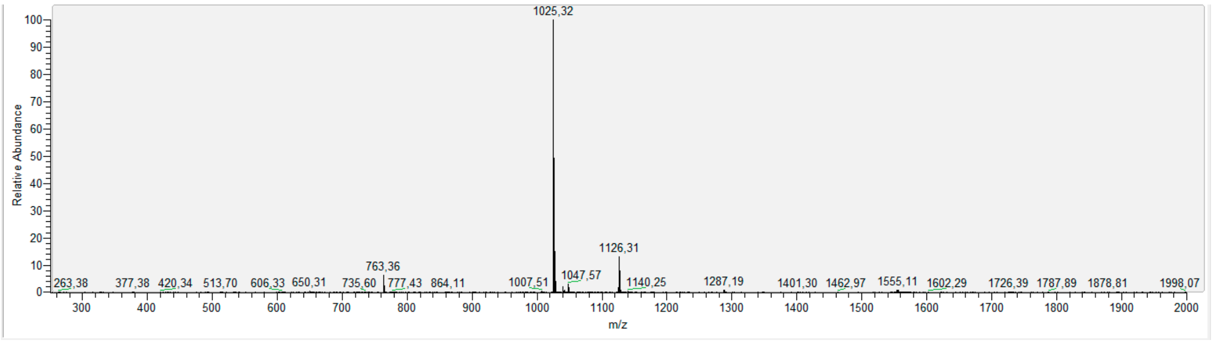

Compound 38 – tBuGly6

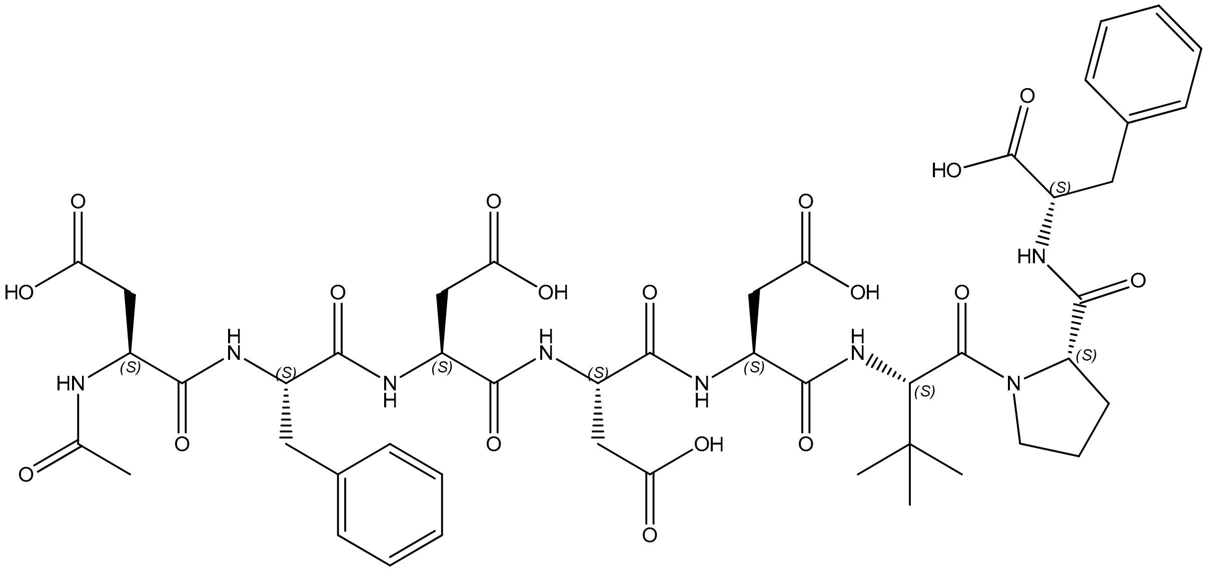

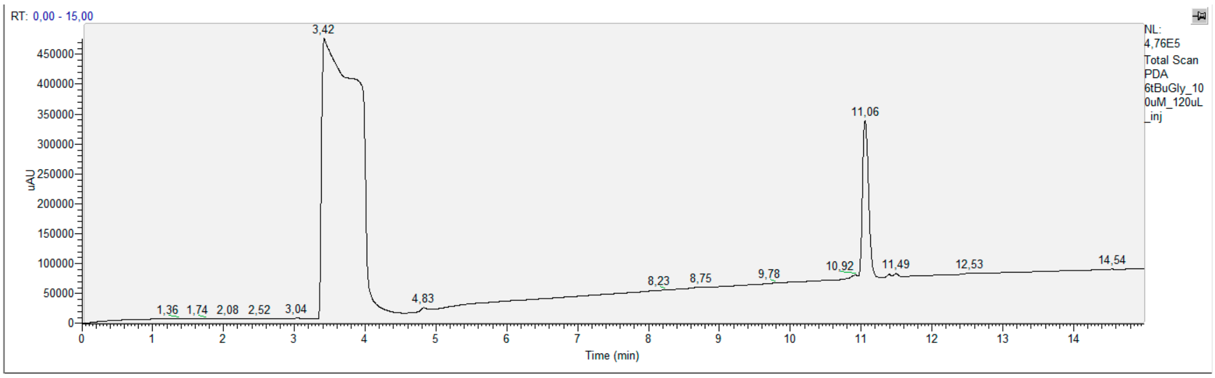

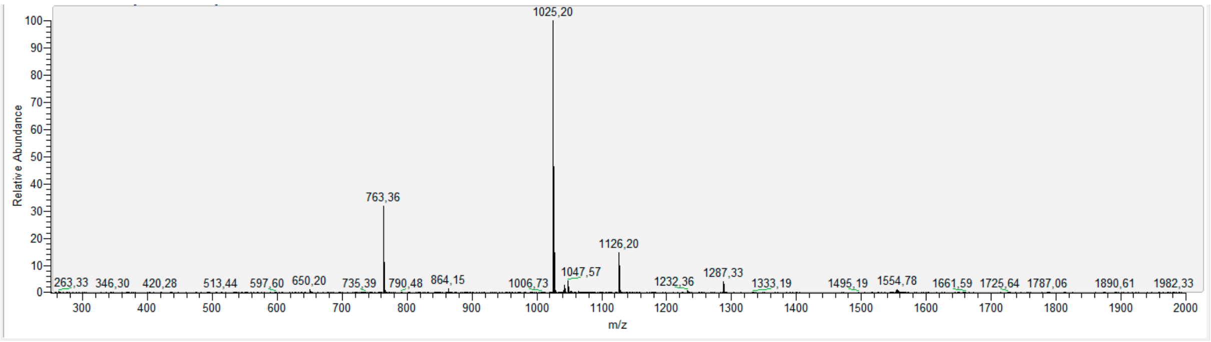

Compound 39 – 4cF-Pro7

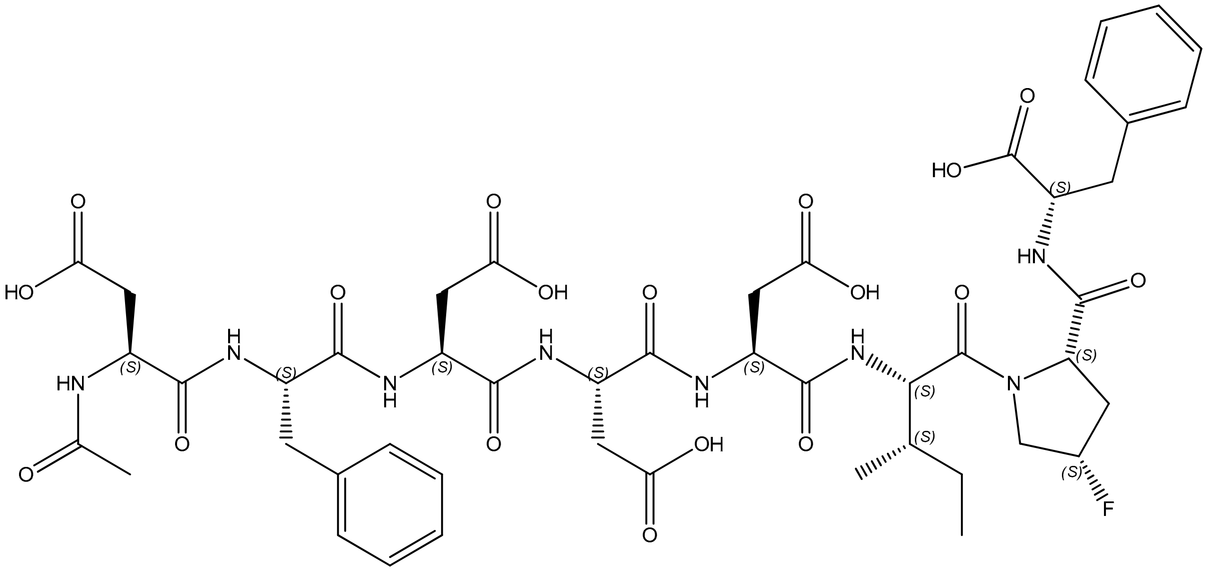

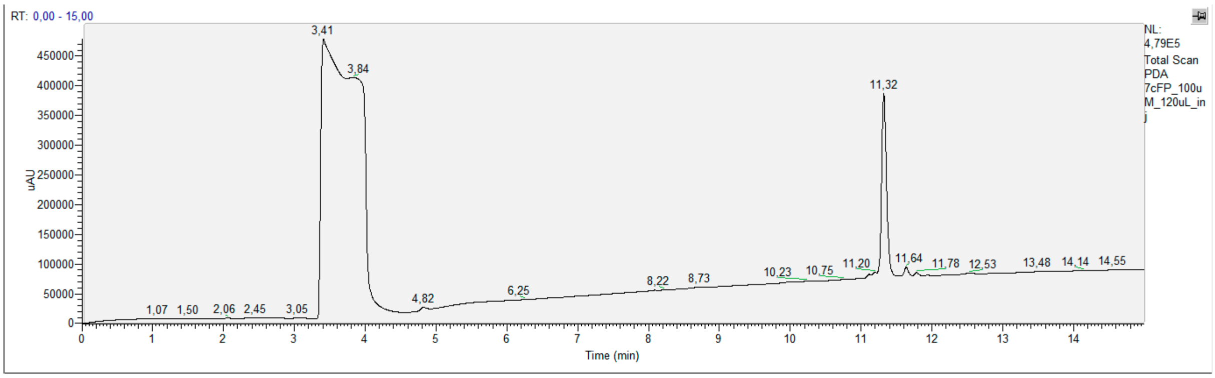

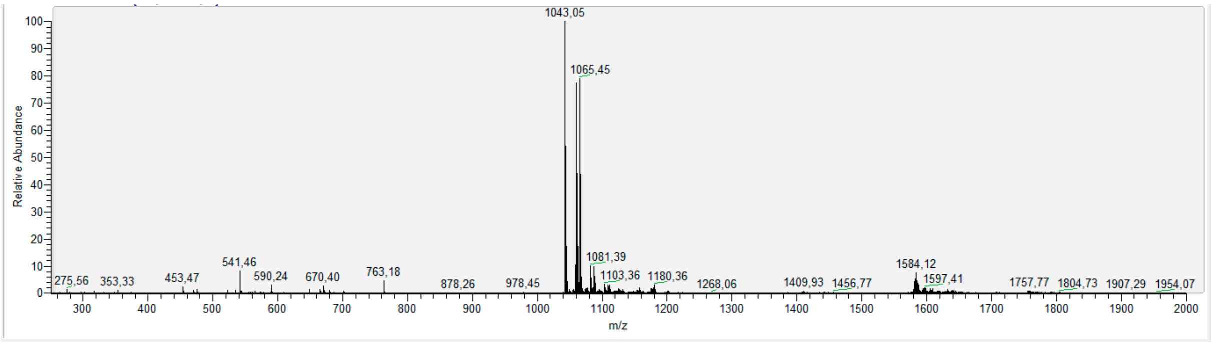

Compound 40 – D-Pro7

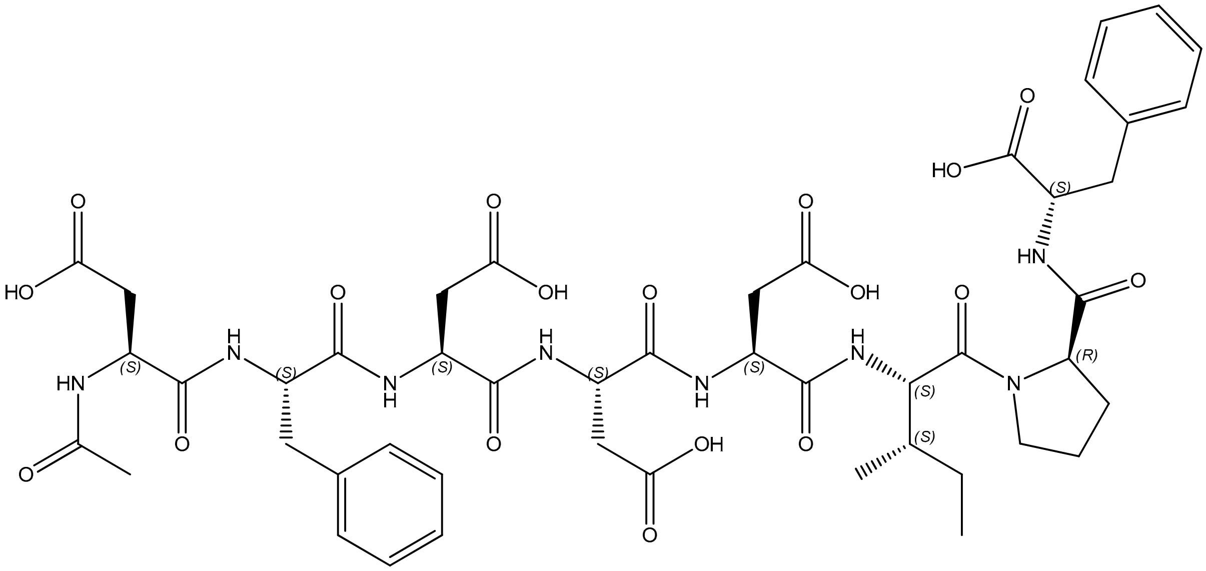

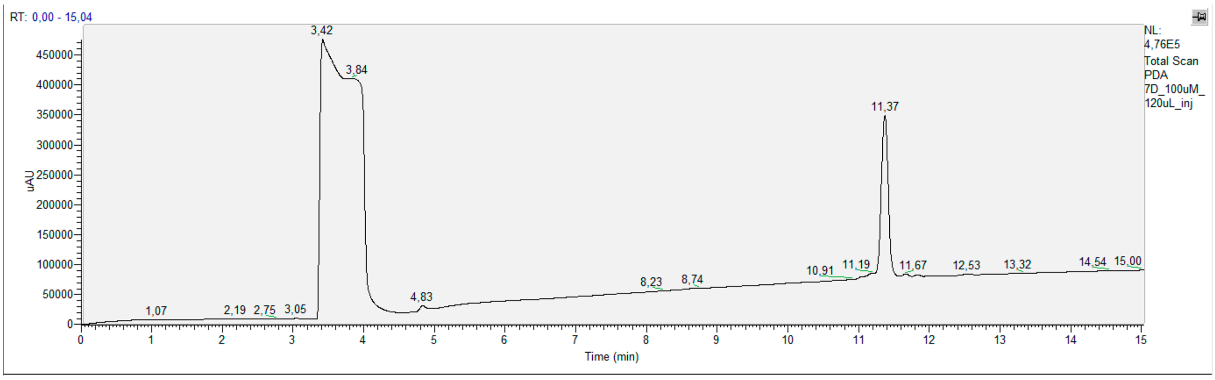

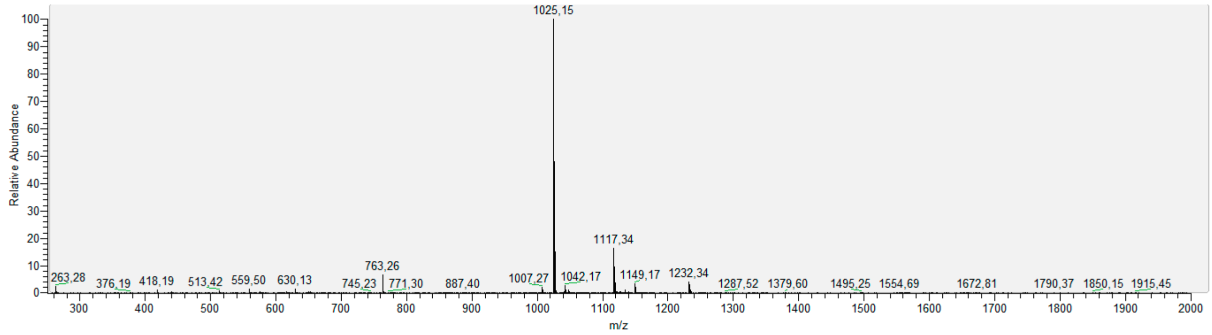

Compound 41 – Hyp7

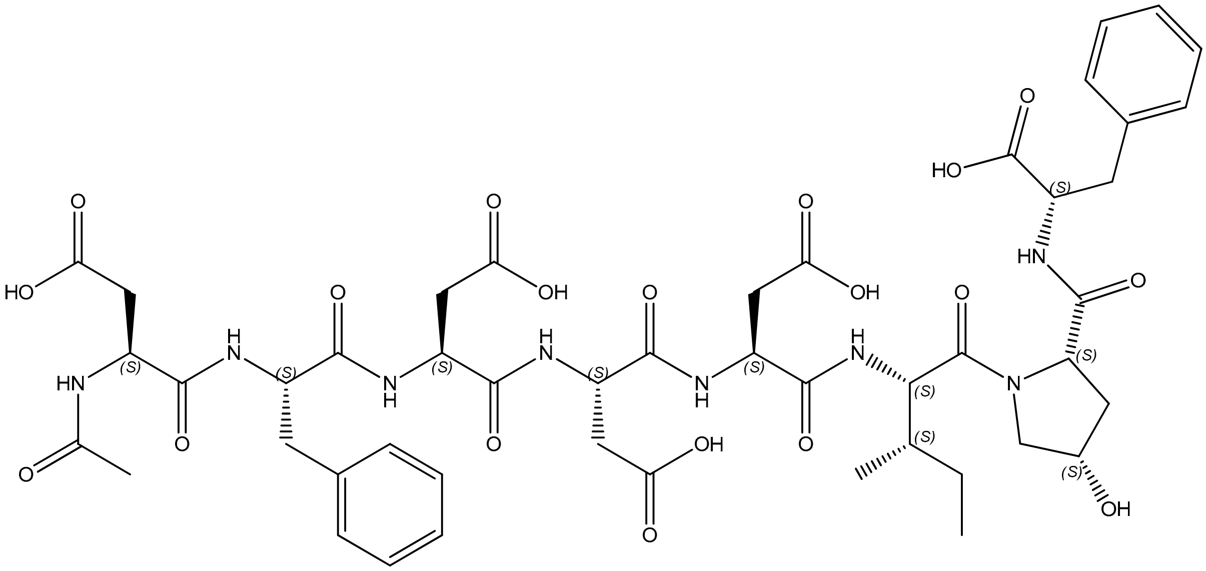

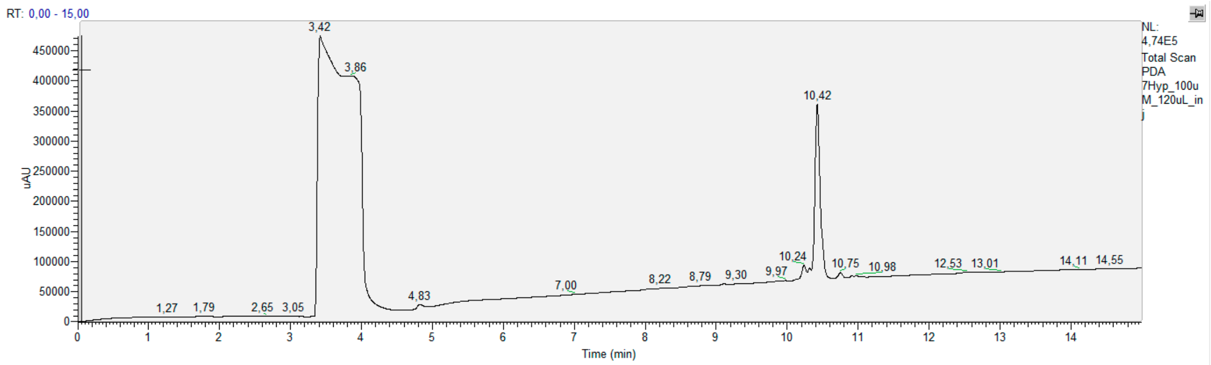

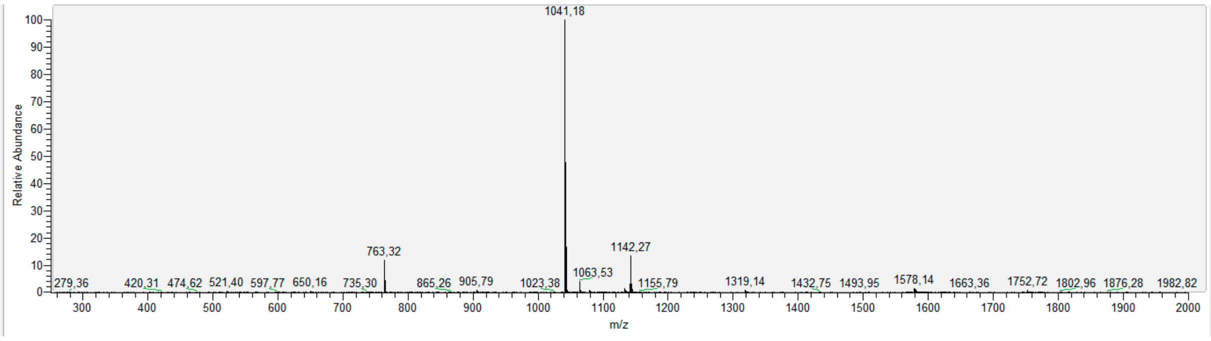

Compound 42 – Pip7

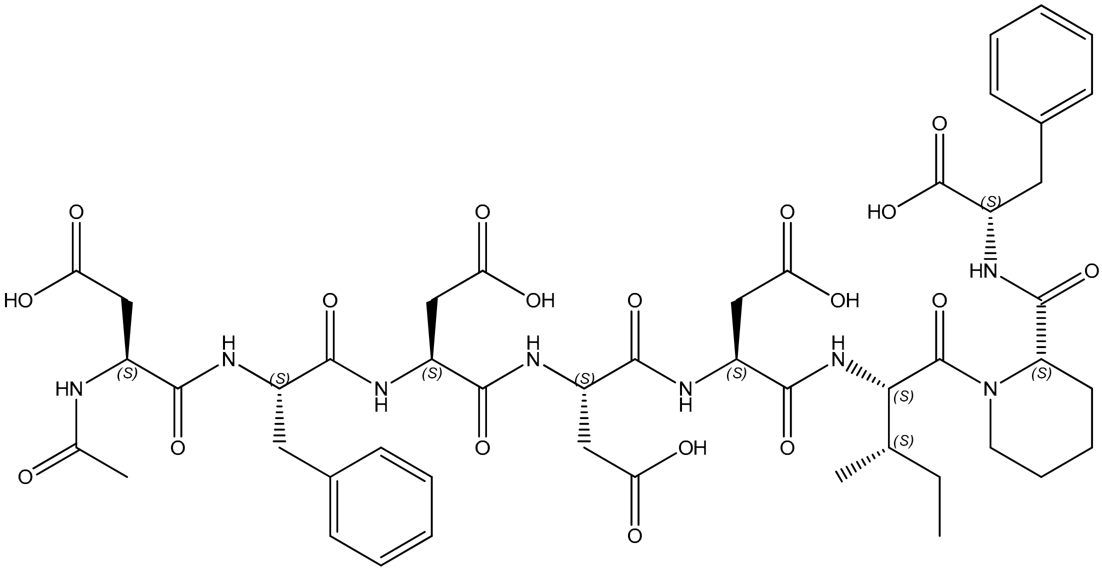

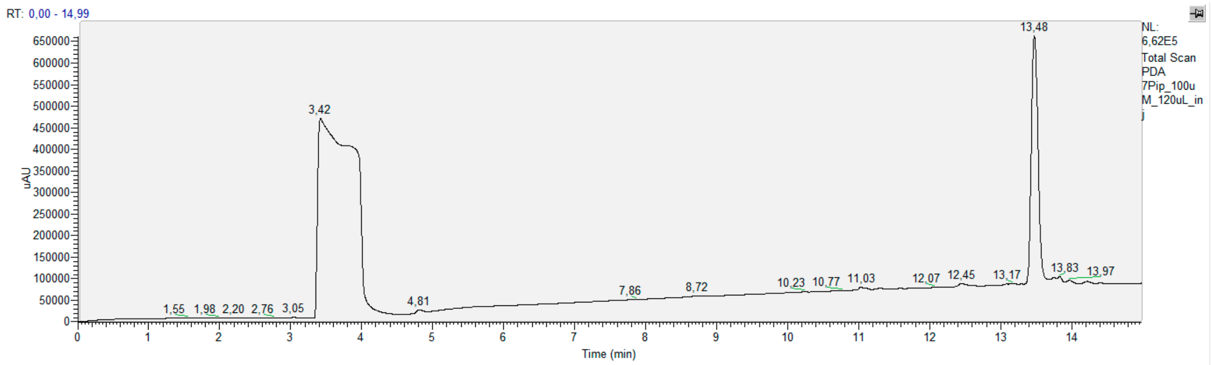

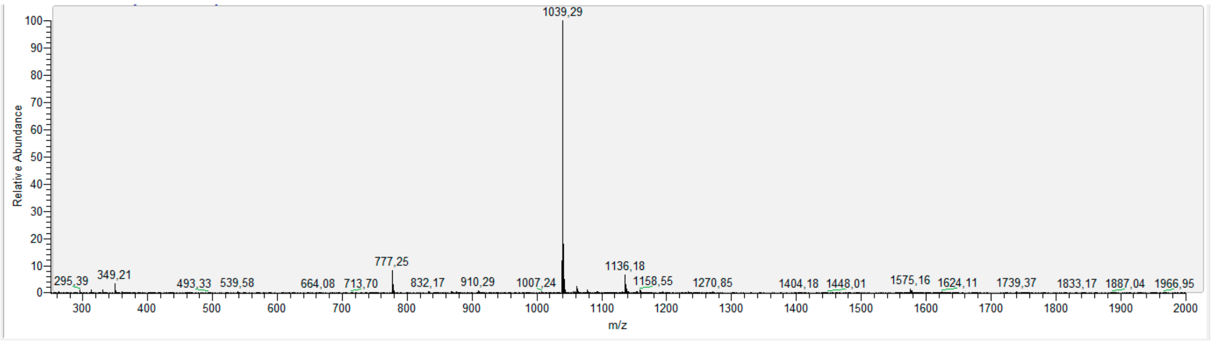

Compound 43 – β3-Phe8

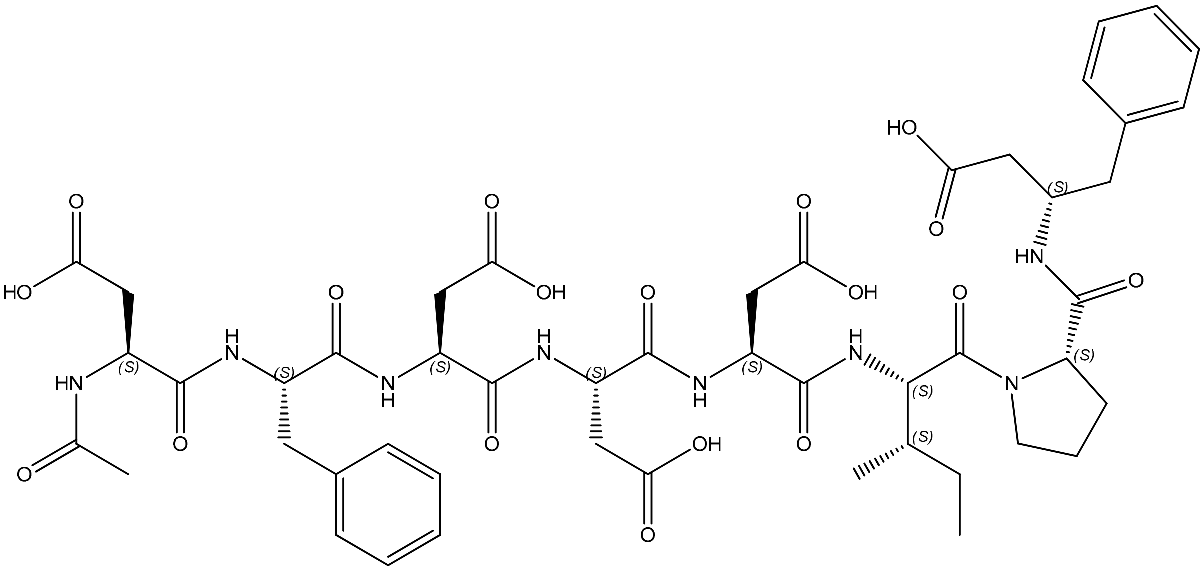

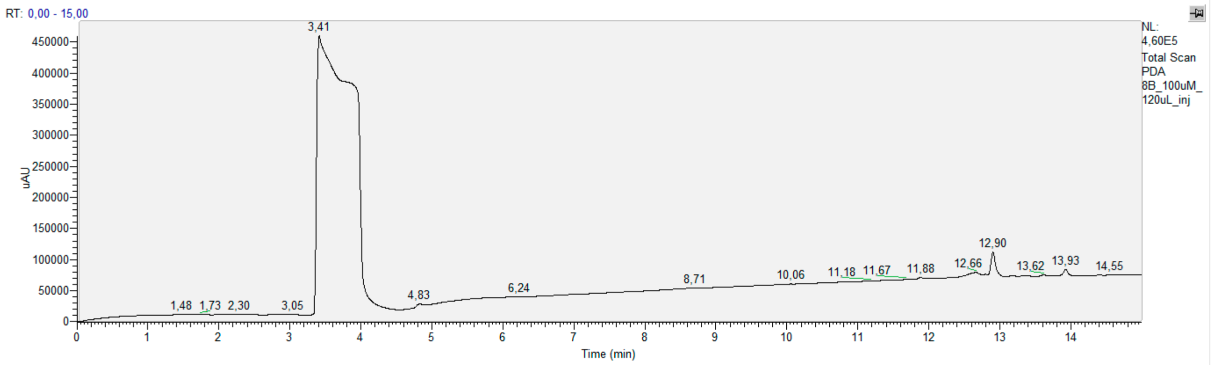

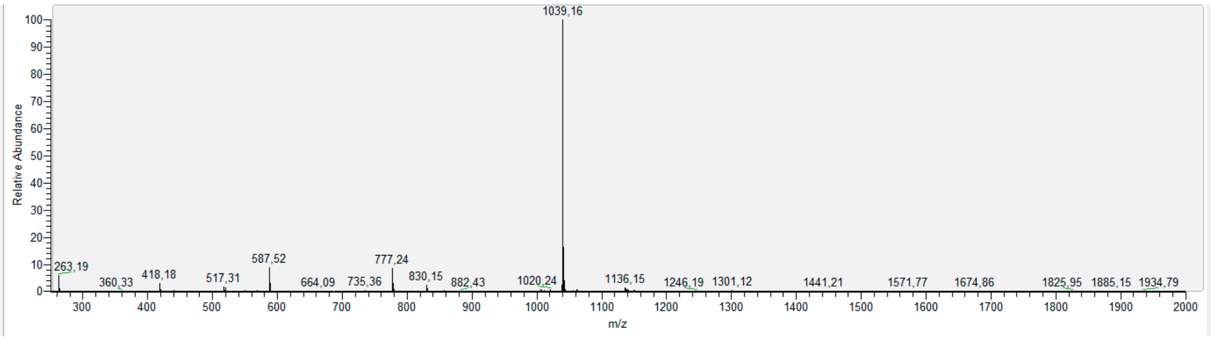

Compound 44 – 3Cl-Phe8

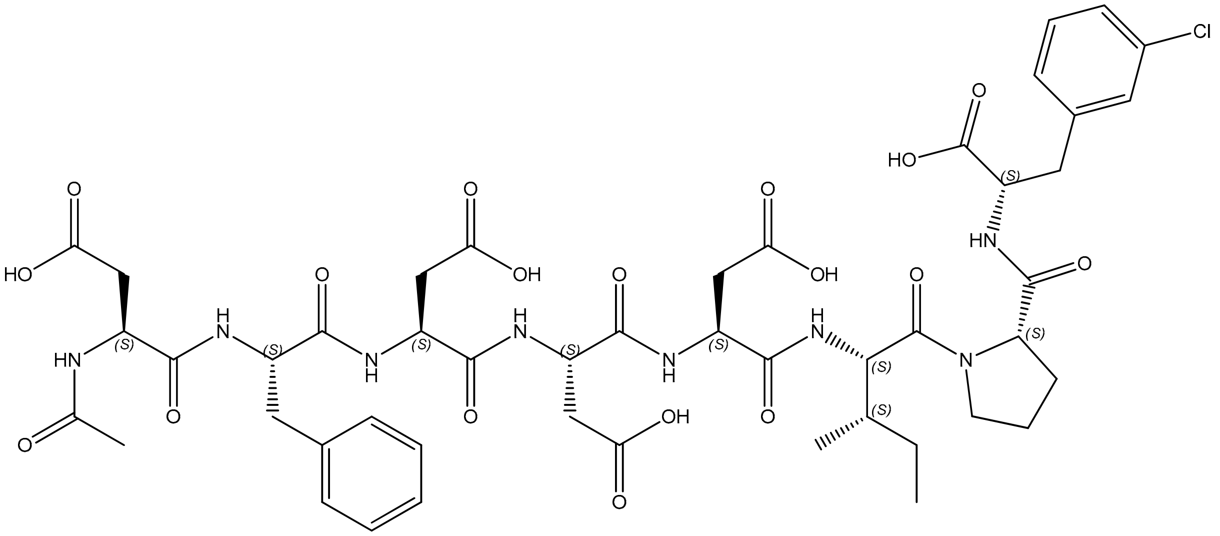

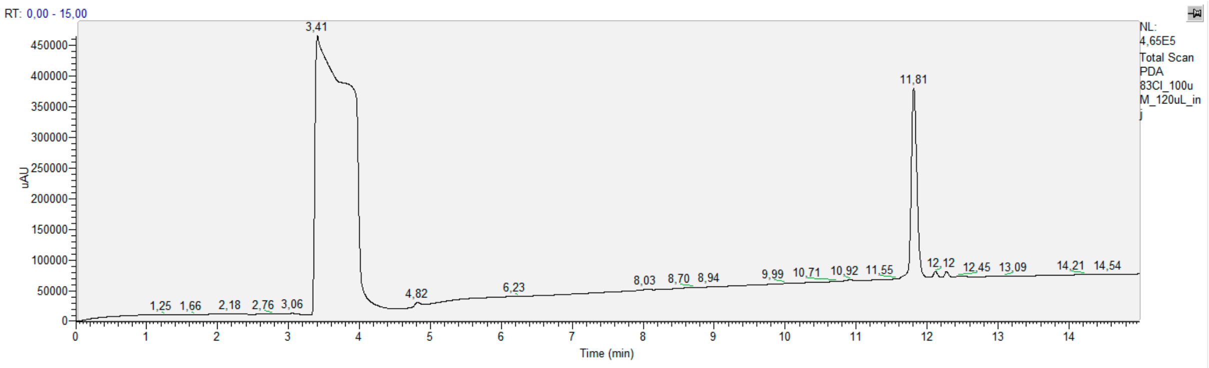

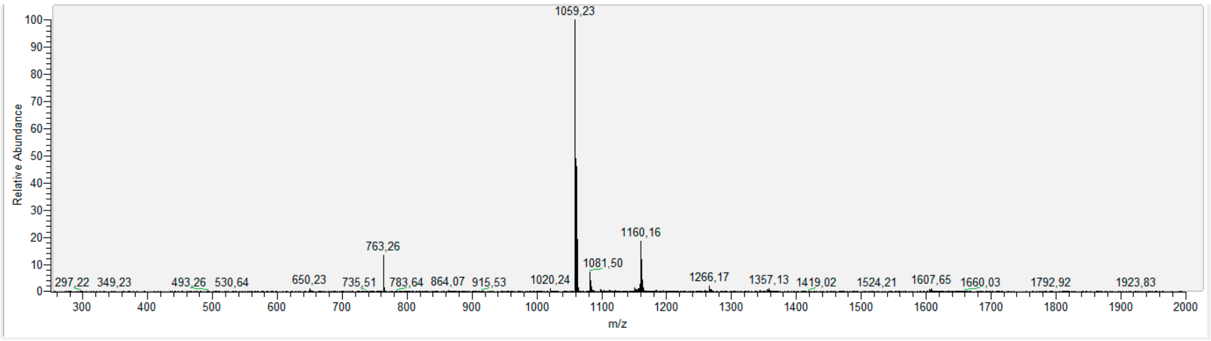

Compound 45 – 4Cl-Phe8

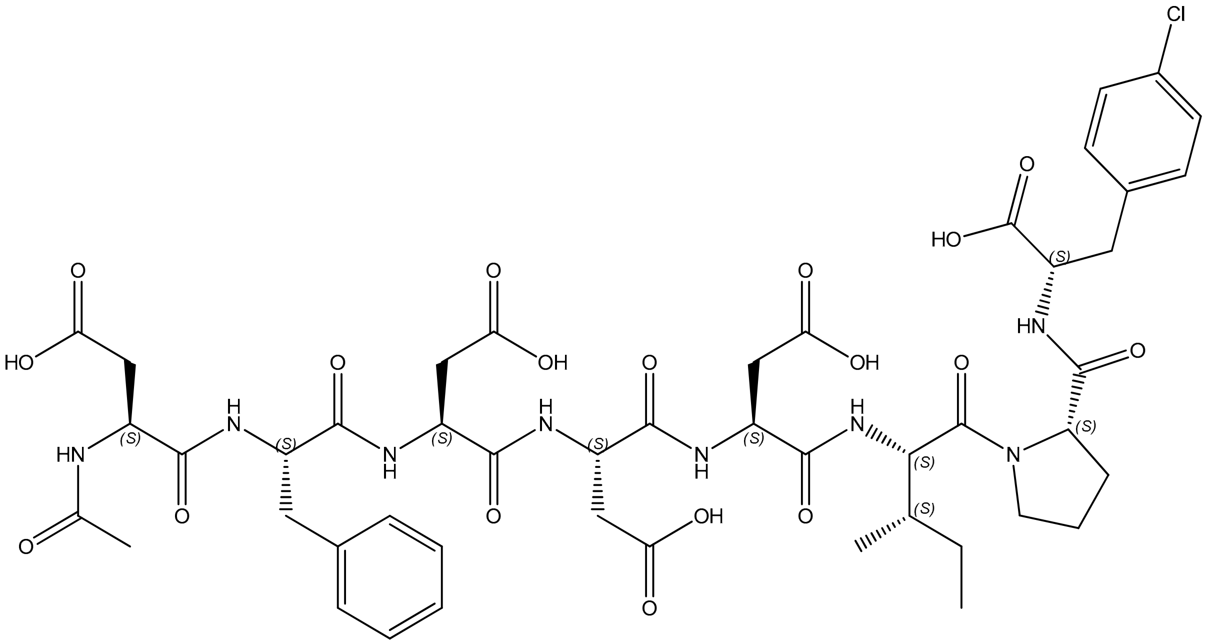

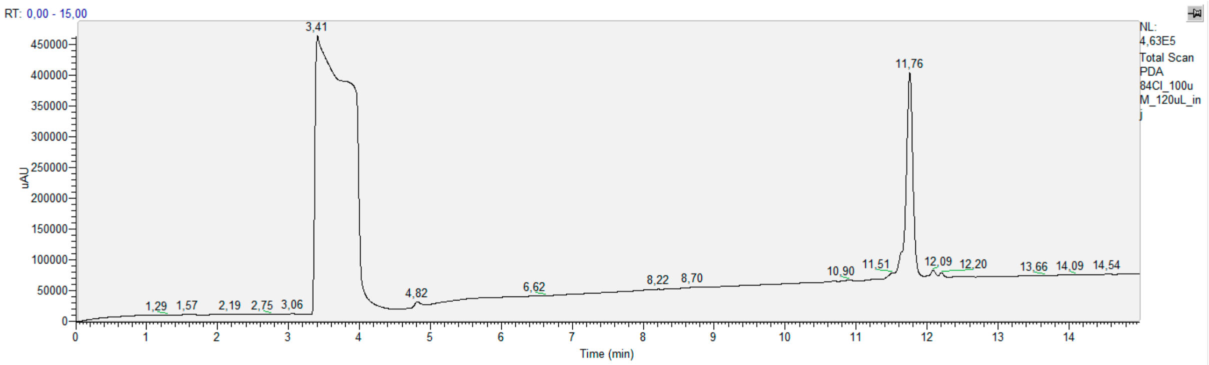

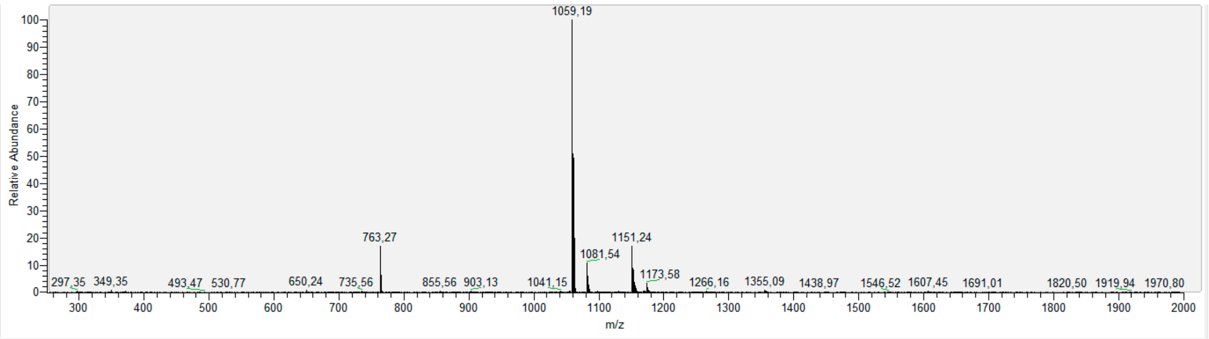

Compound 46 – D-Phe8

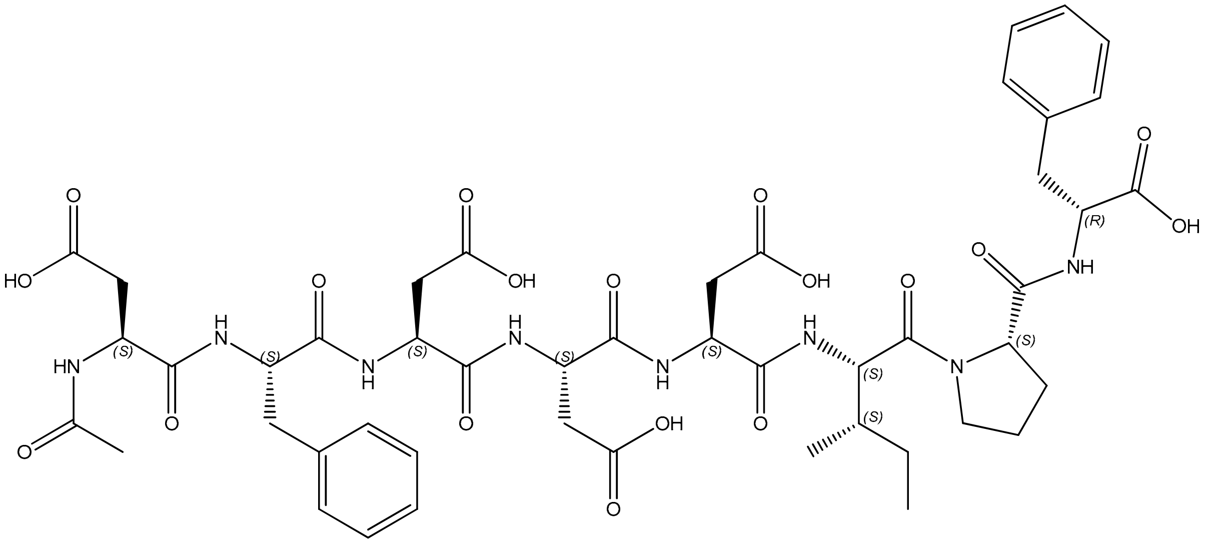

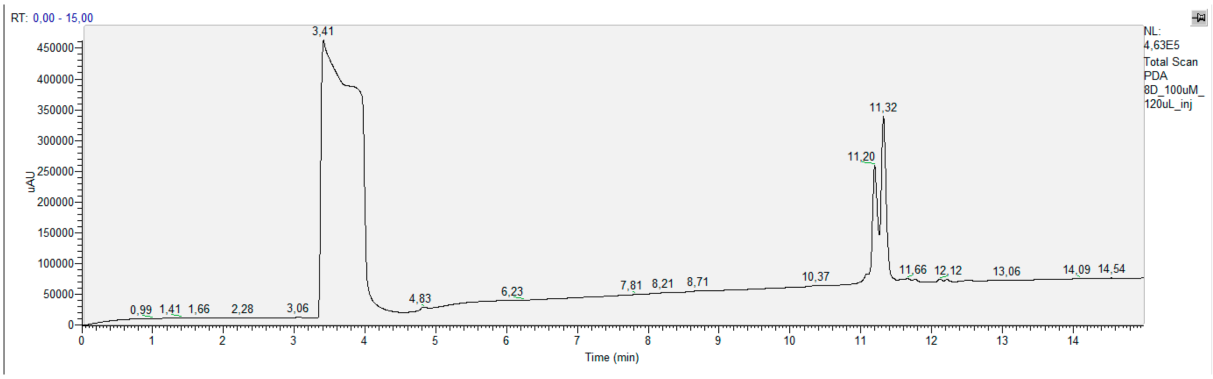

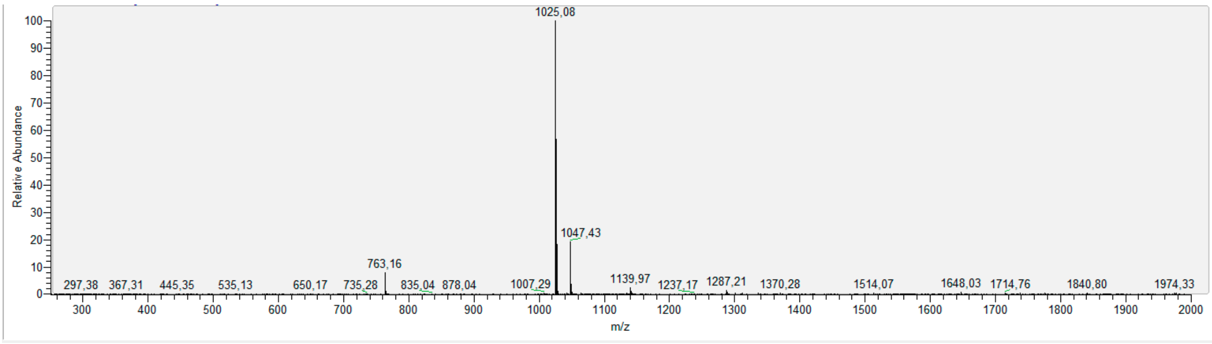

Compound 47 – F-Phe8

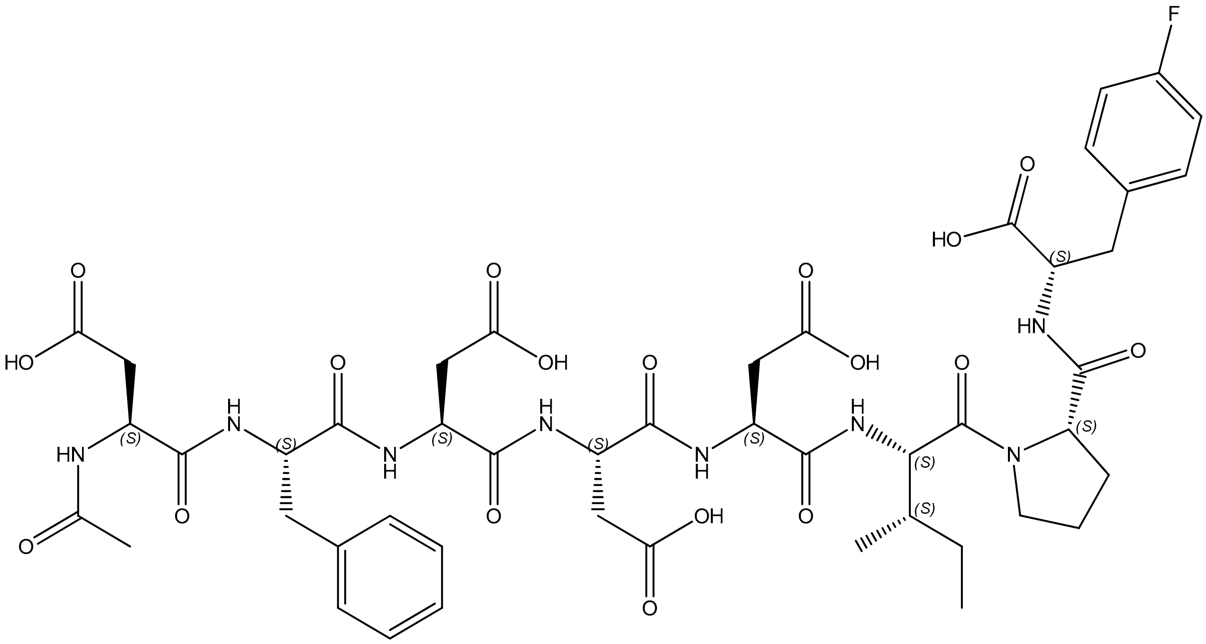

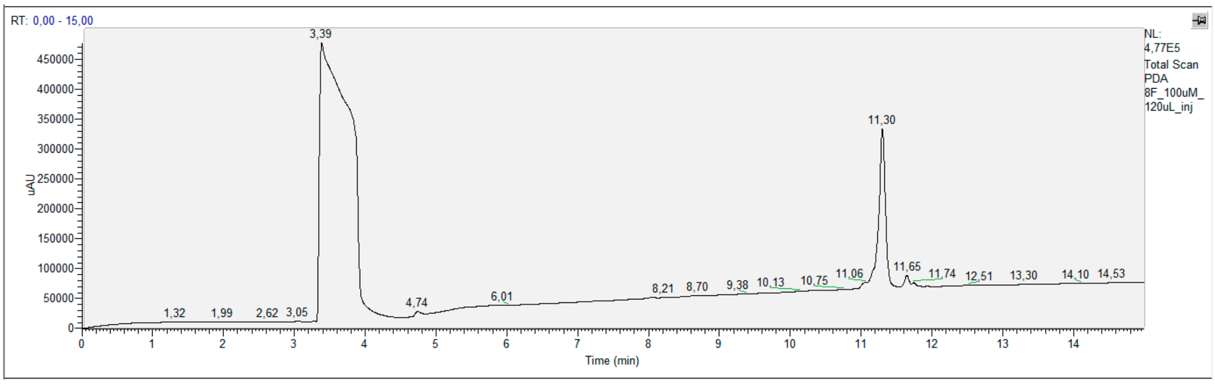

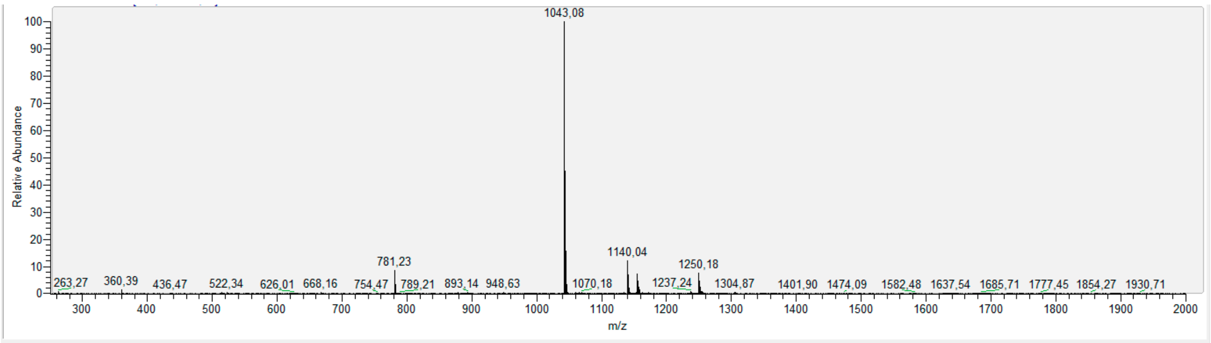

Compound 48 – NM-Phe8

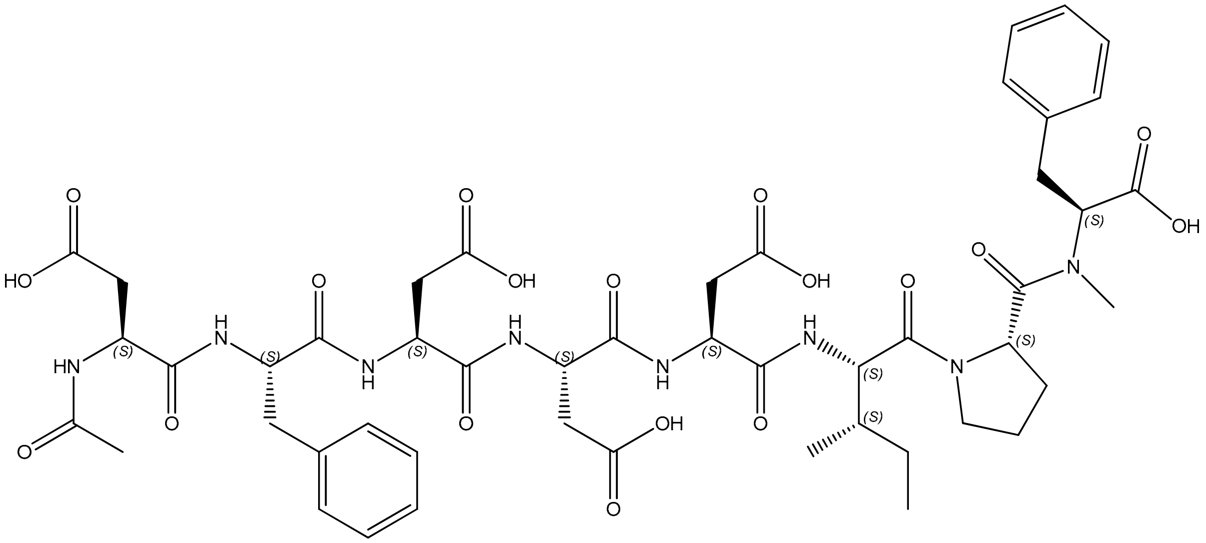

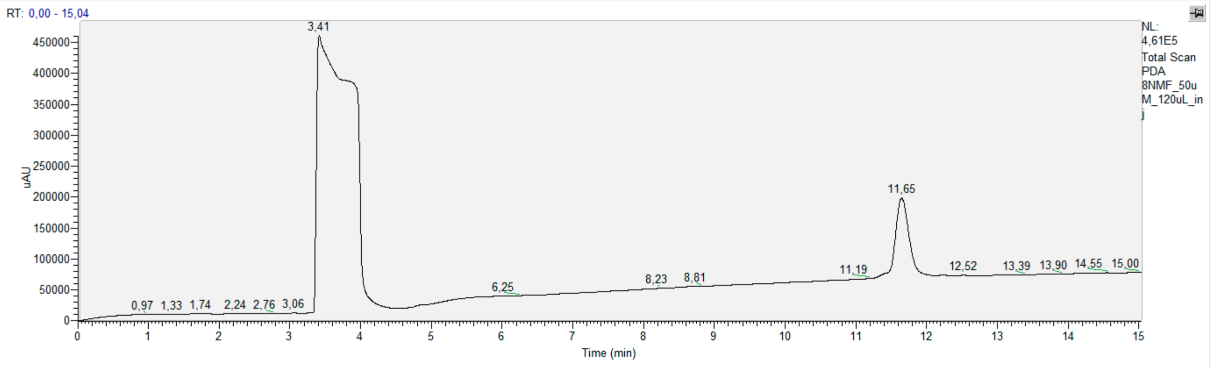

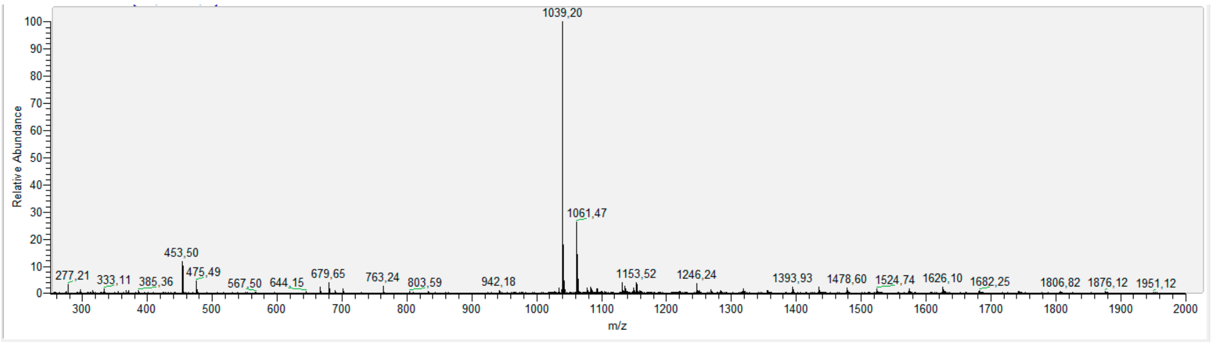

Compound 49 – 3Pal8

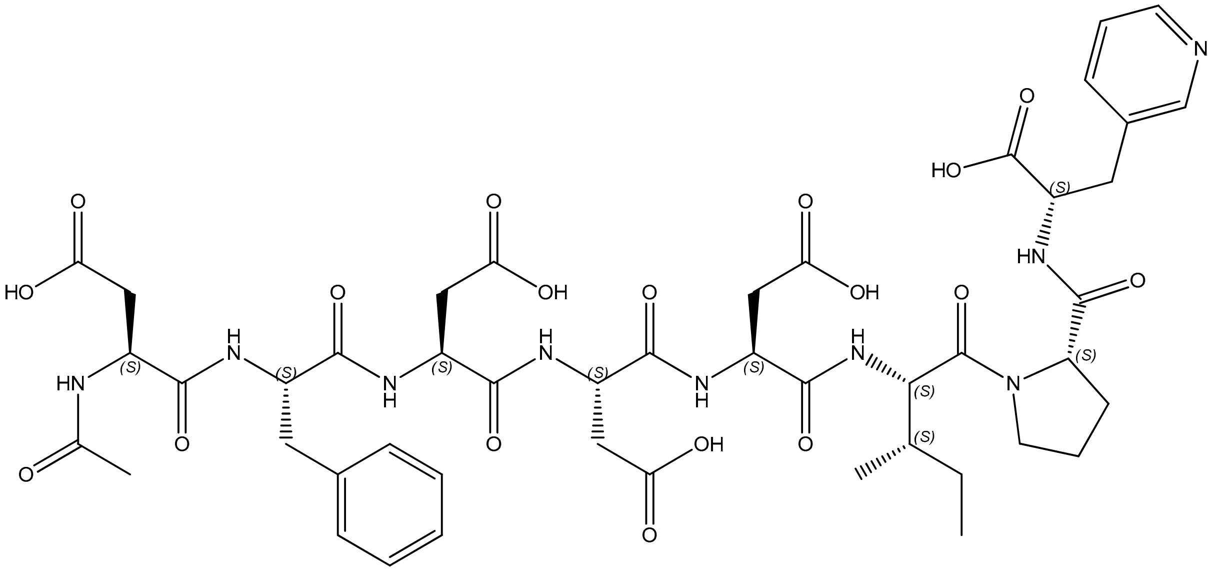

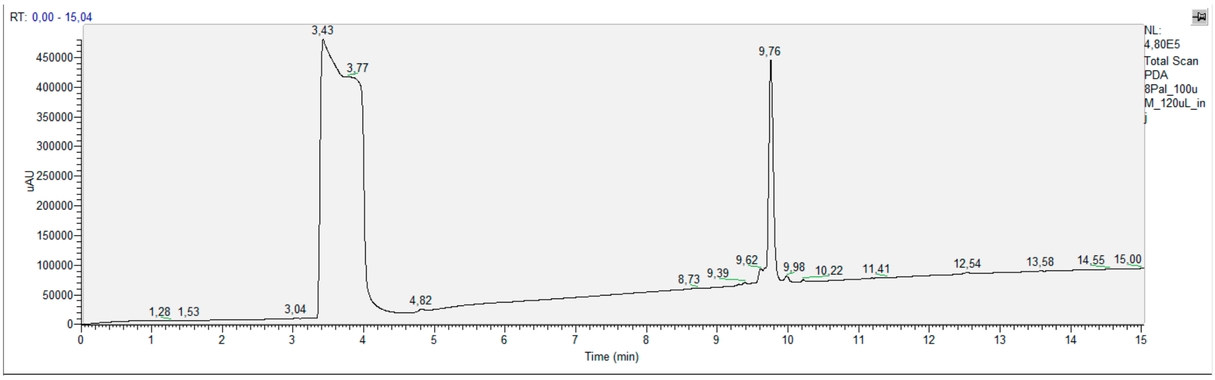

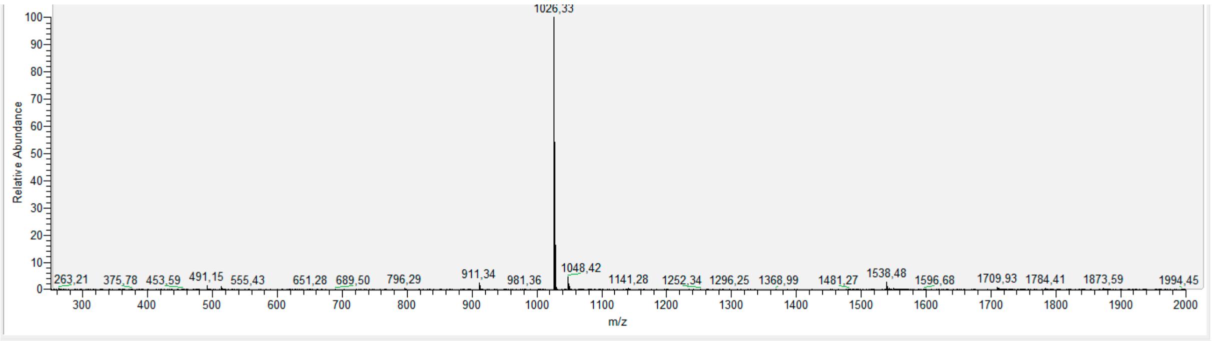

Compound 50 – Thi8

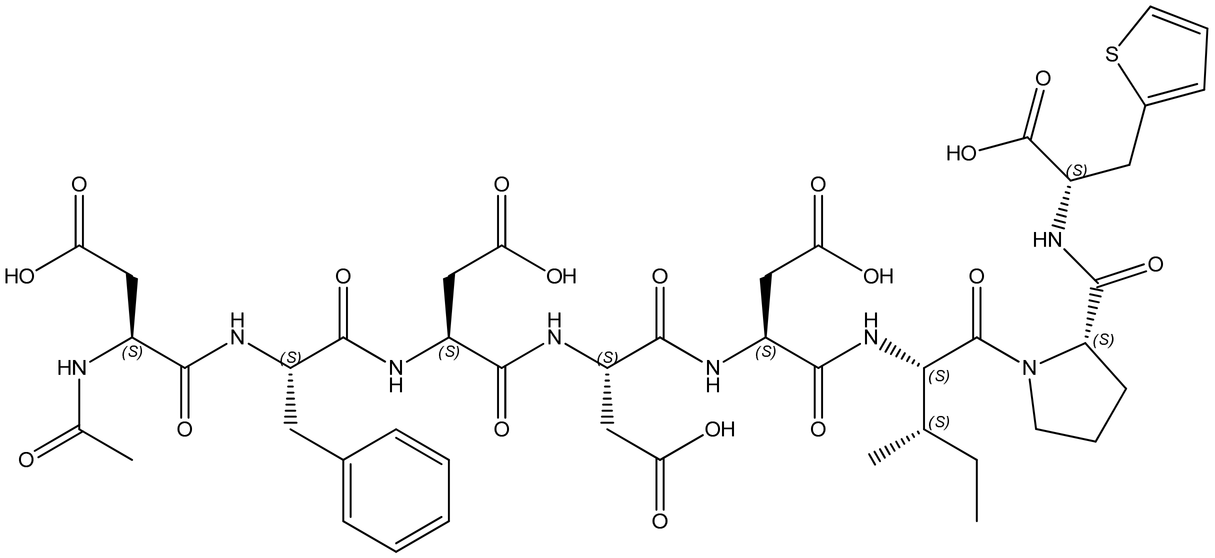

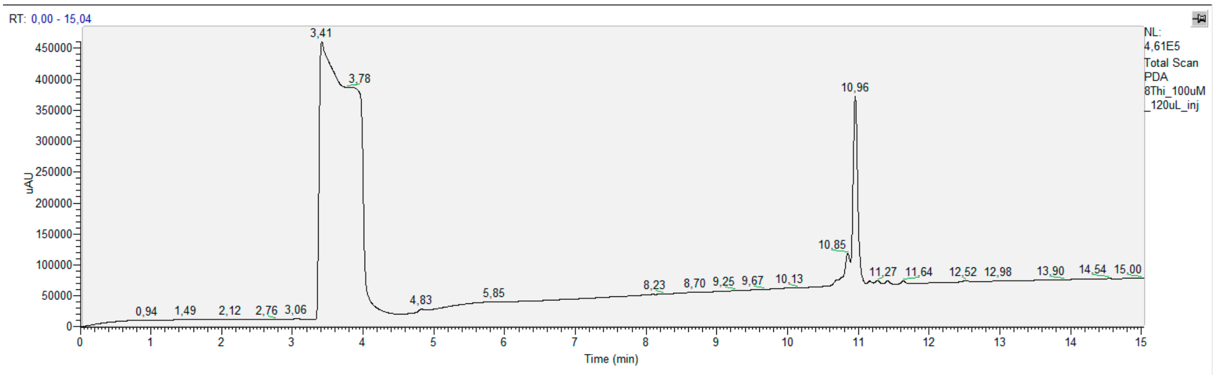

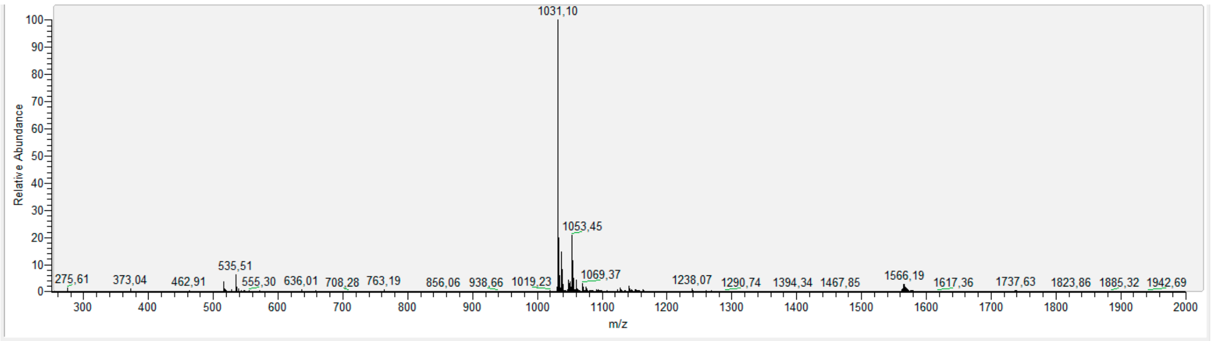

Compound 51 – Tic8

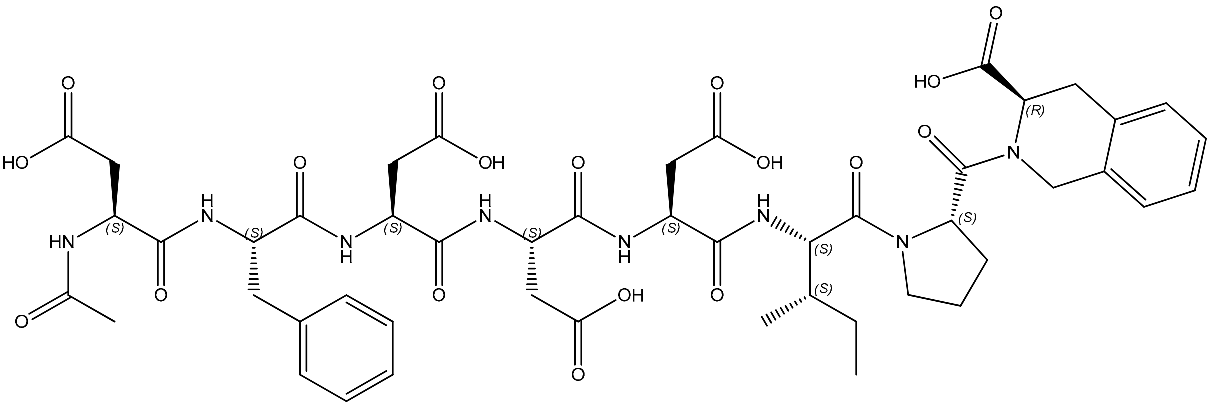

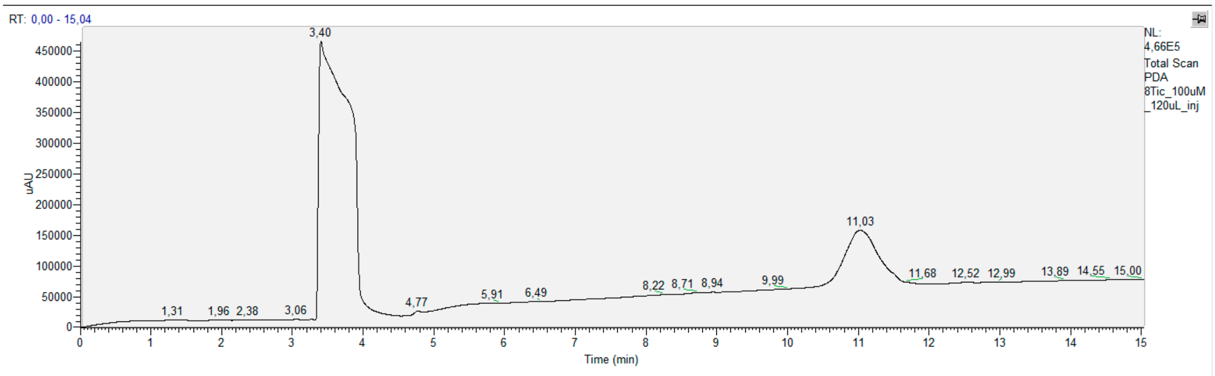

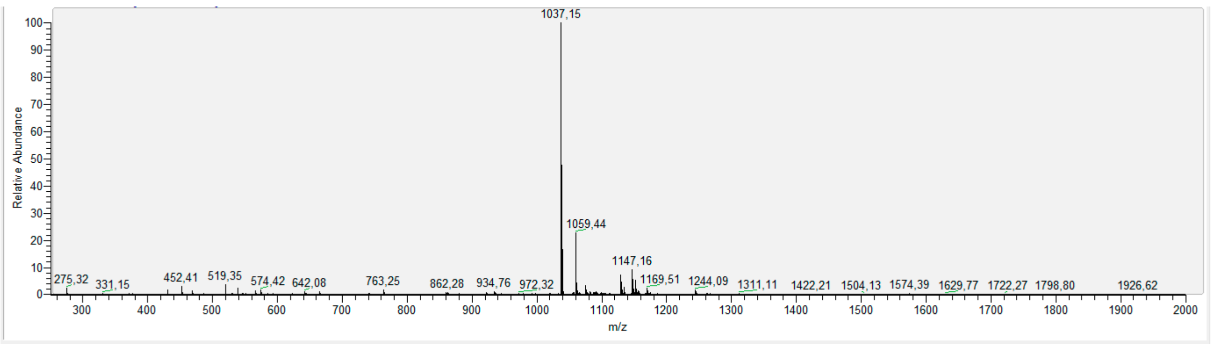

Compound 52 – 4CF3-Phe8

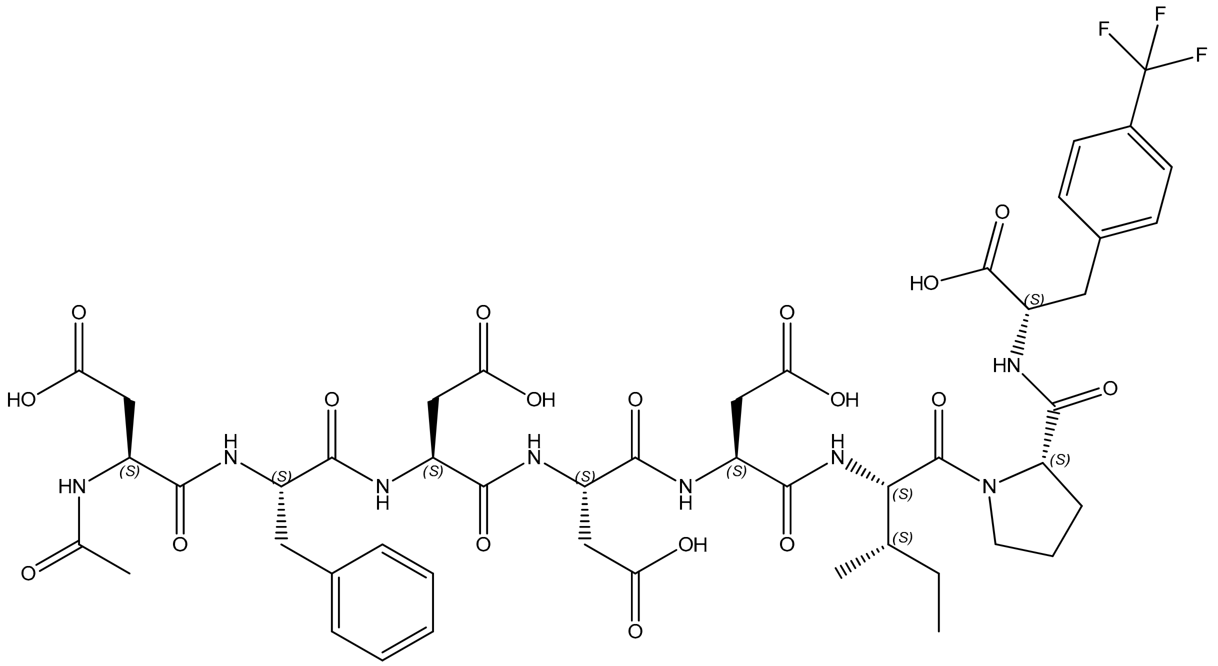

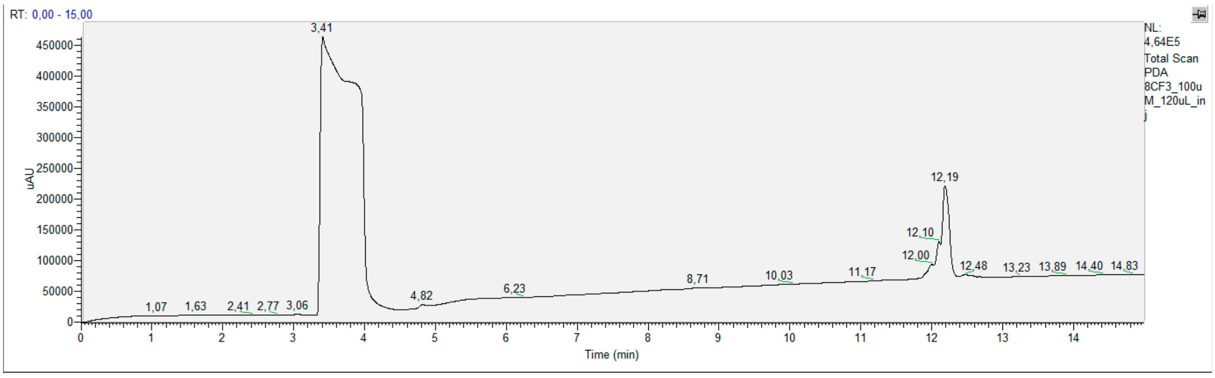

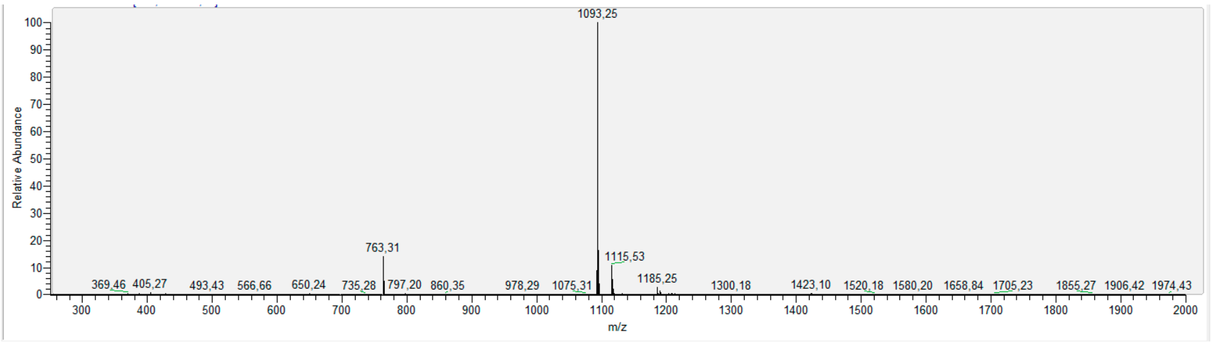

Compound 53 – F-wtSSB-Ct

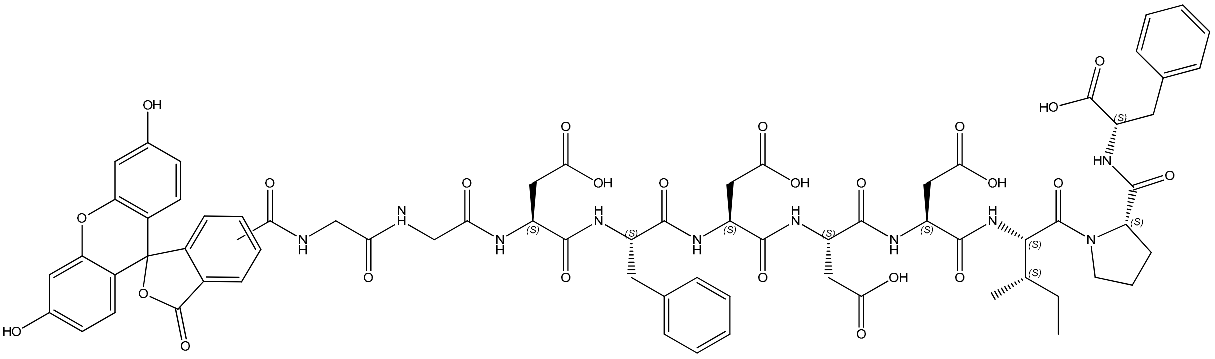

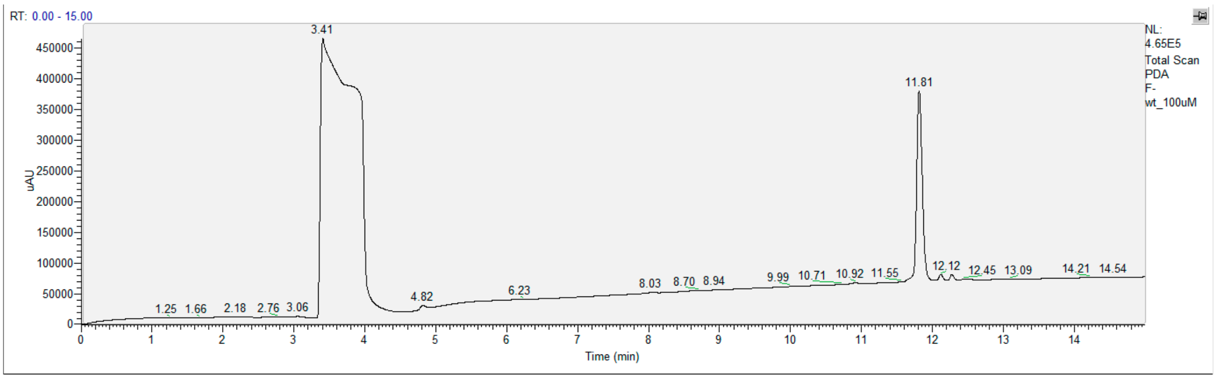

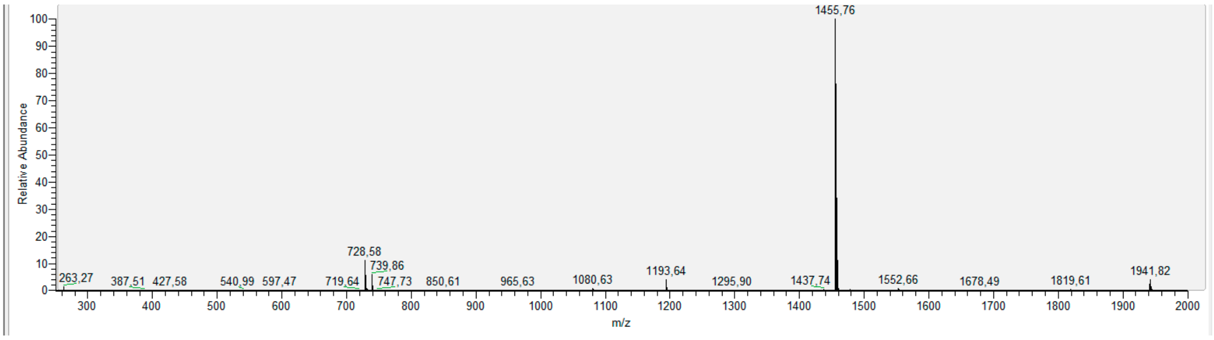

Compound 54 – F-4Cl-Phe2

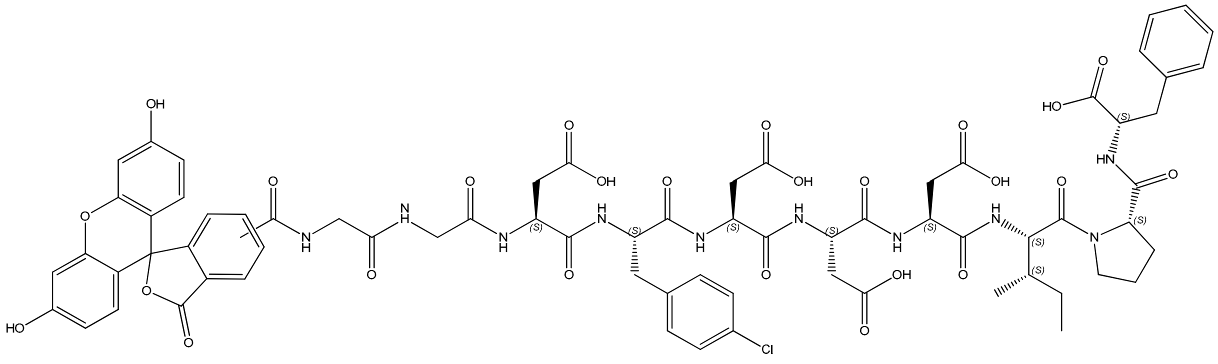

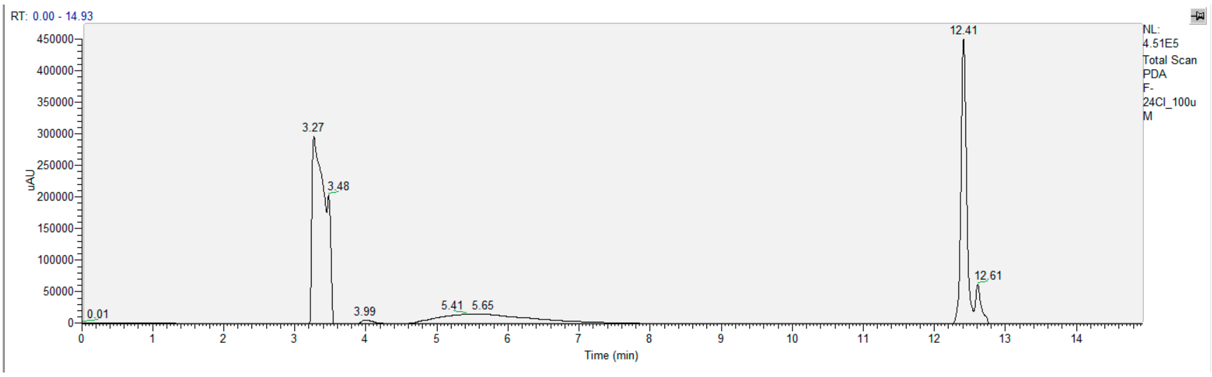

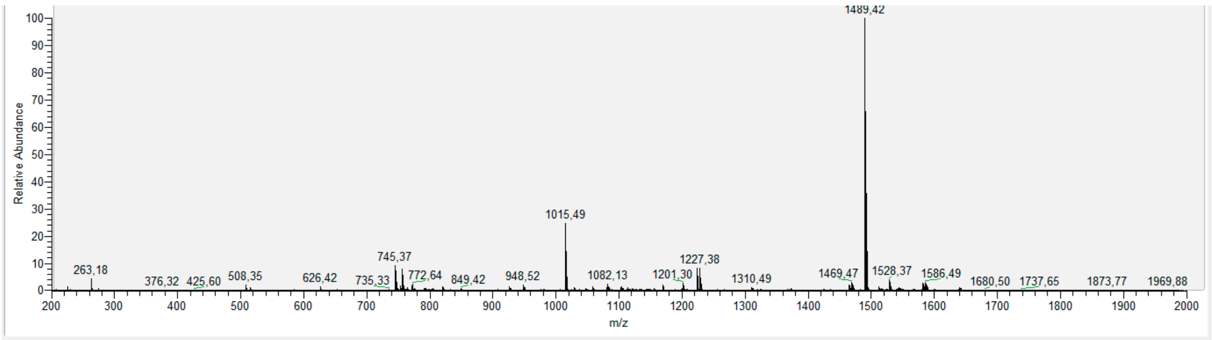

Compound 55 – F-4F-Phe2

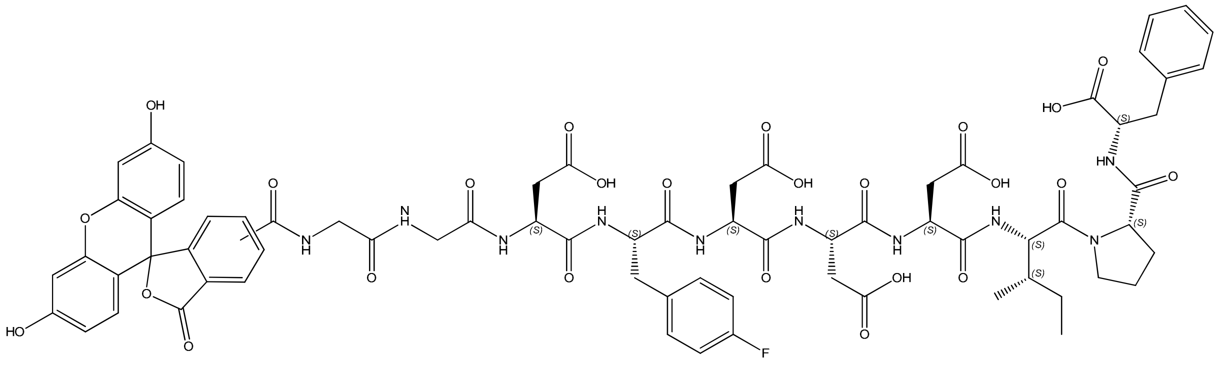

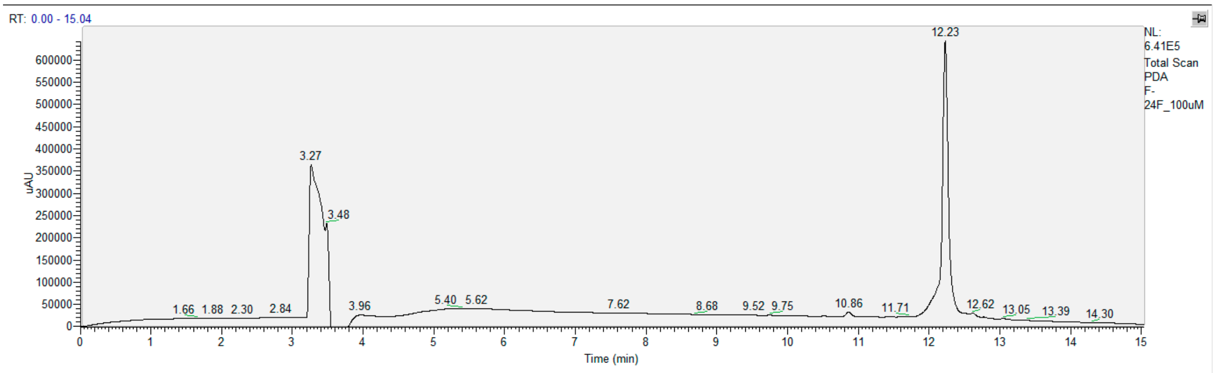

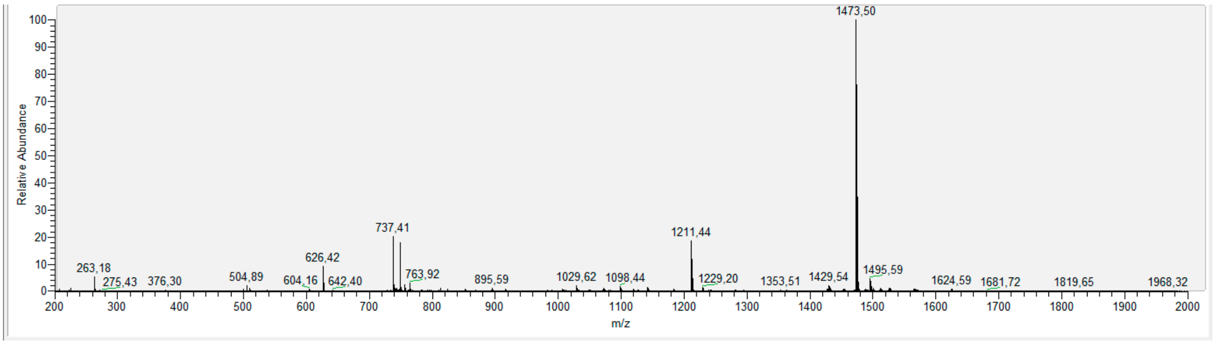

Compound 56 – F-4CF3-Phe2

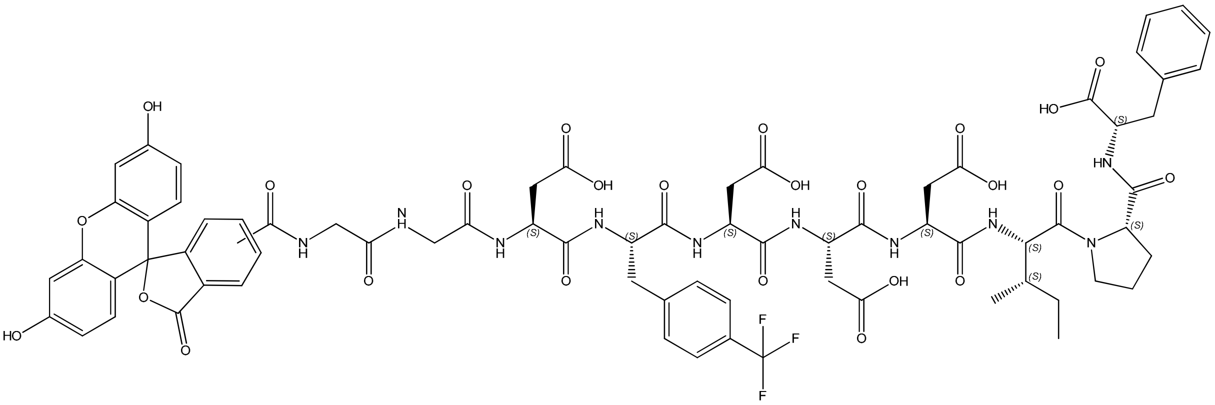

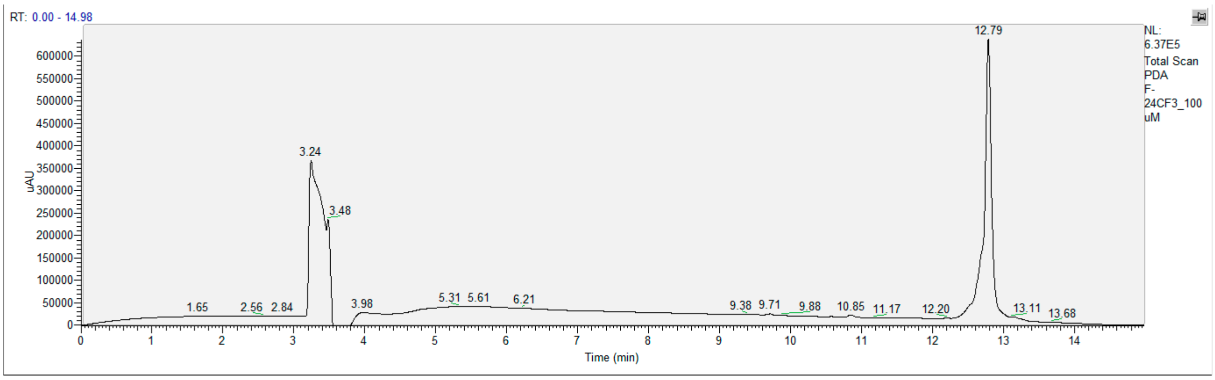

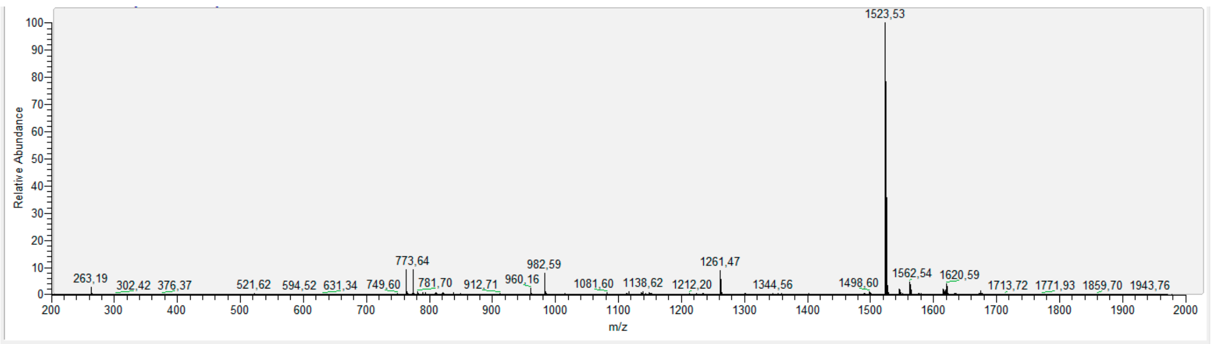

Compound 57 – F-Glu5

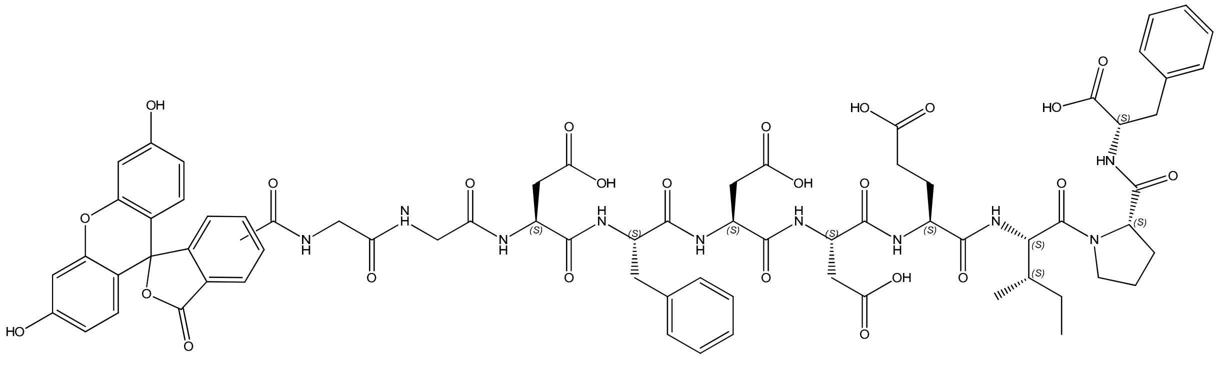

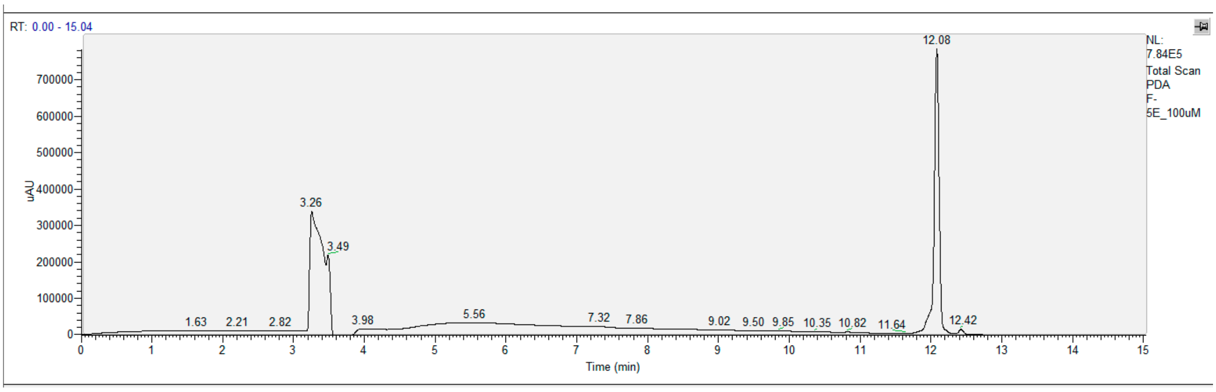

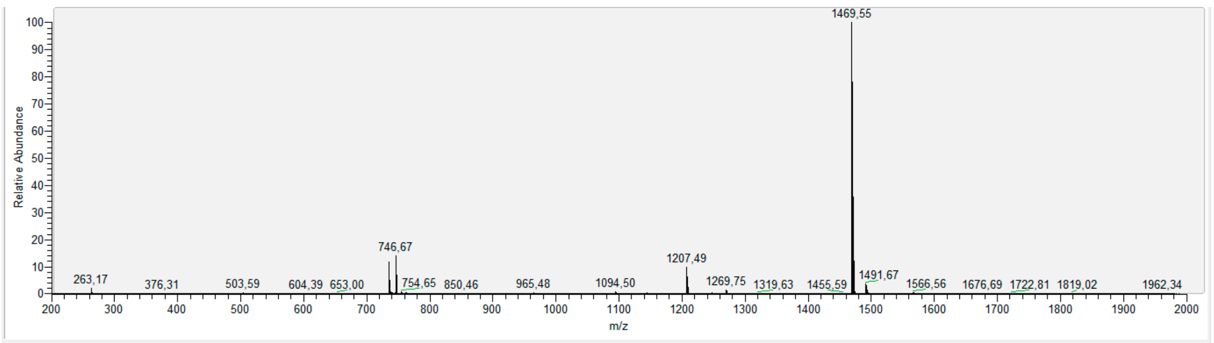

Compound 58– F-Leu6

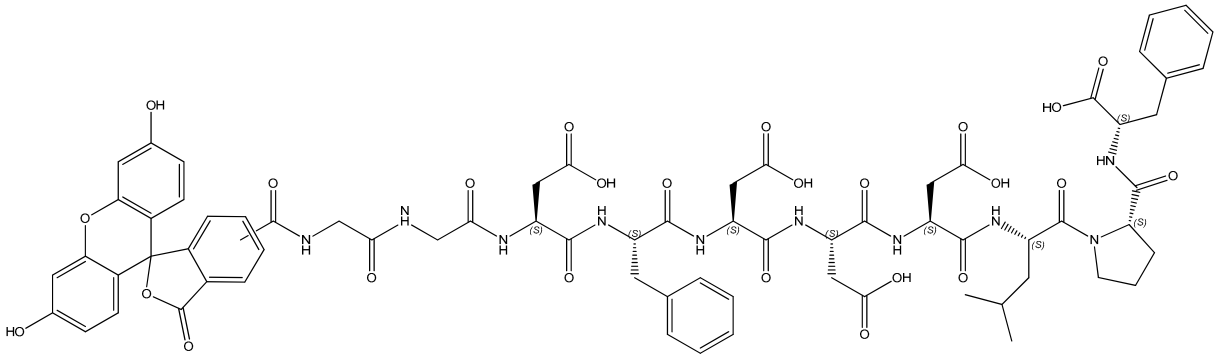

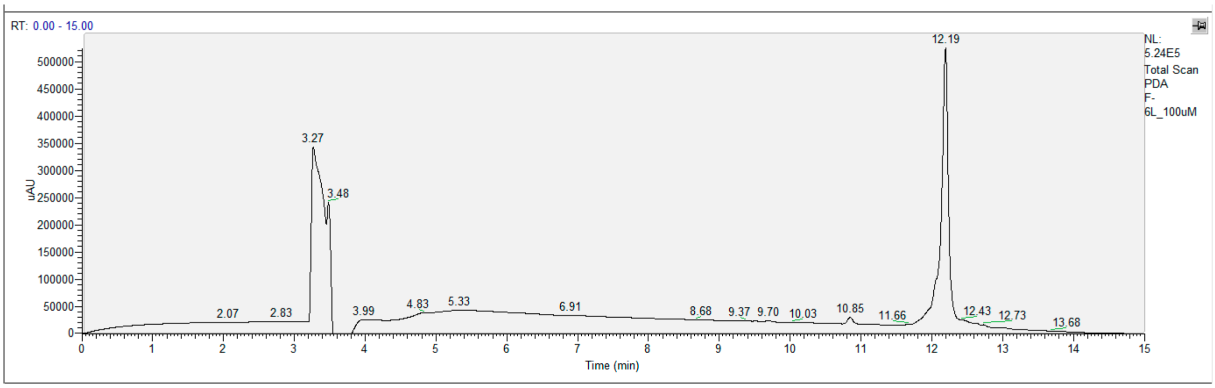

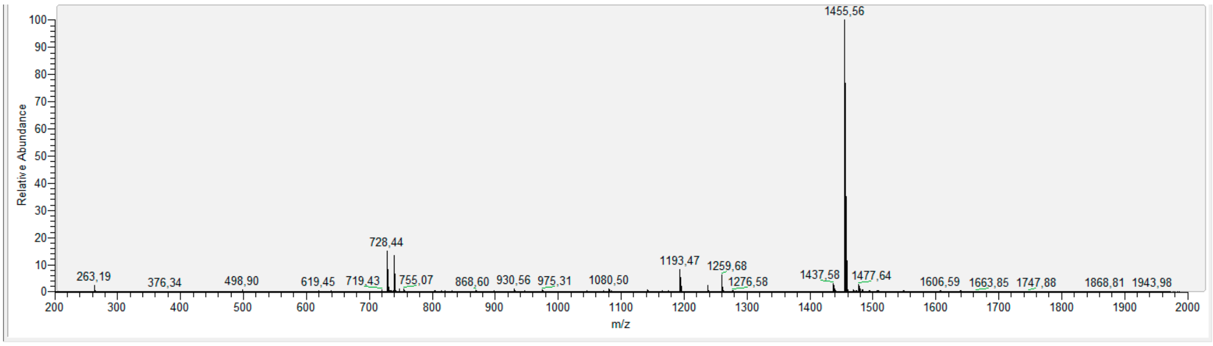

Compound 59 – F-nLe6

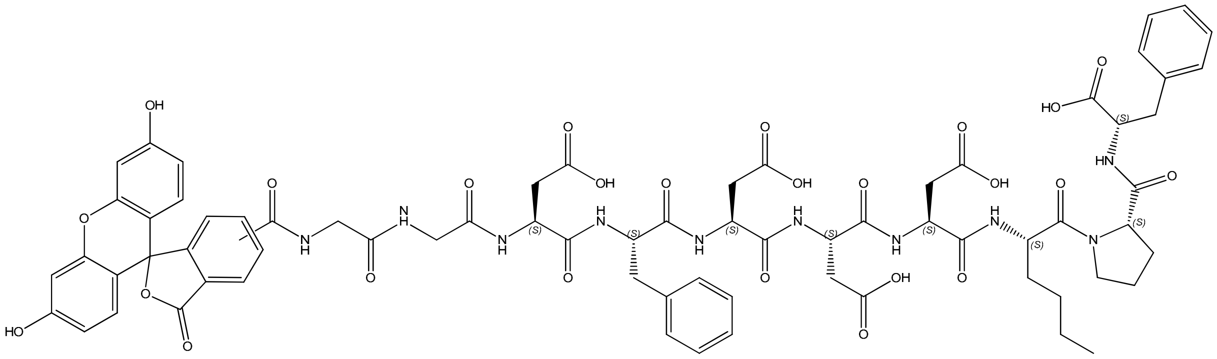

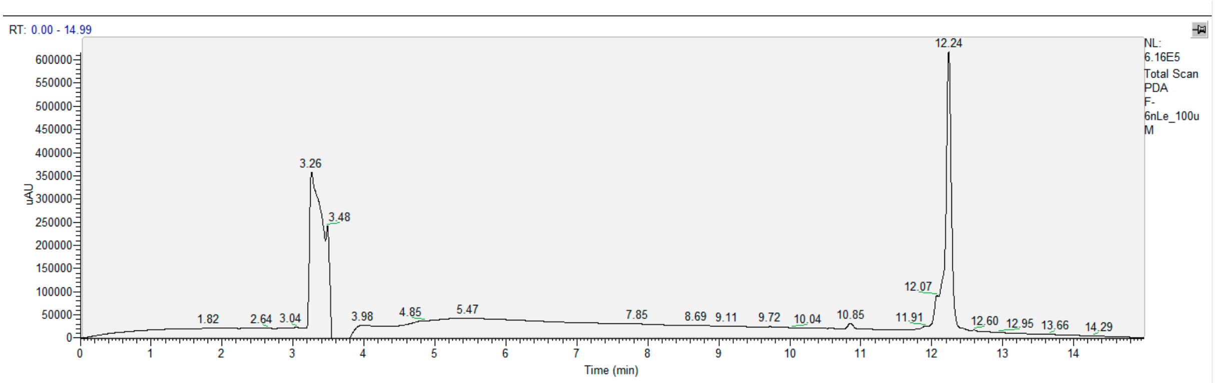

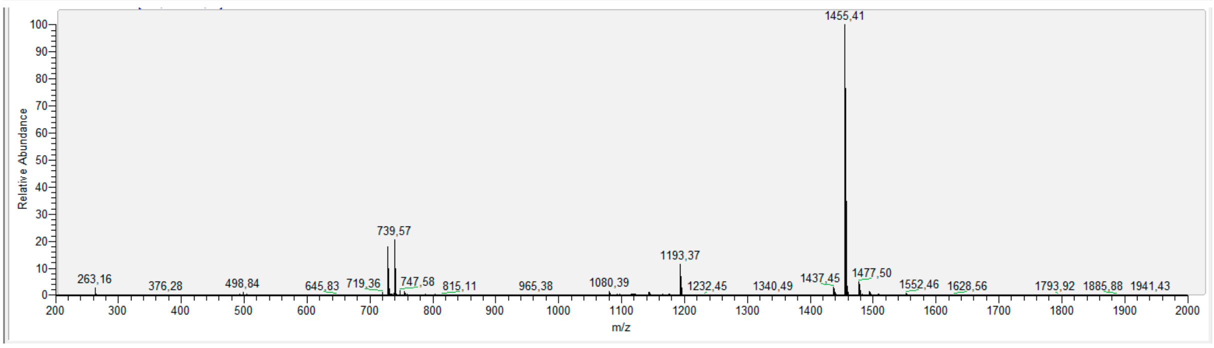

Compound 60 – F-Pip7

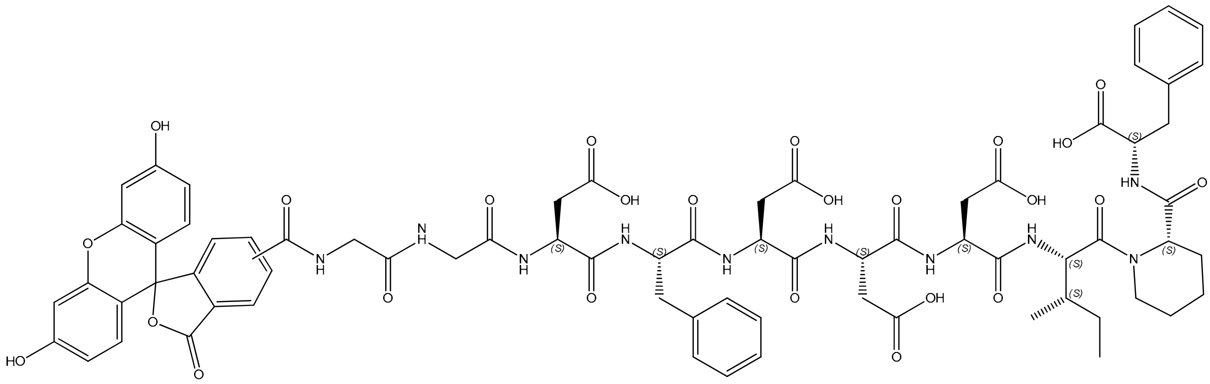

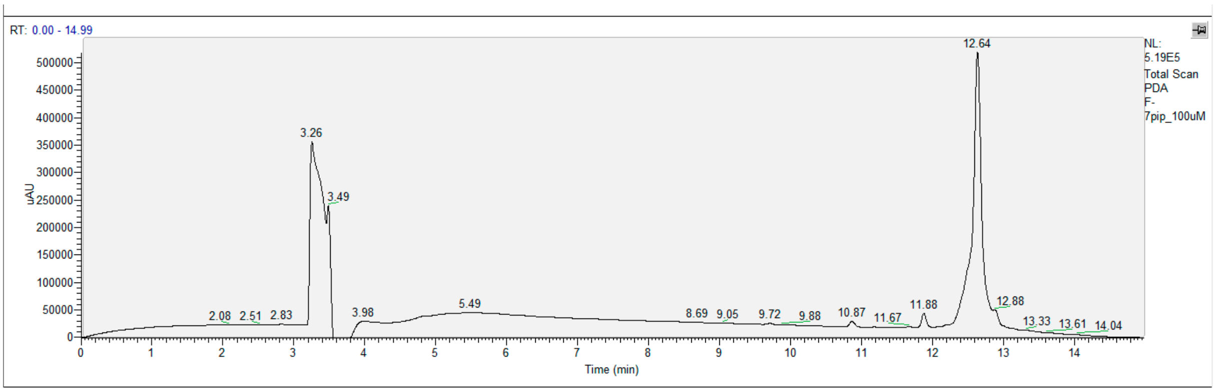

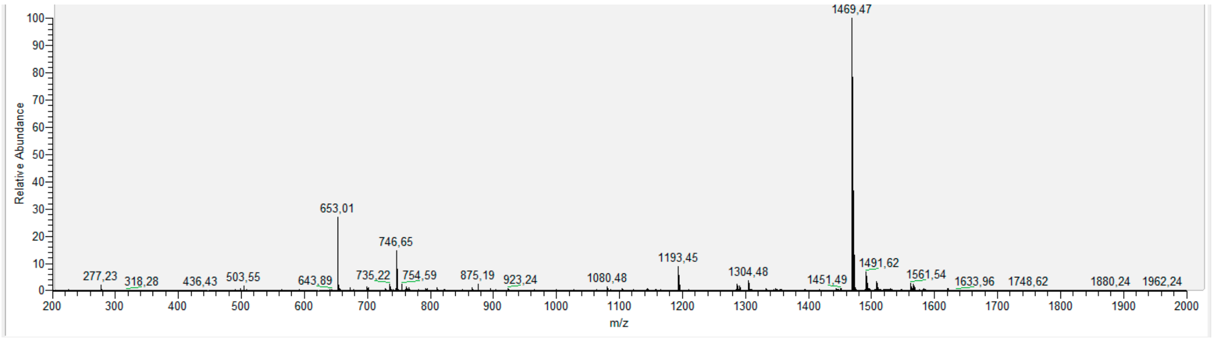

Compound 61 – F-3Cl-Phe8

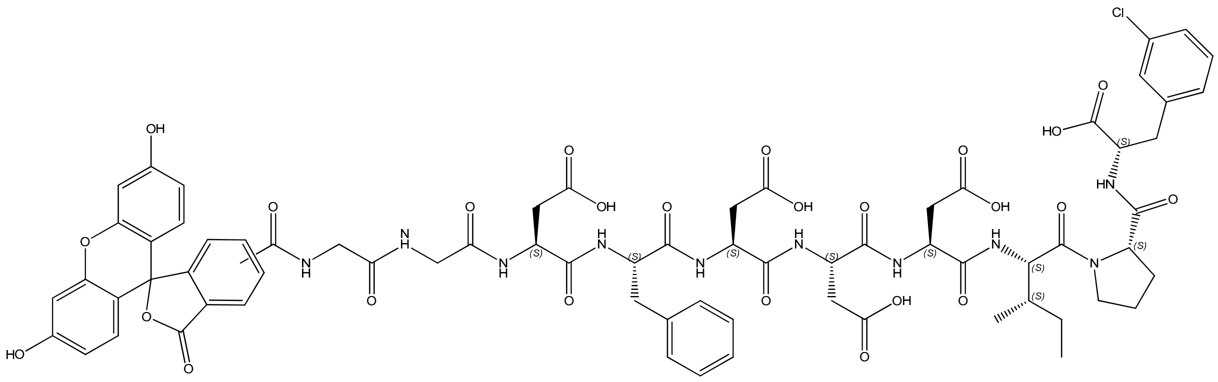

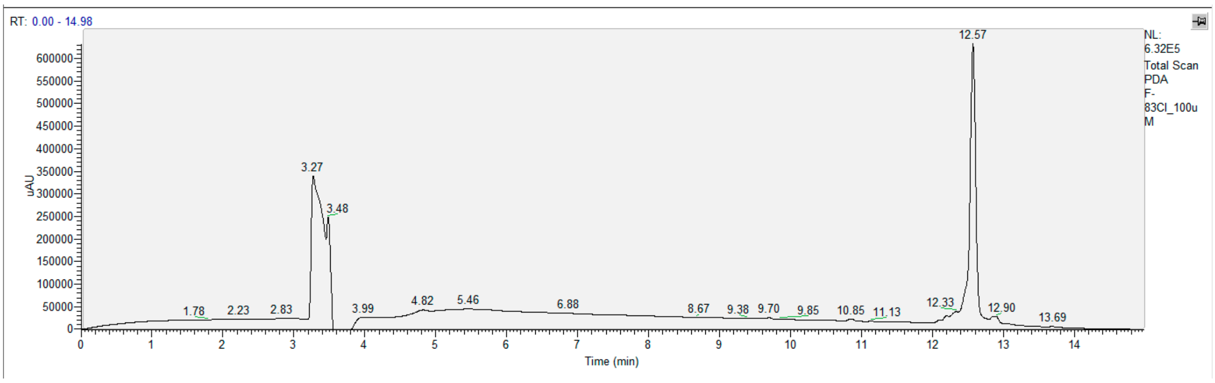

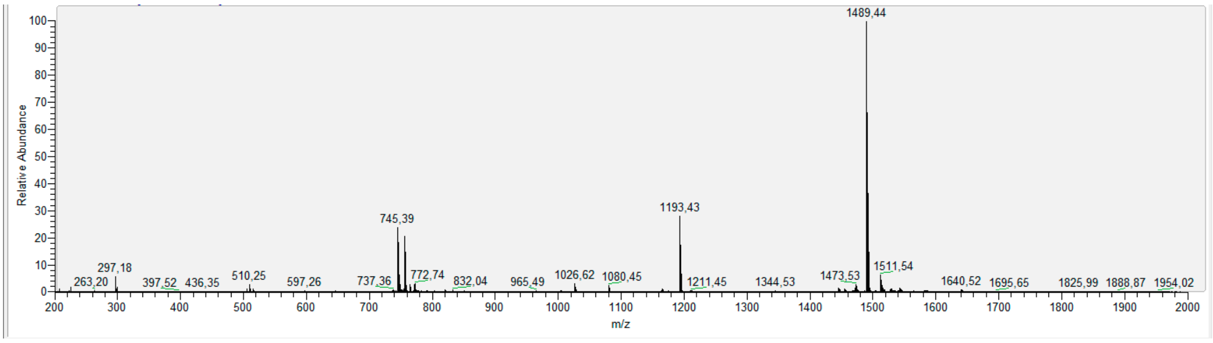

Compound 62 – F-4CF3-Phe8

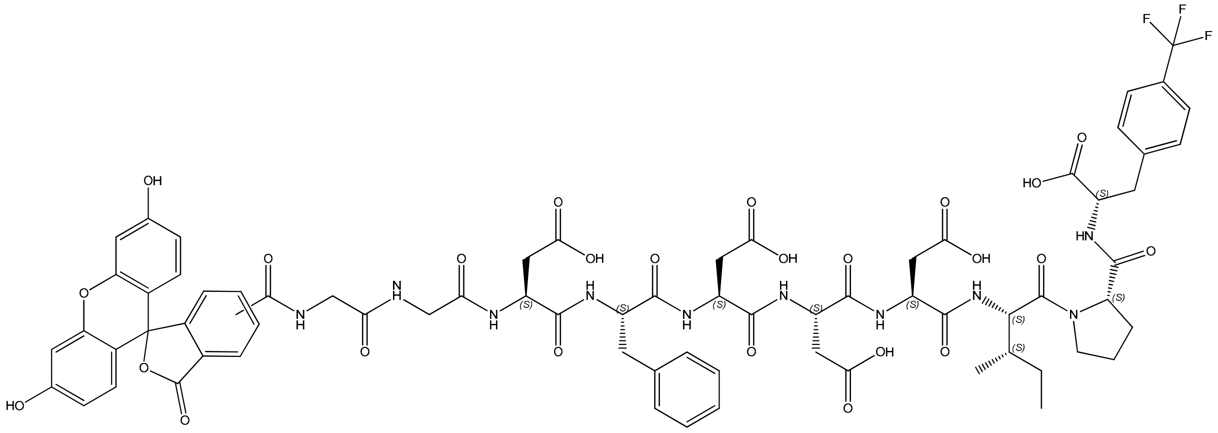

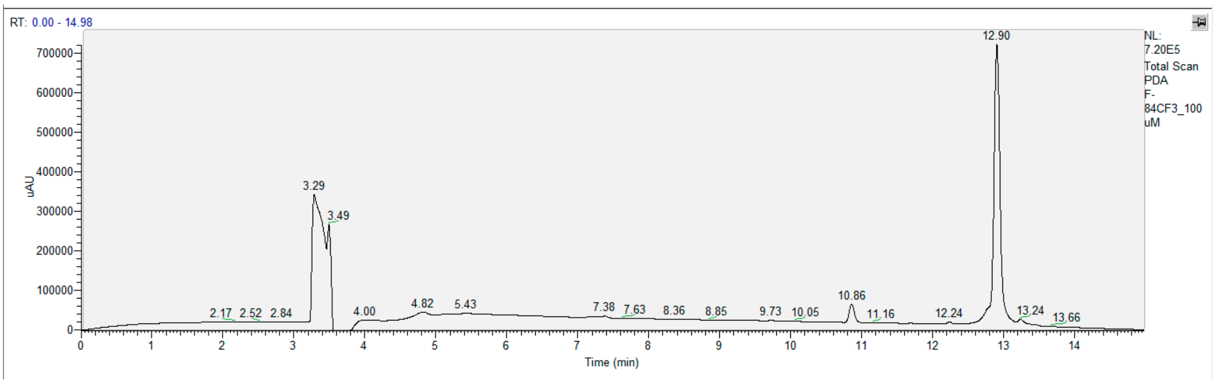

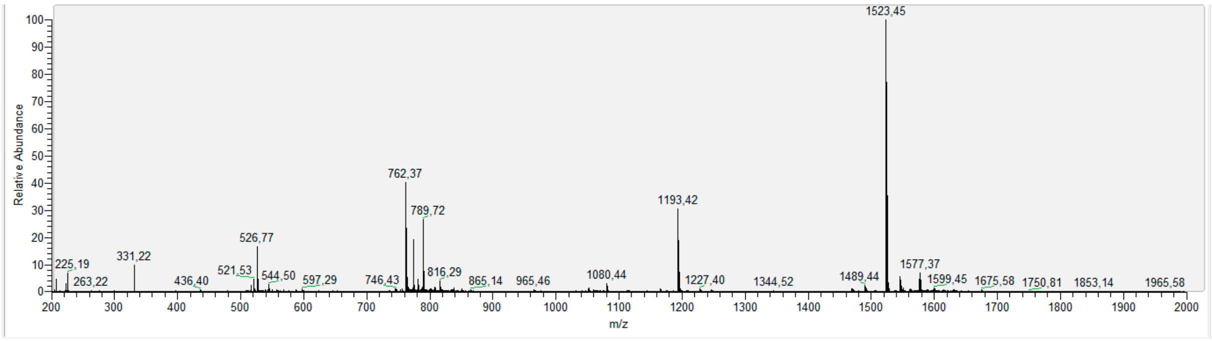

Compound 63 – E1-sSSB-Ct

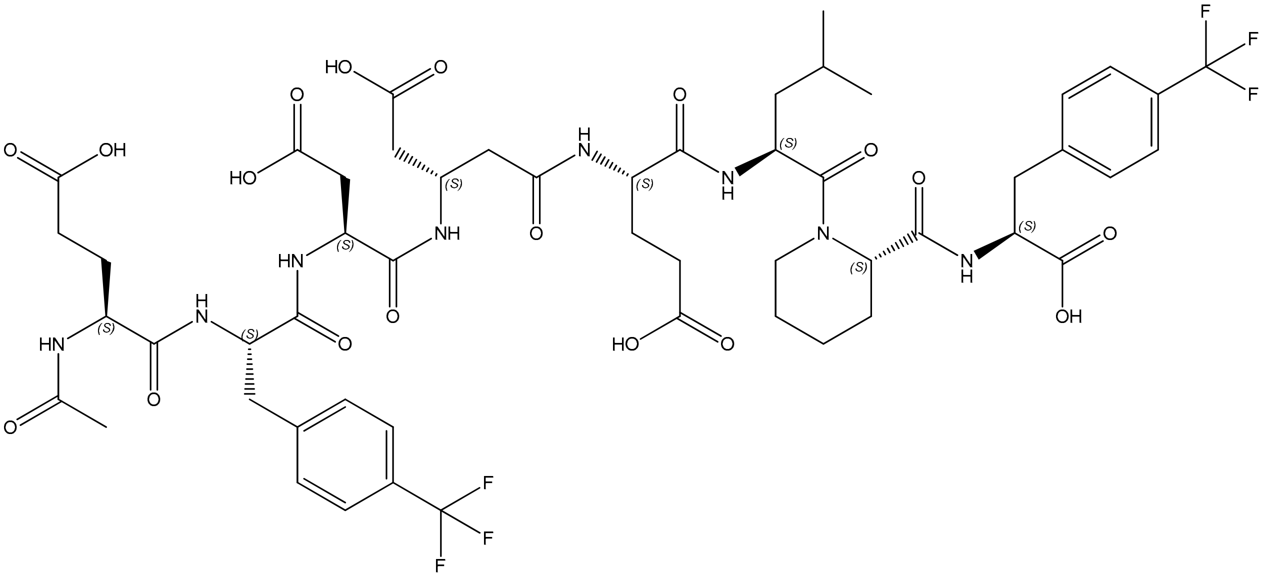

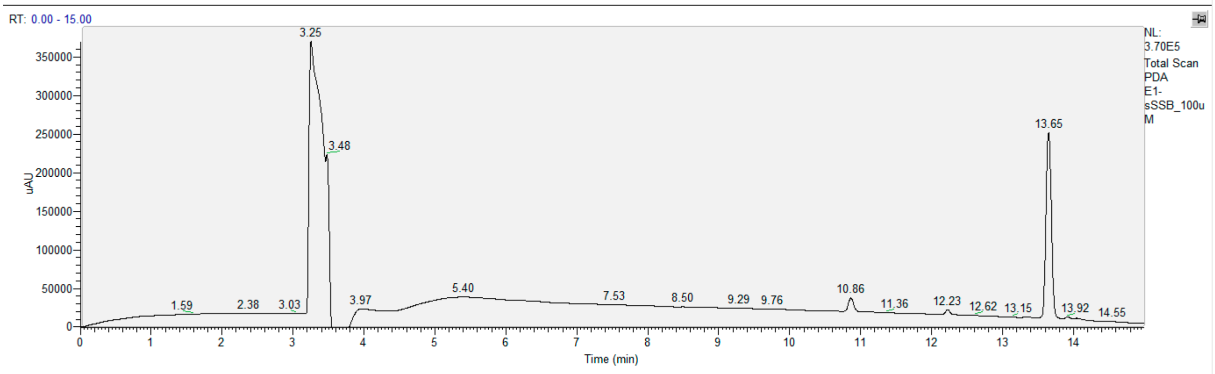

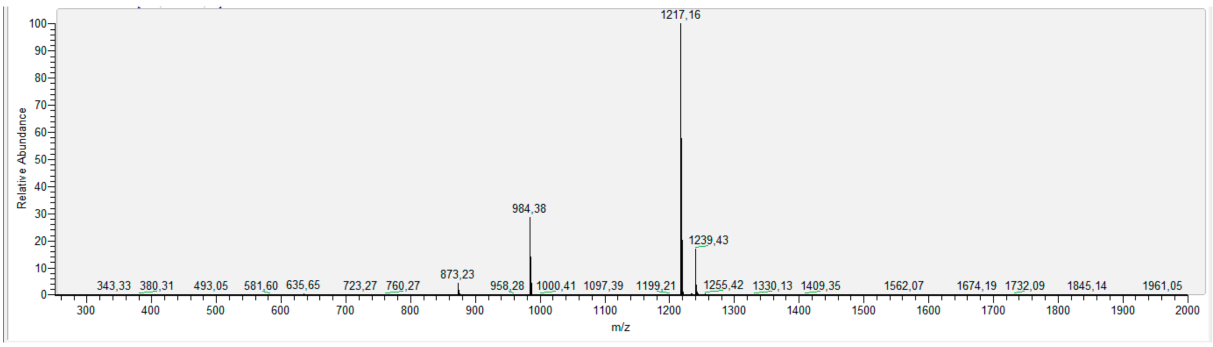

Compound 64 – E2-sSSB-Ct

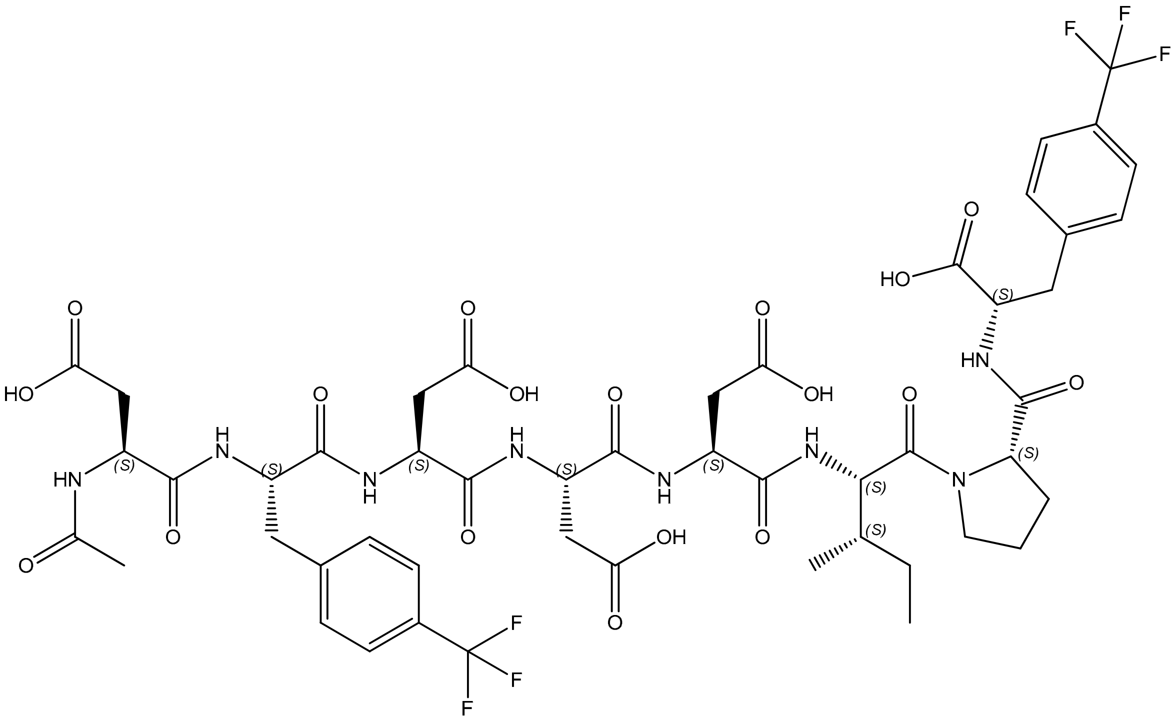

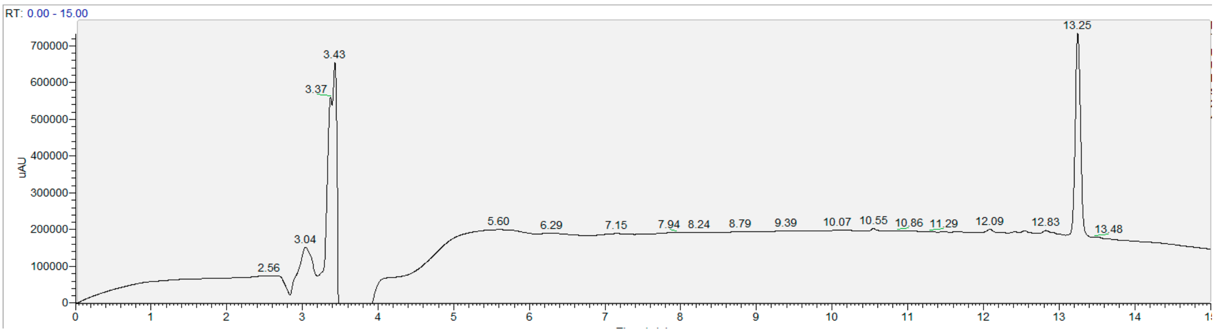

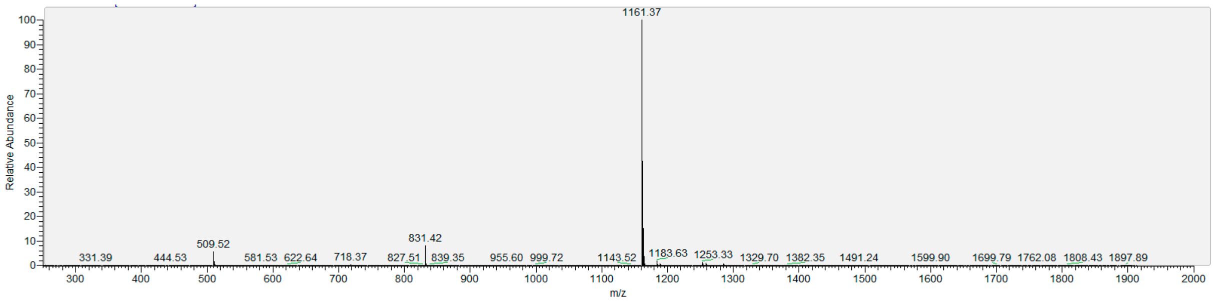

Compound 65 – R1-sSSB-Ct

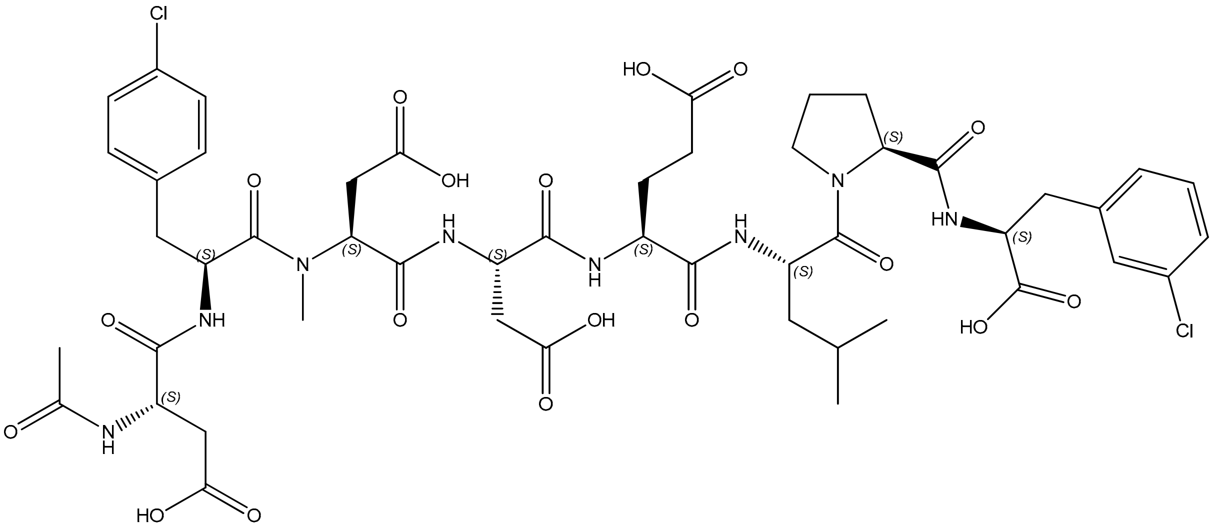

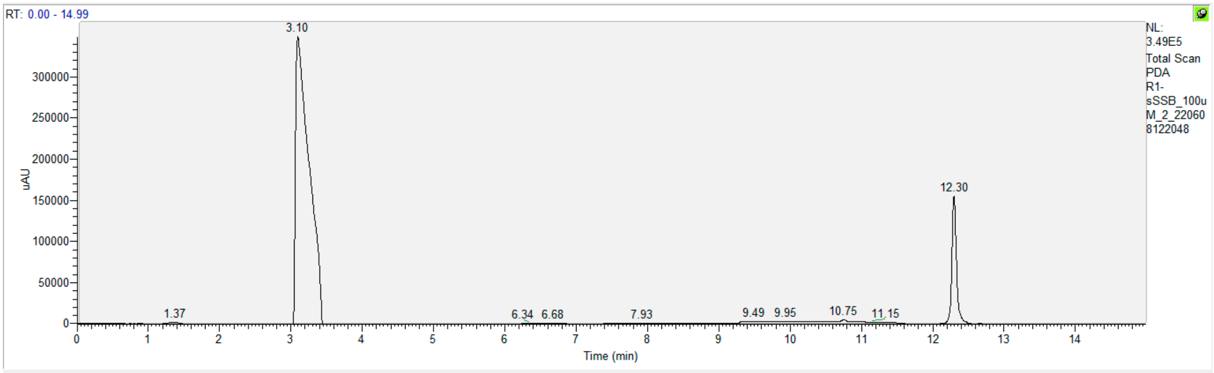

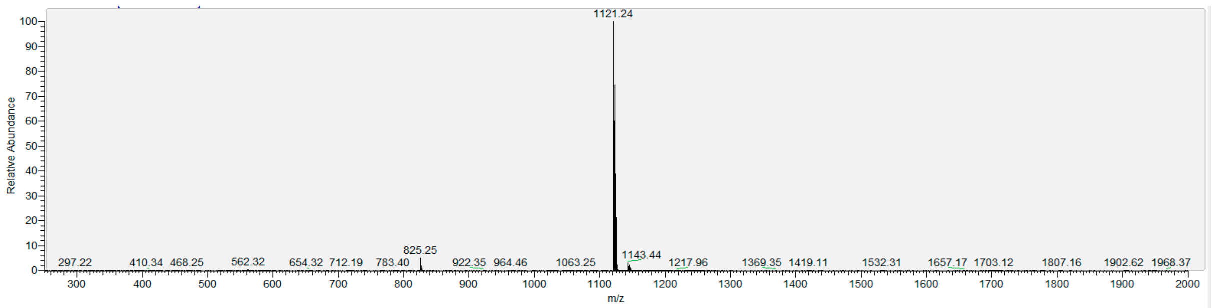

Compound 66 – R2-sSSB-Ct

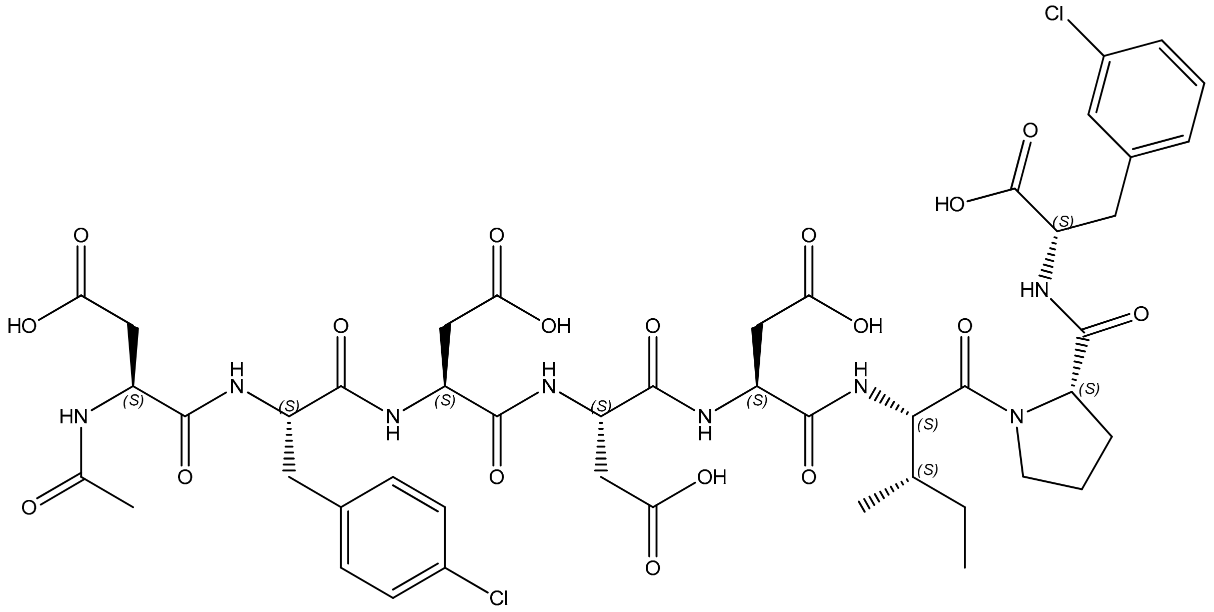

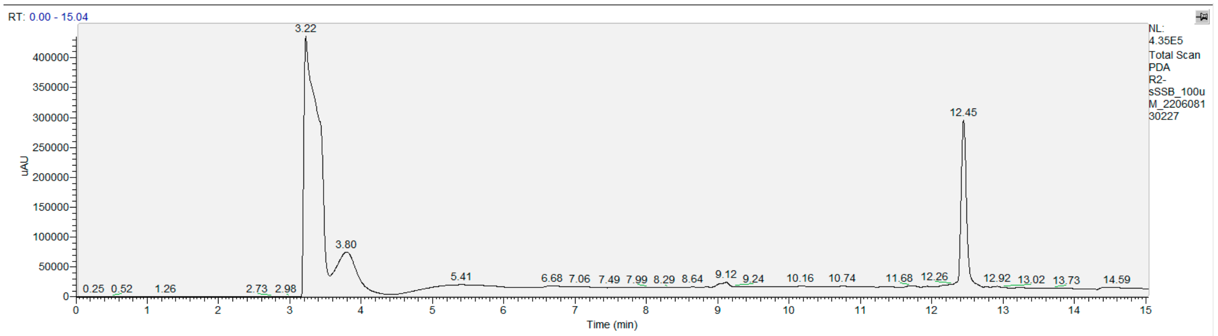

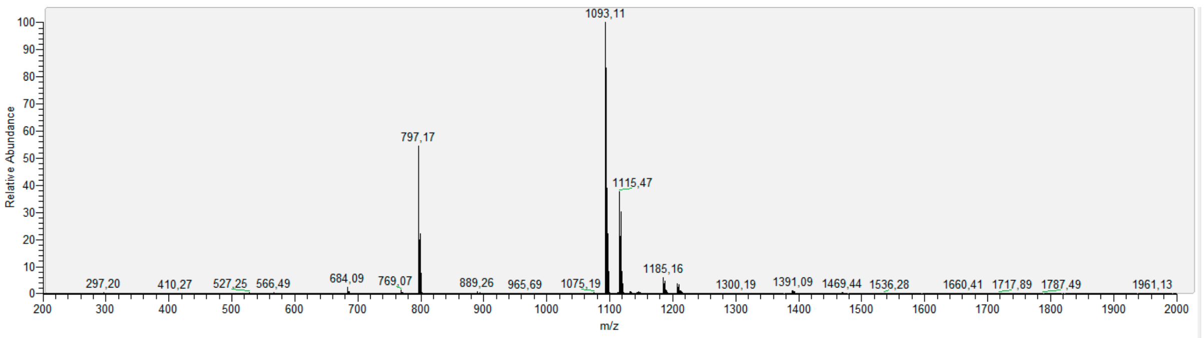

Compound 67 – K(Btn)-wtSSB-Ct

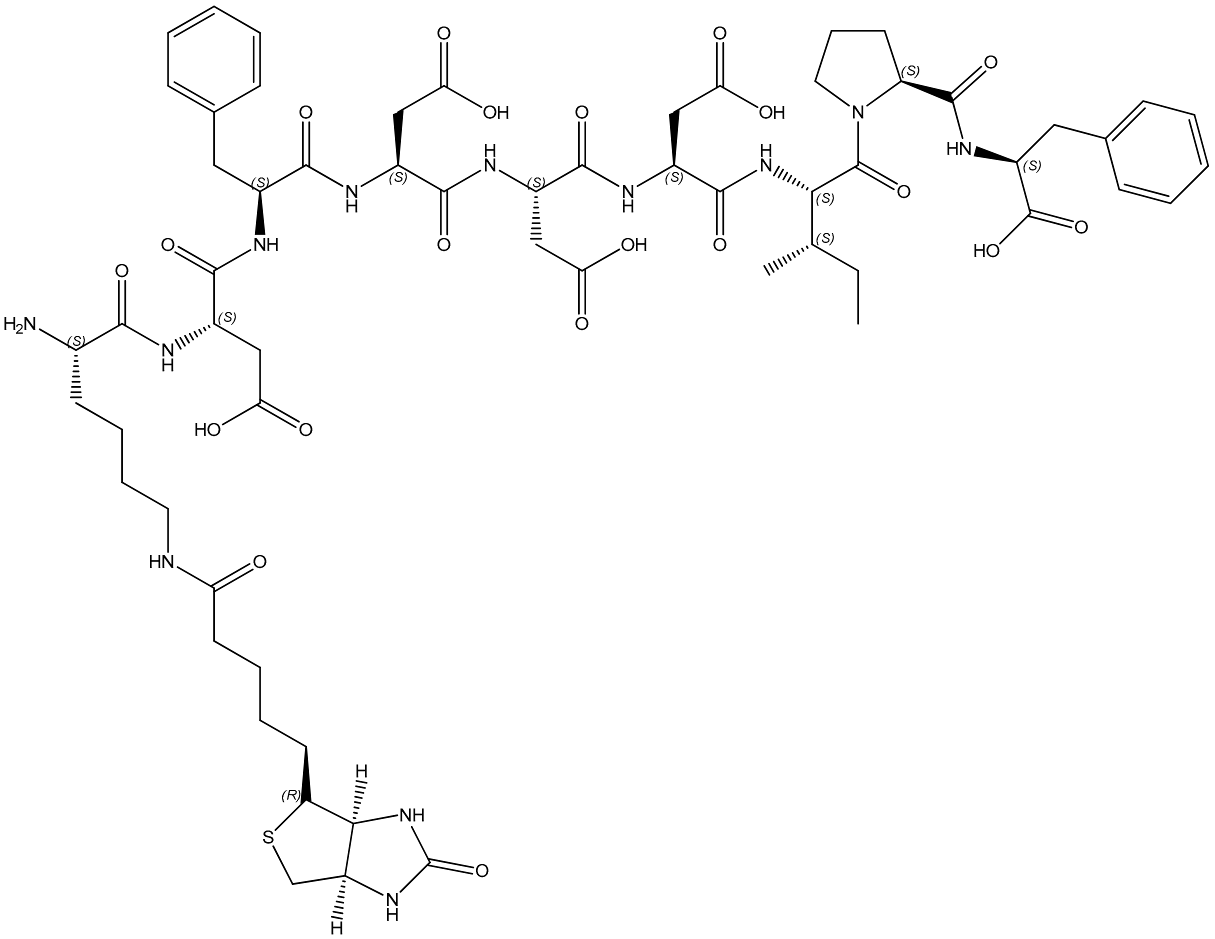

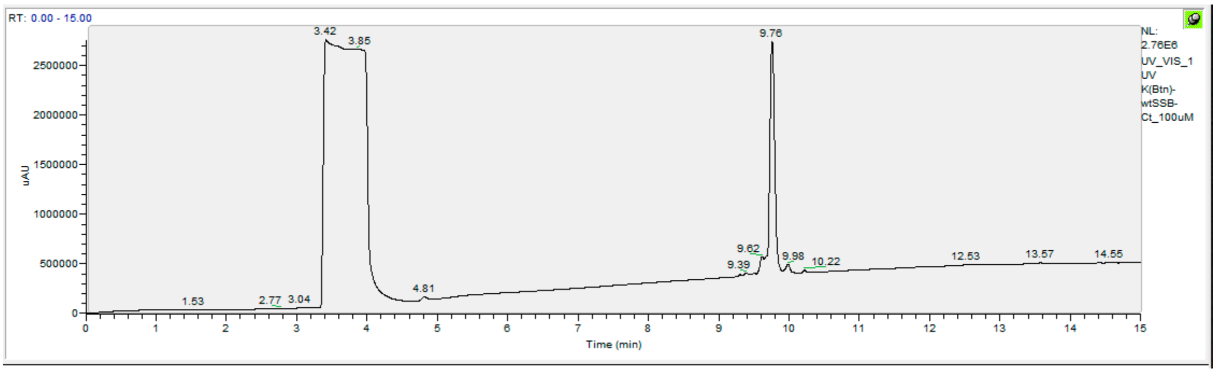

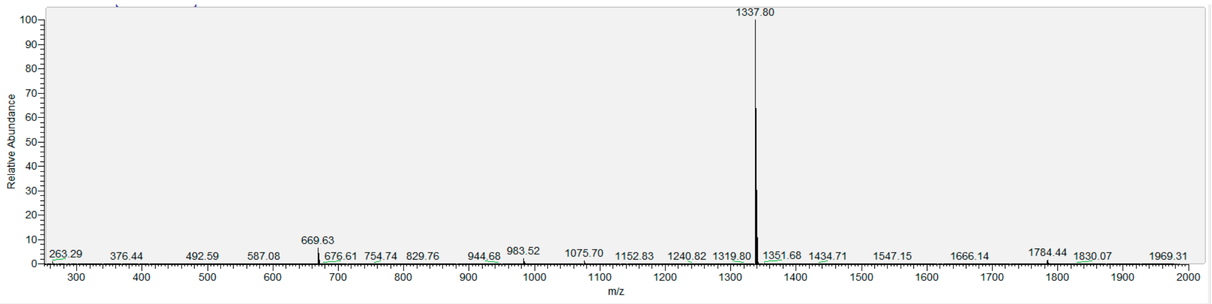

Compound 68 – K(Btn)-E1-sSSB-Ct

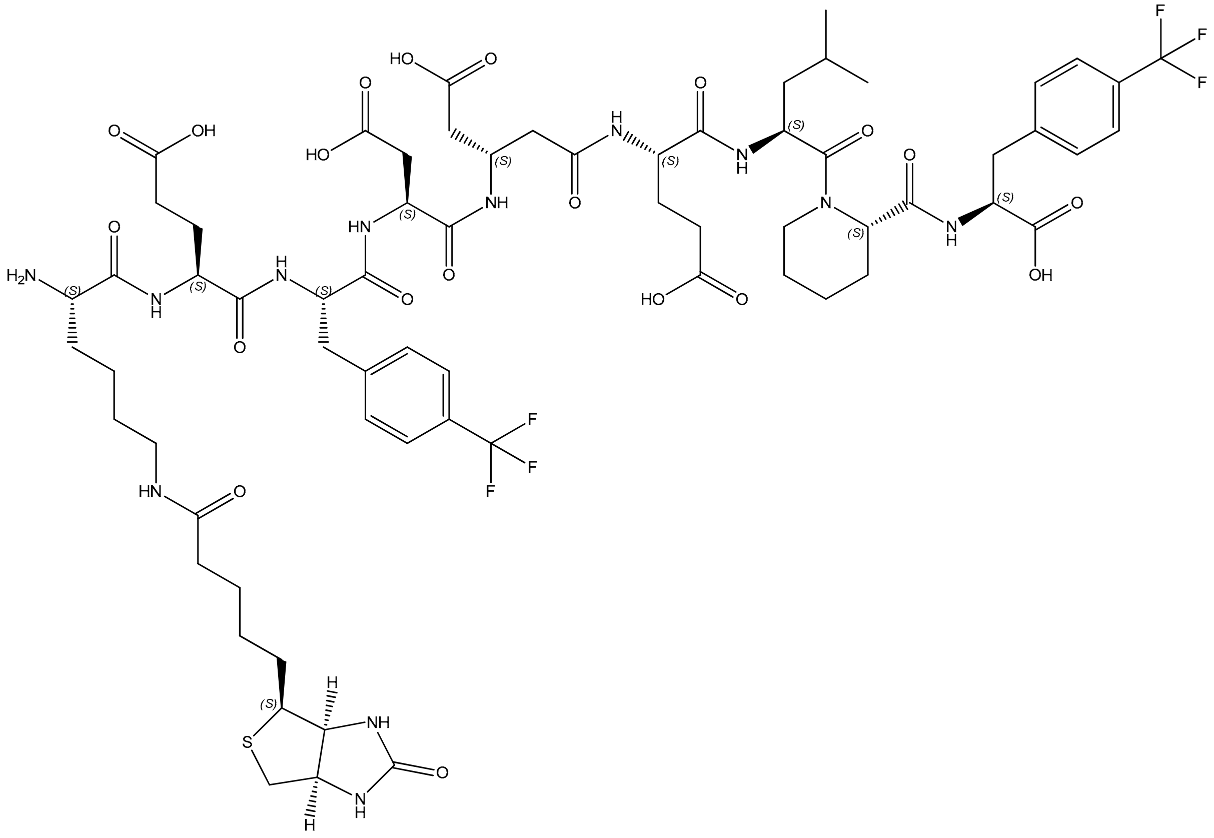

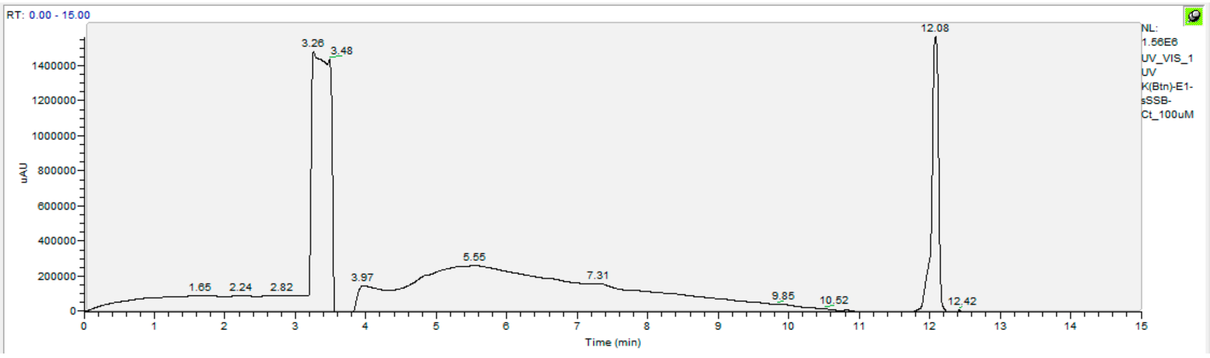

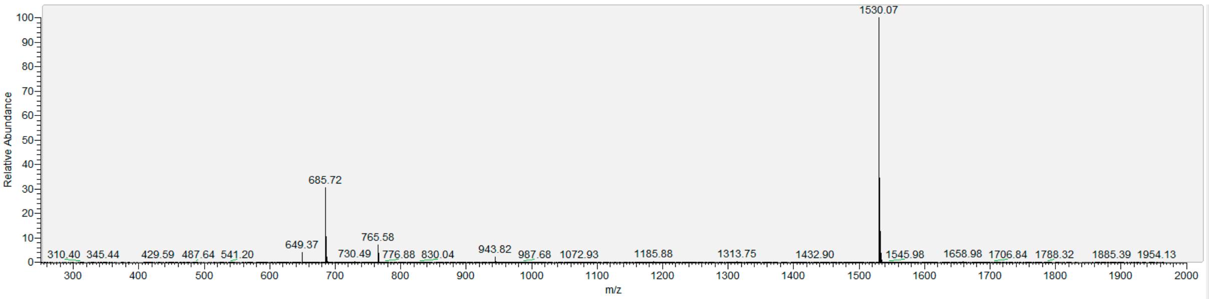

Compound 69 – K(Btn)-R2-sSSB-Ct

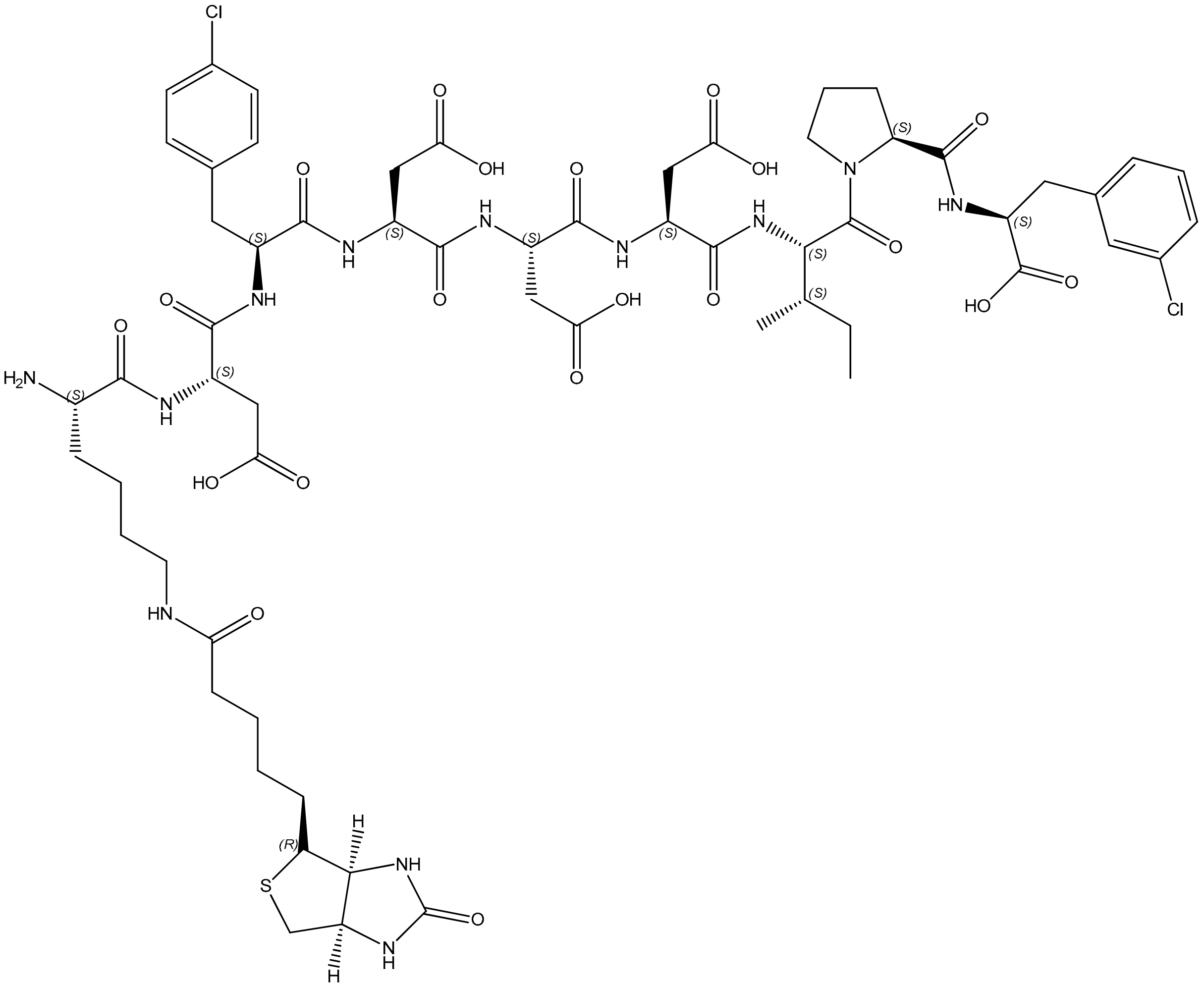

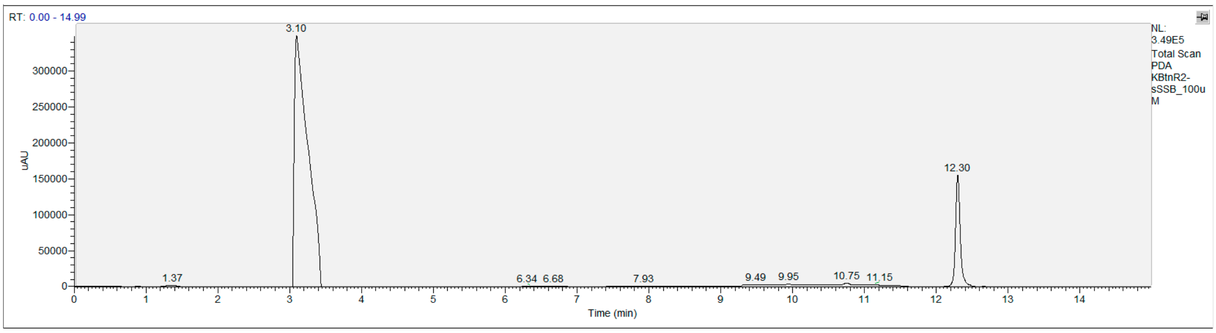

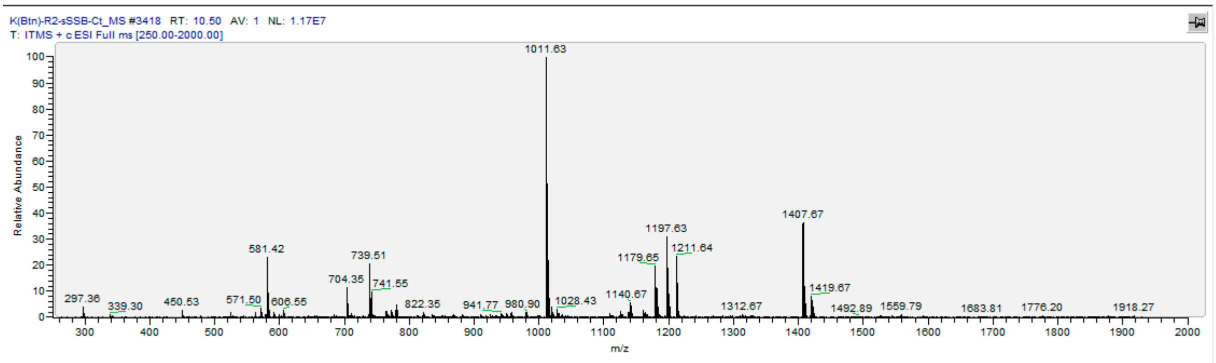

Compound 70 – Ac-DFDD-NH2

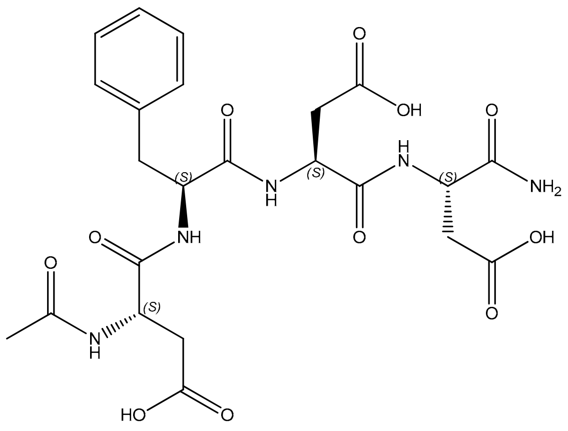

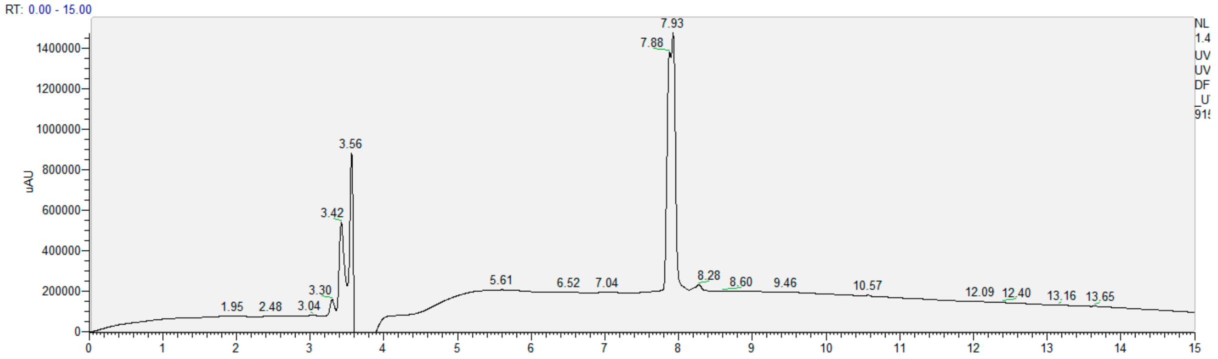

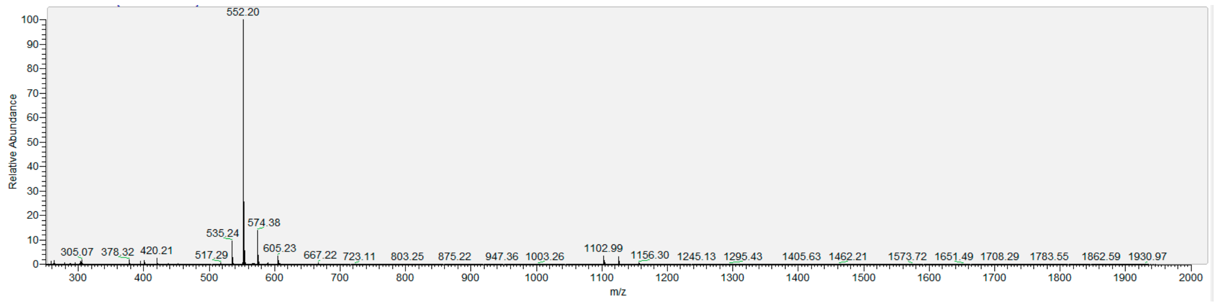

Compound 71 – Ac-DIPF-OH

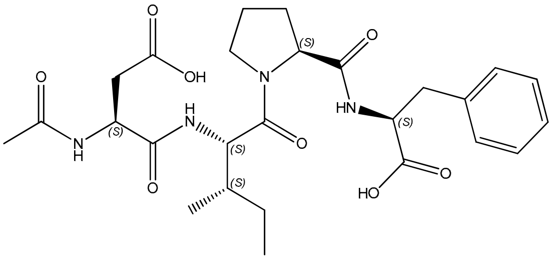

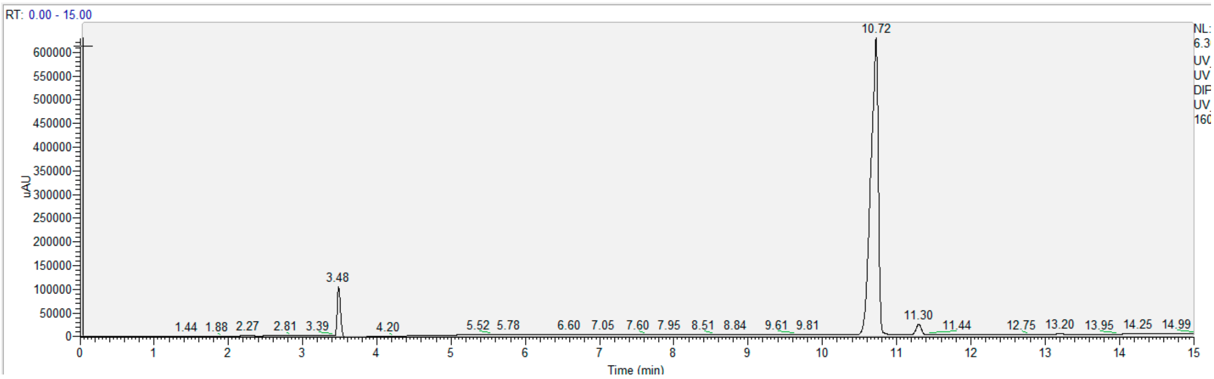

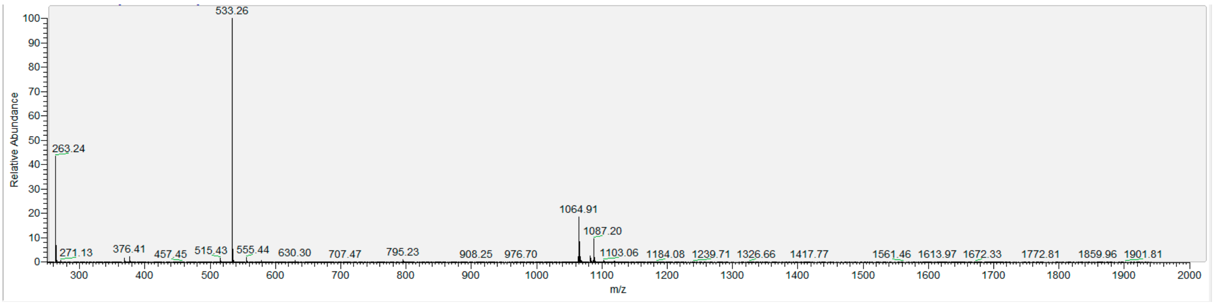

Supplement: Supplementary file 1 [file pharmaceutics-15-01032-s001.zip › pharmaceutics-2237109-supplementary.pdf]
